# Tail Wags Dog’s SINE: Retropositional Mechanisms of Can SINE Depend on Its A-Tail Structure

**DOI:** 10.3390/biology11101403

**Published:** 2022-09-26

**Authors:** Sergei A. Kosushkin, Ilia G. Ustyantsev, Olga R. Borodulina, Nikita S. Vassetzky, Dmitri A. Kramerov

**Affiliations:** Laboratory of Eukaryotic Genome Evolution, Engelhardt Institute of Molecular Biology, Russian Academy of Sciences, 119991 Moscow, Russia

**Keywords:** SINE, retroposon, retrotransposon, RNA polymerase III, transcription terminator, polyadenylation, Carnivora, Caniformia

## Abstract

**Simple Summary:**

The genomes of higher organisms including humans are invaded by millions of repetitive elements (transposons), which can sometimes be deleterious or beneficial for hosts. Many aspects of the mechanisms underlying the expansion of transposons in the genomes remain unclear. Short retrotransposons (SINEs) are one of the most abundant classes of genomic repeats. Their amplification relies on two major processes: transcription and reverse transcription. Here, short retrotransposons of dogs and other canids called Can SINE were analyzed. Their amplification was extraordinarily active in the wolf and, particularly, dog breeds relative to other canids. We also studied a variation of their transcription mechanism involving the polyadenylation of transcripts. An analysis of specific signals involved in this process allowed us to conclude that Can SINEs could alternate amplification with and without polyadenylation in their evolution. Understanding the mechanisms of transposon replication can shed light on the mechanisms of genome function.

**Abstract:**

SINEs, non-autonomous short retrotransposons, are widespread in mammalian genomes. Their transcripts are generated by RNA polymerase III (pol III). Transcripts of certain SINEs can be polyadenylated, which requires polyadenylation and pol III termination signals in their sequences. Our sequence analysis divided Can SINEs in canids into four subfamilies, older a1 and a2 and younger b1 and b2. Can_b2 and to a lesser extent Can_b1 remained retrotranspositionally active, while the amplification of Can_a1 and Can_a2 ceased long ago. An extraordinarily high Can amplification was revealed in different dog breeds. Functional polyadenylation signals were analyzed in Can subfamilies, particularly in fractions of recently amplified, i.e., active copies. The transcription of various Can constructs transfected into HeLa cells proposed AATAAA and (TC)_n_ as functional polyadenylation signals. Our analysis indicates that older Can subfamilies (a1, a2, and b1) with an active transcription terminator were amplified by the T^+^ mechanism (with polyadenylation of pol III transcripts). In the currently active Can_b2 subfamily, the amplification mechanisms with (T^+^) and without the polyadenylation of pol III transcripts (T^−^) irregularly alternate. The active transcription terminator tends to shorten, which renders it nonfunctional and favors a switch to the T^−^ retrotransposition. The activity of a truncated terminator is occasionally restored by its elongation, which rehabilitates the T^+^ retrotransposition for a particular SINE copy.

## 1. Introduction

SINEs or short interspersed elements are non-autonomous mobile genetic retroelements no longer than 600 bp that are transcribed by RNA polymerase III (pol III) (reviewed in [1]). SINEs can be found in the majority of multicellular organisms and their number in the genome can be as high as 10^6^. New SINE copies arise through reverse transcription (retrotransposition), which is mediated by the enzyme encoded in a long interspersed element (LINE) present in the same genome. All diverse SINE families originate from three classes of small cellular RNAs transcribed by pol III. The 7SL RNA gave rise to Alu in primates, including humans [2,3] as well as to B1 in rodents [4,5], and related SINEs in tree shrews [4,6,7]. Apart from that, 7SL-derived SINEs emerged independently only in hagfish [8], a relatively small group. A number of SINE families in fish, squamate reptiles, megabat, rodent, and lepidopteran genomes originated from the 5S rRNA [9,10,11,12,13]. However, the bulk of SINE families descended from tRNA and their 5′-terminal parts (heads) can usually be traced to a particular tRNA species (reviewed in [1,13,14]). Similarly to these RNA classes, SINEs are transcribed by pol III due to the internal promoter of this RNA polymerase. In the case of tRNA- and 7SL RNA-derived SINEs, the promoter includes 11-nt boxes A and B spaced by 30–40 bp. The 3′-terminal part of SINEs is critical for the recognition of their transcripts by the reverse transcriptase of LINEs [15,16]. Most placental SINEs rely on the reverse transcriptase of L1, which recognizes the poly(A) tail at the template RNA [13,17,18,19].

After emergence in the genome, a SINE family is inherited in descendant species. Similarly, individual SINE copies remain in the genomic locus indefinitely and are inherited in all species of the lineage. SINE insertions can be used as nearly homoplasy-free phylogenetic markers and the pattern of SINE presence/absence in orthologous loci can be used to evaluate phylogenetic relations between taxa. Valuable and sometimes surprising conclusions on the phylogeny of mammals and other vertebrates were obtained using this approach (reviewed in [20,21,22]).

SINEs substantially contribute to the evolution and function of genomes [1,23,24,25]. Their integration into genes including introns and regulatory regions can affect gene functioning and induce mutations [3,26]. Accordingly, a variety of cellular mechanisms repressed the transcription and subsequent retrotransposition of SINEs and LINEs [27,28]. At the same time, other SINE insertions into introns and regulatory regions can also be beneficial for gene function by modulating their transcription [29,30,31] as well as splicing [3,32,33] or polyadenylation patterns [34,35,36,37,38,39]. Pairs of SINEs in inverse orientation can promote long hairpin structures in pre-mRNA, and such double-stranded structures can give rise to siRNAs that promote silencing of other genes [40]. Pol III transcripts of SINEs can participate in cellular responses to stress [41].

Previous analyses of mammalian SINE families allowed us to reveal AATAAA and pol III transcription terminators (TCT_≥3_ or T_≥4_) preceding the adenosine-rich tail (referred to as A-tail below) in some of them [42]. These two motifs made it possible to assign such SINEs to the class designated as T^+^, while those lacking these motifs were considered as T^−^ SINEs. All T^+^ SINEs known to date are tRNA-derived and are found in placental mammals [13,42,43]. We have shown the AAUAAA-dependent polyadenylation of SINE transcripts for eight T^+^ families [44,45]. Previously, it was generally accepted that such polyadenylation is limited to pol II-transcripts, primarily mRNA. Our experiments on B2, Dip, and Ves SINEs (from the mouse, jerboa, and bat, respectively) have revealed two regions indispensable for poly(A) synthesis at the 3′-ends of their transcripts apart from the AATAAA motif (polyadenylation signal, PAS) [45]. The former (β signal) is immediately downstream of box B, while the latter (τ signal) precedes the region of AATAAA repeats. In B2 RNA, the τ signal is the binding site of the polyadenylation factor CFIm [43]. In Dip and Ves, polypyrimidine motifs act as τ signals [45]; similar polypyrimidine motifs are typical of four other T^+^ SINE families.

**Figure 1:**
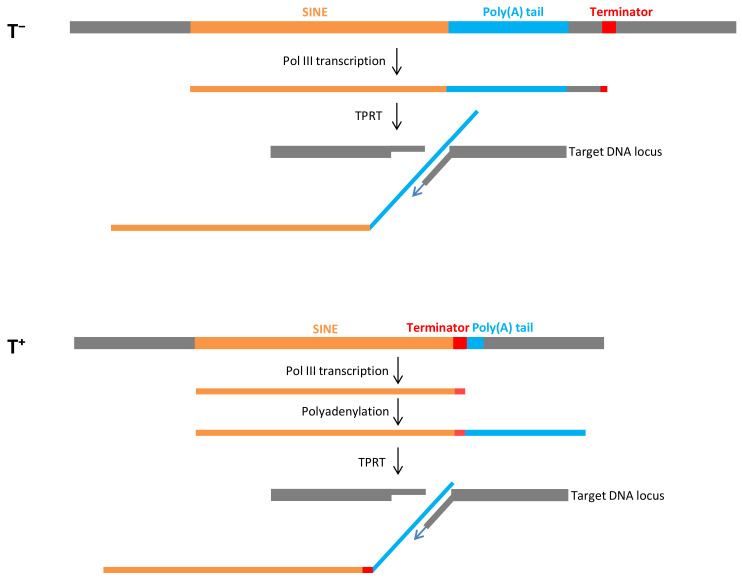
Schematic retrotransposition of SINEs via T^−^ (above) and T^+^ (below) pathways. SINEs and their poly(A) tails are shown in yellow and blue, respectively. The terminators and flanking sequences are shown in red and gray, respectively. DNA and RNA regions are not in scale.

A long poly(A) tail (A_>20_) in SINE transcripts is strictly required for retrotransposition mediated by L1 reverse transcriptase [46]. This process has been well documented for human Alu, which is a T^−^ SINE. The proposed model of Alu retrotransposition was confirmed by experimental and bioinformatics data [3,19,47,48,49]. The human genome contains a relatively small number of young Alu copies with long poly(A) tails. Pol III processes the entire sequence of such Alu copies, including the poly(A) tail, and transcription terminates at random terminators in the downstream sequence (Figure 1). It should be noted that subsequent retrotransposition can be efficient only if the terminator is close (<40 bp) to the poly(A) tail [19]. The L1-encoded ORF2 protein cleaves genomic DNA at 3′-AA↓TTTT^−^5′ site, and the TTTT binds the poly(A) tail of Alu RNA and serves as the primer for reverse transcription carried out by the same ORF2 protein (Figure 1). This process called target-primed reverse transcription (TPRT) gives rise to an Alu copy at the new genomic location.

We proposed a similar model for T^+^ SINEs such as mouse B2, which differs in the initial stages [44,45]. Pol III transcription stops at the terminator in the SINE preceding the A-tail. The resulting transcript is polyadenylated and processed by TPRT to introduce a new SINE copy (Figure 1). This retrotransposition mechanism is referred to as T^+^ as distinct from the T^−^ mechanism described above. Conceivably, the T^+^ mechanism is beneficial since it requires neither a long poly(A) tail in the parental SINE copy nor a nearby terminator in the flanking sequence. On the other hand, terminator shortening and inactivation in descendant SINE copies can be anticipated considering that pol III transcriptions can terminate before reading the entire terminator. However, we have recently found that terminators can be conserved or restored [50]. First, the transcriptional shortening of the moderate terminator TCTTT is a rare event relative to TTTT. Second, the poly(A) tails of SINE genomic copies significantly shorten with time, while the T stretches can gradually lengthen and restore the terminator’s efficiency.

Can SINE was first found in the American mink [51] and then in dogs [52] and harbor seals [53]. Later, this SINE family attributed to Caniformia [54,55,56,57,58] was also found in Feliformia, although Can sequences in cats, civets, and hyenas proved to have a small specific insertion [57,58,59,60]. One of the Can subfamilies in dogs remains retrotranspositionally active, as indicated by the presence/absence of Can copies in ~10,000 loci between dog breeds [33,61]. The Can head is most similar to lysine tRNA, which is the probable ancestor of this SINE family [56,57]. The 3′-terminal part of Can includes a long polypyrimidine motif, AATAAA repeats, pol III transcription terminators (TCT_≥3_), and an A-tail [42,54,57]. These characters assign Can to T^+^ SINEs [42], and further experiments on the transfection of HeLa cells with a Can sequence demonstrated the polyadenylation of its pol III transcripts via an AAUAAA-dependent pathway [45].

Here, we carried out a bioinformatics analysis of the Can family and its subfamilies in the genomes of dogs and other canids. The rate of new Can copies emergence was evaluated in three dog breeds, wolf, foxes, panda, and white bear. Genuine (TCT _≥3_ or T _≥4_) and rudimentary (TCT_≤2_ or T_2–3_) pol III terminators and A-tails in different Can subfamilies were given particular attention. We came to a conclusion that different Can copies can amplify by either the T^+^ or T^−^ mechanism. A model with alternating T^+^ and T^−^ retrotransposition is proposed. Cell transfection experiments using Can constructs with deletions and substitutions demonstrated that the polypyrimidine motif is essential for the polyadenylation of Can transcripts.

## 2. Materials and Methods

### 2.1. Bioinformatics Methods

Genomic data were downloaded from NCBI Genomes (https://www.ncbi.nlm.nih.gov/genome) (accessed on 15 January 2022). The following assemblies were used: domestic dog *Canis lupus familiaris* breeds: Basenji, UNSW_CanFamBas_1.2; boxer: Dog10K_Boxer_Tasha; Chihuahua long coat, ASM1132765v1; German Shepherd, UU_Cfam_GSD_1.0; Great Dane, UMICH_Zoey_3.1; Labrador retriever, ROS_Cfam_1.0; dingo *Canis lupus dingo*, UNSW_AlpineDingo_1.0; grey wolf *Canis lupus*, mCanLor1.2; African wild dog *Lycaon pictus*, sis1-161031-pseudohap; raccoon dog *Nyctereutes procyonoides*, NYPRO_anot_genome; bat-eared fox *Otocyon megalotis*, Otocyon_megalotis_TS305_17_09_2019; Arctic fox *Vulpes lagopus*, ASM1834538v1; Red fox *Vulpes vulpes*, VulVul2.2; Giant panda *Ailuropoda melanoleuca*, AilMel_1.0; Polar bear *Ursus maritimus*, UrsMar_1.0; and human *Homo sapiens*, GRCh38.p14. We used the median time at the TimeTree server (http://www.timetree.org) (accessed on 15 January 2022) as the time of species separation.

Multiple sequence alignments were generated using MAFFT [62] and edited by GeneDoc (http://www.nrbsc.org/gfx/genedoc/index.html) (accessed on 17 May 2020). We used custom Perl scripts based on the Smith–Waterman algorithm [63] to find genomic copies of SINEs with at least 65% identity and 90% length overlap with the query sequence. After all subfamilies were identified, the genome banks were successively depleted using their consensus sequences, and all hits were combined for further analysis.

We considered only ample SINE subfamilies (≥1% of the total number of full-length copies). On the contrary, tribes represent very small groups of highly similar sequences that recently amplified from a single copy. Subfamilies were identified manually and/or by a domestic script SubFam described elsewhere [50]. The mean similarity was determined for ~300–500 randomly selected sequences using the alistat program (Eddy S., Cambridge, 2005). The proportion of TSDs was determined by an original algorithm (TSDSearch described at https://sines.eimb.ru/) (accessed on 30 January 2022).

SINE insertion/deletion loci across genomes were identified by mapping ~200 bp flanking regions of each SINE-containing locus using BWA-MEM [64]; matches were analyzed using various tools including SeqKit [65] and BEDtools [66]. The presence or absence of a SINE was inferred from the locus size and manually verified.

Young Can copies were identified as sequences present in the German Shepherd genome but missing in orthologous wolf loci (for Can_b1) or Great Dane (for Can_b2). Young highly similar copies (tribes) were found in the German Shepherd, Great Dane, and Boxer genomes (canFam4, canFam5, and canFam6, respectively) using the BLAT search at the UCSC server (http://genome.ucsc.edu/cgi-bin/hgBlat) (accessed on 1 March 2022) [67] or the Smith–Waterman algorithm [63].

### 2.2. Experimental Methods

A number of plasmid constructs with long deletions or nucleotide substitutions were generated to reveal the regions in Can SINE essential for polyadenylation of its transcripts and transfected into HeLa cells. The efficiency of Can polyadenylation was evaluated by hybridization of RNA isolated from transfected cells with Can-specific probe. The primary Can transcript was visible as a band, while polyadenylated transcripts appeared as a smear above this band on an autoradiograph. The polyadenylation efficiency was evaluated as the proportion of the smear signal relative to the total signal (band + smear).

The construct with the original SINE copy (Can-T) and the one with both PASs inactivated by T to C substitutions (Can-C) were described previously [45]. All other constructs were obtained using the Phusion Site-Directed Mutagenesis Kit (Thermo Fisher Scientific, Waltham, MA, USA) and Can-T plasmid as the PCR template. The plasmids designed for transfection were isolated by the NucleoBond PC 100 kit (Macherey-Nagel, Dylan, Germany).

HeLa cells (ATCC and CCL-2) were grown to an 80%-confluent monolayer in 60 mm Petri dishes using DMEM with 10% fetal bovine serum. Cells on one plate were transfected by 5 μg of plasmid DNA mixed with 10 μL of TurboFect reagent (Thermo Fisher Scientific, Waltham, MA, USA) according to the manufacturer’s protocol. The cellular RNA was isolated 20 h after transfection using the guanidine-thiocyanate method [68] and further purified by RNase-free DNase I treatment. RNA samples (10 μg) obtained after each transfection were separated by electrophoresis in 6% polyacrylamide gel with 7M urea. Blotting and Northern hybridization with Can-specific probe labeled by α[^32^P]-dATP were carried out as described previously [45]. Hybridization signals were quantified by scanning the membranes in a Phosphorimager (Image Analyzer Typhoon FLA 9000; GE Healthcare Bio-sciences, Uppsala, Sweden).

## 3. Results and Discussion

### 3.1. General Description of Can SINE Family

The study was initiated by an exhaustive computer search for Can copies in the genomes of two dog breeds (Basenji and Boxer), wolf (*Canis lupus*), African wild dog (*Lycaon pictus*), raccoon dog (*Nyctereutes procyonoides*), bat-eared fox (*Otocyon megalotis*), and Arctic fox (*Vulpes lagopus*). The total number of genomic Can copies proved similar in all genomes (6.4–6.7 × 10^5^) except in *O. megalotis* (7.7 × 10^5^), which can be attributed to the proportionally large genome size in this fox. Can copies could be divided into four discernible subfamilies in all these genomes: a1, a2, b1, and b2 (Table 1). The proportion between subfamily copies, mean sequence similarity within subfamilies, and the proportion of copies with target site duplications (TSDs) are also similar across these seven genomes. Based on the two latter parameters, one can propose that a1 and a2, which amount to one-third of Can copies, are the oldest. b1 and, in particular, b2 subfamilies are much younger with a mean sequence similarity of 77 and 90%, respectively.

**Figure 2:**
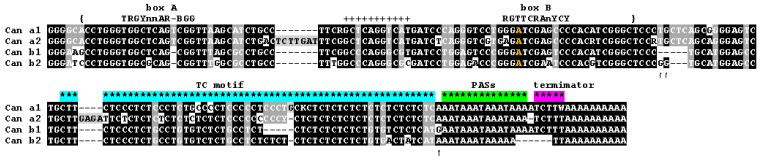
Consensus nucleotide sequences of four Can SINE subfamilies in canids. Consensus sequences of box A and box B of pol III promoter are shown above the alignment. The A nucleotide in the box B differing from the canonical consensus is given in orange. The TC-motif, polyadenylation signals (PASs), and pol III transcription terminator are indicated by asterisks of different colors. The boundaries of the tRNA-related sequence are marked by curly brackets. Plus signs indicate the insertion relative to lysine tRNA. Arrows indicate the distinctive characters of b1 and b2 subfamilies.

Consensus sequences of the Can subfamilies do not differ too much (Figure 2). Can_a2 features two small insertions 7 and 4 bp in length. As mentioned above, the Can head apparently originates from lysine tRNA, but Can SINE includes an extra 11 bp region (marked by plus signs in Figure 2) that is missing in the ancestral tRNA. Noteworthily, Can subfamilies have adenosine at position 3 of box B instead of the canonical thymidine (Figure 2), which is quite rare in both tRNA genes and SINEs [13]. Notice that the lysine tRNA gene and Can SINE from cats and civets have T at this position as distinct from A in Caniformia [57]. This T to A substitution should decrease the pol III promoter strength, and its fixation can be attributed to regulatory aspects of such promoter.

All Can subfamilies have a long (45–60 bp) polypyrimidine motif consisting mainly of T and C residues (referred to as TC-motif hereafter). As shown below (Section 3.6), the TC-motif is critical for the polyadenylation of Can transcripts synthesized by pol III. The tRNA-derived region and the TC-motif are spaced by a short region, 21 bp in Can_a and 16 or 18 bp in Can_b. This distance in other SINEs with a TC-motif is much longer, e.g., 62 and 76 bp in Ves and Dip, respectively [50].

In all Can subfamilies, the TC motif is followed by two or three AAAT tandems that introduce overlapping polyadenylation signals AATAAA (Figure 2). Previously, we demonstrated that the PAS is essential for the polyadenylation of Can transcripts synthesized by pol III [45]. The very end of Can consensus sequences has pol III transcription terminators (or their rudiments) and an A-tail (Figure 2). The terminator sequences are represented by TCTTT in the a1, a2, and b1 subfamilies (similar to mouse B2 or rabbit C SINEs), while the Can_b2 consensus has a TT dinucleotide, which cannot terminate transcription. Below we consider in detail the terminators and oligo(A) tails in Can copies from different subfamilies in the context of their retrotranspositional activity.

It is worth explaining why we identified Can subfamilies in Canini and Vulpini rather than used consensus sequences from Repbase. This database includes 19 records of Can sequences with different names (CAN, SINEC*, MVB2) and origins; some of them are highly similar. Our subfamilies are discernible by relatively large indels and are easy to use. For reference, our subfamilies have the following closest counterparts: **Can_a1**, SINEC1D_CF; **Can_a2**, SINEC_c1; **Can_b1**, SINEC1_CF/SINEC1A_CF/SINEC1B_CF/SINEC1C_CF; and **Can_b2**, SINEC2_CF.

### 3.2. Analysis of Retrotranspositional Activity of Can Subfamilies

**Figure 3:**
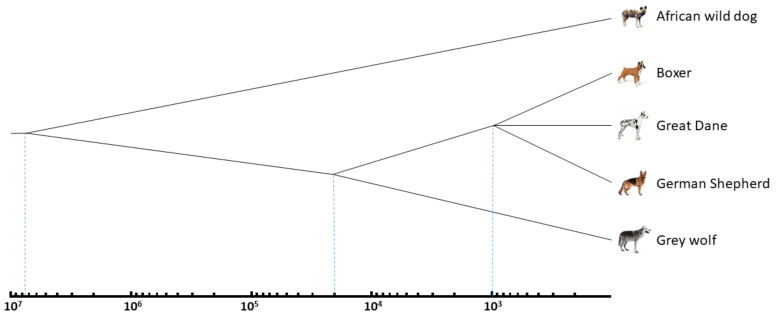
The evolutionary relationships and divergence times of the African wild dog (*Lycaon pictus*), grey wolf (*Canis lupus*) and modern dog breeds. Time is shown on a logarithmic scale. See the text and Table 2 for other explanations and references.

We revealed currently active Can subfamilies by pairwise comparisons of three dog breed genomes (German Shepherd, Great Dane, and Boxer), grey wolf, and African wild dog (their evolutionary tree and divergence times are shown in Figure 3); thus, the copies present in one genome but missing at orthologous sites of other genome(s) were identified. For instance, the German Shepherd genome has 12,074 Can copies missing in the Great Dane, while the latter has 5508 copies absent in the corresponding loci of the German Shepherd (Table 2).

These data agree with the recent comparison of the Great Dane and German Shepherd genomes by Halo et al. [69]. Table 2 also presents similar comparison data for the genomes of the grey wolf and the same three dog breeds. In all cases, the number of wolf-specific Can copies ranged from 7856 to 14,109 between the dog genomes; i.e., it was only marginally larger than the number of specific dog-vs.-dog Can copies.

**Figure 4:**
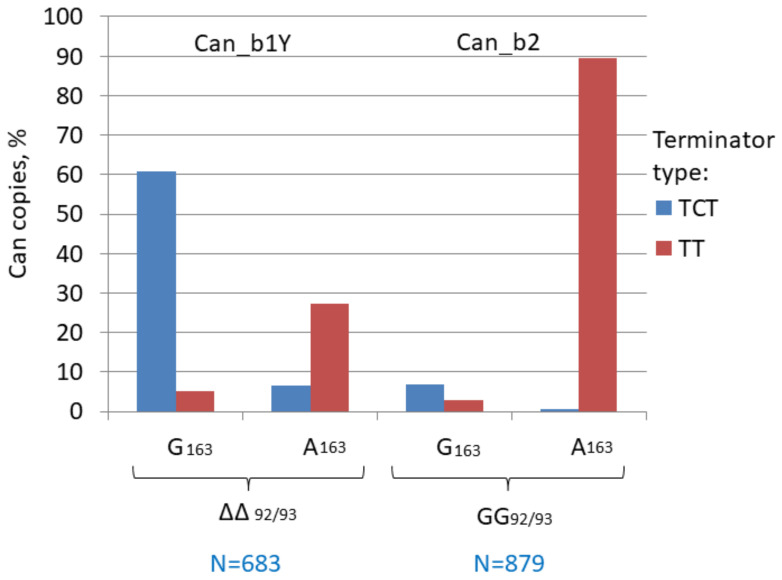
Distribution of TCT and TT terminators among copies of two young Can variants (b1Y and b2). The Can copies present in the German Shepherd genome but missing in the wolf orthologous loci were selected. Can_b2 sequences were distinguished by the GG insertion between Can_b1 positions 92/93 (designated as GG and ΔΔ, respectively). A less definite marker was G or A in position 163 in Can_b1 and Can_b2, respectively. TCT refers to the major TCTTT as well as to TCT, TCTT, and TCT _>3_. TT refers to TT, TTT, and T _>3_.

We analyzed the distribution of German Shepherd copies missing in the wolf genome between the Can subfamilies, i.e., recent copies that emerged after dog domestication in the last 20,000 years [70]. The majority of copies belong to the Can_b2 subfamily and as low as 5–7% could be assigned to Can_b1; no Can_a1 and a2 were found. It should be noted that Can_b1 and b2 sequences are not always easy to discern and we used the GG insertion at positions 92/93 in Can_b2 as the distinctive character (Figure 2). An analysis of young Can_b1 copies (Can_b1Y) demonstrated that most of them had G at position 163 and TCT terminators similar to Can_b1 (Figure 4). Thus, Can_b1Y is a small particular group of Can_b1 that is still retrotranspositionally active in dog genomes. Conceivably, the Can_b2 subfamily evolved from Can_b1 via an intermediate such as Can_b1Y.

Next, we analyzed other Caniformia species to identify and quantify orthologous species-specific Can copies. The compared species pairs (African wild dog vs. grey wolf, red fox vs. Arctic Fox, and giant panda vs. polar bear) diverged much earlier, millions of years ago (Mya) compared to the dog-wolf divergence (20 thousand years ago). The number of species-specific Can copies amounted to dozens of thousands in the compared genome pairs (Table 2). The mean rate of specific copies emergence proved similar in these species, 3-9 × 10^3^ copies/million years (My). The corresponding rates for the wolf/dog genomes were two orders of magnitude higher, at an average of 5 × 10^5^ copies/My (Table 2). The rates for dog breed pairs were even 10–20 times higher (Table 2).

This amazing difference can be attributed to an extreme activation of Can retrotransposition in the course of dog domestication and breeding. Many researchers relate retrotransposon activity to genome instability (e.g., [71,72]) or even consider their activity as a speciation factor [73,74,75]. Arguably, dog breeding is a special case of speciation. Speciation can result from genome instability that has been caused by bottlenecks and founder effects [76]. Clearly, bottlenecks occurred in early dog domestication as well as in recent dog breeding [77,78]; likewise, the wolf passed a bottleneck 15–40 thousand years ago when its population reduced to about 250 animals [79]. Whatever the causal relations, the correlation between retrotransposon activity and speciation/genome instability/bottlenecks is apparent. A similar line of reasoning was used by Hedges et al. (2004) to explain a 2.2 higher rate of Alu emergence between the human and chimpanzee [80].

One more factor can contribute to the different emergence rates of Can copies. Low rates (3–9 × 10^3^ copies/My) were observed for species that diverged relatively long ago, 3.6–17 Mya (Table 2). In the long run, certain de novo Can copies are fixed while many others are gradually lost in population. In this context, the emergence rates of new Can copies should be compared in the genomes of recently diverged lineages (such as dog breeds and wolf) rather than those diverged million years ago. If this is the case, there had been no huge acceleration of Can retrotransposition in the lineages of dogs and wolf. Further studies are required to clarify the relative significance of these factors (increased amplification rate and loss of unfixed copies).

### 3.3. Analysis of Pol III Terminators and Poly(A) Tails

**Figure 5:**
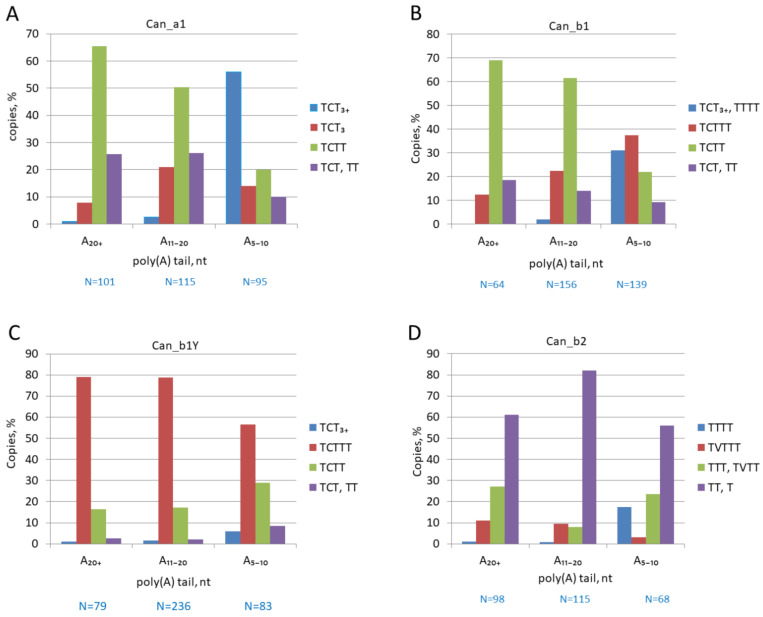
Distribution of pol III terminators or their rudiments among Can copies with poly(A) tails of different lengths. (**A**) Can_a1 subfamily; (**B**) Can_b1 subfamily; (**C**) Can_b1Y, young b1 copies; (**D**) Can_b2 subfamily. The number of analyzed Can copies is indicated below as N = number. V in terminator sequences corresponds to C, A, or G.

The 3′-terminal regions of Can copies from different subfamilies were analyzed in random samples of copies with long (A_>20_), medium (A_11–20_), or short (A_5–10_) poly(A) tails. The proportion of copies with strong (TCT_>3_), moderate (TCTTT), and rudimentary (TCTT and TCT) terminators was evaluated in each sample. Figure 5A demonstrates that as low as 10 and 24% of Can_a1 copies with long and medium poly(A) tails, respectively, have functional terminators, while other copies have rudimentary ones, primarily, TCTT. Conversely, about 70% of Can_a1 with short A-tails have functional terminators; the incidence of strong terminators in such copies is at least 20 times that in copies with long and medium poly(A) tails (Figure 5A). This pattern agrees with our previous data on B2, Dip, and Ves SINEs [50] and can be interpreted as follows. Over the long period of SINE copies’ existence in the genome, their poly(A) tails gradually shorten, which is accompanied by elongation and strengthening of their pol III promoters. Terminator elongation becomes possible only after the A-tail becomes shorter than 10 bp. The mechanism of terminator elongation remains unclear. Conventionally, a long poly A-tail is an indication of a relatively recent emergence of the corresponding SINE copy. However, the Can_a1 subfamily is old and transpositionally inactive for a long period of time, while a fraction of Can_a1 copies (~3%) have long poly(A) tails. We believe that such copies are protected from A-tail shortening due to some reasons, but no terminator elongation is observed in them. This suggests that the direct relationship between the terminator elongation and poly(A) tail shortening becomes less certain in old copies. A similar pattern was observed for old Dip and Ves SINE copies [50].

The Can_b1 subfamily is younger than Can_a1 but still old and largely inactive. Can_b1 copies with poly(A) tails of different lengths demonstrate a distribution of terminators similar to that in Can_a1 (Figure 5B). Most Can_b1 copies with long and medium poly(A) tails have rudimentary terminators (TCTT and TCT), while ~70% of copies with short A-tails have functional terminators. This agrees with our interpretation of the results for Can_a1. A different pattern is observed for Can_b1Y copies, which emerged in the dog genome after the split from the wolf (see above and Figure 3). In this case, the medium and long poly(A) tails (A_≥11_) indeed demonstrate the young age of such copies, which constitute the majority (79%) of Can_b1Y (Figure 5C). About 80% of these have functional although moderate terminators (TCTTT) rather than rudimentary ones as in Can_b1. This corroborates with the retrotranspositional activity of Can_b1Y. The incidence of strong terminators (TCT _>3_) was five times higher in the copies with short A-tails compared to those with long ones; yet, their proportion is as low as 6% (Figure 5C). This can be attributed to their recent emergence (less than 20 thousand years ago); not enough time has passed for a significant shortening of their A-tails and an elongation of their terminators.

For reference, we performed a similar analysis for a sample of a Can subfamily in the giant panda *Ailuropoda melanoleuca* (SINEC1_Ame), which emerged after the panda split from the lineage of the polar bear *Ursus maritimus* (about 17 Mya). Despite the significant difference in the activity periods, such relatively young copies (SINEC1_Ame_Y) in the panda genome are counterparts of the dog Can_b1Y: the terminators in these SINE families largely include TCT, and the terminator distribution patterns are also similar. About 65% of SINEC1_Ame_Y copies have moderate terminators TCTTT (Figure S1); 12% of copies with short A-tails (A_≤10_) have strong terminators (TCT_>3_), which is five times that in the copies with longer poly(A) tails. Thus, both SINEC1_Ame_Y and Can_b1Y demonstrate a high probability of terminator elongation after A-tail shortening.

Finally, a similar analysis was performed for Can_b2, the youngest and most active Can subfamily in the genomes of dogs and related species (Figure 5D). Significantly, only a small fraction of Can_b2 has TVTTT terminators (where V corresponds to C and rarer to A or G) while other (rudimentary) terminators are composed of Ts. Among the copies with long and medium poly(A) tails, the incidence of TVTTT terminators is ~10%, and strong terminators (T_≥4_) are nearly absent. At the same time, the incidence of strong terminators amounts to 18% in the copies with short A-tails (Figure 5D). Thus, the terminator elongation accompanying the shortening of tails with less than 10 As is also observed in Can_b2 copies.

### 3.4. Analysis of Individual Active Can Copies

Here, we analyzed the terminators and A-tails in young active Can copies to identify the pathway (T^+^ or T^−^, Figure 1) of their retrotransposition in dog genomes. We started from Can_b1Y copies found in the German Shepherd genome but that were missing in orthologous wolf loci. Figure S2A exemplifies such a copy (chr1:10744779) with a TCTTT terminator and an A_46_ tail; this copy is absent from Great Dane and Boxer genomes, which indicates its recent emergence. Figure S2A also presents 24 copies identical (not counting the A-tail) to the chr1:10744779 copy representing its closest relatives. Eleven of these copies are found in the genomes of all three dog breeds; hence, one of them could be an ancestor of other 14 copies found in one or two breeds. Most of these 11 copies had shorter A-tails (A_15_ on average) than the descendant copies (with A_23_ on average). This pattern generally agrees with the T^+^ retrotransposition of these copies (Figure S2B). However, three putative parental copies have relatively long tails, A_22_ and A_24_. While unlikely, these could be parental copies for T^−^ retrotransposition.

**Figure 6:**
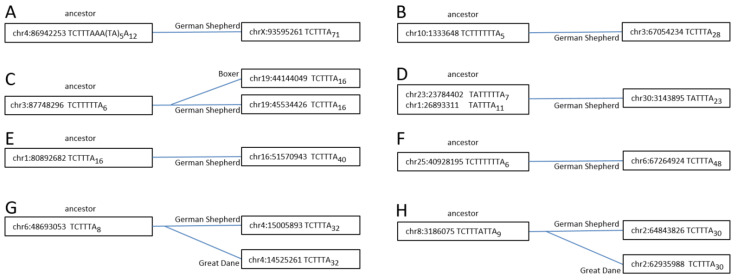
Eight Can_b1Y copies (**A**–**H**) illustrating their retrotransposition via T^+^ mechanism. The left coordinate, terminator structure, and poly(A) tail length are specified. The left (“ancestor”) boxes include copies present in the genomes of the German Shepherd, Great Dane, and Boxer; the right boxes include daughter copies present in one or two dog breeds. Notice that the daughter copies have much longer poly(A) tails; in three cases (B, C, and F), the terminators are significantly shortened in daughter copies relative to parental ones.

To reach firm conclusions, several Can_b1 copies present in the German Shepherd but missing in the Great Dane and/or the Boxer were selected. Their parental copies were identified by identical sequences and presence in the genomes of all three breeds. There were single copies that could be parental for each of selected samples; in addition, all such copies had short A-tails (A_6–16_). The nucleotide sequences of the parental and daughter copies are presented in Figure S3, and the structures of their terminators and A-tails are given in Figure 6, demonstrating the substantial elongation of the daughter poly(A)-tails (A_16–48_) relative to parental ones.

Moreover, whenever the parental terminators were long (e.g., TCT_5–6_), they were shortened to TCT_3_ in their descendants. Overall, this indicates that the pol III transcription of the parental copies stopped at the fifth terminator nucleotide, after which the synthesized RNA was polyadenylated and retrotransposed to yield a daughter copy with a long poly A-tail and a short terminator. These samples confirm the T^+^ amplification of Can_b1 copies. We believe that Can_a1 and Can_a2 were also amplified via this pathway back when these subfamilies were active.

A similar analysis of Can_b2 was primed by their youngest copies. To date, we know nine hereditary diseases in dogs caused by recent deleterious Can integrations into genes. All these cases correspond to the Can_b2 subfamily. We tried to identify parental copies that induced the gene mutations by searching for the most similar sequences in the three dog breeds (German Shepherd, Great Dane, and Boxer). The number of the potential parental copies varied from 2 to 86. The nucleotide sequences of these Can_b2 copies are given in Figure S4, while Table 3 presents the A-tails of copies that induced the mutations as well as similar regions of their candidate parents. The candidates were selected based on the maximum similarity with these copies, presence in the genomes of the three dog breeds, presence of pol III terminators, or, if absent, longer poly(A) tails. We concluded that the two Can_b2 insertions genes (*ASIP* and *F8*-insertion 1) were generated via T^−^ retrotransposition since none of potential parental copies had functional terminators, although some of them had long poly(A)-tails disrupted by few T and/or G nucleotides (Table 3). Can_b2 integrations into the *ATP1B2*, *PTPLA*, *RAB3GAP1*, and *STK38L* genes were likely generated via T^+^ retrotransposition considering that the putative parental copies had functional terminators and relatively short poly A-tails (Table 3). Three more insertions (into the *F8*-insertion 2, *FAM161A,* and *SILV*) were likely generated via T^+^ retrotransposition, although the T^−^ pathway cannot be ruled out. The data obtained indicate that the retrotransposition of Can_b2 can follow both the T^+^ and T^−^ pathways depending on their structure.

**Figure 7:**
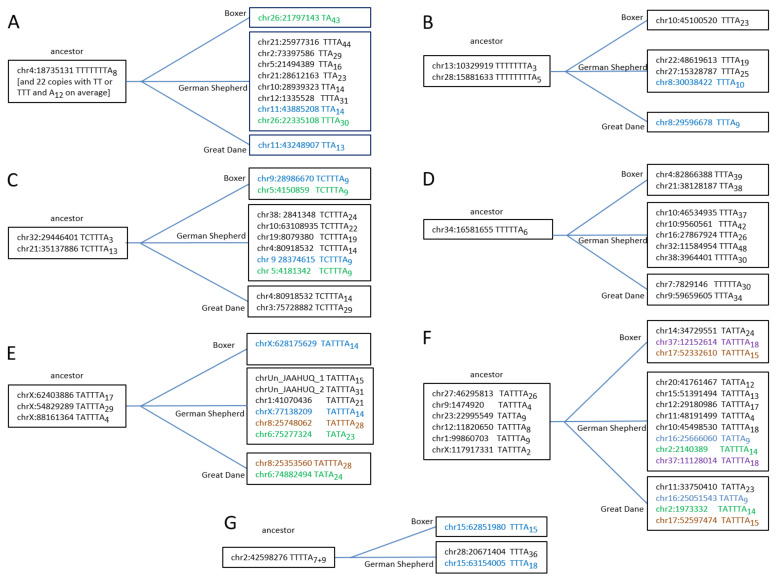
Examples of Can_b2 copies (**A**–**G**) illustrating their retrotransposition via T^+^ mechanism. The left coordinate, terminator structure, and poly(A) tail length are specified. The left (“ancestor”) boxes include copies present in the genomes of the German Shepherd, Great Dane, and Boxer; the right boxes include daughter copies present in one or two dog breeds (copies found in two breeds are given in the same color).

The analysis of young Can_b2 was extended by considering copies present in the German Shepherd but missing in orthologous Great Dane loci. A number of such copies are a good illustration of T^+^ retrotransposition. The nearest related copies were identified in the genomes of the three dog breeds. Figure S5 presents seven alignments of the young Can_b2 copies and their relatives (sequence similarity ≥ 99%), apparently including the parental copies that are present in all three genomes. The structures of the pol III terminators and poly(A) tail lengths as well as the presence of all closely related copies in the three dog breeds are shown in Figure 7. All putative parental copies had functional terminators and generally shorter poly(A) tails compared to the descendants, which indicates the T^+^ retrotransposition. The most illustrative examples, B, D, and G (Figure 7), allow only one interpretation: Tthe parental copies have long terminators (T_4–8_) and short A-tails (A_3–7_), while nearly all descendants (present in only one or two genomes) have rudimentary terminators (T_2–3_) and long A-tails (on average A_30_). This clearly confirms that these descendant Can_b2 copies emerged via T^+^ retrotransposition.

Most young Can_b2 copies in a sample selected by the presence in the German Shepherd and absence at orthologous loci in the Great Dane have no functional terminators but only their rudiments (TT or TTT). For several such copies, we searched their closest relatives (i.e., with highly similar nucleotide sequences) in the genomes of the German Shepherd, Great Dane, and Boxer. Multiple alignments for two such typical groups (tribes) are shown in Figures S6 and S7. The copies of the first tribe (45 members) have two (in most cases) or three Ts in the terminator position; no copies with a longer T-stretch that could be a pol III terminator were found. This clearly indicates the amplification of this tribe via the T^−^ rather than T^+^ mechanism.

The second and larger tribe (265 members) included only 100% identical sequences excluding variations in the TC-motif and A-tail (Figure S7). The majority of copies in this tribe had rudimentary terminators (TT or TTT) in their A-tails, which is common in Can_b2 copies. Most sequences in this tribe included two or more such T_2–3_A_6–14_ modules (Figure S7). In contrast to the first tribe, this one included five copies with a functional TTTT terminator preceding the first A-stretch, which makes possible their retrotransposition via the T^+^ pathway. However, functional terminators were more frequent upstream of the second A-stretch: ten T_4–9_ blocks and ten TCTTTs. These secondary terminators should allow the amplification via the T^+^ mechanism. Finally, 16 copies had terminators (T_4–10_) within their TSDs, which can also provide for T^+^ retrotransposition. (It should be noted that similar copies with potential T-stretch terminators within TSDs also occurred in the first tribe.)

**Figure 8:**
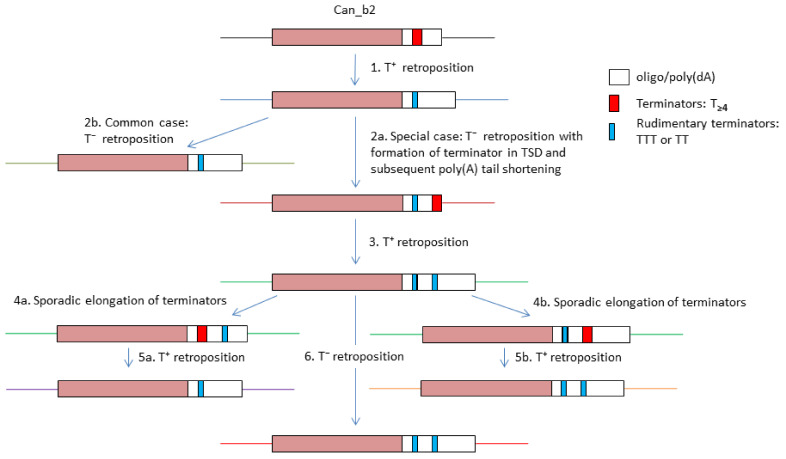
Schematic illustration of the multiplicity of 3′-terminal parts of Can_b2 (tribe 2 copies) and putative underlying mechanisms. SINEs with A-tails and terminators are given as boxes. The same flanking loci are indicated by same colors. See text for other explanations.

Figure 8 illustrates the emergence of copies with two functional or rudimentary terminators as well as a sporadic loss of one of them in Can_b2 copies within a tribe. The most common Can_b2 terminators composed of Ts shorten after transcription and retrotransposition with high probability, which makes them nonfunctional (event 1 in Figure 8). In particular cases, a new terminator can emerge at the end of a Can_b2 copy within a TSD (event 2a). After T^+^ retrotransposition, such a new terminator will likely shorten to give rise to a copy with two rudimentary terminators (event 3). SINE copies are passed through numerous host generations, and sometimes the terminators spontaneously elongate (Figure 5D and Vassetzky et al., 2021 [50]). This can restore the function of the first or second terminator in Can_b2 (events 4a and 4b). After transcription termination at the first or second terminator, the emerging SINE copies can have one or two terminators, respectively (events 5a and 5b). This diagram illustrates the diversity of 3’-terminal sequences in closely related tribe sequences as well as a possible alternation of T^+^ and T^−^ retrotransposition in these copies. It is not improbable that the presence of two terminators can somewhat compensate for the high incidence of their shortening after T^+^ retrotransposition. The capacity for T^+^ retrotransposition in certain copies can be advantageous for tribe expansion since the resulting copies have long poly(A) tails, which consequently favor their T^−^ retrotransposition.

### 3.5. Additional Considerations of A-Tails

Above, the TT and TTT signals preceding A-tails in Can_b2 were interpreted as rudimentary terminators. Here, we argue that these two or three Ts were not generated by spontaneous A to T mutations. Let us consider A-tails in young copies of Alu, a thoroughly studied human SINE [2,3] amplified via the T^−^ mechanism. About 30 human hereditary diseases induced by Alu insertion into genes are known (Table S1). In all cases, these young Alu copies had pure poly(A) tails with the mean length of 50 bp and up to 97 bp. This agrees with the reviews of disease-inducing Alu insertions [3,26]. For some of these copies, we searched for 100% identical (excluding the A-tails) Alu sequences in the reference human genome. The identified closely related Alu copies constituted tribes for each of these copies (Figure S8 exemplifies three such tribes named after the mutated genes: *FGFR2*, *FBP1*, and *PKLR*). All these tribes included copies with very long poly(A) tails; current models presume that such Alu sequences give rise to new copies such as those that induced hereditary diseases [3]. It should be noted that all copies in such tribes have long poly(A) tails, e.g., A_15–65_ in the *FGFR2* tribe (Figure S8A). The majority of these tails are composed of pure As; occasional tails have single-nucleotide substitutions but no di- or trinucleotides other than A. Similar findings concerning A-tails in young Alu have been reported previously [47]. Overall, this indicates that the A-tails of young Alu copies notably differ from those in young Can_b2 copies, which are shorter and often have TT or TTT. We believe that the spontaneous emergence of TT and TTT as a result of A to T substitutions is improbable but cannot be excluded. Poly(A) stretches are largely shorter in the tails of young Can_b2 than in young Alu copies (Figures S7 and S8). It is safe to assume that the T^−^ retrotransposition of Can_b2 is not as sensitive to the poly(A) tail length and the absence of T stretches relative to the T^−^ retrotransposition of Alu.

**Figure 9:**
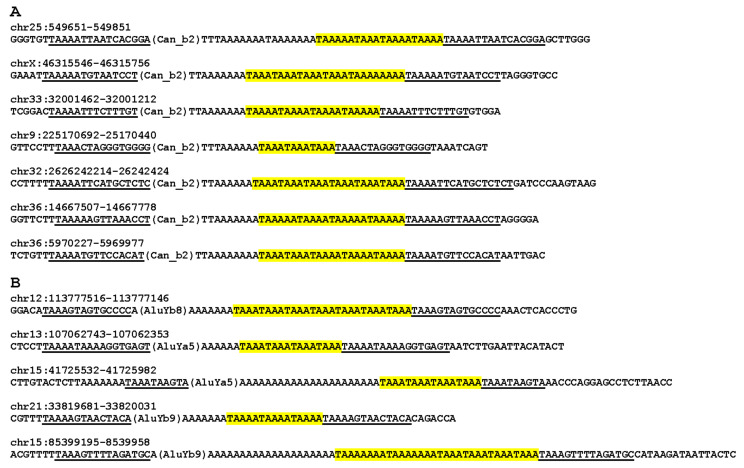
Examples of A-tails of SINEs with TA_3–5_ repeats (shown in yellow) and flanking sequences containing TSDs (underlined). (**A**) Can_b2; (**B**) Alu. Notice that TSDs start with TA_3–5_. The SINE coordinates in the German Shepherd and human genomes are given above sequences.

The analysis of A-tail structure in Can_b2 samples (in particular, tribes 1 and 2) demonstrate that 5–10% of them are composed of several TAAA, TAAAA, or TAAAAA tandem repeats (rarely, TA _>5_). In all such cases, TSD sequences start from TAAA, TAAAA, and TAAAAA, respectively (Figure 9A). These TSD regions could be responsible for the tandem repeats in the A-tails. To our knowledge, no such observations have been reported for SINEs with A-rich tails. In the analyzed three Alu tribes, we also found copies with tandem TA_3–4_ repeats, and their TSDs started from the corresponding 4- or 5-nt sequence (Figure 9B). It is not improbable that copies of any SINE families with A-tails can acquire such tandem repeats in their tail.

A priori, TA_3–5_ repeats can be generated (i) by target-primed reverse transcription at the time of new SINE copy formations and/or (ii) in the course of numerous cycles of genomic DNA replication in the host organism. Neither of these assumptions can be excluded. The proportion of Can_b2 copies with such repeats in tribe 2 was similar in the youngest copies found in a single dog breed as well as in older ones present in two or three breeds, 6.5 and 8.4%, respectively (Figure S7). These data support the first assumption; however, tandem repeats can be generated within a relatively short period after a new Can_b2 copy integration into the genome. Long SINE tails composed of pure poly(A) are unstable in a long series of host generations [47]; thus, TA_3–5_ repeats can stabilize such tails. Indeed, these repeats are quite stable in SINE tails: the analysis of Can_b2 copies in orthologous loci demonstrates that their number and repeat length are highly similar in the dog breeds as well as in the wolf (Figure S9A–C). The mechanism of such repeat generation remains unclear; however, it can be mediated by the slippage of the enzyme realizing the reverse transcription and/or DNA replication [48,90]. Notice that the slippage on a pure poly(A) template can be complicated by the 5′-terminal’s T residue. In this case, a template with individual Ts can be more suitable since it obviates the mismatching problem in slippage.

The amplification of short tandem repeats (microsatellites) by DNA slippage assumes that the distance between the flanking sequences increases with the number of repeat units. However, an analysis of A-tails of Can_b2 or Alu at orthologous loci of different dog breeds or human individuals, respectively, demonstrated that the increased number of TA_3–5_ repeats did not necessarily increase the total A-tail length (Figures S9D, E, and S10C, D). In other words, TA_3–5_ repeats replace poly(A) in such tails. The underlying mechanism is unknown, but if the repeats are amplified by DNA slippage, it should be accompanied by the deletion of a poly(A) region that is equal in size. Finally, the formation of TA_3–5_ repeats can initiate the emergence of T^+^ SINEs from T^−^ ones (and this is not an exceptional event considering that 12 independent T^+^ SINE families are known in placentals [1]). Furthermore, tandem TA_3–5_ repeats constitute AATAAA, a potential polyadenylation signal. A T^−^ SINE with such repeats should acquire a downstream pol III transcription terminator and then other polyadenylation signals (β and/or τ) to become a new T^+^ SINE.

### 3.6. Identification of Can Regions Significant for Polyadenylation of Its Pol III Transcripts

**Figure 10:**
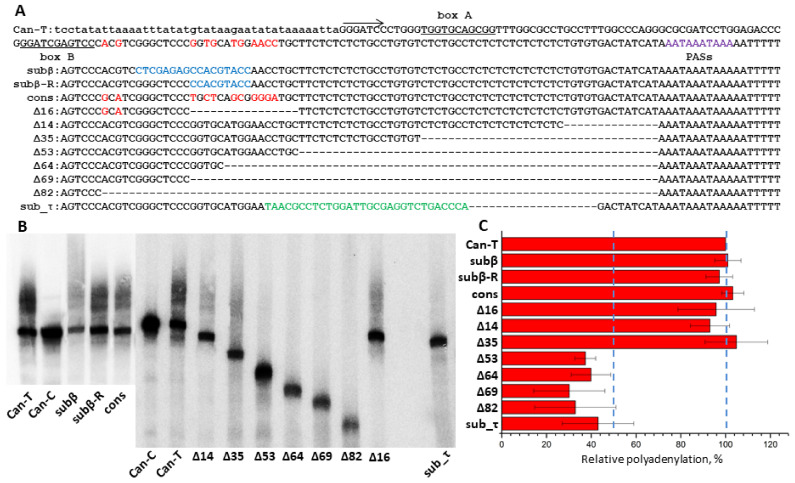
Identification of Can SINE regions required for polyadenylation of its pol III transcripts. (**A**) The Can sequence used in experiments (Can-T, above); the arrow indicates the transcription start; boxes A and B are underlined; polyadenylation signals (PASs) are shown in violet. Below is the alignment of the 3′-part of this sequence and derived constructs with modifications. The nucleotides different from the consensus in the region between the box B and TC-motif are given in red; blue marks the modifications in this region relative to the sequence above; modifications in the polypyrimidine region are shown in green. Deleted nucleotides, “-”. (**B**) Northern blot hybridization of RNA from HeLa cells transfected by Can constructs. The band and the smear above correspond to the primary and polyadenylated transcripts, respectively. Can-C is the construct with T-to-C substitutions in both polyadenylation signals, and its transcripts are not polyadenylated (For original image of Figure 10B, please refer to Supplementary Figure S11). (**C**) Polyadenylation efficiency of the modified constructs relative to Can-T. Error bars, SD, n = 3.

Our previous experiments on cell transfection with a plasmid containing a Can copy demonstrated that its RNA transcribed by pol III can be polyadenylated in an AAUAAA-dependent manner [45]. Here, we tried to identify regions other than AATAAA but that are also essential for the transcript polyadenylation in the same Can copy. By analogy to previously studied T**^+^** SINEs (B2, Dip and Ves), such regions could contain the β and τ signals downstream of the box B and upstream of (AATAAA)_n_, respectively [43,45]. The Can copy studied belongs to the Can_a1 subfamily, although its sequence naturally was not identical to the consensus sequence. A series of constructs were generated based on this copy with nucleotide substitutions and/or deletions; these constructs were used to transfect HeLa cells and the cellular RNA was analyzed by Northern hybridization with the Can-specific probe. We presumed that modifications within the putative β-signal that make it identical to the Can_a1 consensus sequence can increase the polyadenylation efficiency; however, it was not observed (Figure 10, *cons*). A long deletion (*Δ16*) or multiple nucleotide substitutions (*subβ* and *subβ_R*) in this region downstream of the box B scarcely decreased the polyadenylation rate (Figure 10). These data indicate the absence of β-signal in Can SINE. Testing a series of constructs with deletions that started immediately upstream of the PAS and proceeded to the Can head demonstrated a significant drop in polyadenylation efficiency only when the entire TC-motif was deleted (Figure 10, *Δ53*). Longer deletions (*Δ64, Δ69*, and *Δ82*) had no further effect on the polyadenylation efficiency. The deletion of 19 bp in the TC-motif together with the replacement of the rest 30 nt of the motif with a random sequence (*sub_τ* construct) also substantially decreased its polyadenylation efficiency (Figure 10). Thus, the TC-motif has the τ-signal function in Can. Previously, we demonstrated the same function for polypyrimidine motifs in Dip and Ves transcripts [45]; however, in contrast to these SINEs, Can lacks the β signal. This can be attributed to the short distance between box B and TC-motif (Figure 2 and Figure 10). From all appearances, Can is the shortest and simplest of known T^+^ SINEs.

## 4. Conclusions

In this study, we divided Can SINE copies from canid genomes into four distinguishable subfamilies (a1, a2, b1, and b2), although certain Can_b1 and Can_b2 copies are not easy to discern. The high rate of TSD loss as well as sequence divergence within the Can_a1 and Can_a2 subfamilies indicate their old age; Can_b1 and, particularly, Can_b2 are much younger. Currently, Can_b2 and to a lesser extent Can_b1 remain retrotranspositionally active, while Can_a1 and Can_a2, which were active millions of years ago, have largely lost their activity. The genomes of dog breed pairs differ by the presence or absence of Can copies at 5000–12,000 orthologous loci, which indicates an uncommonly high amplification activity of this SINE in dogs. Can subfamilies share a long variable polypyrimidine motif in their 3′-terminal region, which is followed by several overlapping AATAAA sites, a pol III transcription terminator or its rudiment, and the poly(A) or A-rich tail. The polypyrimidine motif, AATAAA, and functional terminator are required for the polyadenylation of pol III transcripts of Can. According to our model, such polyadenylated transcripts are convenient templates for the L1 reverse transcriptase and can give rise to new genomic copies of Can (T^+^ retrotransposition). Pol III transcripts of SINEs that lack the AATAAA and terminator signals in their 3′-terminal part (such as primate Alu) cannot be polyadenylated. In this case, only copies with a long poly(A) tail can give rise to new SINE copies (T^−^ retrotransposition). Thus, certain Can_b2 copies (with functional terminators) can amplify through T^+^ retrotransposition, while many other ones (with reduced terminators and long poly A-tails) can undergo T^−^ retrotransposition. It is not improbable that cycles of T^+^ and T^−^ retrotransposition can alternate to generate different tribes (Figure 8). Our data indicate that 5–10% copies of Can_b2 in dog and young Alu in human genomes have tandem TA_3–5_ repeats and all such copies had TSDs starting from TA_3–5_ as well. Presumably, TA_3–5_ in TSD (i.e., in the integration site) promotes the formation of these tandem repeats in the tail either in the reverse transcription or DNA replication.

## Figures and Tables

**Table 1:** Can subfamilies in certain dog-like and fox-like canids.

	Species		Total Number of Can Copies	Subfamilies: Proportion, %	Mean Similarity of Copies to Consensus	Proportion of Copies with TSD
Canini	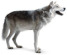	gray wolf	676,669	Can_a: 34%	64%	74%
		*Canis lupus*		a1: 20%	69%	74%
				a2: 14%	64%	70%
				Can_b: 66%	79%	90%
				b1: 48%	77%	87%
				b2: 18%	91%	96%
	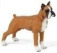	boxer	646,211	Can_a: 33%	64%	72%
		*Canis lupus familiaris*		a1: 18%	69%	74%
				a2: 15%	64%	71%
				Can_b: 67%	78%	86%
				b1: 50%	78%	85%
				b2: 17%	90%	93%
	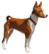	basenji	658,945	Can_a: 34%	63%	74%
		*Canis lupus familiaris*		a1: 19%	69%	73%
				a2: 15%	64%	72%
				Can_b: 66%	79%	86%
				b1: 49%	78%	84%
				b2: 17%	90%	93%
	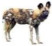	African wild dog	640,065	Can_a: 35%	64%	69%
		*Lycaon pictus*		a1: 20%	68%	73%
				a2: 15%	64%	67%
				Can_b: 65%	78%	89%
				b1: 49%	78%	84%
				b2: 16%	90%	95%
Vulpini	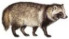	raccoon dog	668,821	Can_a: 32%	64%	69%
		*Nyctereutes procyonoides*		a1: 18%	69%	72%
				a2: 14%	64%	67%
				Can_b: 68%	78%	88%
				b1: 48%	78%	85%
				b2: 20%	90%	92%
	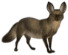	bat-eared fox	771,391	Can_a: 35%	63%	70%
		*Otocyon megalotis*		a1: 20%	55%	72%
				a2: 15%	63%	70%
				Can_b: 65%	78%	90%
				b1: 49%	77%	83%
				b2: 16%	90%	92%
	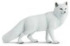	Arctic fox	667,350	Can_a: 33%	63%	76%
		*Vulpes lagopus*		a1: 19%	67%	79%
				a2: 14%	63%	71%
				Can_b: 67%	79%	88%
				b1: 47%	78%	82%
				b2: 20%	91%	93%

**Table 2:** Number of genome-specific Can copies and rates of their emergence in certain dog breeds, wolf, and other Caniformia.

Compared Genomes (Divergence Time)	Number of Genome-Specific Copies *		Mean Rate of Copies Emergence (Copies/My)	
	Genome 1	Genome 2	Genome 1	Genome 2
German Shepherd vs. Great Dane (0.001 Mya) **	12,074	5508	1.2 × 10^7^	5.5 × 10^6^
German Shepherd vs. Boxer (0.001 Mya) **	10.818	4775	1.1 × 10^7^	4.8 × 10^6^
Boxer vs. Great Dane (0.001 Mya) **	5494	6270	5.5 × 10^6^	6.3 × 10^6^
German Shepherd vs. Wolf (0.02 Mya)	12,917	11,763	6.5 × 10^5^	5.9 × 10^5^
Great Dane vs. wolf (0.02 Mya)	9370	14,109	4.7 × 10^5^	7.0 × 10^5^
Boxer vs. wolf (0.02 Mya)	7856	11,898	3.9 × 10^5^	5.9 × 10^5^
African wild dog vs. wolf (7.5 Mya)	19,176	28,691	2.6 × 10^3^	3.8 × 10^3^
Red fox vs. Arctic fox (3.6 Mya)	20,106	33,146	5.6 × 10^3^	9.2 × 10^3^
Giant panda vs. polar bear (17 Mya)	66,876	58,018	3.9 × 10^3^	3.4 × 10^3^

* Genome-specific copies are present in the genome of one species (breed) but missing in the other one. ** Although modern dog breeds were established within the recent 200 years, the time of breed divergence was set as 1000 years considering that the breed ancestors could diverg much earlier. High rates of Can emergence are colored blue (German Shepherd), green (Great Dane), yellow (Boxer), and gray (wolf).

**Table 3:** Can_b2 insertions that caused gene mutations and putative retrotransposition mechanisms.

Mutated Gene	Breed (Reference)	Tail of the Inserted Can Copy	Tail of Probable Parental Can Copy *	T^+^ Way	T^—^ Way
*F8* (insertion 1)	Rhodesian Ridgeback [81]	GTTA_25_TTA_4_	GTTA_10_TTTA_29_	unlikely	highly likely
			GTTA_9_TTTA_13_		
*F8* (insertion 2)	Havanese dog [82]	TATTTA_32_	TATTTA_8–32_	highly likely	likely
	(GenBank acc. number HE574814)		TATTTTA_7_		
*ASIP*	Doberman Pinscher and some other breeds [83]	TGA_14_GGA_36_	TGA_13_TGA_19_	unlikely	highly likely
			TGA_19_TGA_13_		
			TGA_15_GA_22_		
*ATP1B2*	Belgian Shepherd Dog [84]	TCTTTA_34_	TCTTTA_13_	highly likely	unlikely
*FAM161A*	Tibetan Spaniel and Tibetan Terrier [85]	TA_35–50_TA_11_	TA_5_TTTTA_9_TTA_9_	likely	likely
			TA_5_TTTTA_7_		
			TA_5_TTTTTTA_8_		
			TA_5_TTTTTA_6_		
			TTTTA_8_		
			TA_5_TTTTTA_7_		
			TA_5_TTTA_33_		
			TA_13_CA_7_		
*PTPLA*	Labrador [86]	TTA_12_TTTA_11_TTTA_16_	TTA_9_TTTTTTTA_3_	highly likely	unlikely
*RAB3GAP1*	Alaskan Husky [87]	TATTA_25_	TATTTA_11_	highly likely	unlikely
			TATTTTTA_7_		
			TATTTTTA_28_		
*SILV*	Shetland Sheepdog [88]	TTTA_100_	TTTTA_9_	likely	likely
			TTTA_28_		
*STK38L*	Norwegian elkhound [89]	TTTTA_25_	TTTTA_8_	highly likely	unlikely

* In probable paternal copies, terminators and long poly(A) stretches are marked with red and yellow, respectively.

## Data Availability

Not applicable.

## Supplementary Materials

The following supporting information can be downloaded at: https://www.mdpi.com/article/10.3390/biology11101403/s1, Figure S1: Distribution of pol III terminators or their rudiments among relatively young Can copies (SINEC1_Ame_Y) present in the giant panda (*Ailuropoda melanoleuca*) but missing in orthologous loci of the polar bear (*Ursus maritimus*). Figure S2: A. Multiple alignment of sequences from one of Can_b1 tribes in the three dog breeds. B. Analysis of the chr1_10744779 tribe demonstrating their T^+^ retrotransposition. Figure S3: Eight Can_b1Y copies (A–H) illustrating their T^+^ retrotransposition. Figure S4: Can_b2 insertions (A–I) into genes that induced their mutations in dogs. Figure S5: Seven examples (A–G) of groups of related Can_b2 copies for which their structure indicates their retrotransposition by the T^+^ mechanism. Figure S6: Members of the Can_b2 tribe 1 in the three dog breeds. Figure S7: Analysis of 3′-terminal regions of Can_b2 sequences of the tribe 2. Table S1: Examples of mutation-inducing Alu insertions into human genes [91,92,93,94,95,96,97,98,99,100,101,102,103,104,105,106,107,108,109,110,111,112,113,114,115,116,117,118]. Figure S8: A-tails of Alu copies closely related to those that induced mutations in the *FGFR2*, *FBP1*, and *PKLR* genes. Figure S9: Examples of Can_b2 copies with A-tails containing TA_3–5_ repeats at orthologous loci of different dog breeds and wolf. Figure S10: Examples of Alu copies with A-tails containing TA_3–5_ repeats from orthologous loci of different human genomes. Figure S11: Original image of full western blot.
