# Supplementary material for: Tail Wags Dog’s SINE: Retropositional Mechanisms of Can SINE Depend on Its A-Tail Structure
Source: Biology (Basel). 2022 Sep 26;11(10):1403. doi: 10.3390/biology11101403 (PMC9599045; doi:10.3390/biology11101403)
Supplement: Supplementary file 1 [file Figure S10.pdf › Figure S10.pdf]

A

```
1 : GTCCGGCCTGGGCGACAGAGCGAGACTCCGTCTCAAAAAAATAAAATAAAATAAAATAAAAGTAACTACACAGACCACTAGAGTTCTCATAGTAACATGAATGCCATCTG
2 : GTCCGGCCTGGGCGACAGAGCGAGACTCCGTCTCAAAAAAATAAAATAAAATAAAATAAAAGTAACTACACAGACCACTAGAGTTCTCATAGTAACATGAATGCCATCTG
3 : GTCCGGCCTGGGCGACAGAGCGAGACTCCGTCTCAAAAAAATAAAATAAAATAAAATAAAAGTAACTACACAGACCACTAGAGTTCTCATAGTAACATGAATGCCATCTG
4 : GTCCGGCCTGGGCGACAGAGCGAGACTCCGTCTCAAAAAAATAAAATAAAATAAAATAAAAGTAACTACACAGACCACTAGAGTTCTCATAGTAACATGAATGCCATCTG
5 : GTCCGGCCTGGGCGACAGAGCGAGACTCCGTCTCAAAAAAATAAAATAAAATAAAATAAAAGTAACTACACAGACCACTAGAGTTCTCATAGTAACATGAATGCCATCTG
6 : GTCCGGCCTGGGCGACAGAGCGAGACTCCGTCTCAAAAAAATAAAATAAAATAAAATAAAAGTAACTACACAGACCACTAGAGTTCTCATAGTAACATGAATGCCATCTG
7 : GTCCGGCCTGGGCGACAGAGCGAGACTCCGTCTCAAAAAAATAAAATAAAATAAAATAAAAGTAACTACACAGACCACTAGAGTTCTCATAGTAACATGAATGCCATCTG
8 : GTCCGGCCTGGGCGACAGAGCGAGACTCCGTCTCAAAAAAATAAAATAAAATAAAATAAAAGTAACTACACAGACCACTAGAGTTCTCATAGTAACATGAATGCCATCTG
9 : GTCCGGCCTGGGCGACAGAGCGAGACTCCGTCTCAAAAAAATAAAATAAAATAAAATAAAAGTAACTACACAGACCACTAGAGTTCTCATAGTAACATGAATGCCATCTG
10: GTCCGGCCTGGGCGACAGAGCGAGACTCCGTCTCAAAAAAATAAAATAAAATAAAATAAAAGTAACTACACAGACCACTAGAGTTCTCATAGTAACATGAATGCCATCTG
11: GTCCGGCCTGGGCGACAGAGCGAGACTCCGTCTCAAAAAAATAAAATAAAATAAAATAAAAGTAACTACACAGACCACTAGAGTTCTCATAGTAACATGAATGCCATCTG
12: GTCCGGCCTGGGCGACAGAGCGAGACTCCGTCTCAAAAAAATAAAATAAAATAAAATAAAAGTAACTACACAGACCACTAGAGTTCTCATAGTAACATGAATGCCATCTG
                                                         **************
```

B

```
1 : GTCCGACCTGGGCGACAGAGCGAGACTCCGTCTCAAAAAAATAAATAAATAAATAAATAAATAAATAAATAAATAAAGTAGTGCCCCAAACTCACCCTGGACTCATTACCCTAGCAATCG
2 : GTCCGACCTGGGCGACAGAGCGAGACTCCGTCTCAAAAAAA--------TAAATAAATAAATAAATAAATAAATAAAGTAGTGCCCCAAACTCACCCTGGACTCATTACCCTAGCAATCG
3 : GTCCGACCTGGGCGACAGAGCGAGACTCCGTCTCAAAAAAA----TAAATAAATAAATAAATAAATAAATAAATAAAGTAGTGCCCCAAACTCACCCTGGACTCATTACCCTAGCAATCG
4 : GTCCGACCTGGGCGACAGAGCGAGACTCCGTCTCAAAAAAA----TAAATAAATAAATAAATAAATAAATAAATAAAGTAGTGCCCCAAACTCACCCTGGACTCATTACCCTAGCAATCG
5 : GTCCGACCTGGGCGACAGAGCGAGACTCCGTCTCAAAAAAATAAATAAATAAATAAATAAATAAATAAATAAATAAAGTAGTGCCCCAAACTCACCCTGGACTCATTACCCTAGCAATCG
6 : GTCCGACCTGGGCGACAGAGCGAGACTCCGTCTCAAAAAAATAAATAAATAAATAAATAAATAAATAAATAAATAAAGTAGTGCCCCAAACTCACCCTGGACTCATTACCCTAGCAATCG
7 : GTCCGACCTGGGCGACAGAGCGAGACTCCGTCTCAAAAAAATAAATAAATAAATAAATAAATAAATAAATAAATAAAGTAGTGCCCCAAACTCACCCTGGACTCATTACCCTAGCAATCG
8 : GTCCGACCTGGGCGACAGAGCGAGACTCCGTCTCAAAAAAA--------TAAATAAATAAATAAATAAATAAATAAAGTAGTGCCCCAAACTCACCCTGGACTCATTACCCTAGCAATCG
9 : GTCCGACCTGGGCGACAGAGCGAGACTCCGTCTCAAAAAAATAAATAAATAAATAAATAAATAAATAAATAAATAAAGTAGTGCCCCAAACTCACCCTGGACTCATTACCCTAGCAATCG
10: GTCCGACCTGGGCGACAGAGCGAGACTCCGTCTCAAAAAAATAAATAAATAAATAAATAAATAAATAAATAAATAAAGTAGTGCCCCAAACTCACCCTGGACTCATTACCCTAGCAATCG
11: GTCCGACCTGGGCGACAGAGCGAGACTCCGTCTCAAAAAAA----TAAATAAATAAATAAATAAATAAATAAATAAAGTAGTGCCCCAAACTCACCCTGGACTCATTACCCTAGCAATCG
12: GTCCAACCTGGGCGACAGAGCGAGACTCCGTCTCAAAAAAA----TAAATAAATAAATAAATAAATAAATAAATAAAGTAGTGCCCCAAACTCACCCTGGACTCATTACCCTAGCAATCG
                                                                          ***************
```

C

```
1 : CTCCAGCCTGGGCGACAGAGCGAGACTCCGTCTCAAAAAAAAAATAAATAAATAAATAAAATAAAAGGTGAGTAATCTTGAATTACATACTCTAGTAATATTTATTTAATTTACTCAGAA
2 : CTCCAGCCTGGGCGACAGAGCGAGACTCCGTCTCAAAAAAAAAAAAAATAAATAAATAAAATAAAAGGTGAGTAATCTTGAATTACATACTCTAGTAATATTTATTTAATTTACTCAGAA
3 : CTCCAGCCTGGGCGACAGAGCGAGACTCCGTCTCAAAAAAAAAAAAAATAAATAAATAAAATAAAAGGTGAGTAATCTTGAATTACATACTCTAGTAATATTTATTTAATTTACTCAGAA
4 : CTCCAGCCTGGGCGACAGAGCGAGACTCCGTCTCAAAAAAAAAAAAAATAAATAAATAAAATAAAAGGTGAGTAATCTTGAATTACATACTCTAGTAATATTTATTTAATTTACTCAGAA
5 : CTCCAGCCTGGGCGACAGAGCGAGACTCCGTCTCAAAAAAAAAAAAAATAAATAAATAAAATAAAAGGTGAGTAATCTTGAATTACATACTCTAGTAATATTTATTTAATTTACTCAGAA
6 : CTCCAGCCTGGGCGACAGAGCGAGACTCCGTCTC-AAAAAAAAAAAAATAAATAAATAAAATAAAAGGTGAGTAATCTTGAATTACATACTCTAGTAATATTTATTTAATTTACTCAGAA
7 : CTCCAGCCTGGGCGACAGAGCGAGACTCCGTCTCAAAAAAAAAAAAAATAAATAAATAAAATAAAAGGTGAGTAATCTTGAATTACATACTCTAGTAATATTTATTTAATTTACTCAGAA
8 : CTCCAGCCTGGGCGACAGAGCGAGACTCCGTCTCAAAAAAAAAAAAAATAAATAAATAAAATAAAAGGTGAGTAATCTTGAATTACATACTCTAGTAATATTTATTTAATTTACTCAGAA
9 : CTCCAGCCTGGGCGACAGAGCGAGACTCCGTCTC-AAAAAAAAAAAAATAAATAAATAAAATAAAAGGTGAGTAATCTTGAATTACATACTCTAGTAATATTTATTTAATTTACTCAGAA
10: CTCCAGCCTGGGCGACAGAGCGAGACTCCGTCTCAAAAAAAAAAAAAATAAATAAATAAAATAAAAGGTGAGTAATCTTGAATTACATACTCTAGTAATATTTATTTAATTTACTCAGAA
11: CTCCAGCCTGGGCGACAGAGCGAGACTCCGTCTCAAAAAATAAATAAATAAATAAATAAAATAAAAGGTGAGTAATCTTGAATTACATACTCTAGTAATATTTATTTAATTTACTCAGAA
12: CTCCAGCCTGGGCGACAGAGCGAGACTCCGTCTCAAAAAAAAAAAAAATAAATAAATAAAATAAAAGGTGAGTAATCTTGAATTACATACTCTAGTAATATTTATTTAATTTACTCAGAA
                                                         *****************
```

D

```
1 : GTCCGCAGTCCGGCCTGGGCGACAGAGCGAGACTCCGTCTCAAAAAAAAAAAAAAAAAAAAAA-----TAAATAAATAAATAAATAAATAAATAAATAAAGTTTTAGATGCCATAAGATAATTACTCATA
2 : GTCCGCAGTCCGGCCTGGGTGACAGAGCGAGACTCCGTCTCAAAAAAAAAA-----TAAAAAAATAAAAAAAAAAAATAAATAAATAAATAAATAAATAAAGTTTTAGATGCCATAAGATAATTACTCATA
3 : GTCCGCAGTCCGGCCTGGGCGACAGAGCGAGACTCCGTCTCAAAAAAAAAAAAAAAAAAA----TAAAAAAATAAAAAAATAAAAAAATAAATAAATAAAGTTTTAGATGCCATAAGATAATTACTCATA
4 : GTCCGCAGTCCGGCCTGGGTGACAGAGCGAGACTCCGTCTCAAAAAAAAAAAAAAAAAAAAAAAAAAAAAAAAAAAAAAAAAAAA---TAAATAAATAAAGTTTTAGATGCCATAAGATAATTACTCATA
5 : GTCCGCAGTCCGGCCTGGGCGACAGAGCGAGACTCCGTCTCAAAAAAAAAAAAAAAAAAAAA--TAAAAAAATAAAAAAATAAAAAAATAAATAAATAAAGTTTTAGATGCCATAAGATAATTACTCATA
6 : GTCCGCAGTCCGGCCTGGGCGACAGAGCGAGACTCCGTCTCAAAAAAAAAAAAAAAAAAAAAAAAAA-----TAAAAAAATAAA-AAATAAATAAATAAAGTTTTAGATGCCATAAGATAATTACTCATA
7 : GTCCGCAGTCCGGCCTGGGCGACAGAGCGAGACTCCGTCTCAAAAAAAAAAAAAAAAAA-----TAAATAAATAAATAAATAAATAAATAAATAAATAAAGTTTTAGATGCCATAAGATAATTACTCATA
8 : GTCCGCAGTCCGGCCTGGGTGACAGAGCGAGACTCCGTCTCAAAAAAAAAAAA---------------TAAATAAATAAATAAATAAATAAATAAATAAAGTTTTAGATGCCATAAGATAATTACTCATA
9 : GTCCGCAGTCCGGCCTGGGCGACAGAGCGAGACTCCGTCTCAAAAAAAAAAAAAAAAAAAAAAAAAAATAAATAAATAAATAAATAAATAAATAAATAAAGTTTTAGATGCCATAAGATAATTACTCATA
10: GTCCGCAGTCCGGCCTGGGCGACAGAGCGAGACTCCGTCTCAAAAAAAAAAAAAA---------TAAATAAATAAATAAATAAATAAATAAATAAATAAAGTTTTAGATGCCATAAGATAATTACTCATA
11: GTCCGCAGTCCGGCCTGGGCGACAGAGCGAGACTCCGTCTCAAAAAAAAAAAAAAAAAAAA---TAAAAAAATAAAAAAATAAATAAATAAATAAATAAAGTTTTAGATGCCATAAGATAATTACTCATA
12: GTCCGCAGTCCGGCCTGGGCGACAGAGCGAGACTCCGTCTCAAAAAAAAAAAAAAAA---TAAAAATAAAAATAAAAAAATAAAAAAATAAATAAATAAAGTTTTAGATGCCATAAGATAATTACTCATA
                                                                                                    ***************
```

Figure S10. Examples of Alu copies with A-tails containing $TA_{3\text{-}5}$ repeats from orthologous loci of different human genomes. The 3’-terminal Alu sequences (highlighted in black) are given together with A-rich tails and flanking sequences (highlighted gray). $TA_{3\text{-}5}$ repeats are marked yellow. Asterisks indicate TSDs. **A** exemplifies conserved TA_3 repeats. **B** exemplifies A-tails polymorphic by both the length and number of TA_3 repeats (apparently, resulted from the canonical template slippage in DNA replication). **C** exemplifies A-tails of the same length but with a different number of repeat units. **D** exemplifies a combination of the above **B** and **C** A-tails. The A nucleotides at positions were T could be expected are boldfaced (these can result from errors in the canonical template slippage mechanism). Alu coordinates in the reference genome (GRCh38): **A**, chr21:33819681-33820031; **B**, chr12:113777516-113777146; **C**, chr13:107062743-107062353; and **D**, chr15:85399195-8539958. Other human genomes: 1, HG01243 Puerto Rican; 2, NA24385 Ashkenazim (Caucasian); 3, HG00514 Han Chinese Southern; 4, NA19240 Yoruban (African); 5, HG00733 Puerto Rican; 6, SAMD00243993 Japanese; 7, KOREF_S1 Korean; 8, NA12878 Utah Mormon (Caucasian); 9, AK1 Korean; 10, KOREF Korean; 11, hg38; 12, 2T2.
