# Supplementary material for: Tail Wags Dog’s SINE: Retropositional Mechanisms of Can SINE Depend on Its A-Tail Structure
Source: Biology (Basel). 2022 Sep 26;11(10):1403. doi: 10.3390/biology11101403 (PMC9599045; doi:10.3390/biology11101403)
Supplement: Supplementary file 1 [file Figure S9.pdf › Figure S9.pdf]

A

```
G. Shepherd : TCTGTGACTATCATAAATAAATAAAAATTT-AAAAAATTAAAAAAATAAATAAATAAATAAATAAAAAAAATAAAAATGTAATCCTTAGGGTGCCTGGGTGGCTCAGTGGGTTAGGTGTCCAACTCCTGA
Boxer       : TCTGTGACTATCATAAATAAATAAAAATTT-AAAAAATTAAAAAAATAAATAAATAAATAAATAAAAAAAATAAAAATGTAATCCTTAGGGTGCCTGGGTGGCTCAGTGGGTTAGGTGTCCAACTCCTGA
Labrador    : TCTGTGACTATCATAAATAAATAAAAATTT-AAAAAATTAAAAAAATAAATAAATAAATAAATAAAAAAAATAAAAATGTAATCCTTAGGGTGCCTGGGTGGCTCAGTGGGTTAGGTGTCCAACTCCTGA
G. Dane     : TCTGTGACTATCATAAATAAATAAAAATTT-AAAAAATTAAAAAAATAAATAAATAAATAAATAAAAAAAATAAAAATGTAATCCTTAGGGTGCCTGGGTGGCTCAGTGGGTTAGGTGTCCAACTCCTGA
Basenji     : TCTGTGACTATCATAAATAAATAAAAATTTAAAAAAATTAAAAAAATAAATAAATAAATAAATAAAAAAAATAAAAATGTAATCCTTAGGGTGCCTGGGTGGCTCAGTGGGTTAGGTGTCCAACTCCTGA
Dingo       : TCTGTGACTATCATAAATAAATAAAAATTT-AAAAAATTAAAAAAATAAATAAATAAATAAATAAAAAAAATAAAAATGTAATCCTTAGGGTGCCTGGGTGGCTCAGTGGGTTAGGTGTCCAACTCCTGA
wolf        : TCTGTGACTATCATAAATAAATAAAAATTT-AAAAAATTAAAAAAATAAATAAATAAATAAATAAAAAAAATAAAAATGTAATCCTTAGGGTGCCTGGGTGGCTCAGTGGGTTAGGTGTCCAACTCCTGA
                                                                                     ***************
```

B

```
G. Shepherd : TCTGTGACTATCATAAATAAATAAATATTAAAAAAAATAAATAAATAAAATAAAATAAAATAAAATGTTCCACATAATTGACCCCTCTCTCTTAATTTAAACCCAATCTCCAAACTAGGGTACATAGGA
Basenji     : TCTGTGACTATCATAAATAAATAAATATTAAAAAAAATAAATAAATAAAATAAAATAAAATAAAATGTTCCACATAATTGACCCCTCTCTCTTAATTTAAACCCAATCTCCAAACTAGGGTACATAGGA
Dingo       : TCTGTGACTATCATAAATAAATAAATATTAAAAAAAATAAATAAATAAAATAAAATAAAATAAAATGTTCCACATAATTGACCCCTCTCTCTTAATTTAAACCCAATCTCCAAACTAGGGTACATAGGA
wolf        : TCTGTGACTATCATAAATAAATAAATATTAAAAAAAATAAATAAATAAAATAAAATAAAATAAAATGTTCCACATAATTGACCCCTCTCTCTTAATTTAAACCCAATCTCCAAACTAGGGTACATAGGA
                                                                          ***************
```

C

```
G. Shepherd : TCTGTGTGACTATCATAAATAAATAAAAATTTAAAAAAATAAAATAAAATAAAACTTTGATTCTTGTAAACTCCAATTAGTAATTGAAAGAGACCTTACAGCATCTCTGTACCAGAGACT
G. Dane     : TCTGTGTGACTATCATAAATAAATAAAAATTTAAAAAAATAAAATAAAATAAAACTTTGATTCTTGTAAACTCCAATTAGTAATTGAAAGAGACCTTACAGCATCTCTGTACCAGAGACT
Boxer       : TCTGTGTGACTATCATAAATAAATAAAAATTTAAAAAAATAAAATAAAATAAAACTTTGATTCTTGTAAACTCCAATTAGTAATTGAAAGAGACCTTACAGCATCTCTGTACCAGAGACT
Chihuahua   : TCTGTGTGACTATCATAAATAAATAAAAATTTAAAAAAATAAAATAAAATAAAACTTTGATTCTTGTAAACTCCAATTAGTAATTGAAAGAGACCTTACAGCATCTCTGTACCAGAGACT
Dingo       : TCTGTGTGACTATCATAAATAAATAAAAATTTAAAAAAATAAAATAAAATAAAACTTTGATTCTTGTAAACTCCAATTAGTAATTGAAAGAGACCTTACAGCATCTCTGTACCAGAGACT
                                                               ***************
```

D

```
G. Shepherd: TCTGTGACTATCATAAATAAATAAAAATT--AAAAAAAAAAAATTTAAAAAATAAATAAATAAATAAACTAGGGTGGGGTAAATCAGTGGAAGGTGGGGGGAGGTAGAGGGAAGCAGCAA
Boxer      : TCTGTGACTATCATAAATAAATAAAAATTT-AAAAAAAAAAAATTTAAAAAATAAATAAATAAATAAACTAGGGTGGGGTAAATCAGTGGAAGGTGGGGGGAGGTAGAGGGAAGCAGCAA
G. Dane    : TCTGTGACTATCATAAATAAATAAAAATT--AAAAAAAAAAAATTTAAAAAATAAATAAATAAATAAACTAGGGTGGGGTAAATCAGTGGAAGGTGGGGGGAGGTAGAGGGAAGCAGCAA
Dingo      : TCTGTGACTATCATAAATAAATAAAAATTTAAAAAAAAAAAAATTTAAAAAATAAATAAATAAATAAACTAGGGTGGGGTAAATCAGTGGAAGGTGGGGGGAGGTAGAGGGAAGCAGCAA
Basenji    : TCTGTGACTATCATAAATAAATAAAAATTTAAAAAAAAAAAAATTTAAAAAAAAAATAAATAAATAAACTAGGGTGGGGTAAATCAGTGGAAGGTGGGGGGAGGTAGAGGGAAGCAGCAA
wolf       : TCTGTGACTATCATAAATAAATAAAAATTTAAAAAAAAAAAAATTTAAAAAAAAAATAAATAAATAAACTAGGGTGGGGTAAATCAGTGGAAGGTGGGGGGAGGTAGAGGGAAGCAGCAA
                                                                             ***************
```

E

```
G. Shepherd: CTGTGACTATCATAAAATAAAAAATAAAAATTAAAAAAAAATTTAAAAAAAAAATAAAAATAAAAATAAAAAGGCACATTAGAAAGTGTTAGATTTTTAAGTGCTAGCACCTAACTGAGA
Boxer      : CTGTGACTATCATAAAATAAAAAATAAAAATTAAAAAAAAATTTAAAAAAAAAATAAAAATAAAAATAAAAAGGCACATTAGAAAGTGTTAGATTTTTAAGTGCTAGCACCTAACTGAGA
G. Dane    : CTGTGACTATCATAAAATAAAAAATAAAAATTAAAAAAAAATTTAAAAAAAAAATAAAAATAAAAATAAAAAGGCACATTAGAAAGTGTTAGATTTTTAAGTGCTAGCACCTAACTGAGA
Labrador   : CTGTGACTATCATAAAATAAAAAATAAAAATTAAAAAAAAATTTAAAAAAAAAATAAAAATAAAAATAAAAAGGCACATTAGAAAGTGTTAGATTTTTAAGTGCTAGCACCTAACTGAGA
Dingo      : CTGTGACTATCATAAAATAAAAAATAAAAATTAAAAAAAAATTTAAAAGAAAAATAAAAATAAAAATAAAAAGGCACATTAGAAAGTGTTAGATTTTTAAGTGCTAGCACCTAACTGAGA
Basenji    : CTGTGACTATCATAAAATAAAAAATAAAAATTAAAAAAAAATTTAAAATAAAAATAAAAATAAAAATAAAAAGGCACATTAGAAAGTGTTAGATTTTTAAGTGCTAGCACCTAACTGAGA
wolf       : CTGTGACTATCATAAAATAAAAAATAAAAATTAAAAAAAAATTTAAAATAAAAATAAAAATAAAAATAAAAAGGCACATTAGAAAGTGTTAGATTTTTAAGTGCTAGCACCTAACTGAGA
                                                                               ***************
```

Figure S9. Examples of Can_b2 copies with A-tails containing $TA_{3\text{-}5}$ repeats at orthologous loci of different dog breeds and wolf. The 3'-terminal Can sequences (highlighted in black) are given together with A-rich tails and flanking sequences (highlighted gray). $TA_{3\text{-}5}$ repeats are marked yellow. Asterisks indicate TSDs. **A**, **B**, and **C** exemplify conserved $TA_{3\text{-}5}$ repeats, while **D** and **E** illustrate changed number of the repeat units without changing the A-tail length. Can coordinates in the German Shepherd genome: **A**, chrX:46315547-46315870; **B**, chr36:5970227-5969977; **C**, chr8:60196349-60196099; **D**, chr9:225170692-25170440; and **E**, chr4:57990307-57990607.
