# Supplementary material for: Tail Wags Dog’s SINE: Retropositional Mechanisms of Can SINE Depend on Its A-Tail Structure
Source: Biology (Basel). 2022 Sep 26;11(10):1403. doi: 10.3390/biology11101403 (PMC9599045; doi:10.3390/biology11101403)
Supplement: Supplementary file 1 [file biology-11-01403-s001.zip › Figure S3.pdf]

A

```
sdb_chr4_86942253  : TCAATGCAACATTTGCCCAGAATGGGGTCAACCAGAAATATTAGAGGGATCCCTGGGTGGCGCAGCGGTTTGGCGCCTGCCTTTGGCCCGGGGCGCGATCCTGGAGGCCTGGGATCGAGTCCCACGTCGG
s_chrX_93595261    : TGTTAGTTTCCCCTGAGGACGAGCATTGTTAAGAACTGAGTTCTCGGGATCCCTGGGTGGCGCAGCGGTTTGGCGCCTGCCTTTGGCCCGGGGCGCGATCCTGGAGGCCTGGGATCGAGTCCCACGTCGG

sdb_chr4_86942253  : GCTCCCTGCATGGAGCCTGCTTCTCCCTCTGCCTGTGTCTCTGCCTCTCTGTCTCTCTCTGTGTAACTATCATGAATAAATAAATAAAATCTTTAAAATATATATATAAAAAAAAAAAACAGAAATATTA
s_chrX_93595261    : GCTCCCTGCATGGAGCCTGCTTCTCCCTCTGCCTGTGTCTCTGCCTCTCTGTCTCTCTCTGTGTAACTATCATGAATAAATAAATAAAATCTTTAAAAAAAAAAAAAAAAAAAAAAAAAAAAAAAAAAAAA

sdb_chr4_86942253  : GATGAAAAGCGCTAATGAGCACTTCAGAGTATGACCCAAATATATCTTATAGATTCTAAAGGTCCA
s_chrX_93595261    : AAAAAAAAAAAAAAAAAAAAAAAAAAAAAAAAAAGAACTGAGTTCTCTGGGTATTTCAAGATGGTT
```

B

```
sdb_chr10_1333648  : ACCGTCTGTCTCCAAACAGTGTTATTACAATCTTATTCACTATTGGGACGCCTGGGTGGCTCAGTGGTTGAGCATCTGCCTTTGGCCCAGGGCATGATCCCGGAGTCCTGGGATCGAGTCCCGCATCGGG
s_chr3_67054234    : GTAGTTAGTCATCATCACAAAGAAGTCATTAATTATGTAAGGGAGGGACGCCTGGGTGGCTCAGTGGTTGAGCATCTGCCTTTGGCCCAGGGCATGATCCCGGAGTCCTGGGATCGAGTCCCGCATCGGG

sdb_chr10_1333648  : CTCCCTGCATGGAGCCTGCTTCTCCCTCTGCCTGTGTCTCTGCCTCTCTCTCTCTCTGTATCTCTCGTGAATAAATAAATAAAATCTTTTTTAAAAATCTTATTCACTATATTCCCTATGTTGTACTTTT
s_chr3_67054234    : CTCCCTGCATGGAGCCTGCTTCTCCCTCTGCCTGTGTCTCTGCCTCTCTCTCTCTCTGTATCTCTCGTGAATAAATAAATAAAATCTTTAAAAAAAAAAAAAAAAAAAAAAAAAAAAAAATAATTATGTAAGG

sdb_chr10_1333648  : CATTTCTGTGACTTCTTTATTTTATAACTGAAGTTAATACTTCCTAATCCCCTTTATCC
s_chr3_67054234    : GAGGTTTAGGATCATTATTATGCAGGCAGAGTATTCGGAACCTATCTTTTGCCATAAAA
```

C

```
sdb_chr3_87748296  : TGATAGAGAAAATGTTAATAATGCAGAATAAAATTAAAAGTGTAGGGGATCCCTGGGTGGCTCAGTGGTTCAGCGCCTGCCTTTGGCCCAGGGCATGATCCTGGAGTCCCGGGATCGAGTCCCACATCGG
sb_chr19_45534426  : AAAACAAACACAATGAACAACTTGACTTTTAAAAAAGAAGCCTAGGGGATCCCTGGGTGGCTCAGTGGTTCAGCGCCTGCCTTTGGCCCAGGGCATGATCCTGGAGTCCCGGGATCGAGTCCCACATCGG

sdb_chr3_87748296  : GCTCCCTGCATAGAGCCTGCTTCTCCCTCCTCCTGTGTCTCTGCCTCTCTCTCTCTCTATGTCTATCATGAATAAATAAATAAAATCTTTTTAAAAAAAGTGTGTCATGTCTATAGGATTACATTGTGAG
sb_chr19_45534426  : GCTCCCTGCATAGAGCCTGCTTCTCCCTCCTCCTGTGTCTCTGCCTCTCTCTCTCTCTATGTCTATCATGAATAAATAAATAAAATCTTTAAAAAAAAAAAAAAAACGAAGCCTAGTGTAGCGTCTGCTA

sdb_chr3_87748296: TAGAACAAACATTTGAGGAAATATTCTTCTAGTTCAGTGAAGATGTAGTTCATGT
sb_chr19_45534426: TAAGCACAGTGAGTACAATTTAAATACGGCTGTCTGAATGAAAGATGAAATGAGA
```

D

```
sdb_chr23_23784402  : AAATGCAAAACTGTAAACAGTTCTATAAAACTATGGAAGACTGGGGATCCCTGGGTGGCTCAGTGGTCTAGCGCCTGCCTTTGGCCCAGGGCGTGATCCTGGAGTCCCGGGATCGAGTCCCACATCAGGC
sdb_chr1_26893311   : AATTTAGGATGAGAACAAGGGACTCTTTGAAAGATACTTTTAGGGGATCCCTGGGTGGCTCAGTGGTCTAGCGCCTGCCTTTGGCCCAGGGCGTGATCCTGGAGTCCCGGGATCGAGTCCCACATCAGGC
s_chr30_3143895     : GTGTGCTACAGCAAAGGTTAATTTTTTAAAATATCATCTCCAGGGGATCCCTGGGTGGCTCAGTGGTCTAGCGCCTGCCTTTGGCCCAGGGCGTGATCCTGGAGTCCCGGGATCGAGTCCCACATCAGGC

sdb_chr23_23784402  : TCCCTGCATGGAACCTGCTTCTCCTTCTGCCTATGCCTCTGCTTCTCTCTCTTTCTCTGTGTCTCTCATGAATAAATAAATAAAATATTTTTAAAAAAATTATGGAAGACTTAACTGAAATTTCTAAGAA
sdb_chr1_26893311   : TCCCTGCATGGAACCTGCTTCTCCTTCTGCCTATGCCTCTGCTTCTCTCTCTTTCTCTGTGTCTCTCATGAATAAATAAATAAAATATTTAAAAAAAAAAAGAAAGATACTTTTATCTAGAGAAGGATTT
```

s_chr30_3143895 : TCCCTGCATGGAACCTGCTTCTCCTTCTGCCTATGCCTCTGCTTCTCTCTCTTTCT--GTGTCTCTCATGAATAAATAAATAAAATATTTAAAAAAAAAAAAAAAAAAAAAAAATAAAATATCATCTCCAA

sdb_chr23_23784402 : GCTAATAAAACATGCAATTTGTTTAATACCTTTGACTAGAAATTTGATAGTGATTGACTG
sdb_chr1_26893311 : TCTTTGTTTCAGAAAGAAACATGCGGCTACTGAGACAGTGTAGTTCCACATCTGGAACTG
s_chr30_3143895 : TCAGGAGACTAAGATAGACATGCAAGTTATTGATGAAGTAAGGTATTCCTCATTTAATAG

E

sdb_chr1_80441356: GCAAAGTCAATAGAAGTTCACGTAGAAGCTGCTGGAACCCGGGGATCCCTGGATGGCTCAGCGGTTTGGCGCCTGCCTTTGGCCCAGGGTGCGATCCTGGAGTCCCGGGATCGAGTCCCACGTCAGGCTC
4_chr16_51570943 : TTTATGCACCTAATAACAGAGCTTCAAAATACAGGAAGCAGGGGATCCCTGGATGGCTCAGCGGTTTGGCGCCTGCCTTTGGCCCAGGGTGCGATCCTGGAGTCCCGGGATCGAGTCCCACGTCAGGCTC

sdb_chr1_80441356: CCGGCATGGAGCCTGCTTCTCCCTCTGCCTGTGTCTCTGCCTCTCTCTCTCTCTGTCTATCATGAATGAATAAATAAATCTTTAAAAAAAAAAAAAAAAGAAGAAGCAGCTGGAACCCATGTCATGGG
s_chr16_51570943 : CCGGCATGGAGCCTGCTTCTCCCTCTGCCTGTGTCTCTGCCTCTCTCTCTCTCTGTCTATCATGAATGAATAAATAAATCTTTAAAAAAAAAAAAAAAAAAAAAAAAAAAAAAAAAAAAAAAAAAAAACAAAA

sdb_chr1_80441356: TATTTCCTTACAGCTACTTCAGCTAGTTTCAGAAAACTAGGCTGGCTTCAGCCTT
s_chr16_51570943 : TACAGGAAGCAAAATCTGCTAGAACTGTGAAAAGACAAACCCACAGTTGTAGCTG

F

sdb_chr25_40928195 : GAAAAGTAAACAAATGACTACATAGATTTGAAAGTAGCTTAAAGGGACCCCTGAGTGGCTCAGCGGTTGAGCATCTGCCTTTGGCTCAGGTCATGATCCTGGAGTCCCAGGTTCAAGTCCCACATCGGGC
s_chr6_67264924 : TTAATCAGTGCACTTAAAAACTGATATTCAAAATGAGTACTGAGGGACCCCTGAGTGGCTCAGCGGTTGAGCATCTGCCTTTGGCTCAGGTCATGATCCTGGAGTCCCAGGTTCAAGTCCCACATCGGGC

sdb_chr25_40928195 : TCCCTGCATGGAGCCTGCTTCTCCCTCTGCCTCTGACTCTGCCTCTCTCTCTGTGTCTCTCGTGAATAAATAAATAAAATCTTTTTTAAAAAAGAAAGTAACTTAAAGTTTGCATGAATTTTCCCTACAA
s_chr6_67264924 : TCCCTGCATGGAGCCTGCTTCTCCCTCTGCCTCTGACTCTGCCTCTCTCTCTGTGTCTCTCGTGAATAAATAAATAAAATCTTTAAAAAAAAAAAAAAAAAAAAAAAAAAAAAAAAAAAAAAAAAAAAAAAAAAAAAAAAAAA

sdb_chr25_40928195 : TTATCAACACACTAAGAAAACATTAGGGTCATTCATTTCACATTTTTATATATT
s_chr6_67264924 : AACAAAATGAGTACTGATATAGTTGGATCAATACATATTATACTTGTTACTATT

G

sdb_chr6_48693053 : GTGTCTCTCAAGAATAAATAAATTAAATATTTAATGAAAAAAATCGGGCAGCCCCGGTGGCGCAGCGGTTTAGTGCCGCCTACAGCCCAGGGCGTGATCCTGGAGACCCTGGATCGAGTCCCACGTCAGG
sd_chr4_15005893 : GTGCACTTCCTGGAATCTGGATGCCCAATAATAATCTGTTGAATAGGGCAGCCCCGGTGGCGCAGCGGTTTAGTGCCGCCTACAGCCCAGGGCGTGATCCTGGAGACCCTGGATCGAGTCCCACGTCAGG

sdb_chr6_48693053 : CTCCCTGCGTGGAGCCTGCTTCTCCCTCTGCCTGTGTCTCTGCCTCTCTCTCTCTCTCTCTCTGCATCTCTATGAATAAATAAATAAAATCTTTAAAAAAAAGAAAAAAAATCTCACAAAGTATACAGCC
sd_chr4_15005893 : CTCCCTGCGTGGAGCCTGCTTCTCCCTCTGCCTGTGTCTCTGCCTCTCTCTCTCTCTCTCTCTGCATCTCTATGAATAAATAAATAAAATCTTTAAAAAAAAAAAAAAAAAAAAAAAAAAAAAAAAAAATAAT

sdb_chr6_48693053 : AAAAGTATCTTACCCTCTTATAATAATTTTCTGTTTACATTATTTAAAACATCTTCCGGAAGCA
sd_chr4_15005893 : CTGTTGAATAAAATGAGTAAATGAATGAACAAATTCCTGCCTATGACTGCTTCAGGTTGGTATTA

H

```
sdb_chr8_3186075   : GAGCAGAGCATTAGGTAGACCCAAGATCAAAAGGGTGCTGCACAGGGGCAGCCCAGGTGGCTCAGCGGTTTAGCGCCACCTTCAGCCCAGGGCCTGATCCTGGAGACCTGGGATCGAGTCCCGCATCGGG
sd_chr2_64843826   : TGAGAGACAGGGGAGCAAAAAGTCGAGGAGAAAGAGGAGGGAGAGGGGCAGCCCAGGTGGCTCAGCGGTTTAGCGCCACCTTCAGCCCAGGGCCTGATCCTGGAGACCTGGGATCGAGTCCCGCATCGGG

sdb_chr8_3186075   : CTCCCTGCATGGAGCCTGCTTCTCCCTCTGCCTGTGTCTCTGCCTCTCTCTCTCTCTCTCTCTGTGTGT--CTCTCATGAATAAATAAATAAAATCTTTATTAAAAAAAAAGGTGCTGTACAGGGAAG
sd_chr2_64843826   : CTCCCTGCATGGAGCCTGCTTCTCCCTCTGCCTGTGTCTCTGCCTCTCTCTCTCTCTCTCTCTGTGTGTGTCTCTCATGAATAAATAAATAAAATCTTTAAAAAAAAAAAAAAAAAAAAAAAAAAAAA

sdb_chr8_3186075   : CTTGCATTTTACTCTGAAGGCTATCTAGAGACATTTCATTATTTTTTAAAATTTTATTTA
sd_chr2_64843826   : AGAGGAGGGAGAGAGAGAAAGAGGTCTGGGTTCTGAAGTCCGCGCTTGGACAGACTCTCC
```

Figure S3. Eight examples (**A**-**H**) of Can_b1Y copies illustrating their T⁺ retrotransposition. The lower line in alignments is a young Can_b1 copy present in the German Shepherd but missing in the Great Dane and/or the Boxer. The letter prefixes in sequence names indicate the genomes where they are present (s, German Shepherd; d, Great Dane; and b, Boxer). The upper line(s) represent older copies present in all three genomes with 100% identity to the younger copy. (Notice that there are no other 100% identical copies in these genomes.) Pol III terminators and poly(A) tails are marked red and yellow, respectively; TSDs are underlined.
