# Supplementary material for: Tail Wags Dog’s SINE: Retropositional Mechanisms of Can SINE Depend on Its A-Tail Structure
Source: Biology (Basel). 2022 Sep 26;11(10):1403. doi: 10.3390/biology11101403 (PMC9599045; doi:10.3390/biology11101403)
Supplement: Supplementary file 1 [file biology-11-01403-s001.zip › Figure S4.pdf]

A

Can_1_in_F8 : CGAACACAGCTTTTAAAAGGAAAGACACCGGGGATCCCTGG-TGGCGCAGCGGTTTGGCGCCTGCCTTTGGCCCGGGGCACGATCCTGGAGACCCGGGATCGAATCCCACGTCGGGCTCCCGGTGCATGGAGCTTGCTTC
chr19_13787844 : TAATGCTTTTAAATAGAAATTTGAAGAACAGGGATCCCTGGGTGGCGCAGCGGTTTGGCGCCTGCCTTTGGCCCGGGGCACGATCCTGGAGACCCGGGATCGAGTCCCACGTCGGGCTCCCGGTGCATGGAGCTTGCTTC
chr4_32931588 : CGTGAGATGCCTGAGGAAAAGAAAGTTTCAGGGATCCCTGGGTGGCGCAGCGGTTTGGCGCCTGCCTTTGGCCCGGGGCACGATCCTGGAGACCCGGGATCGAATCCCACGTCGGGCTCCCAGTGCATGGAGCTTGCTTC
chr12_42108376 : AGAAGAATTTTTTTAAATATGAAAATACCTGGGATCCCTGGGTGGCGCAGCGGTTTGGCGCCTGCCTTTGGCCCGGGGCACGATCCTGGAGACCCGGGATCGAATCCCACGTCGGGCTCCCGGTGCATGGAGCCTGCTTC
chr15_45574372 : TCAAAATATAACACTAAAGAAAGTCATCAAGGGATCCCTGGGTGGCGCAGCGGTTTGGCGCCTGCCTTTGGCCCGGGGCACGATCCTGGAGACCCGGGATCGAATCCCACGTCGGGCTCCCGGTGCATGGAGCCTGCTTC
chr2_5294782 : TTGGTATTAACAATAGAATTCACCAGGGAGGGGATCCCTGGGTGGCGCAGCGGTTTGGCGCCTGCCTTTGGCCCGGGGCACGATCCTGGAGACCCGGGATCGAATCCCACGTCGGGCTCCCAGTGCATGGAGCTTGCTTC
chr16_57112821 : CTTCCACCCCCGTGTTAAAACTGATGCTGCGGGATCCCTGGGTGGCGCAGCGGTTTGGCGCCTGCCTTTGGCCCGGGGCACGATCCTGGAGACCCGGGATCGAATCCCACGTCGGGCTCCCGGTGCATGGAGCCTGCTTC
chrUn_26084 : CATACTAAGTACTGATAAATATAGATGGAGGGGATCCCTGGGTGGCGCAGCGGTTTGGCGCCTGCCTTTGGCCCGGGGCACGATCCTGGAGACCCGGGATCGAATCCCACGTCGGGCTCCCGGTGCATGGAGCCTGCTTC

Can_1_in_F8 : TCCCTCTGCCTGTGTCTCTGCCTCTCTCTCTCTCTGTGACTATCATAAATAAATAAAAGTTAAAAAAAAAAAAAAAAAAAAAAAAATTAAAATTAAAAGGAAAGACACCATTTTGCCCCTGGGCC----
chr19_13787844 : TCCCTCTGCCTGTGTCTCTGCCTCTCTCTCTCTCTGTGACTATCATAAATAAATAAAAGTTAAAAAAAAAATTTAAAAAAAAAAAAAAAAAAAAAAAAAAAAAGAAATTTGAAGAACAGATCTCTTTAA
chr4_32931588 : TCCCTCTGCCTGTGTCTCTGCCTCTCTCTCTCTCTGTGACTATCATAAATAAATAAAAGTTAAAAAAAATTTAAAAAAAAAAAAAAAAAGAAAGTTTCAAATAGTCTGATCATTGGTAGGAAAACACAA
chr12_42108376 : TCCCTCTGCCTGTGTCTCTGCCTCTCTCTCTCTCTGTGACTATCATAAATAAATAAAAGTTAAAAAAAATTAAAAAAAAATATGAAAATACCTAGCAAGTAAAAAGATTGAAGCCATAAAAAAACAAAC
chr15_45574372 : TCCCTCTGCCTGTGTCTCTGCCTCTCTCTCTCTCTGTGACTATCATAAATAAATAAAAGTTAAAAAAAAATTAAAAAAAAAAAAAAAAGAAAGTCATCAAATCACAAGAAAGAGAGTAAAAGTAGAAAG
chr2_5294782 : TCCCTCTGCCTGTGTCTCTGCCTCTCTCTCTCTCTGTGACTATCATAAATAAATAAAAGTTAAAAAAAATTTAAAAAAAAAAAAAAAAGAATTCACCAGGGATATGTATGTGAATAGAAAAATGGATAT
chr16_57112821 : TCCCTCTGCCTGTGTCTCTGCCTCTCTCTCTCTCTGTGACTATCATAAATAAATAAAAGTTAAAAAAAAAATTAAAAAAAAAAAAAAAAACTGATGCTGAGAATACTGGCTGTCCCACATCTGGCACACT
chrUn_26084 : TCCCTCTGCCTGTGTCTCTGCCTCTCTCTCTCTCTGTGACTATCATAAATAAATAAAAGTTAAAAAAAAAAAATTAAAAAAAAAAAAAAAAAAAGAAAATAGTACAATGACTTTAGAAAACAA------

B

Can_2_in_F8 : GAAGAAACTTGATTTAAAAATTTCTAGTTCGGGATCCCTGGGTGGCGCAGCGGTTTGGCGCCTGCCTTTGGCCCAGGGCGCGATCCTGGAGACCCGGGATCGAATCCCACATCGGGCTCCTGGTGCATGGAGCCTGCTTC
chrX_54829244 : GATTTTTTTTTTTTTGAAAGCACGGTCATGGGGATCCCTGGGTGGCGCAGCGGTTTGGCGCCTGCCTTTGGCCCAGGGCGCGATCCTGGAGACCCGGGATCGAATCCCACATCGGGCTCCTGGTGCATGGAGCCTGCTTC
chrX_62403822 : -AGAGTTTTATAGTTTAAATCTTAAGTCTAGGGATCCCTGGGTGGCGCAGCGGTTTGGCGCCTGCCTTTGGCCCAGGGCGCGATCCTGGAGACCCGGGATCGAATCCCACATCGGGCTCCTGGTGCATGGAGCCTGCTTC
chrX_88161334 : GCTCATCGTTAAAATAAAAACACCTGAAAAGGGATCCCTGGGTGGCGCAGCGGTTTGGCGCCTGCCTTTGGCCCAGGGCGCGATCCTGGAGACCCGGGATCGAATCCCACATCAGGCTCCTGGTGCATGGAGCCTGCTTC
chr25_10034791 : ATAGATTAAGCGCTTAGAAAAATGTTTGGCGGGATCCCTGGGTGGCGCAGCGGTTTGGCGCCTGCCTTTGGCCCAGGGCGCGATCCTGGAGACCCGGGATCGAATCCCACATCGGGCTCCTGGTGCATGGAGCCTGCTTC
chr6_75277277 : GGCTCCCATGGAATTAAGAAAATGCTGCTGGGGATCCCTGGGTGGCGCAGCGGTTTGGCGCCTGCCTTTGGCCCAGGGCGCGATCCTGGAGACCCGGGATCGAATCCCACATCGGGCTCCTGGTGCATGGAGCCTGCTTC
chr8_25747996 : AGCAGTATAGTGATTTAAAAATTTATAACAGGGATCCCTGGGTGGCGCAGCGGTTTGGCGCCTGCCTTTGGCCCAGGGCGCGATCCTGGAGACCCGGGATCGAATCCCACATCGGGCTCCTGGTGCATGGAGCCTGCTTC
chrX_77138179 : TGCAAGATTTATTTATAAATTCTATATTCAGGGATCCCTGGGTGGCGCAGCGGTTTGGCGCCTGCCTTTGGCCCAGGGCGCGATCCTGGAGACCCGGGATCGAATCCCACATCGGGCTCCTGGTGCATGGAGCCTGCTTC
chr1_41070406 : -TAAATAAAATCTTTAAAAAACAACAGACAGGGATCCCTGGGTGGCGCAGCGGTTTGGCGCCTGCCTTTGGCCCAGGGCGCGATCCTGGAGACCCGGGATCGAATCCCACATCGGGCTCCTGGTGCATGGAGCCTGCTTC
chrUn_9932 : GTGCTACTTTGTGTAAAAAATTACATGATAGGGATCCCTGGGTGGCGCAGCGGTTTGGCGCCTGCCTTTGGCCCAGGGCGCGATCCTGGAGACCCGGGATCGAATCCCACATCGGGCTCCTGGTGCATGGAGCCTGCTTC
chrUn_71481 : CAGGGAGATAGAAATCAAAACCACAATGAGGGGATCCCTGGGTGGCGCAGCGGTTTGGCGCCTGCCTTTGGCCCAGGGCGCGATCCTGGAGACCCGGGATCGAATCCCACATCGGGCTCCTGGTGCATGGAGCCTGCTTC

Can_2_in_F8 : TCTCTCTGCCTGTGTCTCTGCCTCTCTCTCTCTCTCTGTCTGTGACTTAAATAAATAAATAATAAATAAAATATTTAAAAAAAAAAAAAAAAAAAAAAAAAAAAAAAATAAAAATTTCTAGTTCATCAGACAGT
chrX_54829244 : TCTCTCTGCCTGTGTCTCTGCCTCTCTCTCTCTCTCTGTCTGTGACTTAAATAAATAAATAATAAATAAAATATTTAAAAAAAAAAAAAAAAAAAAAAAAAAAAAGAAAGCACGGTCATAA-------------
chrX_62403822 : TCTCTCTGCCTGTGTCTCTGCCTCTCTCTCTCTCTCTGTCTGTGACTTAAATAAATAAATAATAAATAAAATATTTAAAAAAAAAAAAAATAAATAAATCTTAAGTCTAACATTTAGGGTTTTTCTTATCTATT
chrX_88161334 : TCTCTCTGCCTGTGTCTCTGCCTCTCTCTCTCTCTCTGTCTGTGACTTAAATAAATAAATAATAAATAAAATATTTAAAAAAAAACACCTGAAAAAAATAAAATAAAATAAAAACACCTGGAGCATTATGTAAT
chr25_10034791 : TCTCTCTGCCTGTGTCTCTGCCTCTCTCTCTCTC--TGTCTGTGACTTAAATAAATAAATAATAAATAAAATATTTTAAAAAAAGAAAAGAAAAATGATTGGCAGACAGTAAATGCCATATAAATGTTAGCTAT
chr6_75277277 : TCTCTCTGCCTGTGTCTCTGCCTCTCTCTCTCTCTCTGTCTGTGACTTAAATAAATAAATAATAAATAAAATATAAAAAAAAAAAAAAAAAAAAAAAAAAGAAAATGCTGCTGATGGGCAGC-------------
chr8_25747996 : TCTCTCTGCCTGTGTCTCTGCCTCTCTCTCTCTCTCTGTCTGTGACTTAAATAAATAAATAATAAATAAAATATTTAAAAAAAAAATAAAAAAAAAATTAAAAAAAATAAAAATTTATAACATAA-GTATATTT
chrX_77138179 : TCTCTCTGCCTGTGTCTCTGCCTCTCTCTCTCTCTCTGTCTGTGACTTAAATAAATAAATAATAAATAAAATATTTAAAAAAAAATAAATAAATAAATTCTATATTCAATTTCTTTGATATATATGTTATACAC
chr1_41070406 : TCTCTCTGCCTGTGTCTCTGCCTCTCTCTCTCTCTCTGTCTGTGACTTAAATAAATAAATAATAAATAAAATATTTAAAAAAAAAAAAAAAAAAAAAAAAAAACAACAGACAAAGCCTGCTGCTTTGTCTGCAT
chrUn_9932 : TCTCTCTGCCTGTGTCTCTGCCTCTCTCTCTCTCTCTGTCTGTGACTTAAATAAATAAATAATAAATAAAATATTTAAAAAAAAAAAAAAAAAAAAAAAAAAAAAAAAAAAAAATTACATGATAAAGATGACTTT
chrUn_71481 : TCTCTCTGCCTGTGTCTCTGCCTCTCTCTCTCTCTCTGTCTGTGACTTAAATAAATAAATAATAAATAAAATATTTAAAAAAAAAAAAAAAAAAACCACAATGAGATACCACCTCACACCAGTGAAAATGGG

C

Can in ASIP :
AATTCTAGAGAATTTCAGGAAGATGGTCAGGTAAGAAACACATTAGAATCAAATTTGCAGGGATCCCTGGGTGGCGCAGCGGTTTAGCGCCTGCCTTTGGCCCAGGGCGCGATCCTGGAGACCCGGGATCAAATCCCACG

CM021970.1:45110505:
CCACTTACATGGTCTCCCTGACTCAGGTGTTAAGTTGCTGTTAAGAGTGAGGTACAGGTGGGATCCCTGGGTGGCGCAGCGGTTTAGCGCCTGCCTTTGGCCCAGGGCGCGATCCTGGAGACCCGGGATCAAATCCCACG
CM021969.1:60195958:
CTGCACCAAAGGCAAGGTAAAGGACGGACAAAGAATATATCCTTTAAAACTTTGATTCTGGGATCCCTGGGTGGCGCAGCGGTTTAGCGCCTGCCTTTGGCCCAGGGCGCGATCCTGGAGACCCGGGATCAAATCCCACG
CM021970.1:41526014:
TTATCACAACAGAATGAAAAGATGTCTTCTTTAGCTCAACTAAAAGATCACAAGTTTGTGGGATCCCTGGGTGGCGCAGCGGTTTAGCGCCTGCCTTTGGCCCAGGGCGCGATCCTGGAGACCCGGGATCAAATCCCACG
CM021962.1:70217506:
ATTAATGCAATCAGCGATATGAACAGGCTAGAGAAGAAAAGCATTTGAATAGAAACATAGGGATCCCTGGGTGGCGCAGCGGTTTAGCGCCTGCCTTTGGCCCAGGGCGCGATCCTGGAGACCCGGGATCAAATCCCACG
CM021980.1:44692371:
TGGTGCTAAATGACCTTTGGAAATTTTCTTTAGGATGGTATTTACAGATAAGAAGTCCAGGGATCCCTGGGTGGCGCAGCGGTTTAGCGCCTGCCTTTGGCCCAGGGCGCGATCCTGGAGACCCGGGATCAAATCCCACG
CM021991.1:22294944:
GTCTGCCAAAATGACGTGATTGTCATCTTGATTATGGTCAAACATTAAATTGTTAATGTGGGATCCCTGGGTGGCGCAGCGGTTTAGCGCCTGCCTTTGGCCCAGGGCGCGATCCTGGAGACCCGGGATCAAATCCCACG
CM021977.1:17215410:
GCCAAAATTCTGTAGTAATAAGTACCTTCAATTAAAGAAAAAATAAAAATTAACATCCAGGGATCCCTGGGTGGCGCAGCGGTTTAGCGCCTGCCTTTGGCCCAGGGCGCGATCCTGGAGACCCGGGATCAAATCCCACG
CM021985.1:23875635:
AATTCTAGAGAATTTCAGGAAGATGGTCAGGTAAGAAACACATTAGAATCAAATTTGCAGGGATCCCTGGGTGGCGCAGCGGTTTAGCGCCTGCCTTTGGCCCAGGGCGCGATCCTGGAGACCCGGGATCAAATCCCACG
JAAHUQ010001485.1 :
CCAGGTCCTGGCAACGGAGAGATGGACCAGATATATTTCTTGCTCAGAAGTGACTTTCAGGGATCCCTGGGTGGCGCAGCGGTTTAGCGCCTGCCTTTGGCCCAGGGCGCGATCCTGGAGACCCGGGATCAAATCCCACG
CM021976.1:54831480:
ACCCATCCAGGCAGACACAAACACCTGCATATATACATACATAAAAATAGAGACAGATTGGGATCCCTGGGTGGCGCAGCGGTTTAGCGCCTGCCTTTGGCCCAGGGCGCGATCCTGGAGACCCGGGATCAAATCCCACG
CM021986.1:29276547:
CTTTCTGATTTCAGTAATTGGTGTGCGCCACGAGGATTTTTTAAAAAAGAAAAATGTAGGGATCCCTGGGTGGCGCAGCGGTTTAGCGCCTGCCTTTGGCCCAGGGCGCGATCCTGGAGACCCGGGATCAAATCCCACG

Can in ASIP : TCAGGCTCCCGGTGCATGGAGCCTGCTTCTCCCTCTGCCTGTGTCTCTGC------------CTCTCTCTCTCTCTCTCTC--
TGTGTGACTATCATAAATAAATGAAAAAAAAAAAAAAGGAAAAAAAAAAAAAAAAAA
CM021970.1:45110505: TCAGGCTCCCGGTGCATGGAGCCTGCTTCTCCCTCTGCCTGTGTCTCTGC----------
CTCTCTCTCTCTCTCTCTCTCTGTGTGTGACTATCATAAATAAATGAAAAAAAAAAAAAAAGACTGAGGTACAGGGCAAG
CM021969.1:60195958: TCAGGCTCCCGGTGCATGGAGCCTGCTTCTCCCTCTGCCTGTGTCTCTGC--CTCTCTCTCTCTCTCTCTCTCTCTCTCTC--
TGTGTGACTATCATAAATAAATAAAAATTTAAAAAAATAAAATAAAATAAAACTTTG
CM021970.1:41526014: TCAGGCTCCCGGTGCATGGAGCCTGCTTCTCCCTCTGCCTGTGTCTCTGC--------------CTCTCTCTCTCTCTCTC--
TGTGTGACTATCATAAATAAATGAAAAAAAAAAAATGAAAAAAAAAAGATCACAAGT
CM021962.1:70217506: TCAGGCTCCCGGTGCATGGAGCCTGCTTCTCCCTCTGCCTGTGTCTCTGC------------CTCTCTCTCTCTCTCTCTC--
TGTGTGACTATCATAAATAAATAAAAATTAAAAAAAAATGAATAGAAACATAAAATG
CM021980.1:44692371: TCAGGCTCCCGGTGCATGGAGCCTGCTTCTCCCTCTGCCTGTGTCTCTGC------------CTCTCTCTCTCTCTCTCTC--
TCTGTGACTATCATAAATAAATGAAAAAAAAAAAAATGAAAAAAAAAAAAAAAAAAA
CM021991.1:22294944: TCAGGCTCCCGGTGCATGGAGCCTGCTTCTCCCTCTGCCTGTGTCTCTGC----------CTCTCTCTCTCTCTCTCTCTC--
TGTGTGACTATCATAAATAAATGAAAAAAAAAAATAATAAATTGTTAATGTAACATA
CM021977.1:17215410: TCAGGCTCCCGGTGCATGGAGCCTGCTTCTCCCTCTGCCTGTGTCTCTGC------------CTCTCTCTCTCTCTCTCTC--
TGTGTGACTATCATAAATAAATGAAAAAAAAAAAAAAAAAAATGAAAAAAAAAAAAA
CM021985.1:23875635: TCAGGCTCCCGGTGCATGGAGCCTGCTTCTCCCTCTGCCTGTGTCTCTGC------------CTCTCTCTCTCTCTCTCTC--
TGTGTGACTATCATAAATAAATGAAAAAAAAAAAAAAAGAAAAAAAAAAAAAAAAAA
JAAHUQ010001485.1 : TCAGGCTCCCGGTGCATGGAGCCTGCTTCTCCCTCTGCCTGTGTCTCTGC--------------CTCTCTCTCTCTCTCTC--
TGTGTGACTATCATAAATAAATGAAGAAAAAAAAAAAAGACTGAGGTACAGGGCAAG
CM021976.1:54831480: TCAGGCTCCCGGTGCATGGAGCCTGCTTCTCCCTCTGCCTGTGTCTCTGC----------CTCTCTCTCTCTCTCTCTCTC--
TGTGTGACTATCATAAATAAATGAAAAAAAAAATGAAAAAAAAAATAGAGACAGATT
CM021986.1:29276547: TCAGGCTCCCGGTGCATGGAGCCTGCTTCTCCCTCTGCCTGTGTCTCTGCCTCTCTCTCTCTCTCTCTCTCTCTCTCTCTC--
TCTGTGACTATCATAAATAAATAATAAAAAAAAAAAGAAAAATGTAAACTGTAGTGA

Can in ASIP : AAAAAAAAAAAAAAAAAAGAATCAAATTTGCAAAAGATAAGACAAATGTAGATTCTTGAAATTAAGCA
CM021970.1:45110505: CCACTCTGGTGGGCTTTCTTACATAGTGAGCTTTCTGCAAACATTTTTATGCTCAAACTTCATGGTTA
CM021969.1:60195958: ATTCTTGTAAACTCCAATTAGTAATTGAAAGAGACCTTACAGCATCTCTGTACCAGAGACTTAGAGTT
CM021970.1:41526014: TTGTGCTACTCAATAATCTTCCATAACAATGCTCTGGAGCAGCAACAAAAACAGCTGACATCAAAAAC
CM021962.1:70217506: AATTTGACAAAATCCAATATGCATTCATTCATACACAAAAAAACTCTCTCAGGAAATAAGGTATAGAG
CM021980.1:44692371: AAGAAGTCCAAAGTTAACATGGCAGATTCTTCTTGAATGTTGTCTGAGAGAAAGACCTTCCTCTGCCC

```
CM021991.1:22294944: AGATTTCATAACCTGCATACAGTTCAGATATTCTGTGGCAGCTGCTCAGCCCAAAGTGCCATCCAAAG
CM021977.1:17215410: TTAACATCCAACTCACAAACACAAAGACAAAAACAAAGGAAAGTAGAAAAAAAAGAAATAAAGATAAA
CM021985.1:23875635: AAAAGAATCAAATTTGCAAAAGATAAGACAAATGTAGATTCTTGAAATTAGCAAGAGAGAAGCTACTA
JAAHUQ010001485.1  : CCACTCTGGTGGGCTTTCTTAAAAAAAAAAAAAAAAAAAAGAAAGTGACTTTCATGTAAAGAAAACAA
CM021976.1:54831480: GATTGTACAAAGCAACTAAAAGATGATACTAGAATTATTTGGTTAGTTAAAACTAAAATTCTCTAATA
CM021986.1:29276547: TAGAAAAAGTTGTTTTAAAAATTACCATAAAAAAAAATAAAAAAAATAAAAATAAAAATAAAAATTACCA
```

D

```
Can_in_ATP1B2  :ATAGAAGACGAGGTAGAAGAGGAGGATGAGGGGATCCCTGGGTGGTGCAGCGGTTTGGCGCCTGCCTTTGGCCCAGGGCGCGATCCTGGAGACCCGGGATCGAATCCCACATCGGGCTCCCAGTGCGTGGAGCCTGCTTC
chr36_14925349:TTACTGTTACATTAAGAATAGGGTCTTTCCGGGATCCCTGGGTGGTGCAGCGGTTTGGCGCCTGCCTTTGGCCCAGGGCGCGATCCTGGAGACCCGGGATCGAATCCCACATCGGGCTCCCAGTGCGTGGAGCCTGCTTC
chrX_19953333  :TAAAGGAAGCTTCATTTAAAATGAGGTTGAGGGATCCCTGGGTGGTGCAGCGGTTTGGCGCCTGCCTTTGGCCCAGGGCGCGATCCTGGAGACCCGGGATCGAATCCCACATCGGGCTCCCAGTGCATGGAGCCTGCTTC

Can_in_ATP1B2  :TCCCTCTGCCTGTGTCTCTGCCTCTCTCTCTCT--GTGACTATCATGAATAAATAAATAAAA-TCTTTAAAAAAAAAAAAAAAAAAAAAAAAAAAAAAAAAAAGAAGAGGAGGATGAAGGCTAGGGAGGAGG
chr36_14925349:TCCCTCTGCCTGTGTCTCTGCCTCTCTCTCTCT--GTGACTATCATGAATAAATAAATAAAA-TCTTTAAAAAAAAAAAAAAATAGGGTCTTTCCTAAAAGAGAACAAGCAGGAGCCAGTAAATTTCCTAGT
chrX_19953333  :TCCCTCTGCCTGTGTCTCTGCCTCTCTCTCTCTCTGTGACTATCATGAATAAATAAATAAAAATCTTTAAAAAAATAAAAAAATAAATAAAAAAAAAAAATAAAATGAGGTTGAGAGGCCCTGAAGGGGGAGCT
```

E

```
Can_in_PTPLA     : TGAATACTTTTTATATAAACCTTTGTGTGGGGGATCCCTGGGTGGCGCAGCGGTTTGGCGCCTGCCTTTGGCCCAGGGCGCGATCCTGGAGACCCGGGATCGAGTCCCACGTCGGGCTCCCGGTGCATGGAGCCTGCTTC
chr6_751818      : ATGCCCCCAAAATTAAATTAGAAAAATTCCGGGATCCCTGGGTGGCGCAGCGGTTTGGCGCCTGCCTTTGGCCCAGGGCGCGATCCTGGAGACCCGGGATCGAGTCCCACGTCGGGCTCCCGGTGCATGGAGCCTGCTTC
chr7_7763922     : CTTCTACATCTTTTTAAATCAGGTTGTTTGGGGATCCCTGGGTGGCGCAGCGGTTTGGCGCCTGCCTTTGGCCCAGGGCGCGATCCTGGAGACCCGGGATCGAGTCCCACGTCGGGCTCCCGGTGCATGGAGCCTGCTTC
chr10_46880259   : GCATCCTTTGTCAAAAATTCTGTTTTATAGGGGATCCCTGGGTGGCGCAGCGGTTTGGCGCCTGCCTTTGGCCCAGGGCGCGATCCTGGAGACCCGGGATCGAGTCCCACGTCGGGCTCCCGGTGCATGGAGCCTGCTTC
chr14_6003797    : ATTGCACTTGACATTAAAAAGGCAACGTAGGGGATCCCTGGGTGGCGCAGCGGTTTGGCGCCTGCCTTTGGCCCAGGGCGCGATCCTGGAGACCCGGGATCGAGTCCCACGTCGGGCTCCCGGTGCATGGAGCCTGCTTC
chr33_24974268   : TCTACAGTAAATTTAAAAATTTATAAACAGGGGATCCCTGGGTGGCGCAGCGGTTTGGCGCCTGCCTTTGGCCCAGGGCGCGATCCTGGAGACCCGGGATCGAGTCCCACGTCGGGCTCCCGGTGCATGGAGCCTGCTTC
chrUn_MU018737   : ATCATAAAAAAAAAATTTAAAAAGAAGTAGGGGATCCCTGGGTGGCGCAGCGGTTTGGCGCCTGCCTTTGGCCCAGGGCGCGATCCTGGAGACCCGGGATCGAGTCCCACGTCGGGCTCCCGGTGCATGGAGCCTGCTTC

Can_in_PTPLA     : TCCCTCTGCCTGTGTCTCTGCCTCTCTCTCTATCTGTGTGTGACTATCATAAATAAATAAAAAAAATTAAAAAAAAAAAAATTTAAAAAAAAAAAATTTAAAAAAAAAAAAAAAAAACCTTTGTGTGTTCCTTTTTC
chr6_751818      : TCCCTCTGCCTGTGTCTCTGCCTCTCTCTCTATCTGTGTGTGACTATCATAAATAAATAAAAA---TTAAAAAAAAAATAAATAAATAAATTAGAAAAATTCCTTCCATAAAGGGTGACTTTCTGGGG-------
chr7_7763922     : TCCCTCTGCCTGTGTCTCTGCCTCTCTCTCTATCTGTGTGTGACTATCATAAATAAATAAAAA---TTAAAAAAAAATTTTTTTAAATCAGGTTGTTTGGCTTTTTCTTAGAAGGGAAGTGTTGGGG-------
chr10_46880259   : TCCCTCTGCCTGTGTCTCTGCCTCTCTCTCTATCTGTGTGTGACTATCATAAATAAATAAAAA---TTAAAAAAAAAATTCTGTTTTATCATTTAGGGAAGTGTATCTTTGCCCCAGAGGAGAGCATA------
chr14_6003797    : TCCCTCTGCCTGTGTCTCTGCCTCTCTCTCTATCTGTGTGTGACTATCATAAATAAATAAAAA---TTAAAAAAAAAAAAAAAAAGGCAACGTGTAAGCTCATTAACTCAGACAAGCAGGGACTAGTGAGGAAA
chr33_24974268   : TCCCTCTGCCTGTGTCTCTGCCTCTCTCTCTATCTGTGTGTGACTATCATAAATAAATAAAAA---TTTAAAAAAAAAAAATAAAATAAAAAAATAAATAAAAATTTATAAACAGAGATGCCTGGGTGGCTCAGC
chrUn_MU018737   : TCCCTCTGCCTGTGTCTCTGCCTCTCTCTCTATCTGTGTGTGACTATCATAAATAAATAAAAA---TTAAAAAAAAAAAAAAATTAAAAAGAAGTTAAAAATAGCGCTATGCTACGATTCCCAAATTGCA----
```

F

```
Can_in_STK38L    : TCCTCTTGAGCCGTAAGAACTCTGTTTCCGGGGATCCCTGGGTGGCGCAGCGGTTTGGCGCCTGCCTTTGGCCCAGGGCGCGGCCCTGGATATCCAGGATCGAATCCCACGTCAGGCTCCCGGTGCATGGAGCCTGCTTC
chr34_2829727    : GGGAAAAAGGTTTAAAGATCTATCTAATGTGGGATCCCTGGGTGGCGCAGCGGTTTGGCGCCTGCCTTTGGCCCAGGGCGCGGCCCTGGATATCCAGGATCGAATCCCACGTCAGGCTCCCGGTGCATGGAGCCTGCTTC
chr8_36794809    : CTCTCTGACCTCCTTAAAATCCTAGAACTCGGGATCCCTGGGTGGCGCAGCGGTTTGGCGCCTGCCTTTGGCCCAGGGCGCGGCCCTGGATATCCAGGATCGAATCCCACGTCAGGCTCCCGGTGCATGGAGCCTGCTTC
chr14_34036119   : ACTTTATGTGTGGTTTTAAATTTCTTTACAGGGATCCCTGGGTGGCGCAGCGGTTTGGCGCCTGCCTTTGGCCCAGGGCGCGGCCCTGGATATCCGGGATCGAATCCCACGTCAGGCTCCCGGTGCATGGAGCCTGCTTC

Can_in_STK38L    : TCCCTCTGCCTGTGTCTCTGCCTCTCTCTCTCTCTCACTGTGTGCCTATCATAAATGAATAAAAGTTTAAAAAAAAAATTTTAAAAAAAAAAAAAAAAAAAAAAAAAAGAACTCTGTTTCCTTTCGGGCA
chr34_2829727    : TCCCTCTGCCTGTGTCTCTGCCTCTCTCTCTCTCTCACTGTGTGCCTATCATAAATGAATAAAAGTTTAAAAAAAATTTTAAAAAAAATAGACTTTAAAGATCTATCTAATGTAAAAACATTAGTTGGGAG
chr8_36794809    : TCCCTCTGCCTGTGTCTCTGCCTCTCTCTCTCTCTCACTGTGTGCCTATCATAAATGAATAAAAGTTTAAAAAAAAAAATCCTAGAACTCTAGAATTTTCAAAGTTGAAGGGAACTTTGAACATCAGCC--
chr14_34036119   : TCCCTCTGCCTGTGTCTCTGCCTCTCTCTCTCTCTCACTGTGTGCCTATCATAAATGAATAAAAGTTTAAAAAAAAAAAAAAAATAAATAAATTTCTTTACAAAAATTATGGATTAAAAAAGGAAAACAGTA
```

G

```
Can in FAM161          : CTTTCCTTTTCACTCTTTCTAAAATCAAATAAAGGGATCCCTGGGTGGCGCAGCGGTTTGGCGCCTGCCTTTGGCCCAGGGCGCGATCCTGGAGACCCGGGATCGAATCCCACATCGGGCTCCCGGTGCATGGAGCCTGCTTCTCCCTCT
CM021962.1:34955691 : TAATCAAAGACTCACATGAAATTGAAAAATAATGGGATCCCTGGGTGGCGCAGCGGTTTGGCGCCTGCCTTTGGCCCAGGGCGCGATCCTGGAGACCCGGGATCGAATCCCACATCGGGCTCCCGGTGCATGGAGCCTGCTTCTCCCTCT
CM021986.1:11818367 : ATTTCATCtttttAAGTTAAAAAAATAAAACCAGGGATCCCTGGGTGGCGCAGCGGTTTGGCGCCTGCCTTTGGCCCAGGGCGCGATCCTGGAGACCCGGGATCGAATCCCACATCGGGCTCCCGGTGCATGGAGCCTGCTTCTCCCTCT
CM021983.1:36632009 : CTTTCAGGTAttatTAAGAATAACTATGAACGGGGGATCCCTGGGTGGCGCAGCGGTTTGGCGCCTGCCTTTGGCCCAGGGCGCGATCCTGGAGACCCGGGATCGAATCCCACATCGGGCTCCCGGTGCATGGAGCCTGCTTCTCCCTCT
CM021964.1:13942518 : AGATGCAGAGCATTATTCAAGAGTTGATAATTCGGGATCCCTGGGTGGCGCAGCGGTTTGGCGCCTGCCTTTGGCCCAGGGCGCGATCCTGGAGACCCGGGATCGAATCCCACATCGGGCTCCCGGTGCATGGAGCCTGCTTCTCCCTCT
CM021972.1:8203613  : ATAAAGATGGTTGATTTTAGAAAAACAACTGCTGGGATCCCTGGGTGGCGCAGCGGTTTGGCGCCTGCCTTTGGCCCAGGGCGCGATCCTGGAGACCCGGGATCGAATCCCACATCGGGCTCCCGGTGCATGGAGCCTGCTTCTCCCTCT
CM021972.1:38978442 : CGGTAAAATGCAGATGTAAGAATGTTTATAGGTGGGATCCCTGGGTGGCGCAGCGGTTTGGCGCCTGCCTTTGGCCCAGGGCGCGATCCTGGAGACCCGGGATCGAATCCCACATCGGGCTCCCGGTGCATGGAGCCTGCTTCTCCCTCT
CM021975.1:12729399 : AAGGCATAACACACTTTAATAAAAAATAGATCAGGGATCCCTGGGTGGCGCAGCGGTTTGGCGCCTGCCTTTGGCCCAGGGCGCGATCCTGGAGACCCGGGATCGAATCCCACATCGGGCTCCCGGTGCATGGAGCCTGCTTCTCCCTCT
CM021969.1:33998971 : CCTGGGTGGCGCATGGGATCCCTGGGTGGCGCAGGGATCCCTGGGTGGCGCAGCGGTTTGGCGCCTGCCTTTGGCCCAGGGCGCGATCCTGGAGACCCGGGATCGAATCCCACATCGGGCTCCCGGTGCATGGAGCCTGCTTCTCCCTCT
CM021971.1:57427098 : CACAGTGCTAAAATAGTAGTTAAAAATAGTCACGGGATCCCTGGGTGGCGCAGCGGTTTGGCGCCTGCCTTTGGCCCAGGGCGCGATCCTGGAGACCCGGGATCGAATCCCACATCGGGCTCCCGGTGCATGGAGCCTGCTTCTCCCTCT
CM021965.1:63222756 : GGTGGCATATCTCTGCTTAAAACAAATATGGTTGGGATCCCTGGGTGGCGCAGCGGtttGGCGCCTGCCtttGGCCCAGGGCGCGATCCTGGAGACCCGGGATCGAATCCCACATCGGGCTCCCGGTGCATGGAGCCTGCTTCTCCCTCT
CM021970.1:45571260 : GATACCAAGAAGGTGGTGAAAAAGCACAACATAGGGATCCCTGGGTGGCGCAGCGGTTTGGCGCCTGCCTTTGGCCCAGGGCGCGATCCTGGAGACCCGGGATCGAATCCCACATCGGGCTCCCGGTGCATGGAGCCTGCTTCTCCCTCT
CM022000.1:57918954 : AACCATTAGCCAACCTTATTAAAAAGAAGAGAGGGGATCCCTGGGTGGCGCAGCGGTTTGGCGCCTGCCTTTGGCCCAGGGCGCGATCCTGGAGACCCGGGATCGAATCCCACATCGGGCTCCCGGTGCATGGAGCCTGCTTCTCCCTCT
CM021965.1:57990141 : AGTATGTTTTCTCTTTGTTAAAAAGGCACATTAGGGATCCCTGGGTGGCGCAGCGGTTTGGCGCCTGCCTTTGGCCCAGGGCGCGATCCTGGAGACCCGGGATCGAATCCCACATCGGGCTCCCGGTGCATGGAGCCTGCTTCTCCCTCT
CM021987.1:23159322 : TGTGCAGTACTTGAATTAATATAAGACAAAGAGGGGATCCCTGGGTGGCGCAGCGGTTTGGCGCCTGCCTTTGGCCCAGGGCGCGATCCTGGAGACCCGGGATCGAATCCCACATCGGGCTCCCGGTGCATGGAGCCTGCTTCTCCCTCT
CM021963.1:53601695 : AATATTCACAGCTCACAAAAGAACACCTAAACTGGGATCCCTGGGTGGCGCAGCGGTTTGGCGCCTGCCTTTGGCCCAGGGCGCGATCCTGGAGACCCGGGATCGAATCCCACATCGGGCTCCCGGTGCATGGAGCCTGCTTCTCCCTCT
CM021991.1:3332171  : AATTTACATATCCTCATTAAAGAACACAGACTAGGGATCCCTGGGTGGCGCAGCGGTTTGGCGCCTGCCTTTGGCCCAGGGCGCGATCCTGGAGACCCGGGATCGAATCCCACATCGGGCTCCCGGTGCATGGAGCCTGCTTCTCCCTCT
CM021981.1:4892108  : CCACACAAAGTATCATTAAAAAAAAAAAAAGAGGGGATCCCTGGGTGGCGCAGCGGTTTGGCGCCTGCCTTTGGCCCAGGGCGCGATCCTGGAGACCCGGGATCGAATCCCACATCGGGCTCCCGGTGCATGGAGCCTGCTTCTCCCTCT
CM021986.1:37118882 : TCCTAGTAAATTGCTATAAAGACAGCGATTAGGGGGATCCCTGGGTGGCGCAGCGGTTTGGCGCCTGCCTTTGGCCCAGGGCGCGATCCTGGAGACCCGGGATCGAATCCCACATCGGGCTCCCGGTGCATGGAGCCTGCTTCTCCCTCT
CM021976.1:16004606 : CTTGGTTGGGGCCTGCTCAAAACCTAAGGCAGAGGGATCCCTGGGTGGCGCAGCGGTTTGGCGCCTGCCTTTGGCCCAGGGCGCGATCCTGGAGACCCGGGATCGAATCCCACATCGGGCTCCCGGTGCATGGAGCCTGCTTCTCCCTCT
CM021998.1:22750779 : TGATCGGAGAAGGTCACTCAGAAAGAGGTGTTTGGGATCCCTGGGTGGCGCAGCGGTTTGGCGCCTGCCTTTGGCCCAGGGCGCGATCCTGGAGACCCGGGATCGAATCCCACATCGGGCTCCCGGTGCATGGAGCCTGCTTCTCCCTCT
CM021970.1:17501127 : ATGGATATTTTATACATAGAACACAACTGCTTAGGGATCCCTGGGTGGCGCAGCGGTTTGGCGCCTGCCTTTGGCCCAGGGCGCGATCCTGGAGACCCGGGATCGAATCCCACATCGGGCTCCCGGTGCATGGAGCCTGCTTCTCCCTCT
CM021963.1:35702036 : ATATAGAATTCTAGATTTGAAATTACTTTTCTTGGGATCCCTGGGTGGCGCAGCGGTTTGGCGCCTGCCTTTGGCCCAGGGCGCGATCCTGGAGACCCGGGATCGAATCCCACATCGGGCTCCCGGTGCATGGAGCCTGCTTCTCCCTCT
CM021972.1:32305978 : CATTTATTTGGACACTTTTTAAAAATCTATTTAGGGATCCCTGGGTGGCGCAGCGGTTTGGCGCCTGCCTTTGGCCCAGGGCGCGATCCTGGAGACCCGGGATCGAATCCCACATCGGGCTCCCGGTGCATGGAGCCTGCTTCTCCCTCT
CM021983.1:53301297 : ATAGTATGGAGTTTCTTCAAAAAATTAAAAATAGGGATCCCTGGGTGGCGCAGCGGTTTGGCGCCTGCCTTTGGCCCAGGGCGCGATCCTGGAGACCCGGGATCGAATCCCACATCGGGCTCCCGGTGCATGGAGCCTGCTTCTCCCTCT
CM021968.1:10325141 : ATTTTATTTTATTTTTAAATTTTGTTTTTTTTAGGGATCCCTGGGTGGCGCAGCGGTTTGGCGCCTGCCTTTGGCCCAGGGCGCGATCCTGGAGACCCGGGATCGAATCCCACATCGGGCTCCCGGTGCATGGAGCCTGCTTCTCCCTCT
CM021964.1:30661009 : GTGGTCTCTGAAATATTTATAAAAACTGCTGTCGGGATCCCTGGGTGGCGCAGCGGTTTGGCGCCTGCCTTTGGCCCAGGGCGCGATCCTGGAGACCCGGGATCGAATCCCACATCGGGCTCCCGGTGCATGGAGCCTGCTTCTCCCTCT
CM021978.1:18216705 : TTACCTCTTCTTTACGTAAAAAAAATTTGTGTTGGGATCCCTGGGTGGCGCAGCGGTTTGGCGCCTGCCTTTGGCCCAGGGCGCGATCCTGGAGACCCGGGATCGAATCCCACATCGGGCTCCCGGTGCATGGAGCCTGCTTCTCCCTCT
CM021966.1:47327703 : CTAAATAAAGTTTGTTATTGAAAGCTAAAAATCGGGATCCCTGGGTGGCGCAGCGGTTTGGCGCCTGCCTTTGGCCCAGGGCGCGATCCTGGAGACCCGGGATCGAATCCCACATCGGGCTCCCGGTGCATGGAGCCTGCTTCTCCCTCT
CM021970.1:55413363 : GGGTTGGAGGCCTTCCTTAAGACCACAGCATTTGGGATCCCTGGGTGGCGCAGCGGTTTGGCGCCTGCCTTTGGCCCAGGGCGCGATCCTGGAGACCCGGGATCGAATCCCACATCGGGCTCCCGGTGCATGGAGCCTGCTTCTCCCTCT
CM021966.1:60666245 : CACATTCTGTTGCAAATGTTTAAAATTTTCTAGGGGATCCCTGGGTGGCGCAGCGGTTTGGCGCCTGCCTTTGGCCCAGGGCGCGATCCTGGAGACCCGGGATCGAATCCCACATCGGGCTCCCGGTGCATGGAGCCTGCTTCTCCCTCT
CM021998.1:2561342  : AGAATTAACAAGATTCATGGAAACTAATGATAAGGGATCCCTGGGTGGCGCAGCGGTTTGGCGCCTGCCTTTGGCCCAGGGCGCGATCCTGGAGACCCGGGATCGAATCCCACATCGGGCTCCCGGTGCATGGAGCCTGCTTCTCCCTCT
CM021966.1:57346575 : CTTCACATTCACATATACAAAATCTAAATAATTGGGATCCCTGGGTGGCGCAGCGGTTTGGCGCCTGCCTTTGGCCCAGGGCGCGATCCTGGAGACCCGGGATCGAATCCCACATCGGGCTCCCGGTGCATGGAGCCTGCTTCTCCCTCT
CM022000.1:5548874  : GGTATGCACAGGATCTCAAAATGCATGTTTCCCGGGATCCCTGGGTGGCGCAGCGGTTTGGCGCCTGCCTTTGGCCCAGGGCGCGATCCTGGAGACCCGGGATCGAATCCCACATCGGGCTCCCGGTGCATGGAGCCTGCTTCTCCCTCT
CM021987.1:24241989 : ACTGGAACAGGAAGAAATAGAAAACCTTAACAGGGGATCCCTGGGTGGCGCAGCGGTTTGGCGCCTGCCTTTGGCCCAGGGCGCGATCCTGGAGACCCGGGATCGAATCCCACATCGGGCTCCCGGTGCATGGAGCCTGCTTCTCCCTCT
CM021989.1:1299907  : TGCTAAATAATTGTTGTTTAAGTCACCAGTCTAGGGATCCCTGGGTGGCGCAGCGGTTTGGCGCCTGCCTTTGGCCCAGGGCGCGATCCTGGAGACCCGGGATCGAATCCCACATCGGGCTCCCGGTGCATGGAGCCTGCTTCTCCCTCT
CM021976.1:5736094  : AAGTGTGGAGTGACGTTTAAAAATGAAAACGTCGGGATCCCTGGGTGGCGCAGCGGTTTGGCGCCTGCCTTTGGCCCAGGGCGCGATCCTGGAGACCCGGGATCGAATCCCACATCGGGCTCCCGGTGCATGGAGCCTGCTTCTCCCTCT
CM021969.1:31664805 : AGATTTTATTTATCTTTTTAAAAGTAATCTCTGGGGATCCCTGGGTGGCGCAGCGGTTTGGCGCCTGCCTTTGGCCCAGGGCGCGATCCTGGAGACCCGGGATCGAATCCCACATCGGGCTCCCGGTGCATGGAGCCTGCTTCTCCCTCT
CM021984.1:50603247 : GTGATTCATCAGTTGTATATAATACCCAGTGCTGGGATCCCTGGGTGGCGCAGCGGTTTGGCGCCTGCCTTTGGCCCAGGGCGCGATCCTGGAGACCCGGGATCGAATCCCACATCGGGCTCCCGGTGCATGGAGCCTGCTTCTCCCTCT
CM021962.1:57612401 : GTCTAATATGAAATGGATTAAGAGCAGAGTCAAGGGATCCCTGGGTGGCGCAGCGGTTTGGCGCCTGCCTTTGGCCCAGGGCGCGATCCTGGAGACCCGGGATCGAATCCCACATCGGGCTCCCGGTGCATGGAGCCTGCTTCTCCCTCT
CM021976.1:38824663 : AAAAAAAAAAAAAAAAAAAAAAAAAAAAAAAAGGGATCCCTGGGTGGCGCAGCGGTTTGGCGCCTGCCTTTGGCCCAGGGCGCGATCCTGGAGACCCGGGATCGAATCCCACATCGGGCTCCCGGTGCATGGAGCCTGCTTCTCCCTCT
CM021983.1:612810   : ACAAGTGAAATCAGATTAAAGAACATGAAGGATGGGATCCCTGGGTGGCGCAGCGGTTTGGCGCCTGCCTTTGGCCCAGGGCGCGATCCTGGAGACCCGGGATCGAATCCCACATCGGGCTCCCGGTGCATGGAGCCTGCTTCTCCCTCT
CM021984.1:7474507  : ACATGCTTGAAACGGGTAAGAAAACAAAATCTCGGGATCCCTGGGTGGCGCAGCGGTTTGGCGCCTGCCTTTGGCCCAGGGCGCGATCCTGGAGACCCGGGATCGAATCCCACATCGGGCTCCCGGTGCATGGAGCCTGCTTCTCCCTCT
CM021981.1:55525507 : AGAAAAGAGTCTTTTTAAGAACTGAAGATGGAGGGGATCCCTGGGTGGCGCAGCGGTTTGGCGCCTGCCTTTGGCCCAGGGCGCGATCCTGGAGACCCGGGATCGAATCCCACATCGGGCTCCCGGTGCATGGAGCCTGCTTCTCCCTCT
CM021972.1:27660724 : CAAAAGATAAAGTCTTTTTAAAAGCCAGAGGTGGGGATCCCTGGGTGGCGCAGCGGTTTGGCGCCTGCCTTTGGCCCAGGGCGCGATCCTGGAGACCCGGGATCGAATCCCACATCGGGCTCCCGGTGCATGGAGCCTGCTTCTCCCTCT
CM021968.1:44424747 : CCAAGAAATACATTTATATGAGAATATTATGAGGGGATCCCTGGGTGGCGCAGCGGTTTGGCGCCTGCCTTTGGCCCAGGGCGCGATCCTGGAGACCCGGGATCGAATCCCACATCGGGCTCCCGGTGCATGGAGCCTGCTTCTCCCTCT
CM021986.1:18354655 : ATGTCTTTGGTGTTATTTAAAAAGTCAACACCAGGGATCCCTGGGTGGCGCAGCGGTTTGGCGCCTGCCTTTGGCCCAGGGCGCGATCCTGGAGACCCGGGATCGAATCCCACATCGGGCTCCCGGTGCATGGAGCCTGCTTCTCCCTCT
CM021980.1:17876643 : GAGCCAGGATGGGGAATTAAAAAAAGAGAGAAGGGGATCCCTGGGTGGCGCAGCGGtttGGCGCCTGCCtttGGCCCAGGGCGCGATCCTGGAGACCCGGGATCGAATCCCACATCGGGCTCCCGGTGCATGGAGCCTGCTTCTCCCTCT
CM021980.1:41754496 : GAAAGACACTAACCAGGATGGAAAGGAAAACTAGGGATCCCTGGGTGGCGCAGCGGTTTGGCGCCTGCCTTTGGCCCAGGGCGCGATCCTGGAGACCCGGGATCGAATCCCACATCGGGCTCCCGGTGCATGGAGCCTGCTTCTCCCTCT
CM021962.1:7987457  : ACATGTGTCTGCTGCCTTAAAAATACTACTCCTGGGATCCCTGGGTGGCGCAGCGGTTTGGCGCCTGCCTTTGGCCCAGGGCGCGATCCTGGAGACCCGGGATCGAATCCCACATCGGGCTCCCGGTGCATGGAGCCTGCTTCTCCCTCT
CM021973.1:59099445 : TTGCTTGGACTCTTATTAGTCATATTCAGTCTAGGGATCCCTGGGTGGCGCAGCGGTTTGGCGCCTGCCTTTGGCCCAGGGCGCGATCCTGGAGACCCGGGATCGAATCCCACATCGGGCTCCCGGTGCATGGAGCCTGCTTCTCCCTCT
CM021983.1:4637355  : TTCCATGTATGCCTGAAAAGAATGTTTGTTCTTGGGATCCCTGGGTGGCGCAGCGGTTTGGCGCCTGCCTTTGGCCCAGGGCGCGATCCTGGAGACCCGGGATCGAATCCCACATCGGGCTCCCGGTGCATGGAGCCTGCTTCTCCCTCT
CM021986.1:24336044 : CAATTTTTTTTTTTTTTTTAAATAACAGCATAAGGGATCCCTGGGTGGCGCAGCGGTTTGGCGCCTGCCTTTGGCCCAGGGCGCGATCCTGGAGACCCGGGATCGAATCCCACATCGGGCTCCCGGTGCATGGAGCCTGCTTCTCCCTCT
CM021970.1:15683527 : CTTTCTAGGAACTGTGGCATAAAACTGTGGTGAGGGATCCCTGGGTGGCGCAGCGGTTTGGCGCCTGCCTTTGGCCCAGGGCGCGATCCTGGAGACCCGGGATCGAATCCCACATCGGGCTCCCGGTGCATGGAGCCTGCTTCTCCCTCT
CM021979.1:3742243  : CTGAATTAAAATATGATTAAATTAGCATCGTTGGGGATCCCTGGGTGGCGCAGCGGTTTGGCGCCTGCCTTTGGCCCAGGGCGCGATCCTGGAGACCCGGGATCGAATCCCACATCGGGCTCCCGGTGCATGGAGCCTGCTTCTCCCTCT
CM021997.1:1943637  : AGTCCTTGTCCTGTCAATAAATTGTTTTTGTCGGGGATCCCTGGGTGGCGCAGCGGTTTGGCGCCTGCCTTTGGCCCAGGGCGCGATCCTGGAGACCCGGGATCGAATCCCACATCGGGCTCCCGGTGCATGGAGCCTGCTTCTCCCTCT
```

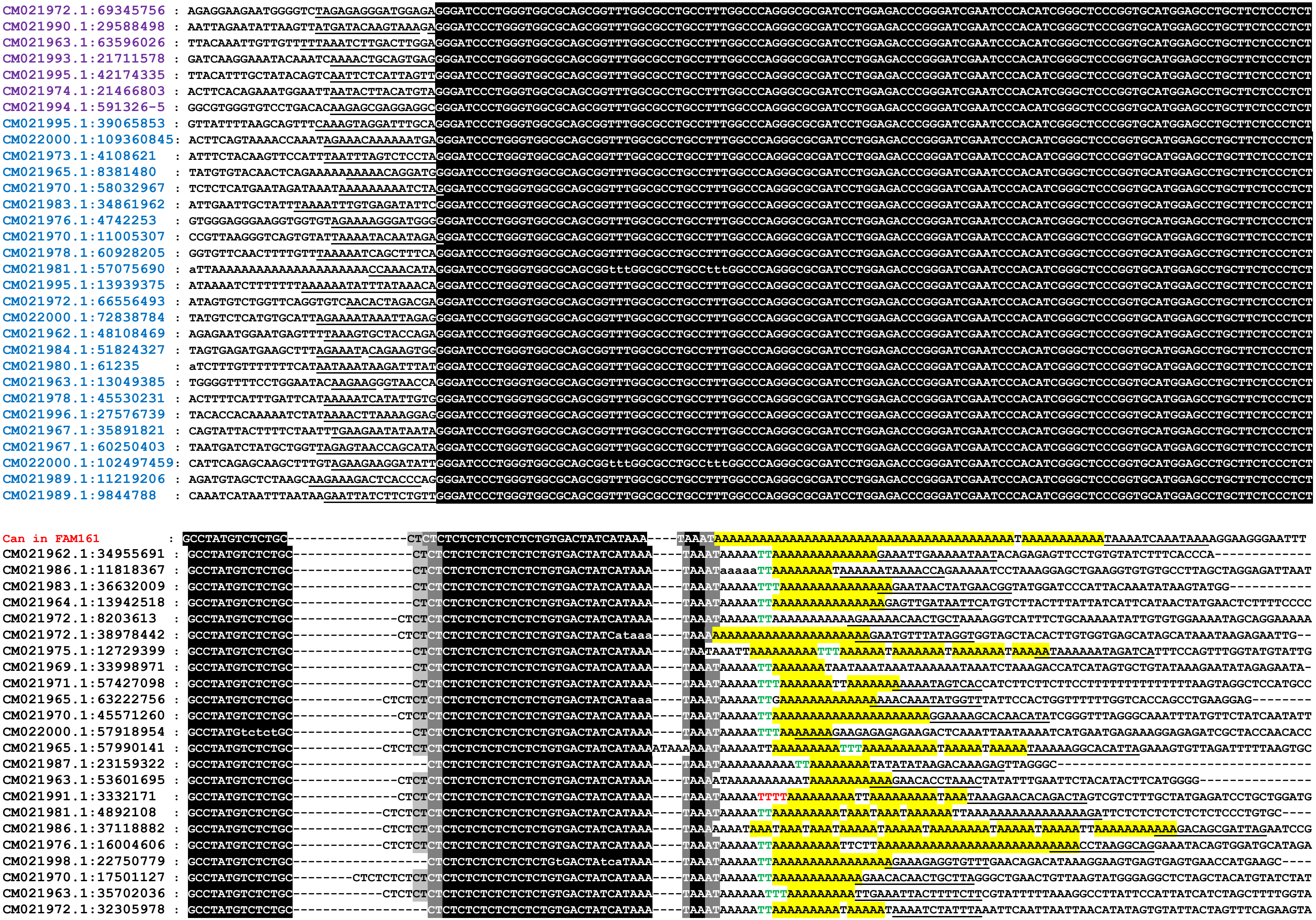

CM021972.1:69345756
CM021990.1:29588498
CM021963.1:63596026
CM021993.1:21711578
CM021995.1:42174335
CM021974.1:21466803
CM021994.1:591326-5
CM021995.1:39065853
CM022000.1:109360845
CM021973.1:4108621
CM021965.1:8381480
CM021970.1:58032967
CM021983.1:34861962
CM021976.1:4742253
CM021970.1:11005307
CM021978.1:60928205
CM021981.1:57075690
CM021995.1:13939375
CM021972.1:66556493
CM022000.1:72838784
CM021962.1:48108469
CM021984.1:51824327
CM021980.1:61235
CM021963.1:13049385
CM021978.1:45530231
CM021996.1:27576739
CM021967.1:35891821
CM021967.1:60250403
CM022000.1:102497459
CM021989.1:11219206
CM021989.1:9844788
Can in FAM161
CM021962.1:34955691
CM021986.1:11818367
CM021983.1:36632009
CM021964.1:13942518
CM021972.1:8203613
CM021972.1:38978442
CM021975.1:12729399
CM021969.1:33998971
CM021971.1:57427098
CM021965.1:63222756
CM021970.1:45571260
CM022000.1:57918954
CM021965.1:57990141
CM021987.1:23159322
CM021963.1:53601695
CM021991.1:3332171
CM021981.1:4892108
CM021986.1:37118882
CM021976.1:16004606
CM021998.1:22750779
CM021970.1:17501127
CM021963.1:35702036
CM021972.1:32305978

```
CM021983.1:53301297 : GCCTATGTCTCTGC------------CTCTCTCTCTCTCTCTCTCTCTCTGTGACTATCATAAA----TAAAAAAAAAAAAAAAAAAGAAAACATTAATTCAAAAAGATATATGTGCCCTTACATTTACTGAAGCATTATATA---------
CM021968.1:10325141 : GCCTATGTCTCTGC--------------CTCTCTCTCTCTCTCTCTCTCTGTGACTATCATAAA----TAAATAAAAATTTTAAAAAAATTTTTGTTTTTTTATTGATTTATGATAGTCACACACACACACACACACACACACACACA
CM021964.1:30661009 : GCCTATGTCTCTGC----------CTCTCTCTCTCTCTCTCTCTCTCTCTCTGTGACTATCATAAA----TAAATAAAAATTAAAAAAAAACTGCTGTCATCATTGCCTAATAAAAGCTGATTTTTAATTAGATACAATTTTTAGAA-------
CM021978.1:18216705 : GCCTATGTCTCTGC----------------CTCTCTCTCTCTCTCTCTCTGTGACTATCATAAA----TAAATAAAAATTAAAAAAAAAATTTGTGTTTAAAGATTTTATTTATTTATCTGAGAGAGACAGAGTTTTCTAACAGG-------
CM021966.1:47327703 : GCCTATGTCTCTGC--------CTCTCTCTCTCTCTCTCTCTCTCTCTCTCTGTGACTATCATAAA----TAAATAAATAAATTAATTAATTAAAAAAAAAAAGAAAGCTAAAAATCATAAATTGAGGGATCAAAAGAACAGAAACAAAC----
CM021970.1:55413363 : GCCTATGTCTCTGC------------CTCTCTCTCTCTCTCTCTCTCTCTGTGACTATCATAAA----TAAATTAAAAAAAAATTAATAATAAAAAAAAAAGCCCAAATTTAAAAAAAAAAAAAAAAAAAAAAAAGACCACAGCATTTCACC
CM021966.1:60666245 : GCCTATGTCTCTGC----------CTCTCTCTCTCTCTCTCTCTCTCTCTCTGTGACTATCATAAA----TAAATAAAAATTAAAAAAAAATAAAATAAAATAAAATTTCTAGTTAAAATGCACCAAACTCTCCCCGAACACTAAAAAAAGGG
CM021998.1:2561342  : GCCTATGTCTCTGCCTCTCTCTCTCTCTCTCTCTCTCTCTCTCTCTCTCTCTGTGACTATCATAAA----TAATAAATAAAAGAAAAAATTAAAAAAAAAAGAAACTAATGAGAATGAAGATACAACTGTTCAAAATCTTTGGGATG-------
CM021966.1:57346575 : GCCTATGTCTCTGC------------------CTCTCTCTCTCTCTCTCTGTGACTATCATAAA----TAAATAAAAATTAAAAAAAAATAATAATAAAAATAAAACAAAATCCAAATAATTAATGAGCATCCGTTTCAGAGGTTGGTAA--
CM022000.1:5548874  : GCCTATGTCTCTGC------------------CTCTCTCTCTCTCTCTCTGTGACTATCATAAA----TAAATAAAAATTAAAAAAAAATTAAATCAAAATGCATGTTTCCCTTAATTTACTATCTAATCATCACTGATGGTTTTAAAAT--
CM021987.1:24241989 : GCCTATGTCTCTGC--------CTCTCTCTCTCTCTCTCTCTCTCTCTCTCTGTGACTATCATAAA----TAAATTAAAAAAAAAAAAAAAAGAAAACCTTAACAGGCCAATAACCAGGGAGGAAATTGAAGCAGTCATCAAAAA---------
CM021989.1:1299907  : GCCTATGTCTCTGC------------------CTCTCTCTCTCTCTCTCTGTGACTATCATAAA----TAAATAAAAATTAAAAAAAATAAGTCACCAGTCTATGGAATTTTTGTCATAGCAGATTGAATAGACTTAGGTCAATGAATTAGG
CM021976.1:5736094  : GCCTATGTCTCTGC------------------CTCTCTCTCTCTCTCTCTGTGACTATCATAAA----TAAATAAAAATAAAAAAAAAAAATCAGTCATAAAAAAAAAATGAAAACGTCTGCCTTCCTTATAGGGAATCTCCGATCTGATAGA
CM021969.1:31664805 : GCCTATGTCTCTGC----------------CTCTCTCTCTCTCTCTCTCTGTGACTATCATAAA----TAAATAAAAAAAATTACAAAAAATAAAAGTAATCTCTACACCCAGCATAGGGCTTAAATTCACAACCCCCAAATCAAGAGTCAA
CM021984.1:50603247 : GCCTATGTCTCTGC----------------CTCTCTCTCTCTCTCTCTCTGTGACTATCATAAA----TAAATAAAAAAATTAAAAAAAAAATACCCAGTGCTCATTACATCACATGCCCTCCTTAATGTCCATCACCAGT-----------
CM021962.1:57612401 : GCCTATGTCTCTGC------------------CTCTCTCTCTCTCTCTCTGTGACTATCATAAA----TAAATATAAATTAAAAAAAAAAAAAGAGCAGAGTCAAAATAATGTTCATGAGTATACAGGCCTGGTCCAAG------------
CM021976.1:38824663 : GCCTATGTCTCTGC------------CTCTCTCTCTCTCTCTCTCTCTCTGTGACTATCATAAA----TAAATAAAAAAATTTAAAAAAAAATTTAAAAAAAGGATGTTGTTTTAAGCTGCTAAATTTTGTGGTGGTTTGTTATACAGCAAT
CM021983.1:612810   : GCCTATGTCTCTGC----------------CTCTCTCTCTCTCTCTCTCTGTGACTATCATAAA----TAAATAAAAATTTAAAAAAAAAAAAAAAAAAAAAAAAAAAAAAAAAGAACATGAAGGCATCTGTTTTTATGGGAAAATGAAACA
CM021984.1:7474507  : GCCTATGTCTCTGC----------------CTCTCTCTCTCTCTCTCTCTGTGACTATCATAAA----TAAATAAAAATTTAAAAAAAAAAAAAAAAAAAAGAAAACAAAATCTCAGCAAGAAACCAGAAAATCTCAGGAAAGAAATAGAAGA
CM021981.1:55525507 : GCCTATGTCTCTGC--CTCTCTCTCTCTCTCTCTCTCTCTCTCTCTCTCTCTGTGACTATCATAAA----TAAATAAAAATTAAAAAAAAAAAAAAAGAACTGAAGATGGGGTAAAAAAAAT----------------------------------
CM021972.1:27660724 : GCCTATGTCTCTGC--------------CTCTCTCTCTCTCTCTCTCTCTGTGACTATCATAAA----TAAATAAAAAAATAAAAATTAAAAAAAAAAAAAAAAAAAAAGCCAGAGGTAAGCGATACAAT---------------------
CM021968.1:44424747 : GCCTATGTCTCTGC------------------CTCTCTCTCTCTCTCTCTGTGACTATCATAAA----TAAATAAAAAAAAAAAAAAGCAAGACCCATCTATTTGCTGTCTAAAAGAGACTCATTAAAAAAAAAAAGAATATTATGAGCAAT
CM021986.1:18354655 : GCCTATGTCTCTGC------------CTCTCTCTCTCTCTCTCTCTCTCTGTGACTATCATAAA----TAAATAAAAAAATTGAAAAAAAAAAAAAAAAAAAAGAATGTTCTTTAAAAAAAAAAAAAAAAAAGTCAACACCAGGGC------
CM021980.1:17876643 : GCCTATGTCTCTGC------------CTCTCTCTCTCTCTCTCTCTCTCTGTGACTATCATaaa----TAAAAAAATAAAAATAAAAAAAAAAATAAAAAAATAAAAAAAGAGAGAAGAGTGGTTCTGTCATCAAAATGTCACTAAAAAG---
CM021980.1:41754496 : GCCTATGTCTCTGC------------CTCTCTCTCTCTCTCTCTCTCTCTGTGACTATCATAAA----TAAATAAAAATTAAAAAAAAATTAAAAAAAAAAAGAAAGGAAAACTAGTTTGGAATCATCTGTTTGCTGGGGATAACAGAGAAA
CM021962.1:7987457  : GCCTATGTCTCTGC--------CTCTCTCTCTCTCTCTCTCTCTCTCTCTCTGTGACTATCATAAA----TAAATAAAAATTTAAAAAAAAAAAAAAAAAAATACTACTCCTGCTCACAAAGTGATTGTGAACTAAACAAGCAAAAACTCTTGTA
CM021973.1:59099445 : GCCTATGTCTCTGC--------CTCTCTCTCTCTCTCTCTCTCTCTCTCTCTGTGACTATCATAAA----TAAATAAAAATTAAAAAAAAAAAAAAAGAAAGTCATATTCAGTCTAACTAAGCTAGAATATCAATATATGAACTCAAAAGA---
CM021983.1:4637355  : GCCTATGTCTCTGC--------------CTCTCTCTCTCTCTCTCTCTCTGTGACTATCATAAA----TAAATAAAAATTGAAAAAAAAAAAAAAAAGAATGTTCTTTTCAAGTGTTGTATGTGGGTTCCAGTGAATCCATTAAATA-----
CM021986.1:24336044 : GCCTATGTCTCTGC--------CTCTCTCTCTCTCTCTCTCTCTCTCTCTCTGTGACTATCAtaaa----taaATAAAAAAAAATTAAAAAATAAATAAAAAATAAAATAACAGCATAATTGGAATCTCTCAAGACACATTATATACTTGTGAT
CM021970.1:15683527 : GCCTATGTCTCTGC------------------CTCTCTCTCTCTCTCTCTGTGACTATCATAAA----TAAATAAAAAAATTAAAAAAAAATAAATAAATAAAACTGTGGTGAGTTTTCTGGGGAAGATAACAGAA---------------
CM021979.1:3742243  : GCCTATGTCTCTGC------------CTCTCTCTCTCTCTCTCTCTCTCTGTGACTATCATAAA----TAAATAAATAAATAAAATTAAAAAAAAAATTAGCATCGTTGTTTCTTTATAGCTTGCTTTATATTTCTTTTGGATAATGGTAAT
CM021997.1:1943637  : GCCTATGTCTCTGC------------CTCTCTCTCTCTCTCTCTCTCTCTGTGACTATCATAAA----TAAATAAAAATTTTTTAAAAAAAATTGTTTTTGTCCTCAGATAAAACTATATCTTAGGGTTttgtttttTTTTTTCTGTACTTC
CM021972.1:69345756 : GCCTATGTCTCTGC----------------ctctctctctctctctctctGTGACTATCATAAA----TAAAAAAAAAAAAATTAGAGAGGGATGGAGAAAACAAACATTGGGAAGTCACTCGTTATTTGTCCTTGCTGGTGGT-------
CM021990.1:29588498 : GCCTATGTCTCTGC------------------CTCTCTCTCTCTCTCTCTGTGACtatcataaa----TAAATAAATAAAAATTGAAAAAAAATGATACAAGTAAAAATTTCAAAGTTATTCATTTTTTTGTTACAATTAGGACTA------
CM021963.1:63596026 : GCCTATGTCTCTGC------------------CTCTCTCTCTCTCTCTCTGTGACTATCATAAA----TAAATAAAAAAATTTAAAAAAAAAAAAATTTAAATCTTGACTTGGAAACTTCCCCCCTTTTATCAATAGCTATAGTTCTTGCAA
CM021993.1:21711578 : GCCTATGTCTCTGC------------------CTCTCTCTCTCTCTCTCTGTGACTATCATAAA----TAAATAAAAAAAAAAAAAACAAAAAAACTGCAGTGAGATACCACTTTACACCGCGAGAATGGCTATTAATTATAAGGCAGGAAA-
CM021995.1:42174335 : GCCTATGTCTCTGC------------------CTCTCTCTCTCTCTCTCTGTGACTATCATAAA----TAAATTAAAAATTAAAAAAAAAATTCTCATTAGTTGTGGTAGTTTTGTTCTACAACGTTGCTACAAATACTGAATTAGTAAA--
CM021974.1:21466803 : GCCTATGTCTCTGC-----CTCTCTCTCTCTCCTCTCTCTCTCTCTCTCTGTGACTATCATAAA----TAAATAAAAATTTTTAAAAAATACTTACATGTAGAGGGTGTATGAATGTTTTCCAAGTGCTTCCACATTTTAACGCCAA-----
CM021994.1:591326   : GCCTATGTCTCTGC----------------CTCTCTCTCTCTCTCTCTCTGTGACTATCATAAA----TAAATAAAAATTAAAAAAAAAAAAAAAAAAGAGCGAGGAGGCACACGCACACCAGCAAGCTC----------------------
CM021995.1:39065853 : GCCTATGTCTCTGC----------------CTCTCTCTCTCTCTCTCTCTGTGACTATCATAAA----TAAATAAAAATTAAAAAAAAAAAAAAAAACAAAGTAGGATTTGCAAAAAATTAAATGATCAATCACCTGCAGGCCTATATAGAAA
CM022000.1:109360845: GCCTATGTCTCTGC----------------CTCTCTCTCTCTCTCTCTCTGTGACTATCATAAA----TAAATAAAAATTTAAAAAAAAAAAAAAAAAAAAAAAAAAAAAAAAGAAACAAAAAATGATGTGATATTTTCTTTTAGGAGGCA
CM021973.1:4108621  : GCCTATGTCTCTGC----------------CTCTCTCTCTCTCTCTCTCTGTGACTATCATAAA----TAAATAAAAATTTAAAAAAAAAAAAAAAAAAAAAAAAATAATTTAGTCTCCTAGCTGTAAGTATGTAAATAGCGTTTTAAAGG--
CM021965.1:8381480  : GCCTATGTCTCTGC----------------CTCTCTCTCTCTCTCTCTCTGTGACTATCATAAA----TAAATAAAAATTTAAAAAAAAAAAAAACAAAAAACAAAAAAACAAAAACAGGATGAATGGTTGGATGGATGGATGGA-----
CM021970.1:58032967 : GCCTATGTCTCTGC----------------CTCTCTCTCTCTCTCTCTCTGTGACTATCATAAA----TAAATAAAAATTAAAAAAAAAAATCTAAAAAAAAAAAAATAGCTAAGTCATTTATCTATTGTGATGACTCCTAGCTGGTGATAAT
CM021983.1:34861962 : GCCTATGTCTCTGC--------------CTCTCTCTCTCTCTCTCTCTCTGTGACTATCATAAA----TAAATAAAAAATTTAAAAAAAAATAAATAAATAAAAAATAAAAAAAAAATAAAATAAAATTTGTGAGATATTCAGATAA------
CM021976.1:4742253  : GCCTATGTCTCTGC--------------CTCTCTCTCTCTCTCTCTCTCTGTGACTATCATAAA----TAAATAAAAATTTAAAAAAAAAAAAAAAATAAAAAAAAAAAAAAAGAAAAGGATGGGTCACAGGTCGGGGAAGTCTAGTTAAGG
CM021970.1:11005307 : GCCTATGTCTCTGC--------------CTCTCTCTCTCTCTCTCTCTCTGTGACTATCATAAA----TAAATAAAAATTAAAAAATAAATAAATAAAAATTTAATAAAAAAAAATAAAAAATAAAAAAATAAAATACAATAGATT------
CM021978.1:60928205 : GCCTATGTCTCTGC--------------CTCTCTCTCTCTCTCTCTCTCTGTGACTATCATAAA----TAAATAAAAAAAAAATAAAATAAAATAAAATAAAATAAAAATCAGCTTTCAG---------------------------------
CM021981.1:57075690 : GCCTATGTCTCTGC--------------CTCTCTCTCTCTCTCTCTCTCTGTGACTATCATaaa----TAAATAAAAATTAAAAAAAAAAAAAAACCCAAACATAGGGACTC-----------------------------------------
CM021995.1:13939375 : GCCTATGTCTCTGC--------------CTCTCTCTCTCTCTCTCTCTCTGTGACTATCATAAA----TAAATTAAAAAAAATATTTATAAACAAAAACAATGCAATAGCAAAAACAAGTTATAAATTCTAAGTTTAATGATGAGACACAAA
CM021972.1:66556493 : GCCTATGTCTCTGC----------------CTCTCTCTCTCTCTCTCTCTGTGACTATCATAAA----TAAATAAAAATTAAAAAAAAAAAAACACTAGACGAGTATTGTTGAATAAATGTTGGAGGAG---------------------
CM022000.1:72838784 : GCCTATGTCTCTGC------------CTCTCTCTCTCTCTCTCTCTCTCTGTGACTATCATAAA----TAAATAAATAAATAAAAGAAAATAAATAGAAAATAAATTAGAAAATAAAGAGAAAATAAATGAGAATAATATTTATACAGTTG-
CM021962.1:48108469 : GCCTATGTCTCTGC------------------CTCTCTCTCTCTCTCTCTGTGACTATCATAAA----TAAATAAAAATTTAAAAAAAATAAATAAATAAAAAATAAAGTGCTACCAGAAAAAAATAGCACACTGTCAACACAATTCTTTAT
CM021984.1:51824327 : GCCTATGTCTCTGC------------------CTCTCTCTCTCTCTCTCTGTGACTATCATAAA----TAAATAAATAAATTAAAAAAAAAAAGAAATTCAGAAGTGGAAAGAAGGAATGATGTAGGGAGATGGCTAGAGGGAGGAGG-
CM021980.1:61235    : GCCTATGTCTCTGC----------CTCTCTCTCTCTCTCTCTCTCTCTCTCTGTGACTATCATAAA----TAAATAAAAAAAATAAAAAAAAATAAATAAGATTTATTTTGAAAATTGACTTAAATTAATCAGGCTCCAAGTGTTAGACATTTT
CM021963.1:13049385 : GCCTATGTCTCTGC----------------CTCTCTCTCTCTCTCTCTCTGTGACTATCATAAA----TAAATAAAAATCTAAAAAAAAAAAAAAAAAAAAGAAGAGTAACTGAACACATTAACAAATCAAGGGATGAAATAACGTAAT----
CM021978.1:45530231 : GCCTATGTCTCTGC--------------CTCTCTCTCTCTCTCTCTCTCTGTGACTATCATAAA----TAAAtAAAAATTTAAAAAAAAATCATATTGTGATAATGATTCTTGTTTTTTTATTTTATTAACTTAGGAACTGAGTTTTA----
```

```
CM021996.1:27576739 : GCCTATGTCTCTGC--------------CTCTCTCTCTCTCTCTCTCTGTGACTATCATAAA----TAAATAAAAATTAAAAAAAAAAAACTTAAAAGGAGCTACTTCATCAGTGGCTAAATTTAGTATTCTAAAATATCATTGCATTTG
CM021967.1:35891821 : GCCTATGTCTCTGC------------------CTCTCTCTCTCTCTCTCTGTGACTATCATAAA----TAAAAAAAAAAAAAAAAAAAAAAAAAAAAATGAAGAATATAATAAATAAGCTTGCCCCAGACTGTTATTTTCAATCCTCTCA
CM021967.1:60250403 : GCCTATGTCTCTGC------------------CTCTCTCTCTCTCTCTCTGTGACTATCATAAA----TAAATAAAAAAAAAAAAAAATTAGAGTAACCAGCATAGAGTGGAGATGGGGAGAACTGGACAATCTAGACACATTAAAAGCTA
CM022000.1:102497459: GCCTATGTCTCTGC------------------CTCTCTCTCTCTCTCTCTGTGACTATCATAAA----TAAATTTTAAAAAAAAGAAAGAAGGATATTATGCTTAGGAGTGATGAAGTCAGTTTCACTTACTGAGTACTCATAAGATGCaaa
CM021989.1:11219206 : GCCTATGTCTCTGC------------CTCTCTCTCTCTCTCTCTCTCTGTGACTATCATAAA----TAAATAAATAAATAAATAAATAAAAAAAAAAGAAAGACTCACCCTCTCCACCAGGAACAATAGCCTCGTCGTGGTGATTCTGGA
CM021989.1:9844788  : GCCTATGTCTCTGC------------CTCTCTCTCTCTCTCTCTCTCTGTGACTATCATAAA----TAAATAAAAATTTTTAAAAAAATGAATTATCTTCTGTTAATGTCAAAGTCTGACCCATTATTTGGCTCACAGAATAAAGCTCCT
```

H

```
Can_in_RAB3GAP : ATCCAGCAGACCTGATAAATGAGTGTACTG--------TGGGTGGCGCAGCGGTTTGGTGCCTGCCTTTGGCCCAGGGCGCGATCCTGGAGACCCGGGATCGAATCCCACGTCAGGCTCCCGGTGCATGGAGCCTGCTTC
chr11_50563233 : TTTATTTATTTATTTATTTATAGAGAGCATGGGATCCCTGGGTGGCGCAGCGGTTTGGTGCCTGCCTTTGGCCCAGGGCGCGATCCTGGAGACCCGGGATCGAATCCCACGTCAGGCTCCCGGTGCATGGAGCCTGCTTC
chr20_45149567 : CTTCCAGCAATTTATCAAAACTGGAGTGGAGGGATCCCTGGGTGGCGCAGCGGTTTGGTGCCTGCCTTTGGCCCAGGGCGCGATCCTGGAGACCCGGGATCGAATCCCACGTCAGGCTCCCGGTGCATGGAGCCTGCTTC
chr23_10559329 : AGAAATGCCAGTTTTTAAAATCTGGGGGGTGGGATCCCTGGGTGGCGCAGCGGTTTGGTGCCTGCCTTTGGCCCAGGGCGCGATCCTGGAGACCCGGGATCGAATCCCACGTCAGGCTCCCGGTGCATGGAGCCTGCTTC
chr14_6885890  : CATGACTTTGTGAATATACTAAAAATCACTGGGATCCCTGGGTGGCGCAGCGGTTTGGCGCCTGCCTTTGGCCCAGGGCGCGATCCTGGAGACCCGGGATCGAATCCCACGTCAGGCTCCCGGTGCATGGAGCCTGCTTC
chr14_6723646  : TAAATAAAATATTTTTTTAAATAAAAATAAGGGATCCCTGGGTGGCGCAGCGGTTTGGTGCCTGCCTTTGGCCCAGGGCGCGATCCTGGAGACCCGGGATCGAATCCCACGTCAGGCTCCCGGTGCATGGAGCCTGCTTC
chr23_11296985 : GAGAAATATTACATAATAATAAAAGAGACAGGGATCCCTGGGTGGCGCAGCGGTTTGGTGCCTGCCTTTGGCCCAGGGCGCGATCCTGGAGACCCGGGATCGAATCCCACGTCAGGCTCCCGGTGCATGGAGCCTGCTTC
chr37_25790079 : AATAAAATCTTTTTTAAAAATGCCCATGTAGGGATCCCTGGGTGGCGCAGCGGTTTGGTGCCTGCCTTTGGCCCAGGGCGCGATCCTGGAGACCCGGGATCGAATCCCACGTCAGGCTCCCGGTGCATGGAGCCTGCTTC
chr25_39686772 : GATGAGCTGTTATCCAAAATACACTTGGTGGGGATCCCTGGGTGGCGCAGCGGTTTGGTGCCTGCCTTTGGCCCAGGGCGCGATCCTGGAGACCCGGGATCGAATCCCACGTCAGGCTCCCGGTGCATGGAGCCTGCTTC
chr2_72065051  : AGCCATTGGAGAATTTTAAACATGGAACTTGGGATCCCTGGGTGGCGCAGCGGTTTGGTGCCTGCCTTTGGCCCAGGGCGCGATCCTGGAGACCCGGGATCGAATCCCACGTCAGGCTCCCGGTGCATGGAGCCTGCTTC
chr9_41032131  : AGGAAGTGCAATTTAAAATATGAATAGCGGGGGATCCCTGGGTGGCGCAGCGGTTTGGTGCCTGCCTTTGGCCCAGGGCGCGATCCTGGAGACCCGGGATCGAATCCCACGTCGGGCTCCCGGTGCATGGAGCCTGCTTC
chr16_10058126 : AAAAACTCTTTATTAAAAACCTGAAAAACTGGGATCCCTGGGTGGCGCAGCGGTTTGGTGCCTGCCTTTGGCCCAGGGCGCGATCCTGGAGACCCGGGATCGAATCCCACGTCAGGCTCCCGGTGCATGGAGCCTGCTTC
chr18_13883695 : CAAACAGAAACAGAATTGAAAACATGACCAGGGATCCCTGGGTGGCGCAGCGGTTTGGTGCCTGCCTTTGGCCCAGGGCGCGATCCTGGAGACCCGGGATCGAATCCCACGTCAGGCTCCCGGTGCATGGAGCCTGCTTC
chr21_4425642  : TAAAATCCATCCTTAGGATTACCCATTTGTGGGATCCCTGGGTGGCGCAGCGGTTTGGTGCCTGCCTTTGGCCCAGGGCGCGATCCTGGAGACCCGGGATCGAATCCCACGTCAGGCTCCCGGTGCATGGAGCCTGCTTC
chr24_14483734 : ATTAGTTTAAGCAAAAAAGGGTTTCTTTCGGGGATCCCTGGGTGGCGCAGCGGTTTGGTGCCTGCCTTTGGCCCAGGGCGCGATCCTGGAGACCCGGGATCGAATCCCACGTCAGGCTCCCGGTGCATGGAGCCTGCTTC
chr33_2401332  : TGCAAAACTTTCTTTAACAACACATCAAAGGGGATCCCTGGGTGGCGCAGCGGTTTGGTGCCTGCCTTTGGCCCAGGGCGCGATCCTGGAGACCCGGGATCGAATCCCACGTCAGGCTCCCGGTGCATGGAGCCTGCTTC

Can_in_RAB3GAP : TCCCTCTGCCTGTGTCTCTGCCTCTCTCTCTCTCTGTAACTATCATAAATAAATAAAAATTAAAAAAAAA--------TAAAAATATTAAAAAAAAAAAAAAAAAAAAAAAAAAAAAATGAGTGTACTGATTTGG
chr11_50563233 : TCCCTCTGCCTGTGTCTCTGCCTCTCTCTCTCTCTGTAACTATCATAAATAAATAAAAATTAAAAAAAAATAA-----TAAAAATATATTTATAGAAAGCATGTGAATGGTGGGAGGGGCAAAGAGATAGGGA
chr20_45149567 : TCCCTCTGCCTGTGTCTCTGCCTCTCTCTCTCTCTGTAACTATCATAAATAAATAAAAATTAAAAAAAAAA-------TAAAAATATTAAAAAAAAAAAAAAAAAAAAAAACTGGAGTGGAGTGTGGAAACCCT--
chr23_10559329 : TCCCTCTGCCTGTGTCTCTGCCTCTCTCTCTCTCTGTAACTATCATAAATAAATAAAAATTAAAAAAAAA--------TAAAAATATTTAAAAAAAAAAAATAAATAAAATCTGGGGGGAAGGTTTTTGGAAA-
chr14_6885890  : TCCCTCTGCCTGTGTCTCTGCCTCTCTCTCTCTCTGTAACTATCATAAATAAATAAAAATTAAAAAAAAA--------TAAAAATATTTTTAAAAAAAATAAAAAAAATCACTGAATGGTTTGCTTTAAAAGGA
chr14_6723646  : TCCCTCTGCCTGTGTCTCTGCCTCTCTCTCTCTCTGTAACTATCATAAATAAATAAAAATTAAAAAAAAAAATAAAAATAAAAATATTAAATAAAAATAAATAAAAGCCAAGAAGAGGGGAGGTGGAG-----
chr23_11296985 : TCCCTCTGCCTGTGTCTCTGCCTCTCTCTCTCTCTGTAACTATCATAAATAAATAAAAATTAAAAAAAAAAA------TAAAAATATAAAAAAAAAAATAAATAAAATAAAAGAGACAATCCACCAATGAAATA
chr37_25790079 : TCCCTCTGCCTGTGTCTCTGCCTCTCTCTCTCTCTGTAACTATCATAAATAAATAAAAATT--AAAAA-----------AAAATATTTAAAAATGCCCATGTAAAACTGTATTACTTCTGATATGGAAATTG
chr25_39686772 : TCCCTCTGCCTGTGTCTCTGCCTCTCTCTCTCTCTGTAACTATCATAAATAAATAAAAATTTAAAAAAAA-------TAAAAATATTTTAAAAAAAAAAAAAAAACAAAAAAAAACAAAATACACTTGGTGGGT
chr2_72065051  : TCCCTCTGCCTGTGTCTCTGCCTCTCTCTCTCTCTGTAACTATCATAAATAAATAAAAATTAAAAAAAAA--------TAAAAATATTAAAAAAAAATAAAATAAAAAAAATAAACATGGAACTTATGTGGTCC-
chr9_41032131  : TCCCTCTGCCTGTGTCTCTGCCTCTCTCTCTCTCTGTAACTATCATAAATAAATAAAAATTAAAAAAAAAA-------TAAAAATATTTAAAAAAAAAAATAAATAAAATATGAATAGCGGGATCCCTGGGCGG
chr16_10058126 : TCCCTCTGCCTGTGTCTCTGCCTCTCTCTCTCTCTGTAACTATCATAAATAAATAAAAATTTAAAAAAAA-------TAAAAATATTTAAAAAAAAAAAAAAAAAAAAAAAAAAAAACCTGAAAAACATACATCT
chr18_13883695 : TCCCTCTGCCTGTGTCTCTGCCTCTCTCTCTCTCTGTAACTATCATAAATAAATAAAAATTAAAAAAAAA--------TAAAAATATTTTTAAAAAAAAAAAAAAAAAAAAAAAAAAAAAGAAAACATGACCA-
chr21_4425642  : TCCCTCTGCCTGTGTCTCTGCCTCTCTCTCTCTCTGTAACTATCATAAATAAATAAAAATTAAAAAAAAAAAAA----TAAAAATATAAAAAAAAAAAAAAAAAAAAAAAAAAGGATTACCCATTTGTCATTT
chr24_14483734 : TCCCTCTGCCTGTGTCTCTGCCTCTCTCTCTCTCTGTAACTATCATAAATAAATAAAAATTAAAAAAAAAA-------TAAAAATATTAAAAAAAAAAAAAGGGTTTCTTTCTTTTTCTGCTAATGTAATTAAA
chr33_2401332  : TCCCTCTGCCTGTGTCTCTGCCTCTCTCTCTCTCTGTAACTATCATAAATAAATAAAAATTTAAAAAAA--------TAAAAATATTTAAAAAAAAAAAAAAAAACAACACATCAAAGGAGAAAAATAGACAA
```

I

```
Can in SILV               : -----------------------------------TTTCTCCTTTATTCTCCCAATGTTAGGGGAAGACCTCTGGGATCCCTGGGTGGCGCAGCGGTTTGGCGCCTGCCTT
chr1:113871832-113872406  : CCCAGAGGGGATGGCCTTGCTCTTTTCTTGCTTTGATTCTCACCCCCAAGTACTTTTCAAATGATAGCAACTGGGATCCCTGGGTGGCGCAGCGGTTTGGCGCCTGCCTT
chr22:30949619-30950193   : AATATCATAATATTGAAAGACTAAAGACTGATTTGTAATGGTTTCCTCCTCAGATTAAGGAACTAGGAAAGAGGGATCCCTGGGTGGCGCAGCGGTTTGGCGCCTGCCTT
chrX:24155953-24156527    : CCACTTGTTAACACTCTAATAAATCATTTCAGTTGTTCTCAAACTACAGTTTTTCTTTAAAACAAAGAGAGAGGGATCCCTGGGTGGCGCAGCGGTTTGGCGCCTGCCTT
chr6:64255883-64256457    : AGCATTGGCCTACATAATTAACAATTATTTCTTGGGCATAAATAAATAAAAATTTAAAAAAAAAAAAAAAAAGGGATCCCTGGGTGGCGCAGCGGTTTGGCGCCTGCCTT
chr6:20337906-20338480    : AAGTGAATCAGACAGAGAAAGACAAATATCATATGATTACACTTATAGGTGGAATCTAAAAACAAACAAACAGGGATCCCTGGGTGGCGCAGCGGTTTGGCGCCTGCCTT
chrX:56731204-56731778    : TTATAAGATGAAGGAACAAATTCCTTGTGATTTTCCAGGGTGTTCTGGGATTCAATTAAGATAAGTGATGGAGGGATCCCTGGGTGGCGCAGCGGTTTGGCGCCTGCCTT
chr10:51773037-51773611   : CAGGCAGCCCAGCAGAGGACCCACAGAAAGTAGAATAAAACTTCTGTCAAACAATATTGAAAAGGTTTCCACGGGATCCCTGGGTGGCGCAGCGGTTTGGCGCCTGCCTT
```

```
chr8:35944961-35945534     : TTGCCAACATGCTGGCACAGCACATCTTTCAAGTTGCTGGCATATCTCTGATTTAAAATACAAAGAAATACGGGGATCCCTGGGTGGCGCAGCGGTTTGGCGCCTGCCTT
chr18:17868194-17868767    : GAACAAAAGCAAATTAGTGGTGTAAAGAGGATTATAGGAAAGTGACCAACCTGTTTAAGAGAGCAACCTACTGGGATCCCTGGGTGGCGCAGCGGTTTGGCGCCTGCCTT
chr7:47465084-47465658     : TGTGTCTCATGGATAAATAAATAAAATCTTAAAAAAAAAAGAAAAGAAAACACATTTAAAGTACATAGCCAGGGGATCCCTGGGTGGCGCAGCGGTTTGGCGCCTGCCTT
chr18:4946916-4947489      : GGGAATATGTAAACGTAAAATATGAATTAGTTCATTGGTAGTCGAGTTCCCTTGCCTAGAAAGTTAAGTGTGGGGATCCCTGGGTGGCGCAGCGGTTTGGCGCCTGCCTT
chr36:18738626-18739199    : GATAATGTACTGTTGAA-TTTATAAAGGCTAAAATTGTGGATGGTTTGACCTTCTTATAAAAGTTCATGGTCGGGATCCCTGGGTGGCGCAGCGGTTTGGCGCCTGCCTT
chr32:14385689-14386262    : CTAGGACACAAAACTGAACTAGACACAATTCCTGATACCAGGGATCTCAAATTTTGGTAAAGAAGACAGATAGGGATCCCTGGGTGGCGCAGCGGTTTGGCGCCTGCCTT
chr7:32693685-32694258     : GCAACAGGCAGCTTGGTCTTAGCATGTTCCTTAAGTTCTCAAATCCGTAACTAGTTTAAAGACTAAATTATAGGGATCCCTGGGTGGCGCAGCGGTTTGGCGCCTGCCTT
chr32:10213640-10214214    : TGTAACCATTTGGATGCATCATTTCCTTCTAAGCAGATTAATACCTTGAGAATCTTAAAAATCATTCCATATGGGATCCCTGGGTGGCGCAGCGGTTTGGCGCCTGCCTT
chr27:3057190-3057763      : GCCCCTCCCCCTGCTCATGCCGTCTCTCTTTCTCTCTCTCAAATAAATTTTTTTAAAAATCTTTAAAAAATTGGGATCCCTGGGTGGCGCAGCGGTTTGGCGCCTGCCTT
chr12:35677164-35677737    : TGAGCCACCGGGGCTGCCCGAACTTCTTTCTTTGATGCCTCTAGCTAGCCTATTAAAAAATTGTACAGAATGGGGATCCCTGGGTGGCGCAGCGGTTTGGCGCCTGCCTT
chr4:27401883-27402456     : TAGATATGAATTCTTACCTTCACGGGGCTTTGAATCTGTGAGAGAGTCAGACGTTATACAAACACAACCACAGGGATCCCTGGGTGGCGCAGCGGTTTGGCGCCTGCCTT
chrX:103869600-103870173   : CAAAATGGGTGTTATTAATGTCCCCTTTGAATGGTGATGGTGAGGATTAAATGAGATGATAAACATAAACTGGGGATCCCTGGGTGGCGCAGCGGTTTGGCGCCTGCCTT
chr22:34375382-34375955    : AAAGCCAACATTGGAGATACCGTATTTATGTTTTGGGGTTCATGGGTACTAACCCATGTCCATGGATTCCAAGGGATCCCTGGGTGGCGCAGCGGTTTGGCGCCTGCCTT
chr23:26368704-26369278    : TACCAGAGGACCTTGGGAAAGTGGCTTAGCCCTCAGGGTGTCAGTGCTCACACCTATAAAACCAACACCATCGGGATCCCTGGGTGGCGCAGCGGTTTGGCGCCTGCCTT
chr15:39588710-39589283    : TTCTTGCACTACCTATTATATTATCTTCAGGGTCAAAAAGTGAACTAGTTATTTTTTTAAATGTTGATATGAGGGATCCCTGGGTGGCGCAGCGGTTTGGCGCCTGCCTT
chr7:62448662-62449235     : TAAATCTGGATGAAGGGGATATGGGAGTTATTTCTTGCAACTCTTCTCTAAGTTTGAAATTATTTCCAAATAGGGATCCCTGGGTGGCGCAGCGGTTTGGCGCCTGCCTT
chr5:83185969-83186542     : TAAATGGTGAAAGTCCTAACATATTCTCCCCTTAAAAAAAAATGTGTGCTTTATGTATATTTGAAATCCTCAGGGATCCCTGGGTGGCGCAGCGGTTTGGCGCCTGCCTT
chr28:31772028-31772602    : TTGAAAGCCCAGCTCAGTGAGACACATCTCCTCCTTAGAAGCTACTAATATTTGTTTTAAAATGAGCAGCTCGGGATCCCTGGGTGGCGCAGCGGTTTGGCGCCTGCCTT
chr2:8763851-8764425       : CCCACTATTGTCTAGGAGCTGAGCTCAATTTCTCTTTCCTGTTGCAATAGTCTTGAATAAATTTTTCCTTTAGGGATCCCTGGGTGGCGCAGCGGTTTGGCGCCTGCCTT
chr31:24350903-24351477    : AAAAAAAAAAAAAAAATGTGTCTTCTCCCAGTTGAACTGAAAAGTTAGTAAGCTACCTTGAAAGTTGAGATATGGGATCCCTGGGTGGCGCAGCGGTTTGGCGCCTGCCTT
chr9:38181488-38182061     : TCCTGAAATATGAAAATATTTGGTCATAATACCACCCTCTTTTTTTTTTTTTTTAAGATTTTATTTATTTAGGGGATCCCTGGGTGGCGCAGCGGTTTGGCGCCTGCCTT
chr19:22328635-22329209    : GCCTCAGTGTAGTGACCTATGTTCCGGAAGGTTAATATGTGCCAGCAATGAGCGTCTGTAAGGCAACAGCTTGGGATCCCTGGGTGGCGCAGCGGTTTGGCGCCTGCCTT
chr7:12912291-12912864     : GGGGTGGAGGGCATAGGCAGTGAATCTAGGACAGAGAAATTACCGTAGGTTCTTAATCAGCAAGAGACCCTGGGGATCCCTGGGTGGCGCAGCGGTTTGGCGCCTGCCTT
```

```
Can in SILV                : TGGCCCAGGGCGCGATCCTGGAGACCCGGGATCGAATCCCACGTCGGGCTCCCGGTGCATGGAGCCTGCTTCTCCCTCTGCCTGTGTCTCTGCGCCTCTCTCTCTCTCTC
chr1:113871832-113872406   : TGGCCCAGGGCGCGATCCTGGAGACCCGGGATCGAATCCCACGTCGGGCTCCCGGTGCATGGAGCCTGCTTCTCCCTCTGCCTGTGTCTCTGCGCCTCTCTCTCTCTCTC
chr22:30949619-30950193    : TGGCCCAGGGCGCGATCCTGGAGACCCGGGATCGAATCCCACGTCGGGCTCCCGGTGCATGGAGCCTGCTTCTCCCTCTGCCTGTGTCTCTGCGCCTCTCTCTCTCTCTC
chrX:24155953-24156527     : TGGCCCAGGGCGCGATCCTGGAGACCCGGGATCGAATCCCACGTCGGGCTCCCGGTGCATGGAGCCTGCTTCTCCCTCTGCCTGTGTCTCTGCGCCTCTCTCTCTCTCTC
chr6:64255883-64256457     : TGGCCCAGGGCGCGATCCTGGAGACCCGGGATCGAATCCCACGTCGGGCTCCCGGTGCATGGAGCCTGCTTCTCCCTCTGCCTGTGTCTCTGCGCCTCTCTCTCTCTCTC
chr6:20337906-20338480     : TGGCCCAGGGCGCGATCCTGGAGACCCGGGATCGAATCCCACGTCGGGCTCCCGGTGCATGGAGCCTGCTTCTCCCTCTGCCTGTGTCTCTGCGCCTCTCTCTCTCTCTC
chrX:56731204-56731778     : TGGCCCAGGGCGCGATCCTGGAGACCCGGGATCGAATCCCACGTCGGGCTCCCGGTGCATGGAGCCTGCTTCTCCCTCTGCCTGTGTCTCTGCGCCTCTCTCTCTCTCTC
chr10:51773037-51773611    : TGGCCCAGGGCGCGATCCTGGAGACCCGGGATCGAATCCCACGTCGGGCTCCCGGTGCATGGAGCCTGCTTCTCCCTCTGCCTGTGTCTCTGCGCCTCTCTCTCTCTCTC
chr8:35944961-35945534     : TGGCCCAGGGCGCGATCCTGGAGACCCGGGATCGAATCCCACGTCGGGCTCCCGGTGCATGGAGCCTGCTTCTCCCTCTGCCTGTGTCTCTGCGCCTCTCTCTCTCTCTC
chr18:17868194-17868767    : TGGCCCAGGGCGCGATCCTGGAGACCCGGGATCGAATCCCACGTCGGGCTCCCGGTGCATGGAGCCTGCTTCTCCCTCTGCCTGTGTCTCTGCGCCTCTCTCTCTCTCTC
chr7:47465084-47465658     : TGGCCCAGGGCGCGATCCTGGAGACCCGGGATCGAATCCCACGTCGGGCTCCCGGTGCATGGAGCCTGCTTCTCCCTCTGCCTGTGTCTCTGCGCCTCTCTCTCTCTCTC
chr18:4946916-4947489      : TGGCCCAGGGCGCGATCCTGGAGACCCGGGATCGAATCCCACGTCGGGCTCCCGGTGCATGGAGCCTGCTTCTCCCTCTGCCTGTGTCTCTGCGCCTCTCTCTCTCTCTC
chr36:18738626-18739199    : TGGCCCAGGGCGCGATCCTGGAGACCCGGGATCGAATCCCACGTCGGGCTCCCGGTGCATGGAGCCTGCTTCTCCCTCTGCCTGTGTCTCTGCGCCTCTCTCTCTCTCTC
chr32:14385689-14386262    : TGGCCCAGGGCGCGATCCTGGAGACCCGGGATCGAATCCCACGTCGGGCTCCCGGTGCATGGAGCCTGCTTCTCCCTCTGCCTGTGTCTCTGCGCCTCTCTCTCTCTCTC
chr7:32693685-32694258     : TGGCCCAGGGCGCGATCCTGGAGACCCGGGATCGAATCCCACGTCGGGCTCCCGGTGCATGGAGCCTGCTTCTCCCTCTGCCTGTGTCTCTGCGCCTCTCTCTCTCTCTC
chr32:10213640-10214214    : TGGCCCAGGGCGCGATCCTGGAGACCCGGGATCGAATCCCACGTCGGGCTCCCGGTGCATGGAGCCTGCTTCTCCCTCTGCCTGTGTCTCTGCGCCTCTCTCTCTCTCTC
chr27:3057190-3057763      : TGGCCCAGGGCGCGATCCTGGAGACCCGGGATCGAATCCCACGTCGGGCTCCCGGTGCATGGAGCCTGCTTCTCCCTCTGCCTGTGTCTCTGCGCCTCTCTCTCTCTCTC
chr12:35677164-35677737    : TGGCCCAGGGCGCGATCCTGGAGACCCGGGATCGAATCCCACGTCGGGCTCCCGGTGCATGGAGCCTGCTTCTCCCTCTGCCTGTGTCTCTGCGCCTCTCTCTCTCTCTC
chr4:27401883-27402456     : TGGCCCAGGGCGCGATCCTGGAGACCCGGGATCGAATCCCACGTCGGGCTCCCGGTGCATGGAGCCTGCTTCTCCCTCTGCCTGTGTCTCTGCGCCTCTCTCTCTCTCTC
chrX:103869600-103870173   : TGGCCCAGGGCGCGATCCTGGAGACCCGGGATCGAATCCCACGTCGGGCTCCCGGTGCATGGAGCCTGCTTCTCCCTCTGCCTGTGTCTCTGCGCCTCTCTCTCTCTCTC
chr22:34375382-34375955    : TGGCCCAGGGCGCGATCCTGGAGACCCGGGATCGAATCCCACGTCGGGCTCCCGGTGCATGGAGCCTGCTTCTCCCTCTGCCTGTGTCTCTGCGCCTCTCTCTCTCTCTC
chr23:26368704-26369278    : TGGCCCAGGGCGCGATCCTGGAGACCCGGGATCGAATCCCACGTCGGGCTCCCGGTGCATGGAGCCTGCTTCTCCCTCTGCCTGTGTCTCTGCGCCTCTCTCTCTCTCTC
chr15:39588710-39589283    : TGGCCCAGGGCGCGATCCTGGAGACCCGGGATCGAATCCCACGTCGGGCTCCCGGTGCATGGAGCCTGCTTCTCCCTCTGCCTGTGTCTCTGCGCCTCTCTCTCTCTCTC
chr7:62448662-62449235     : TGGCCCAGGGCGCGATCCTGGAGACCCGGGATCGAATCCCACGTCGGGCTCCCGGTGCATGGAGCCTGCTTCTCCCTCTGCCTGTGTCTCTGCGCCTCTCTCTCTCTCTC
chr5:83185969-83186542     : TGGCCCAGGGCGCGATCCTGGAGACCCGGGATCGAATCCCACGTCGGGCTCCCGGTGCATGGAGCCTGCTTCTCCCTCTGCCTGTGTCTCTGCGCCTCTCTCTCTCTCTC
chr28:31772028-31772602    : TGGCCCAGGGCGCGATCCTGGAGACCCGGGATCGAATCCCACGTCGGGCTCCCGGTGCATGGAGCCTGCTTCTCCCTCTGCCTGTGTCTCTGCGCCTCTCTCTCTCTCTC
chr2:8763851-8764425       : TGGCCCAGGGCGCGATCCTGGAGACCCGGGATCGAATCCCACGTCGGGCTCCCGGTGCATGGAGCCTGCTTCTCCCTCTGCCTGTGTCTCTGCGCCTCTCTCTCTCTCTC
chr31:24350903-24351477    : TGGCCCAGGGCGCGATCCTGGAGACCCGGGATCGAATCCCACGTCGGGCTCCCGGTGCATGGAGCCTGCTTCTCCCTCTGCCTGTGTCTCTGCGCCTCTCTCTCTCTCTC
chr9:38181488-38182061     : TGGCCCAGGGCGCGATCCTGGAGACCCGGGATCGAATCCCACGTCGGGCTCCCGGTGCATGGAGCCTGCTTCTCCCTCTGCCTGTGTCTCTGCGCCTCTCTCTCTCTCTC
chr19:22328635-22329209    : TGGCCCAGGGCGCGATCCTGGAGACCCGGGATCGAATCCCACGTCGGGCTCCCGGTGCATGGAGCCTGCTTCTCCCTCTGCCTGTGTCTCTGCGCCTCTCTCTCTCTCTC
chr7:12912291-12912864     : TGGCCCAGGGCGCGATCCTGGAGACCCGGGATCGAATCCCACGTCGGGCTCCCGGTGCATGGAGCCTGCTTCTCCCTCTGCCTGTGTCTCTGCGCCTCTCTCTCTCTCTC
```

```
Can in SILV                : TGTGACTATCATAAATAAATAAAAATTT(A)100TAGGCGAAGACTTCTGAAGCAGGGCTCAGCTCTCCCCCTTCCCCAG--------------------
chr1:113871832-113872406 : TGTGACTATCATAAATAAATAAAAATTTAAAAAAAATTATTCAAATGATAGCAACTGGTCCTAGTGACTGAACATGTGTTAGGGGACTCTGTGTAGAACT
chr22:30949619-30950193  : TGTGACTATCATAAATAAATAAAAATTTTAAAAAAAAATTTTAAAAAAAAATAAAAAAAAAAGGAACTAGGAAAGATGCAACTTTCATCAATTCAGTATT
chrX:24155953-24156527   : TGTGACTATCATAAATAAATAAAAATTTAAAAAAAAAATAAATAAATAAAACAAAGAGAGAAATATTACTAAAACACCTTAAAAAGTGGTCTACAGAATT
chr6:64255883-64256457   : TGTGACTATCATAAATAAATAAAAATTTAAAAAAAAAATTATTTCTTATTTGGCATCAAATTCTAACAGAAAATTTATCTAAATACATGGAGCATTGGCC
chr6:20337906-20338480   : TGTGACTATCATAAATAAATAAAAATTTAAAAATAAATAAATAAATAAATAAATAAATAAAAATAAAAACAAACAAACAATAAATACAAACAGATTCATA
chrX:56731204-56731778   : TGTGACTATCATAAATAAATAAAAATTTTAAAAAAAAATTTAAGATAAGTGATGGATTACTGAAAAATACTACTTGGTTCTCTATTTAACCAAAATCATG
chr10:51773037-51773611  : TGTGACTATCATAAATAAATAAAAATTTAAAAAAAAAATTTTTTTTAAAAAAAAAGAAAAGGTTTCCACATAAATGATAGTGATTTATAATGGCCTAAGG
chr8:35944961-35945534   : TGTGACTATCATAAATAAATAAAAATTAAAAAAATAAATAAATAAAATACAAAGAAATAAATAAATAAAATAAAATAAAATACAAAGAAATTTGGGATGC
chr18:17868194-17868767  : TGTGACTATCATAAATAAATAAAAATTAAAAAAAAAAAAAAAAAAGAGAACAACCTACTTGTAAGCAATCATTTGAAGTAAGCATTCATTATTCACCCAG
chr7:47465084-47465658   : TGTGACTATCATAAATAAATAAAAATTTAAAAATATATTAAAAAAAAAATAAAGTACATAGCCAGGCCCTGGCACATAGTGATCACTCAGGAAATTTTAG
chr18:4946916-4947489    : TGTGACTATCATAAATAAATAAAAATTAAAAAAAAAAAAAAGTTAAGTGCTATCTTCCTCAATTGCCATGCAGGTAAATAAATTTAGAATGAATTATTTTG
chr36:18738626-18739199  : TGTGACTATCATAAATAAATAAAAATTAAAAAAAAAAAAAAGTTCATGGTCTTCTTGGCTTTAACATTTCATTAAGTTAAAAATAAAATCTTAAACACTTA
chr32:14385689-14386262  : TGTGACTATCATAAATAAATAAAAATTAAAAAAAAAGAAGACAGATAAATAAAATTCCCAGGTAGCTCCAAAGAAGAGTTAAAGAAATTAGTTCCTATTTG
chr7:32693685-32694258   : TGTGACTATCATAAATAAATAAAAATTAAAAAAAAAATTTTTTTAAAAGACTAAATTATATAGCATATATAAAGTGCTTAGCACAATGCCTCACACATGG
chr32:10213640-10214214  : TGTGACTATCATAAATAAATAAAAATTTAAAAAAATTTTTAAAAAAATCATTCCATATATAAACAAGTGTCTTTTATCTGGGCTTAAATTTTATTTTTCA
chr27:3057190-3057763    : TGTGACTATCATAAATAAATAAAAATTAAAAAAAATCTTTAAAAAATTTAAATGCTTTAGGATATACTTAAAGCAAGTTTAGTTATCTTATCATTATGCA
chr12:35677164-35677737  : TGTGACTATCATAAATAAATAAAAATTAAAAAAAAATTGTACAGAATGATCTCTTTACTCTCAATAGGTTTACATGCAAAAATATCCAAACAAATGTCAT
chr4:27401883-27402456   : TGTGACTATCATAAATAAATAAAAATTAAAAAAAAAACACAACCACAAAAATGAGTATTATGAGAGAAAAATAATTGAGTGTCCTCAGAGAAAATATGGG
chrX:103869600-103870173 : TGTGACTATCATAAATAAATAAAAATTAAAAAAAAATAAACATAAACTGTTCTATTGTGATACCTGGTACAGAATTGACCACGAATAGTGGTACTTGCTG
chr22:34375382-34375955  : TGTGACTATCATAAATAAATAAAAATTAAAAAAAAAAAAAGAAATTATGCCTTGTGACTACTCTGGCTGCTCAGAAAATTTTATTCATCATTTACAAAAT
chr23:26368704-26369278  : TGTGACTATCATAAATAAATAAAAATTTAAAAAAAAAAAAAAAAAAAAAAAAAAAACCAACACCATCAATACGTATCTCATATGGGTTTTGTGAGCTTTA
chr15:39588710-39589283  : TGTGACTATCATAAATAAATAAAAATTAAAAAAAAAAAATTAAAAAAAAAATGTTGATATGAGCTAGGTCAAGAAATACACTTTATGTTACAGTCTGTTAC
chr7:62448662-62449235   : TGTGACTATCATAAATAAATAAAAATTAAAAAAAAAAAAAAAAAAGAAATTATTTCCAAATAAAAAGTTAAAAATGTAAGTAAATTCATTTTACTCCTCTG
chr5:83185969-83186542   : TGTGACTATCATAAATAAATAAAAATTAAAAAAAAAAAAAAAAAAGAAATCCTCAGCTGGGTCAGGGACTGTTATGCATAAACAGGACTAAAATCCTTGCC
chr28:31772028-31772602  : TGTGACTATCATAAATAAATAAAAATTTAAAAAAATAAAAAAATAAAATGAGCAGCTCCTACCAGCACCATGACACTTTTTCATCACCAATGAGTTTAGG
chr2:8763851-8764425     : TGTGACTATCATAAATAAATAAAAATTTAAAAAAAAAAAAAAAAAAAAAAAAATATAAGCACTGTTGGGGCTACTGGGTGACTCAGCTGGTTGAGTGTTC
chr31:24350903-24351477  : TGTGACTATCATAAATAAATAAAAATTTAAAAAAAAAAAAAAAAAAAGAAAGTTGAGATATTATTTTATGTACATTTTACAGAAATTATCAACAATAATC
chr9:38181488-38182061   : TGTGACTATCATAAATAAATAAAAATTAAAAAAAAAAATTGAAAAAAAAAAAAAAAAGATTTTATTTATTTACTCATGAGAGACACATGGAGAGAGAGAG
chr19:22328635-22329209  : TGTGACTATCATAAATAAATAAAAATTTAAAAAAAAAAAAAAATGTAAGGCAACAGCTTGAATCTGGGCAGGCCATGCATTGTT-CCAGCTCCTGAAATG
chr7:12912291-12912864   : TGTGACTATCATAAATAAATAAAAATTAAAAAAAAAAAAAAAAAAGAGACCCAGGTACGGGTTATGCTAGACTAAAATTGACACTTACAACAGCCAAAAAA
```

Figure S4. Can_b2 insertions (**A**-**I**) into genes that induced their mutations in dogs. The upper sequence in each alignment is the insertion (see Table 3 for references); sequences below are the most similar Can_b2 copies. Sequence names are given in blue if present in the German Shepherd alone; violet, if present in the German Shepherd and Great Dane or Boxer; and black, if present in all three breeds. The names of the most probable ancestral copies are underlined. The terminators and their rudiments are marked red and green, respectively; poly(A) tails are marked yellow; TSDs are underlined.
