# Supplementary material for: Tail Wags Dog’s SINE: Retropositional Mechanisms of Can SINE Depend on Its A-Tail Structure
Source: Biology (Basel). 2022 Sep 26;11(10):1403. doi: 10.3390/biology11101403 (PMC9599045; doi:10.3390/biology11101403)
Supplement: Supplementary file 1 [file biology-11-01403-s001.zip › Figure S5.pdf]

A

```
sdb_chr4_18735131  : TTTTTTAAAGGCATATGCAAGGCTGATTAGAAAGATTTTTCTTGGGATCCCTGGGTGGCGCAGCGGTTTAGCGCCTGCCTTTGGCCCAGGGCGCGATCCTGGAGACCCGGGATCGAATCCCACATCGGGCTCCCGGTGCA
sb_chr25_22335108  : ATTAAAGCAACAGTCTGACTTCTAATTAAAACTCAGAAGAGGGGGATCCCTGGGTGGCGCAGCGGTTTAGCGCCTGCCTTTGGCCCAGGGCACGATCCTGGAGACCCGGGATCGAATCCCACATCGGGCTCCCGGTGCA
sd_chr11_43885208  : TCTATGGTTTGAGATTTTTGTTCTTACGATTTCAAAGATTCTTGGGATCCCTGGGTGGCGCAGCGGTTTAGCGCCTGCCTTTGGCCCAGGGCGCGATCCTGGAGACCCGGGATCGAATCCCACATCGGGCTCCCGGTGCA
s_chr2_73397586    : GGACGTGGAGCGTCTGAGAGTTAGAAGGAAGGCAAAACTGAGCGGGATCCCTGGGTGGCGCAGCGGTTTGGCGCCTGCCTTTGGCCCAGGGCGCGATCCTGGAGACCCGGGATCGAATCCCACATCGGGCTCCCGGTGCA
s_chrd_21494389    : CTCTCTGTCTATCATGAATAAATAAATAAATCTTAAAAAAATCGGGATCCCTGGGTGGCGCAGCGGTTTAGCGCCTGCCTTTGGCCCAGGGCGCGATCCTGGAGACCCGGGATCGAATCCCACATCGGGCTCCCGGTGCA
s_chr10_28939323   : CAGTGACCCCAAGCAAGATAAACACAAAGAAAACTATACTCCAGGGATCCCTGGGTGGCGCAGCGGTTTAGCGCCTGCCTTTGGCCCAGGGCGCGATCCTGGAGACCCGGGATCGAATCCCACATCGGGCTCCCGGTGCA
s_chr12_1335528    : CTGTAGTATAATTATGAGAAAATATTAGATAAACCTAAATTGAGGGATCCCTGGGTGGCGCAGCGGTTTAGCGCCTGCCTTTGGCCCAGGGCGCGATCCTGGAGACCCGGGATCGAATCCCACATCGGGCTCCCGGTGCG
s_chr21_25977316   : AAAACAATCTGACAAAAAGACATATCAAGTTCTTGGAAAGGAAGGGATCCCTGGGTGGCGCAGCGGTTTGGCGCCTGCCTTTGGCCCAGGGCGCGATCCTGGAGACCCGGGATCGAATCCCACATCGGGCTCCCGGTGCA
s_chr21_28612163   : GCATCATACTAATTAATCAATTAACTATTAAATTATATATATAGGGATCCCTGGGTGGCGCAGCGGTTTAGCGCCTGCCTTTGGCCCAGGGCGCGATCCTGGAGACCCGGGATCGAATCCCACATCGGGCTCCCGGTGCA

sdb_chr4_18735131  : TGGAGCCTGTTTCTCCCTCTGCCTGTGTCTCTGCCTCTCTCGCTCTCTCTGTGTGACTATCATAAATAAATAAAAATTTTTTTAAAAAAAAGAAAGATTTTTCTTGTTTCTTTTTTGAAACATAGCATATGTTTTATGTG
sb_chr25_22335108  : TGGAGCCTGTTTCTCCCTCTGCCTGTGTCTCTGCCTCTCTCGCTCTCTCTGTGTGACTATCATAAATAAATAAAAATTTAAAAAAAAAAAAAAAAAAAAAAAAAAAAAAAAAAACTCAGAAGAGGGCAGCCCAGGTGGCCC
sd_chr11_43885208  : TGGAGCCTGTTTCTCCCTCTGCCTGTGTCTCTGCCTCTCTCGCTCTCTCTGTGTGACTATCATAAATAAATAAAAATTAAAAAAAAAAAAAAGATTCTTATCTGGGAACAAGTTCATTGAGATGCCTCACTCTTTTCCTT
s_chr2_73397586    : TGGAGCCTGTTTCTCCCTCTGCCTGTGTCTCTGCCTCTCTCGCTCTCTCTGTGTGACTATCATAAATAAATAAAAATTAAAAAAAAAAAAAAAAAAAAAAAAAAAAAACTGAGCACGAATCCCACTGATAGTGAAAATGAG
s_chrd_21494389    : TGGAGCCTGTTTCTCCCTCTGCCTGTGTCTCTGCCTCTCTCGCTCTCTCTGTGTGACTATCATAAATAAATAAAAATTAAAAAAAAAAAAAAAATCTTAAAAAAATAAAAATAAAAATAAAAAAGTTTGTAATTGTGCAT
s_chr10_28939323   : TGGAGCCTGTTTCTCCCTCTGCCTGTGTCTCTGCCTCTCTCGCTCTCTCTGTGTGACTATCATAAATAAATAAAAATTAAAAAAAAAAAAAACTATACTCCAGCATATTACATAAATGCTGAAAACCAAAGATAAACAAA
s_chr12_1335528    : TGGAGCCTGTTTCTCCCTCTGCCTGTGTCTCTGCCTCTCTCGCTCTCTCTGTGTGACTATCATAAATAAATAAAAATTTAAAAAAAAAAAAAAAAAAAAAAAAAAAAAAAAAAACCTAAATTGAGGGATGGTCTCTAAAA
s_chr21_25977316   : TGGAGCCTGTTTCTCCCTCTGCCTGTGTCTCTGCCTCTCTCGCTCTCTCTGTGTGACTATCATAAATAAATAAAAATTTAAAAAAAAAAAAAAAAAAAAAAAAAAAAAAAAAAAAAAAAAAAAAAGTTCTTGGAAAGGATGA
s_chr21_28612163   : TGGAGCCTGTTTCTCCCTCTGCCTGTGTCTCTGCCTCTCTCGCTCTCTCTGTGTGACTATCATAAATAAATAAAAATTAAAAAAAAAAAAAAAAAAAAAAATAAATTATATATATATACATATATAATGAAAATATACAG
```

B

```
sdb_chr13_10329919: AAATCTACAACAAAAGGAAAAAAACTACTAATACTTGTTTTTAAAACTCATGAGTGGGATCCCTGGGTGGTGCAGCGGTTTAGCGCCTGCCTTTGGCCCAGGGCGGGATCCTGGAGACCCGGGATCGAATCCCACATCGG
sdb_chr28_15881633: TTTTAAAATACATTTTTAAAACTATTTCATGTTTGCCTTTAGAATCATGAATGGTGGGATCCCTGGGTGGTGCAGCGGTTTAGCACCTGCCTTTGGCCCAGGGCGGGATCCTGGAGACCCGGGATCGAATCCCACATCGG
sd_chr8_30038422  : TGTCTTTTTCTTTGCTTTAGGTTCCATTAAAATGAGGTTAAAAATGTGACATATTGGGATCCCTGGGTGGTGCAGCGGTTTAGCGCCTGCCTTTGGCCCAGGGCGGGATCCTGGAGACCCGAGATCGAATCCCACATCGG
s_chr10_45100520  : TATAACTTTGAAAATGATAAATAAAAAATTTAAAAAAAAAAAAAAAAAAAAGAAAGGGATCCCTGGGTGGTGCAGCGGTTTAGCGCCTGCCTTTGGCCCAGGGCGGGATCCTGGAGACCCGGGATCGAATCCCACATCGG
s_chr22_48619613  : GTAAAGCAGGAGAAGGATGAACTTGTGGGATGTGGGGAGAAGAAAGCACCATGAGGGGATCCCTGGGTGGTGCAGCAGTTTAGCGCCTGCCTTTGGCCCAGGGCGGGATCCTGGAGACCCGGGATCGAATCCCACATCGG
s_chr27_15328787  : GTGCTCCTCAATTTATTTCTCCTGGAACATAATTTTTAAACGGTTGAAAAAAATAGGGATCCCTGGGTGGTGCAGCGGTTTAGCGCCTGCCTTTGGCCCAGGGCGGGATCCTGGAGACCCGGGATCGAATCCCACATCGG

sdb_chr13_10329919: GCTCCTGGTGCATGGAGCCTGCTTCTCCCTCTGCCTCTGTCTCTGCCTCTCTCTCTCTCTCTCTGTGTGCCTATCATAAATAAATAAAAAAATTTTTTTAAACTCATGAGTTATAATAATGCAAAACAAATTTGTCATTTT
sdb_chr28_15881633: GCTCCTGGTGCATGGAGCCTGCTTCTCCCTCTGCCTCTGTCTCTGCCTCTCTCTCTCTCTCTCTGTG--CCTATCATAAATAAATAAAAAAAATTTTTTTTAAAAAGAATCATGAATGGTGGTTCTGGACTTCCTGCTTGT
sd_chr8_30038422  : GCTCCTGGTGCATGGAGCCTGCTTCTCCCTCTGCCTCTGTCTCTGCCTCTCTCTCTCTCTCTCTGTGTGCCTATCATAAATAAATAAAAAAATTTAAAAAAAAAATGTGACATATTTTGTTTACCTGCAGGAGAAATTTGT
s_chr10_45100520  : GCTCCTGGTGCATGGAGCCTGCTTCTCCCTCTGCCTCTGTCTCTGCCTCTCTCTCTCTCTCTCTGTGTGCCTATCATAAATAAATAAAAAAATTTAAAAAAAAAAAAAAAAAAAAAAAGAAAAAGACTGTCACACTGCTTC
s_chr22_48619613  : GCTCCTGGTGCATGGAGCCTGCTTCTCCCTCTGCCTCTGTCTCTGCCTCTCTCTCTCTCTCTCTGTGTGCCTATCATAAATAAATAAAAAAATTTAAAAAAAAAAAAAAAAAAAGAAAGCACCATGAGGCAGGAAAAGGAA
s_chr27_15328787  : GCTCCTGGTGCATGGAGCCTGCTTCTCCCTCTGCCTCTGTCTCTGCCTCTCTCTCTCTCTCTCTGTGTGCCTATCATAAATAAATAAAA--TTTAAAAAAAATAAAATAAAAAAAAAAAGAAAAAAATAATAGCAAATAC
```

C

```
sdb_chr32_29446401: AGTTAGGAACTGGCATTTAAATAACCACTCAGGGATCCCTGGGTGGCGCAGCGGTTTGGCCCCTGCCTTTGGCCCAGGGCGCGATCCTGGAGACCCGGGATCGAATCCCACGTCGGGCTCCCGGTGCATGGAGCCTGCTT
sdb_chr21_35137886: GCTAGTTTCCAAGATCTATAAAGAACTTATGGGGATCCCTGGGTGGCGCAGCGGTTTGGCGCCTGCCTTTGGCCCAGGGCGCGATCCTGGAGACCCGGGATCGAATCCCACGTCGGGCTCCCGGTGCATGGAGCCTGCTT
sb_chr5_4150859.  : TTTCTATTTTTTATTTAAATTCAGTTAGCCCGGGATCCCTGGGTGGCGCAGCGGTTTGGCGCCTGCCTTTGGCCCAGGGCGCGATCCTGGAGACCCGGGATCGAATCCCACGTCGGGCTCCCGGTGCATGGAGCCTGCTT
sb_chr9_28986670. : TATGATTATGAGAATAAAATATAAAGGTGGGGGGATCCCTGGGTGGCGCAGCGGTTTGGCGCCTGCCTTTGGCCCAGGGCGCGATCCTGGAGACCCGGGATCGAATCCCACGTCGGGCTCCCGGTGCATGGAGCCTGCTT
s_chr10_63108935  : AGCAGCATGGTGAAATGGAAAAGACCCAGGAGGGATCCCTGGGTGGCGCAGCGGTTTGGCGCCTGCCTTTGGCCCAGGGTGCGATCCTGGAGACCCGGGATCGAATCCCACGTCGGGCTCCCGGTGCATGGAGCCTGCTT
s_chr19_8079380.  : AAATGGAGTCAAATTAACAATATATCTACTAGGGATCCCTGGGTGGCGCAGCGGTTTGGCGCCTGCCTTTGGCCCAGGGCGCGATCCTGGAGACCCGGGATCGAATCCCACGTCGGGCTCCCGGTGCATGGAGCCTGCTT
s_chr38_2841348.  : AGGTAAGTACCATAAAGAAAATCATAGGATGGGGATCCCTGGGTGGCGCAGCGGTTTGGCGCCTGCCTTTGGCCCAGGGCGCAATCCTGGAGACCCGGGATCGAATCCCACGTCGGGCTCCCGGTGCATGGAGCCTGCTT
d_chr3_75728882.  : CCTACAAATTAATTAAAATTTCAGTGAAATAGGGATCCCTGGGTGGCGCAGCGGTTTGGCGCCTGCCTTTGGCCCAGGGCGCGATCCTGGAGACCCGGGATCGAATCCCACGTCGGGCTCCCGGTGCATGGAGCCTGCTT
d_chrs_80918532.  : AAGTTAAATTACCTTTCAAAAGTTATGGTGAGGGATCCCTGGGTGGCGCAGCGGTTTGGCGCCTGCCTTTGGCC-AGGGCGCGATCCTGGAGACCCGGGATCGAATCCCACGTCGGGCTCCCGGTGCATGGAGCCTGCTT
```

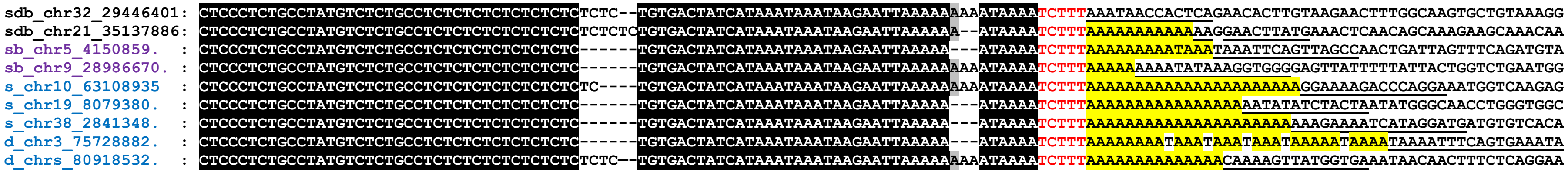
sdb_chr32_29446401:
sdb_chr21_35137886:
sb_chr5_4150859.
sb_chr9_28986670.
s_chr10_63108935
s_chr19_8079380.
s_chr38_2841348.
d_chr3_75728882.
d_chrs_80918532.

D

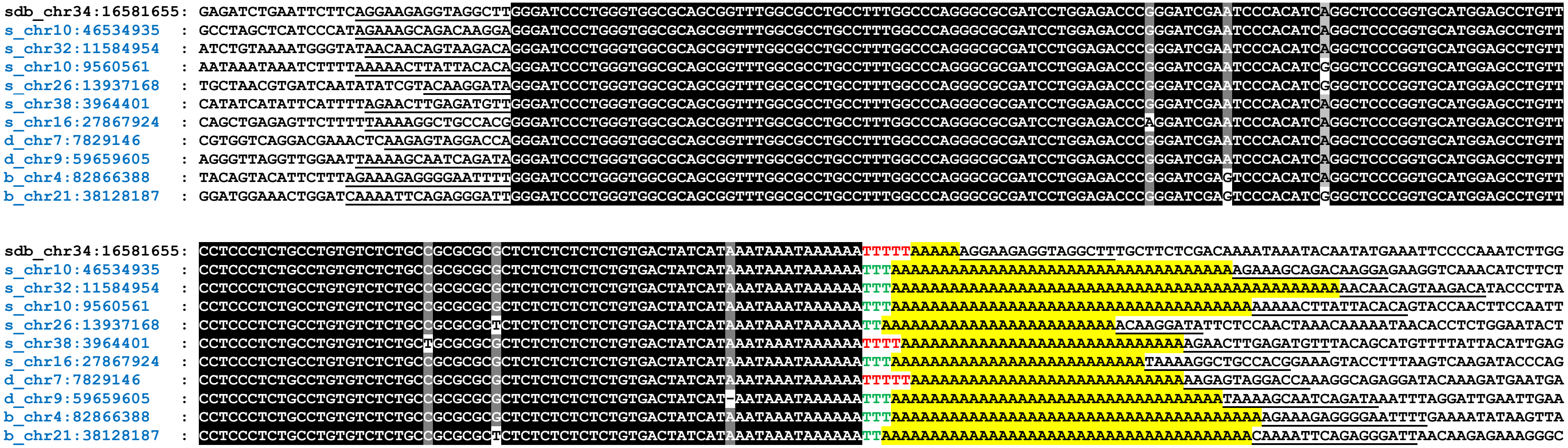
sdb_chr34:16581655:
s_chr10:46534935
s_chr32:11584954
s_chr10:9560561
s_chr26:13937168
s_chr38:3964401
s_chr16:27867924
d_chr7:7829146
d_chr9:59659605
b_chr4:82866388
b_chr21:38128187

E

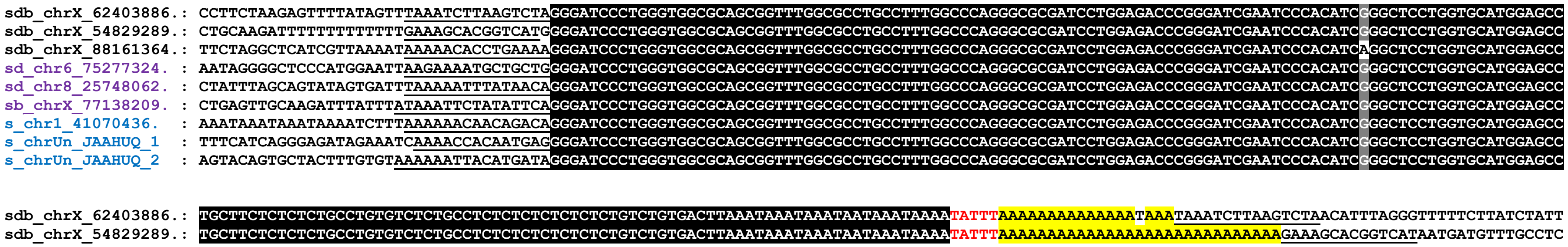
sdb_chrX_62403886.:
sdb_chrX_54829289.:
sdb_chrX_88161364.:
sd_chr6_75277324.
sd_chr8_25748062.
sb_chrX_77138209.
s_chr1_41070436.
s_chrUn_JAAHUQ_1
s_chrUn_JAAHUQ_2

```
sdb_chrX_88161364.: TGCTTCTCTCTCTGCCTGTGTCTCTGCCTCTCTCTCTCTCTCTGTCTGTGACTTAAATAAATAAATAATAAATAAAATATTTAAAAAAAAACACCTGAAAAAAATAAAATAAAATAAAAACACCTGGAGCATTATGTAAT
sd_chr6_75277324. : TGCTTCTCTCTCTGCCTGTGTCTCTGCCTCTCTCTCTCTCTCTGTCTGTGACTTAAATAAATAAATAATAAATAAAATATAAAAAAAAAAAAAAAAAAAAAAAAAAGAAAATGCTGCTGATGGGCAGCCCGGGTGGCTCAG
sd_chr8_25748062. : TGCTTCTCTCTCTGCCTGTGTCTCTGCCTCTCTCTCTCTCTCTGTCTGTGACTTAAATAAATAAATAATAAATAAAATATTTAAAAAAAAAATAAAAAAAAAATTAAAAAAAATAAAAATTTATAACATAAGTATATTTT
sb_chrX_77138209. : TGCTTCTCTCTCTGCCTGTGTCTCTGCCTCTCTCTCTCTCTCTGTCTGTGACTTAAATAAATAAATAATAAATAAAATATTTAAAAAAAAATAAATAAATAAATTCTATATTCAATTTCTTTGATATATATGTTATACAC
s_chr1_41070436.  : TGCTTCTCTCTCTGCCTGTGTCTCTGCCTCTCTCTCTCTCTCTGTCTGTGACTTAAATAAATAAATAATAAATAAAATATTTAAAAAAAAAAAAAAAAAAAAAAAAAAAAAACAACAGACAAAGCCTGCTGCTTTGTCTGCAT
s_chrUn_JAAHUQ_1  : TGCTTCTCTCTCTGCCTGTGTCTCTGCCTCTCTCTCTCTCTCTGTCTGTGACTTAAATAAATAAATAATAAATAAAATATTTAAAAAAAAAAAAAAAAAAACCACAATGAGATACCACCTCACACCAGTGAAAATGGGGG
s_chrUn_JAAHUQ_2  : TGCTTCTCTCTCTGCCTGTGTCTCTGCCTCTCTCTCTCTCTCTGTCTGTGACTTAAATAAATAAATAATAAATAAAATATTTAAAAAAAAAAAAAAAAAAAAAAAAAAAAAAAAAAAAAAATTACATGATAAAGATGACTTT
```

F

```
sdb_chr27_46295813: TGGATGTGGGCGCCATCTCTTTGAAATGCTGTCATCAGGGATCCCTGGGTGGCGCAGCGGTTTGGCGCCTGCCTTTGGCCCAGGGCGCGATCCTGGAGACCCGGGATCGAGTCCCACGTCGGGCTCCCGGTGCATGGAGC
sdb_chr9_1474920  : CATTGGGCATAGAGCTCACTTAATAATAATAATAACAGGGATCCCTGGGTGGCGCAGCGGTTTGGCGCCTGCCTTTGGCCCAGGGCGCGATCCTGGAGACCCGGGATCGAATCCCACGTCGGGCTCCCGGTGCATGGAGC
sdb_chr23_22995549: TTCATAATATATTGCCTTTTAAGATTTTTAAAAAACCGGGATCCCTGGGTGGCGCAGCGGTTTGGCGCCTGCCTTTGGCCCAGGGCGCGATCCTGGAGACCCGGGATCGAATCCCACGTCGGGCTCCCGGTGCATGGAGC
sdb_chr12_11820650: ATTCAGAGAACCTACAAGTTTAAAATGCCAGTTAATAGGGATCCCTGGGTGGCGCAGCGGTTTGGCGCCTGCCTTTGGCCCAGGGCGCGATCCTGGAGACCCGGGATCGAATCCCACGTCGGGCTCCCGGTGCATGGAGC
sdb_chr1_99860703 : CCCAAAACAAAGATTTTTAAATAAAAAAGTAAAAATAGGGATCCCTGGGTGGCGCAGCGGTTTGGCGCCTGCCTTTGGCCCAGGGCGCGATCCTGGAGACCCGGGATCGAATCCCACGTCGGGCTCCCGGTGCATGGAGC
sdb_chrX_117917331: AATAAATAAAATCTTTTTTTAAAAATACAATGAAATAGGGATCCCTGGGTGGCGCAGCGGTTTGGCGCCTGCCTTTGGCCCAGGGCGCGATCCTGGAGACCCGGGATCGAATCCCACGTCGGGCTCCCGGTGCATGGAGC
sd_chr16_25666060 : CCTGCTTGTCAGTCTGGAAAAGAGATTAGGCTTTTCGGGGATCCCTGGGTGGCGCAGCGGTTTGGCGCCTGCCTTTGGCCCAGGGCGCGATCCTGGAGACCCGGGATCGAATCCCACGTCGGGCTCCCGGTGCATGGAGC
db_chr17_52597474 : CCTTTTCTTCTAAAGCCCTTAAAAGCTTTAACTTCTAGGGATCCCTGGGTGGCGCAGCGGTTTGGCGCCTGCCTTTGGCCCAGGGCGCGATCTTGGAGACCCGGGATCGAGTCCCACGTCGGGCTCCCGGTGCATGGAGC
sd_chr2_2140389   : TGCTTATGTTATATATGTAACTTAAAAGCACATCCAGGGGATCCCTGGGTGGCGCAGCGGTTTGGCGCCTGCCTTTGGCCCAGGGCGCGATCCTGGAGACCCGGGATCGAATCCCACGTCGGGCTCCCGGTGCATGGAGC
sb_chr37_12152614 : CTTTCTCTGTCTCTCGTGAATAAACAAAGCAAAACAGGGGATCCCTGGGTGGCGCAGCGGTTTGGCGCCTGCCTTTGGCCCAGGGCGCGATCCTGGAGACCCGGGATCGAGTCCCACGTCAGGCTCCCGGTGCATGGAGC
s_chr10_45498530  : TGACCTGCATGGTGTTTAATTAGAAATCAGCTATTCAGGGATCCCTGGGTGGCGCAGCGGTTTGGCGCCTGCCTTTGGCCCAGGGCGCGATCCTGGAGACCCGGGATCGAATCCCACGTCGGGCTCCCGGTGCATGGAGC
s_chr11_48191499  : TGAAAAGAATTAATGTGGTGCAAAAATGTTCTTTAGAGGGATCCCTGGGTGGCGCAGCGGTTTGGCGCCTGCCTTTGGCCCAGGGCGCGATCCTGGAGACCCGGGATCGAATCCCACGTCGGGCTCCCGGTGCATGGAGC
s_chr12_29180986  : TTAAGAAAATTCTCATGTATAAGAATTCAACTTGAGGGGGATCCCTGGGTGGCGCAGCGGTTTGGCGCCTGCCTTTGGCCCAGGGCGCGATCCTGGAGACCCGGGATCGAATCCCACGTCGGGCTCCCGGTGCATGGAGC
s_chr15_51391494  : CATGAATAAATAATAAAATCTTAAAAAAAAAAAACTAGGGATCCCTGGGTGGCGCAGCGGTTTGGCGCCTGCCTTTGGCCCAGGGCGCGATCCTGGAGACCCGGGATCGAATCCCACGTCGGGCTCCCGGTGCATGGAGC
s_chr20_41761467  : ATGAAGTGAGCAAACAGGTGTTAGAAAATGATGTCGAGGGATCCCTGGGTGGCGCAGCGGTTTGGCGCCTGCCTTTGGCCCAGGGCGCGATCCTGGAGACCCGGGATCGAGTCCCACGTCGGGCTCCCGGTGCATGGAGC
d_chr11_33750410  : CACTTGGCTTCTCAGTTGCCATAGAAAAGGGCATGTGGGGATCCCTGGGTGGCGCAGCGGTTTGGCGCCTGCCTTTGGCCCAGGGCGCGATCCTGGAGACCCGGGATCGAATCCCACGTCGGGCTCCCGGTGCATGGAGC
b_chr14_34729551  : GGATCACTGAGGGACTTCACTAGGAACAAAGGAGCTGGGGATCCCTGGGTGGCGCAGCGGTTTGGCGCCTGCCTTTGGCCCACGGCGCGATCCTGGAGACCCGGGATCGAGTCCCACGTCGGGCTCCCGGTGCATGGAGC
```

```
sdb_chr27_46295813: CTGCTTCTCCCTCTGCCTGTGTCTCTGCCTCTCCCTCTCTCTCT--------GTGACTATCCTAAATAAATAAATAAAA-TATTTAAAAAAAAAAAAAAAAAAAAAAAAAAAGAAATGCTGTCATCAAGGAAGAAAACATT
sdb_chr9_1474920  : CTGCTTCTCCCTCTGCCTGTGTCTCTGCCTCTCTCTCTCTCTCTCTCTCT--GTGACTATCCTAAATAAATAAATAAAA-TATTTAAAATAATAATAATAATAATAACAAAAGTCACAAACGAGAATGTGAGACGACAAT
sdb_chr23_22995549: CTGCTTCTCCCTCTGCCTGTGTCTCTGCCTCTCTCTCTCTCTCTCTCTCTCTGTGACTATCCTAAATAAATAAATAAAA-TATTAAAAAAAAAAAGATTTTTAAAAAACCAAAAAGGACTTTTGAGGCAATATTTATT
sdb_chr12_11820650: CTGCTTCTCCCTCTGCCTGTGTCTCTGCCTCTCTCTCTCTCTCTCT------GTGACTATCCTAAATAAATAAATAAAA-TATTTAAAAAAAATAAATAAATAAAATGCCAGTTAATATTTTCAGGCCTATTCACTTAGT
sdb_chr1_99860703 : CTGCTTCTCCCTCTGCCTGTGTCTCTGCCTCTCTCTCTCTCATTCT------GTGACTATCCTAAATAAATAAATAAAA-TATTTCAAAAAAAAAAAAAAAAAAGTAAAAATATTGGAAAATAAGTGACTCAGATCAGCTT
sdb_chrX_117917331: CTGCTTCTCCCTCTGCCTGTGTCTCTGCCTCTCTCTCTCTCTCTCTCTCT--GTGACTATCCTAAATAAATAAATAAAAATATTTAAAAAAAATACAATGAAATAAAATTAATTTTGCTTGAACAGAATTTGATAGCTTTA
sd_chr16_25666060 : CTGCTTCTCCCTCTGCCTGTGTCTCTGCCTCTCTCTCTCTCTCT--------GTGACTATCCTAAATAAATAAATAAAA-TATTAAAAAAAAAAAAAGAGATTAGGCTTTTCTATCTTCAATGAGAAGTAGAAGCATCTA
db_chr17_52597474 : CTGCTTCTCCCTCTGCCTGTGTCTCTGCCTCTCTCTCTCTCTCTCT------GTGACTATCCTAAATAAATAAATAAAA-TATTTAAAAAAAAAAAAAAAAAAAAGCTTTAACTTCTAGAGAAGTTAAAGAGCGTAGTTAG
sd_chr2_2140389   : CTGCTTCTCCCTCTGCCTGTGTCTCTGCCTCTCTCTCTCTCTCTCTCT----GTGACTATCCTAAATAAATAAATAAAA-TATTTAAAAAAAAAAAAAAAAAAAGCACATCCATAGAAATCTCCTATAAGCTGTACCTTCC
sb_chr37_12152614 : CTGCTTCTCCCTCTGCCTGTGTCTCTGCCTCTCTCTCTCTCTCT--------GTGACTATCCTAAATAAATAAATAAAA-TATTTAAAAAAAAAAAAAAAAAAAAAACAAAGCAAAACAAAACACGAAGATTTCTTGTAAG
s_chr10_45498530  : CTGCTTCTCCCTCTGCCTGTGTCTCTGCCTCTCTCTCTCTCATTCT------GTGACTATCCTAAATAAATAAATAAAA-TATTTAAAAAAAAAAAAAAAAAAAGAAATCAGCTATTCATTTTACTGAAGATTTCTTGCA
s_chr11_48191499  : CTGCTTCTCCCTCTGCCTGTGTCTCTGCCTCTCTCTCTCTCTCTCTCT----GTGACTATCCTAAATAAATAAATAAAA-TATTTAAAAAAAAAATGTTCTTTAGAGAATTGTGCTATAGAACTATAGTAGTTCATAAGAA
s_chr12_29180986  : CTGCTTCTCCCTCTGCCTGTGTCTCTGCCTCTCTCTCTCTCTCTCTCT----GTGACTATCCTAAATAAATAAATAAAA-TATTTAAAAAAAAAAAAAAAAAAAGAATTCAACTTGAGATGTCTCCAATATACATTTTA
s_chr15_51391494  : CTGCTTCTCCCTCTGCCTGTGTCTCTGCCTCTCTCTCTCTCATTCT------GTGACTATCCTAAATAAATAAATAAAA-TATTTAAAAAAAAAAAAAAAAAAAAAAAAAACTACTAGTTATTATTACAAAACCATTCATC
s_chr20_41761467  : CTGCTTCTCCCTCTGCCTGTGTCTCTGCCTCTCTCTCTCTCTCT--------GTGACTATCCTAAATAAATAAATAAAA-TATTAAAAAAAAAAAAGAGGGGATCCCTGGGTGGCGCAGCGGTTTGGCGCCTGCCTTTGG
d_chr11_33750410  : CTGCTTCTCCCTCTGCCTGTGTCTCTGCCTCTCTCTCTCTCTCTCTCTCT--GTGACTATCCTAAATAAATAAATAAAA-TATTAAAAAAGAAAAAAAAAAAAAAAAAGAAAAGGGCATGTGGTGGGCAGGGATGGGAAG
b_chr14_34729551  : CTGCTTCTCCCTCTGCCTGTGTCTCTGCCTCTCTCTCTCTCTCT--------GTGACTATCCTAAATAAATAAATAAAA-TATTAAAAAAAAAAAAAAAAAAAAAAAAAAGGAACAAAGGAGCTGTCATGAGCCATCTCTC
```

G

```
sdb_chr2_42598276 : GCTGCTCAAGAATATTATCAGCTTGGGGATCCCTGGGTGGCGCAGTGGTTTGGCGCCTGCCTTTGGCCCGGGGCGTGATCCTGGAGACCCGGGATCGAATCCCACGTCGGGCTCCCAGTGCATGGAGCCTGCTTCTCCCT
sb_chr15_63154005 : ATGTAAATAAAAATAAGTTTATCCGGGGATCCCTGGGTGGCGCAGTGGTTTGGCGCCTGCCTTTGGCCCGGGGCGTGATCCTGGAGACCCGGGATCGAATCCCACGTCGGGCTCCCAGTGCATGGAGCCTGCTTCTCCCT
s_chr28_20671404  : TAAAACTGTTCAAAAGTATTTCTTGGGGATCCCTGGGTGGCGCAGTGGTTTGGCGCCTGCCTTTGGCCCGGGGCGTGATCCTGGAGACCCGGGATCGAATCCCACGTCGGGCTCCCAGTGCATGGAGCCTGCTTCTCCCT
```

```
sdb_chr2_42598276 : CTGCCTGTGTCTCTGCCTCTCTCTCTCTCTCTCTCTCTCTCTGTGTGACTATCATAAATAAATAAAAATTTTAAAAAAAGAAATTAAAAAAAAAGAATATTATCAGCTGTGGAAAAGAACTGTGGACCATAGGACAAAAG
sb_chr15_63154005 : CTGCCTGTGTCTCTGCCTCTCTCTCTCTCTCTCTCTCTCT--GTGTGACTATCATAAATAAATAAAAATTTAAAAAAAAAAAAAAAAAATAAGTTTATCCTGTACACCCAGGTGGCTCAGCGGTTGAGCATCTGCCTTCA
s_chr28_20671404  : CTGCCTGTGTCTCTGCCTCTCTCTCTCTCTCTCTCTCTCTCTGTGTGACTATCATAAATAAATAAAAATTTAAAAAAAAAAAAAAAAAAAAAAAAAAAAAAAAAAAAACAAAAGTATTTCTTAAGGTATTATAAGAGGAAT
```

Figure S5. Seven examples (**A**-**G**) of groups of related Can_b2 copies whose structure indicates their retrotransposition by the T^+ mechanism. The copy names are given in black if present in the three dog breeds. The names of young copies found in one or two breeds are marked blue and violet, respectively. The letter prefix in sequence names indicates the genome where they are present (s, German Shepherd; d, Great Dane; and b, Boxer). Names of sequences with functional and rudimentary pol III transcription terminators are marked red and green, respectively; poly(A) tails are marked yellow; TSDs are underlined.
