# Supplementary material for: Tail Wags Dog’s SINE: Retropositional Mechanisms of Can SINE Depend on Its A-Tail Structure
Source: Biology (Basel). 2022 Sep 26;11(10):1403. doi: 10.3390/biology11101403 (PMC9599045; doi:10.3390/biology11101403)
Supplement: Supplementary file 1 [file biology-11-01403-s001.zip › Figure S6.pdf]

```
chr29 Can               : ------------------------TTTATATCAGGATATAATGGGGGATCCCTGGGTGGCGCAGCGGTTTGGCGCCTGCCTTTGGCCCAGGGCGCGATCC
s_CM021973.1:11564704  : GGTTTTTTGCTGTGGGAGAAAATGTTTTAAGATTCTCCATTGCTGGGATCCCTGGGTGGCGCAGCGGTTTGGCGCCTGCCTTTGGCCCAGGGCGCGATCC
d_CM016580.1:11386360  : GGTTTTTTGCTGTGGGAGAAAATGTTTTAAGATTCTCCATTGCTGGGATCCCTGGGTGGCGCAGCGGTTTGGCGCCTGCCTTTGGCCCAGGGCGCGATCC
b_CM000012.4:11404058  : GGTTTTTTGCTGTGGGAGAAAATGTTTTAAGATTCTCCATTGCTGGGATCCCTGGGTGGCGCAGCGGTTTGGCGCCTGCCTTTGGCCCAGGGCGCGATCC
b_CM000029.4:16380901  : TTATATTTATTTTACCAGATTGTTTTTATATCAGGATATAATGGGGGATCCCTGGGTGGCGCAGCGGTTTGGCGCCTGCCTTTGGCCCAGGGCGCGATCC
s_CM021990.1:16532681  : TTATATTTATTTTACCAGATTGTTTTTATATCAGGATATAATGGGGGATCCCTGGGTGGCGCAGCGGTTTGGCGCCTGCCTTTGGCCCAGGGCGCGATCC
d_CM016577.1:13904421  : CACAGTTTCACTTGTTGGGTTTTTGAATTTAAAACTAGGAAGAAGGGATCCCTGGGTGGCGCAGCGGTTTGGCGCCTGCCTTTGGCCCAGGGCGCGATCC
s_CM021965.1:28875877  : TTCTTTATAGTATAGTTTAAAATATTGAAAAAAAAAAAAAAAGGGGGATCCCTGGGTGGCGCAGCGGTTTGGCGCCTGCCTTTGGCCCAGGGCGCGATCC
d_CM016572.1:28320367  : TGTTCTTTATAGTATAGTTTAAAATATTGAAAAAAAAAAAAAGGGGGATCCCTGGGTGGCGCAGCGGTTTGGCGCCTGCCTTTGGCCCAGGGCGCGATCC
b_CM000004.4:28272540  : TGTTCTTTATAGTATAGTTTAAAATATTGAAAAAAAAAAAAAGGGGGATCCCTGGGTGGCGCAGCGGTTTGGCGCCTGCCTTTGGCCCAGGGCGCGATCC
s_CM021995.1:29864792  : GGAATTCATTGATGTTGAAGACTCTTTCAGAAAAACTGAATAGAGGGATCCCTGGGTGGCGCAGCGGTTTGGCGCCTGCCTTTGGCCCAGGGCGCGATCC
b_CM000034.4:38698570  : GGAATTCATTGATGTTGAAGACTCTTTCAGAAAAACTGAATAGAGGGATCCCTGGGTGGCGCAGCGGTTTGGCGCCTGCCTTTGGCCCAGGGCGCGATCC
d_CM016602.1:29638837  : GGAATTCATTGATGTTGAAGACTCTTTCAGAAAAACTGAATAGAGGGATCCCTGGGTGGCGCAGCGGTTTGGCGCCTGCCTTTGGCCCAGGGCGCGATCC
s_CM021979.1:45176522  : GTCTCTCATGAATAAATAAATAAAATCTTAAAAAGAAGGAAAGAGGGATCCCTGGGTGGCGCAGCGGTTTGGCGCCTGCCTTTGGCCCAGGGCGCGATCC
s_CM021973.1:55357485  : GAAATATCAGTAGTATTAGTAGATTGTTTAAGAAGTTGAATACTGGGATCCCTGGGTGGCGCAGCGGTTTGGCGCCTGCCTTTGGCCCAGGGCGCGATCC
d_CM016580.1:55248210  : GAAATATCAGTAGTATTAGTAGATTGTTTAAGAAGTTGAATACTGGGATCCCTGGGTGGCGCAGCGGTTTGGCGCCTGCCTTTGGCCCAGGGCGCGATCC
b_CM000012.4:54909836  : GAAATATCAGTAGTATTAGTAGATTGTTTAAGAAGTTGAATACTGGGATCCCTGGGTGGCGCAGCGGTTTGGCGCCTGCCTTTGGCCCAGGGCGCGATCC
s_CM021976.1:36514210  : TTTCATCCAGGTGGTAGAGAAGGAGACTTAAAAAGTCTAAACTCGGGATCCCTGGGTGGCGCAGCGGTTTGGCGCCTGCCTTTGGCCCAGGGCGCGATCC
b_CM000015.4:36603774  : TTTCATCCAGGTGGTAGAGAAGGAGACTTAAAAAGTCTAAACTCGGGATCCCTGGGTGGCGCAGCGGTTTGGCGCCTGCCTTTGGCCCAGGGCGCGATCC
s_CM021987.1:29177051  : CAGAAAAGGTGTTCCTTTTCAAAATGGAAAAAAAAAAACAATAGGGGATCCCTGGGTGGCGCAGCGGTTTGGCGCCTGCCTTTGGCCCAGGGCGCGATCC
b_CM000026.4:28974192  : CAGAAAAGGTGTTCCTTTTCAAAATGGAAAAAAAAAAACAATAGGGGATCCCTGGGTGGCGCAGCGGTTTGGCGCCTGCCTTTGGCCCAGGGCGCGATCC
s_CM021992.1:39506440  : CAACACCCAGGAAATGAACAATCCAGTCAAGAAATGGGCAGAAGGGGATCCCTGGGTGGCGCAGCGGTTTGGCGCCTGCCTTTGGCCCAGGGCGCGATCC
d_CM016599.1:39027764  : CAACACCCAGGAAATGAACAATCCAGTCAAGAAATGGGCAGAAGGGGATCCCTGGGTGGCGCAGCGGTTTGGCGCCTGCCTTTGGCCCAGGGCGCGATCC
b_CM000031.4:38752766  : CAACACCCAGGAAATGAACAATCCAGTCAAGAAATGGGCAGAAGGGGATCCCTGGGTGGCGCAGCGGTTTGGCGCCTGCCTTTGGCCCAGGGCGCGATCC
d_CM016588.1:3987471   : CAATTTACACGTTTAATGCAATCCCTATCAAAATACCATGGACTGGGATCCCTGGGTGGCGCAGCGGTTTGGCGCCTGCCTTTGGCCCAGGGCGCGATCC
s_CM021995.1:14669264  : -CAGGAGACTGAGCAGGGAGTGATGTCTTTCAAACTACGTTTCAGGGATCCCTGGGTGGCGCAGCGGTTTGGCGCCTGCCTTTGGCCCAGGGCGCGATCC
d_CM016602.1:14442100  : -CAGGAGACTGAGCAGGGAGTGATGTCTTTCAAACTACGTTTCAGGGATCCCTGGGTGGCGCAGCGGTTTGGCGCCTGCCTTTGGCCCAGGGCGCGATCC
b_CM000034.4:18577358  : -CAGGAGACTGAGCAGGGAGTGATGTCTTTCAAACTACGTTTCAGGGATCCCTGGGTGGCGCAGCGGTTTGGCGCCTGCCTTTGGCCCAGGGCGCGATCC
s_CM021983.1:15827289  : ACCTACTCCCATATATCCTCAACTGATATTAGAAAGTAAAAATAGGGATCCCTGGGTGGCGCAGCGGTTTGGCGCCTGCCTTTGGCCCAGGGCGCGATCC
b_CM000022.4:15829122  : ACCTACTCCCATATATCCTCAACTGATATTAGAAAGTAAAAATAGGGATCCCTGGGTGGCGCAGCGGTTTGGCGCCTGCCTTTGGCCCAGGGCGCGATCC
s_CM021974.1:52019303  : ATGTTATCATTGGGGGTAAAATTATCTATAGAAAGGGAGAAATTGGGATCCCTGGGTGGCGCAGCGGTTTGGCGCCTGCCTTTGGCCCAGGGCGCGATCC
b_CM000013.4:51160355  : ATGTTATCATTGGGGGTAAAATTATCTATAGAAAGGGAGAAATTGGGATCCCTGGGTGGCGCAGCGGTTTGGCGCCTGCCTTTGGCCCAGGGCGCGATCC
s_MU018711.1:230916    : -CAGAAATATTGATTCATCAATCAAGTCATCAAAAGTGAAGATGGGGATCCCTGGGTGGCGCAGCGGTTTGGCGCCTGCCTTTGGCCCAGGGCGCGATCC
s_CM021981.1:2716075   : ATTTCTAGAGTTGCACATTCAGTTATTTAAAATGTCTAGTTTTCGGGATCCCTGGGTGGCGCAGCGGTTTGGCGCCTGCCTTTGGCCCAGGGCGCGATCC
d_CM016588.1:2429172   : ATTTCTAGAGTTGCACATTCAGTTATTTAAAATGTCTAGTTTTCGGGATCCCTGGGTGGCGCAGCGGTTTGGCGCCTGCCTTTGGCCCAGGGCGCGATCC
b_CM000020.4:2724602   : ATTTCTAGAGTTGCACATTCAGTTATTTAAAATGTCTAGTTTTCGGGATCCCTGGGTGGCGCAGCGGTTTGGCGCCTGCCTTTGGCCCAGGGCGCGATCC
s_CM021980.1:49263820  : CTTTTTTTAATTTTCCATTTTAGTCCTATAAGGCAATGGTCCTGGGGATCCCTGGGTGGCGCAGCGGTTTGGCGCCTGCCTTTGGCCCAGGGCGCGATCC
d_CM016587.1:47735003  : CTTTTTTTAATTTTCCATTTTAGTCCTATAAGGCAATGGTCCTGGGGATCCCTGGGTGGCGCAGCGGTTTGGCGCCTGCCTTTGGCCCAGGGCGCGATCC
s_CM021967.1:7694852   : TAATGCCATTATCATGCCTAACAAAATTAATAATAATTCCTTTAGGGATCCCTGGGTGGCGCAGCGGTTTGGCGCCTGCCTTTGGCCCAGGGCGCGATCC
d_CM016574.1:7567605   : TAATGCCATTATCATGCCTAACAAAATTAATAATAATTCCTTTAGGGATCCCTGGGTGGCGCAGCGGTTTGGCGCCTGCCTTTGGCCCAGGGCGCGATCC
s_CM021962.1:106324370: GTAGCTTAAAATTTACTAATGGCCACATTTAAAAAGTAGAAAGAGGGATCCCTGGGTGGCGCAGCGGTTTGGCGCCTGCCTTTGGCCCAGGGCGCGATCC
d_CM016569.1:105888100: GTAGCTTAAAATTTACTAATGGCCACATTTAAAAAGTAGAAAGAGGGATCCCTGGGTGGCGCAGCGGTTTGGCGCCTGCCTTTGGCCCAGGGCGCGATCC
b_CM000001.4:105279680: GTAGCTTAAAATTTACTAATGGCCACATTTAAAAAGTAGAAAGAGGGATCCCTGGGTGGCGCAGCGGTTTGGCGCCTGCCTTTGGCCCAGGGCGCGATCC
s_CM021982.1:34324034  : ATACAATGAAAGGTCTGAGACTTTAAGAAAGAATTCAGGGCTCTGGGATCCCTGGGTGGCGCAGCGGTTTGGCGCCTGCCTTTGGCCCAGGGCGCGATCC
b_CM000021.4:33288465  : ATACAATGAAAGGTCTGAGACTTTAAGAAAGAATTCAGGGCTCTGGGATCCCTGGGTGGCGCAGCGGTTTGGCGCCTGCCTTTGGCCCAGGGCGCGATCC
s_CM021971.1:29008115  : TGGCATTCCCAAGTCTCTTACAGTGTCCTAAGAAGATCTCTCCAGGGATCCCTGGGTGGCGCAGCGGTTTGGCGCCTGCCTTTGGCCCAGGGCGCGATCC
d_CM016578.1:28521418  : TGGCATTCCCAAGTCTCTTACAGTGTCCTAAGAAGATCTCTCCAGGGATCCCTGGGTGGCGCAGCGGTTTGGCGCCTGCCTTTGGCCCAGGGCGCGATCC
```

```
b_CM000010.4:27962182  : TGGCATTCCCAAGTCTCTTACAGTGTCCTAAGAAGATCTCTCCAGGGATCCCTGGGTGGCGCAGCGGTTTGGCGCCTGCCTTTGGCCCAGGGCGCGATCC
d_CM016581.1:36417555  : CCAGCGAAGGGACGCTCCCGGGTCCTCTAGGAAAAGGCCAGCTCAGGATCCCTGGGTGGCGCAGCGGTTTGGCGCCTGCCTTTGGCCCAGGGCGCGATCC
b_CM000014.4:26681805  : TTTATACGGGATATGGATGAACAGCCTAATAAATTGGTACACTGGGGATCCCTGGGTGGCGCAGCGGTTTGGCGCCTGCCTTTGGCCCAGGGCGCGATCC
s_CM021983.1:49473247  : CCCAGGCACCCTGCTTTCTTTTGCTTTTTAATTTTATTTTTTGGGGGATCCCTGGGTGGCGCAGCGGTTTGGCGCCTGCCTTTGGCCCAGGGCGCGATCC
s_CM021969.1:53495922  : GTATAGTTTCTTTGATGATTTTTGTTTTTAAAGCTTCCTGTCTTGGGATCCCTGGGTGGCGCAGCGGTTTGGCGCCTGCCTTTGGCCCAGGGCGCGATCC
d_CM016576.1:53100186  : GTATAGTTTCTTTGATGATTTTTGTTTTTAAAGCTTCCTGTCTTGGGATCCCTGGGTGGCGCAGCGGTTTGGCGCCTGCCTTTGGCCCAGGGCGCGATCC
s_CM022000.1:124513739: AATTTTCATGTACTGGACAGACTAATTCTAAAATTTCCCATTCAGGGATCCCTGGGTGGCGCAGCGGTTTGGCGCCTGCCTTTGGCCCAGGGCGCGATCC
d_CM016607.1:122259922: AATTTTCATGTACTGGACAGACTAATTCTAAAATTTCCCATTCAGGGATCCCTGGGTGGCGCAGCGGTTTGGCGCCTGCCTTTGGCCCAGGGCGCGATCC
b_CM000039.4:108377960: AATTTTCATGTACTGGACAGACTAATTCTAAAATTTCCCATTCAGGGATCCCTGGGTGGCGCAGCGGTTTGGCGCCTGCCTTTGGCCCAGGGCGCGATCC
d_CM016583.1:30685492  : TCTTTTAGGTATGTTTGAAATTTTCTTTTAATAGAAAGCTTCTTGGGATCCCTGGGTGGCGCAGCGGTTTGGCGCCTGCCTTTGGCCCAGGGCGCGATCC
b_CM000015.4:31191229  : TCTTTTAGGTATGTTTGAAATTTTCTTTTAATAGAAAGCTTCTTGGGATCCCTGGGTGGCGCAGCGGTTTGGCGCCTGCCTTTGGCCCAGGGCGCGATCC
s_CM021983.1:27220998  : ATGACTGTATGGTTGTGAATTATATCTCATTAAAGCTAGTTTAAGGGATCCCTGGGTGGCGCAGCGGTTTGGCGCCTGCCTTTGGCCCAGGGCGCGATCC
d_CM016590.1:27110103  : ATGACTGTATGGTTGTGAATTATATCTCATTAAAGCTAGTTTAAGGGATCCCTGGGTGGCGCAGCGGTTTGGCGCCTGCCTTTGGCCCAGGGCGCGATCC
b_CM000022.4:26972534  : ATGACTGTATGGTTGTGAATTATATCTCATTAAAGCTAGTTTAAGGGATCCCTGGGTGGCGCAGCGGTTTGGCGCCTGCCTTTGGCCCAGGGCGCGATCC
s_CM021990.1:35056255  : TTTCAGAAAGTAACATCTTGATTTTGTTATTTAAAGGAAAAATAGGGATCCCTGGGTGGCGCAGCGGTTTGGCGCCTGCCTTTGGCCCAGGGCGCGATCC
b_CM000029.4:34574364  : TTTCAGAAAGTAACATCTTGATTTTGTTATTTAAAGGAAAAATAGGGATCCCTGGGTGGCGCAGCGGTTTGGCGCCTGCCTTTGGCCCAGGGCGCGATCC
b_CM000030.4:32702878  : TCTCTCATGAATAAATAAATAAAATCTTTAAAAGACAAATAAAGGGGATCCCTGGGTGGCGCAGCGGTTTGGCGCCTGCCTTTGGCCCAGGGCGCGATCC
s_CM021965.1:52147775  : AACTAATGTAATATTGTGCGTTACCTATAAAAAAATTTTTTTAGGGATCCCTGGGTGGCGCAGCGGTTTGGCGCCTGCCTTTGGCCCAGGGCGCGATCC
d_CM016572.1:51525463  : AACTAATGTAATATTGTGCGTTACCTATAAAAAAATTTTTTTAGGGATCCCTGGGTGGCGCAGCGGTTTGGCGCCTGCCTTTGGCCCAGGGCGCGATCC
b_CM000023.4:38752119  : GCATTTGCTTATTGGAGTATTTTTAGTAAAAGAATATATTTTCAGGGATCCCTGGGTGGCGCAGCGGTTTGGCGCCTGCCTTTGGCCCAGGGCGCGATCC
s_CM021995.1:32404702  : CTAATTTCGATCATGTCACTTGACTCTTTAAAACCCTGCAATGGGGGATCCCTGGGTGGCGCAGCGGTTTGGCGCCTGCCTTTGGCCCAGGGCGCGATCC
b_CM000034.4:41229417  : CTAATTTCGATCATGTCACTTGACTCTTTAAAACCCTGCAATGGGGGATCCCTGGGTGGCGCAGCGGTTTGGCGCCTGCCTTTGGCCCAGGGCGCGATCC
d_CM016602.1:32172425  : CTAATTTCGATCATGTCACTTGACTCTTTAAAACCCTGCAATGGGGGATCCCTGGGTGGCGCAGCGGTTTGGCGCCTGCCTTTGGCCCAGGGCGCGATCC
s_CM021997.1:14667324  : CTTTTAAAAAGAATAGTTCAGTGGTTCTTTAAAAAGTTAAACCTGGGATCCCTGGGTGGCGCAGCGGTTTGGCGCCTGCCTTTGGCCCAGGGCGCGATCC
d_CM016604.1:14565027  : CTTTTAAAAAGAATAGTTCAGTGGTTCTTTAAAAAGTTAAACCTGGGATCCCTGGGTGGCGCAGCGGTTTGGCGCCTGCCTTTGGCCCAGGGCGCGATCC
s_CM021963.1:71894134  : GCCCTCCATTTTCTCCTGTGCTCCTTTTTTAAAAAATTAAATTAGGGATCCCTGGGTGGCGCAGCGGTTTGGCGCCTGCCTTTGGCCCAGGGCGCGATCC
s_CM021983.1:45943175  : TGTGAATTACGAAACTGTGTAGTACAATTTAGAAACGTAGCAGGGGGATCCCTGGGTGGCGCAGCGGTTTGGCGCCTGCCTTTGGCCCAGGGCGCGATCC
s_CM021963.1:18318682  : CTGGGCCTGCTTTCAAAGAGTTTGTTTTACAAAAGGACAAAATAGGGATCCCTGGGTGGCGCAGCGGTTTGGCGCCTGCCTTTGGCCCAGGGCGCGATCC
b_CM000002.4:16472404  : CTGGGCCTGCTTTCAAAGAGTTTGTTTTACAAAAGGACAAAATAGGGATCCCTGGGTGGCGCAGCGGTTTGGCGCCTGCCTTTGGCCCAGGGCGCGATCC
s_CM021981.1:32911909  : TAATGACAGGAATAGAAGAAAGTGAAATTTAAAAATGTGCCATAGGGATCCCTGGGTGGCGCAGCGGTTTGGCGCCTGCCTTTGGCCCAGGGCGCGATCC
d_CM016588.1:32382961  : TAATGACAGGAATAGAAGAAAGTGAAATTTAAAAATGTGCCATAGGGATCCCTGGGTGGCGCAGCGGTTTGGCGCCTGCCTTTGGCCCAGGGCGCGATCC
b_CM000020.4:32582772  : TAATGACAGGAATAGAAGAAAGTGAAATTTAAAAATGTGCCATAGGGATCCCTGGGTGGCGCAGCGGTTTGGCGCCTGCCTTTGGCCCAGGGCGCGATCC
s_CM021991.1:17604839  : TTCTTCTGATTTGGAAGTGACCTTCTTTTAAAAAATAGAAAGGTGGGATCCCTGGGTGGCGCAGCGGTTTGGCGCCTGCCTTTGGCCCAGGGCGCGATCC
d_CM016598.1:17380206  : TTCTTCTGATTTGGAAGTGACCTTCTTTTAAAAAATAGAAAGGTGGGATCCCTGGGTGGCGCAGCGGTTTGGCGCCTGCCTTTGGCCCAGGGCGCGATCC
s_CM021972.1:23265552  : TCAGTGCTGCCTCCTGGCCCTGTACCTCTTTAGAACAAAATTGGGGGATCCCTGGGTGGCGCAGCGGTTTGGCGCCTGCCTTTGGCCCAGGGCGCGATCC
d_CM016579.1:22756425  : TCAGTGCTGCCTCCTGGCCCTGTACCTCTTTAGAACAAAATTGGGGGATCCCTGGGTGGCGCAGCGGTTTGGCGCCTGCCTTTGGCCCAGGGCGCGATCC
b_CM000011.4:22000418  : TCAGTGCTGCCTCCTGGCCCTGTACCTCTTTAGAACAAAATTGGGGGATCCCTGGGTGGCGCAGCGGTTTGGCGCCTGCCTTTGGCCCAGGGCGCGATCC
s_CM021988.1:9926312   : GAGTTTTTAAGTCATCTTTGTTCAGCAATAAAAAAATTCCATTAGGGATCCCTGGGTGGCGCAGCGGTTTGGCGCCTGCCTTTGGCCCAGGGCGCGATCC
b_CM000027.5:10401007  : GAGTTTTTAAGTCATCTTTGTTCAGCAATAAAAAAATTCCATTAGGGATCCCTGGGTGGCGCAGCGGTTTGGCGCCTGCCTTTGGCCCAGGGCGCGATCC
b_CM000008.4:72800272  : TTTAACACCAATGAAAATAAATTTATTATTTAAAAAATAACATAGGGATCCCTGGGTGGCGCAGCGGTTTGGCGCCTGCCTTTGGCCCAGGGCGCGATCC
s_CM021970.1:18974382  : GCAAAACCACAGTCTGGTTATCACGCTCAAGAAATTCAACATTGGGGATCCCTGGGTGGCGCAGCGGTTTGGCGCCTGCCTTTGGCCCAGGGCGCGATCC
s_CM021998.1:30009360  : CAAACAAACACCATGAAACACAAGCTTAAAGATAGTAACTTCTAGGGATCCCTGGGTGGCGCAGCGGTTTGGCGCCTGCCTTTGGCCCAGGGCGCGATCC
d_CM016605.1:30057161  : CAAACAAACACCATGAAACACAAGCTTAAAGATAGTAACTTCTAGGGATCCCTGGGTGGCGCAGCGGTTTGGCGCCTGCCTTTGGCCCAGGGCGCGATCC
b_CM000037.4:30888146  : CAAACAAACACCATGAAACACAAGCTTAAAGATAGTAACTTCTAGGGATCCCTGGGTGGCGCAGCGGTTTGGCGCCTGCCTTTGGCCCAGGGCGCGATCC
b_CM000032.5:23742260  : CCAAGAATTAGAGTAACCAGCCAACTAGCAAAAAACTGATCTACGGGATCCCTGGG-GGCGCAGCGG-TTGGCGCCTGCCTTTGG-CCAGGGCGCGATCC
```

```
chr29 Can               : TGGAGACCCGGGATCGAATCCCACGTCGGGCTT--CCGGTGCATGGAGCCTGCTTCTCCCTCTGCCTATGTCTCTGC----CTCTCTCTCTCTCACTG
s_CM021973.1:11564704   : TGGAGACCCGGGATCGAATCCCACGTCGGGCTT--CCGGTGCATGGAGCCTGCTTCTCCCTCTGCCTATGTCTCTGC----CTCTCTCTCTCTCACTG
d_CM016580.1:11386360   : TGGAGACCCGGGATCGAATCCCACGTCGGGCTT--CCGGTGCATGGAGCCTGCTTCTCCCTCTGCCTATGTCTCTGC----CTCTCTCTCTCTCACTG
b_CM000012.4:11404058   : TGGAGACCCGGGATCGAATCCCACGTCGGGCTT--CCGGTGCATGGAGCCTGCTTCTCCCTCTGCCTATGTCTCTGC----CTCTCTCTCTCTCACTG
b_CM000029.4:16380901   : TGGAGACCCGGGATCGAATCCCACGTCGGGCTT--CCGGTGCATGGAGCCTGCTTCTCCCTCTGCCTATGTCTCTGC----CTCTCTCTCTCTCACTG
s_CM021990.1:16532681   : TGGAGACCCGGGATCGAATCCCACGTCGGGCTT--CCGGTGCATGGAGCCTGCTTCTCCCTCTGCCTATGTCTCTGC----CTCTCTCTCTCTCACTG
d_CM016577.1:13904421   : TGGAGACCCGGGATCGAATCCCACGTCGGGCTT--CCGGTGCATGGAGCCTGCTTCTCCCTCTGCCTATGTCTCTGC----CTCTCTCTCTCTCACTG
s_CM021965.1:28875877   : TGGAGACCCGGGATCGAATCCCACGTCGGGCTT--CCGGTGCATGGAGCCTGCTTCTCCCTCTGCCTGTGTCTCTGC----CTCTCTCTCTCTCACTG
d_CM016572.1:28320367   : TGGAGACCCGGGATCGAATCCCACGTCGGGCTT--CCGGTGCATGGAGCCTGCTTCTCCCTCTGCCTGTGTCTCTGC----CTCTCTCTCTCTCACTG
b_CM000004.4:28272540   : TGGAGACCCGGGATCGAATCCCACGTCGGGCTT--CCGGTGCATGGAGCCTGCTTCTCCCTCTGCCTGTGTCTCTGC----CTCTCTCTCTCTCACTG
s_CM021995.1:29864792   : TGGAGACCCGGGATCGAATCCCACGTCGGGCTT--CCGGTGCATGGAGCCTGCTTCTCCCTCTGCCTGTGTCTCTGC----CTCTCTCTCTCTCACTG
b_CM000034.4:38698570   : TGGAGACCCGGGATCGAATCCCACGTCGGGCTT--CCGGTGCATGGAGCCTGCTTCTCCCTCTGCCTGTGTCTCTGC----CTCTCTCTCTCTCACTG
d_CM016602.1:29638837   : TGGAGACCCGGGATCGAATCCCACGTCGGGCTT--CCGGTGCATGGAGCCTGCTTCTCCCTCTGCCTGTGTCTCTGC------CTCTCTCTCTCACTG
s_CM021979.1:45176522   : TGGAGACCCGGGATCGAATCCCACGTCGGGCTT--CCGGTGCATGGAGCCTGCTTCTCCCTCTGCCTGTGTCTCTGC----CTCTCTCTCTCTCACTG
s_CM021973.1:55357485   : TGGAGACCCGGGATCGAATCCCACGTCGGGCTT--CCGGTGCATGGAGCCTGCTTCTCCCTCTGCCTGTGTCTCTGC----CTCTCTCTCTCTCACTG
d_CM016580.1:55248210   : TGGAGACCCGGGATCGAATCCCACGTCGGGCTT--CCGGTGCATGGAGCCTGCTTCTCCCTCTGCCTGTGTCTCTGC----CTCTCTCTCTCTCACTG
b_CM000012.4:54909836   : TGGAGACCCGGGATCGAATCCCACGTCGGGCTT--CCGGTGCATGGAGCCTGCTTCTCCCTCTGCCTGTGTCTCTGC----CTCTCTCTCTCTCACTG
s_CM021976.1:36514210   : TGGAGACCCGGGATCGAATCCCACGTCGGGCTT--CCGGTGCATGGAGCCTGCTTCTCCCTCTGCCTATGTCTCTGC----CTCTCTCTCTCTCACTG
b_CM000015.4:36603774   : TGGAGACCCGGGATCGAATCCCACGTCGGGCTT--CCGGTGCATGGAGCCTGCTTCTCCCTCTGCCTATGTCTCTGC----CTCTCTCTCTCTCACTG
s_CM021987.1:29177051   : TGGAGACCCGGGATCGAATCCCACGTCGGGCTT--CCGGTGCATGGAGCCTGCTTCTCCCTCTGCCTATGTCTCTGC----CTCTCTCTCTCTCACTG
b_CM000026.4:28974192   : TGGAGACCCGGGATCGAATCCCACGTCGGGCTT--CCGGTGCATGGAGCCTGCTTCTCCCTCTGCCTATGTCTCTGC----CTCTCTCTCTCTCACTG
s_CM021992.1:39506440   : TGGAGACCCGGGATCGAATCCCACGTCGGGCTT--CCGGTGCATGGAGCCTGCTTCTCCCTCTGCCTATGTCTCTGC----CTCTCTCTCTCTCACTG
d_CM016599.1:39027764   : TGGAGACTCGGGATCGAATCCCACGTCGGGCTT--CCGGTGCATGGAGCCTGCTTCTCCCTCTGCCTATGTCTCTGC----CTCTCTCTCTCTCACTG
b_CM000031.4:38752766   : TGGAGACCCGGGATCGAATCCCACGTCGGGCTT--CCGGTGCATGGAGCCTGCTTCTCCCTCTGCCTATGTCTCTGC----CTCTCTCTCTCTCACTG
d_CM016588.1:3987471    : TGGAGACCCGGGATCGAATCCCACGTCGGGCTT--CCGGTGCATGGAGCCTGCTTCTCCCTCTGCCTATGTCTCTGC----CTCTCTCTCTCTCACTG
s_CM021995.1:14669264   : TGGAGACCCGGGATCGAATCCCACGTCGGGCTT--CCGGTGCATGGAGCCTGCTTCTCCCTCTGCCTGTGTCTCTGC----CTCTCTCTCTCTCACTG
d_CM016602.1:14442100   : TGGAGACCCGGGATCGAATCCCACGTCGGGCTT--CCGGTGCATGGAGCCTGCTTCTCCCTCTGCCTGTGTCTCTGC----CTCTCTCTCTCTCACTG
b_CM000034.4:18577358   : TGGAGACCCGGGATCGAATCCCACGTCGGGCTT--CCGGTGCATGGAGCCTGCTTCTCCCTCTGCCTGTGTCTCTGC----CTCTCTCTCTCTCACTG
s_CM021983.1:15827289   : TGGAGACCCGGGATCGAATCCCACGTCGGGCTT--CCGGTGCATGGAGCCTGCTTCTCCCTCTGCCTATGTCTCTGC----CTCTCTCTCTCTCACTG
b_CM000022.4:15829122   : TGGAGACCCGGGATCGAATCCCACGTCGGGCTT--CCGGTGCATGGAGCCTGCTTCTCCCTCTGCCTATGTCTCTGC----CTCTCTCTCTCTCACTG
s_CM021974.1:52019303   : TGGAGACCCGGGATCGAATCCCACGTCGGGCTT--CCGGTGCATGGAGCCTGCTTCTCCCTCTGCCTGTGTCTCTGC--CTCTCTCTCTCTCTCACTG
b_CM000013.4:51160355   : TGGAGACCCGGGATCGAATCCCACGTCGGGCTT--CCGGTGCATGGAGCCTGCTTCTCCCTCTGCCTGTGTCTCTGC----CTCTCTCTCTCTCACTG
s_MU018711.1:230916     : TGGAGACCCGGGATCGAATCCCACGTCGGGCTC--CCGGTGCATGGAGCCTGCTTCTCCCTCTGCCTATGTCTCTGC----CTCTCTCTCTCTCACTG
s_CM021981.1:2716075    : TGGAGACCCGGGATCGAATCCCACGTCGGGCTT--CCGGTGCATGGAGCCTGCTTCTCCCTCTGCCTGTGTCTCTGC----CTCTCTCTCTCTCACTG
d_CM016588.1:2429172    : TGGAGACCCGGGATCGAATCCCACGTCGGGCTT--CCGGTGCATGGAGCCTGCTTCTCCCTCTGCCTGTGTCTCTGC--CTCTCTCTCTCTCTCACTG
b_CM000020.4:2724602    : TGGAGACCCGGGATCGAATCCCACGTCGGGCTT--CCGGTGCATGGAGCCTGCTTCTCCCTCTGCCTGTGTCTCTGC----CTCTCTCTCTCTCACTG
s_CM021980.1:49263820   : TGGAGACCCGGGATCGAATCCCACGTCGGGCTT--CCGGTGCATGGAGCCTGCTTCTCCCTCTGCCTGTGTCTCTGC----CTCTCTCTCTCTCACTG
d_CM016587.1:47735003   : TGGAGACCCGGGATCGAATCCCACGTCGGGCTT--CCGGTGCATGGAGCCTGCTTCTCCCTCTGCCTGTGTCTCTGC----CTCTCTCTCTCTCACTG
s_CM021967.1:7694852    : TGGAGACCCGGGATCGAATCCCACGTCGGGCTT--CCGGTGCATGGAGCCTGCTTCTCCCTCTGCCTGTGTCTCTGC----CTCTCTCTCTCTCACTG
d_CM016574.1:7567605    : TGGAGACCCGGGATCGAATCCCACGTCGGGCTT--CCGGTGCATGGAGCCTGCTTCTCCCTCTGCCTGTGTCTCTGC----CTCTCTCTCTCTCACTG
s_CM021962.1:106324370: TGGAGACCCGGGATCGAATCCCACGTCGGGCTT--CCGGTGCATGGAGCCTGCTTCTCCCTCTGCCTGTGTCTCTGC----CTCTCTCTCTCTCACTG
d_CM016569.1:105888100: TGGAGACCCGGGATCGAATCCCACGTCGGGCTT--CCGGTGCATGGAGCCTGCTTCTCCCTCTGCCTGTGTCTCTGC----CTCTCTCTCTCTCACTG
b_CM000001.4:105279680: TGGAGACCCGGGATCGAATCCCACGTCGGGCTT--CCGGTGCATGGAGCCTGCTTCTCCCTCTGCCTGTGTCTCTGC----CTCTCTCTCTCTCACTG
s_CM021982.1:34324034   : TGGAGACCCGGGATCGAATCCCACGTCGGGCTT--CCGGTGCATGGAGCCTGCTTCTCCCTCTGCCTGTGTCTCTGC----CTCTCTCTCTCTCACTG
b_CM000021.4:33288465   : TGGAGACCCGGGATCGAATCCCACGTCGGGCTT--CCGGTGCATGGAGCCTGCTTCTCCCTCTGCCTGTGTCTCTGC----CTCTCTCTCTCTCACTG
s_CM021971.1:29008115   : TGGAGACCCGGGATCGAATCCCACGTCGGGCTT--CCGGTGCATGGAGCCTGCTTCTCCCTCTGCCTGTGTCTCTGC----CTCTCTCTCTCTCACTG
d_CM016578.1:28521418   : TGGAGACCCGGGATCGAATCCCACGTCGGGCTT--CCGGTGCATGGAGCCTGCTTCTCCCTCTGCCTGTGTCTCTGC----CTCTCTCTCTCTCACTG
b_CM000010.4:27962182   : TGGAGACCCGGGATCGAATCCCACGTCGGGCTT--CCGGTGCATGGAGCCTGCTTCTCCCTCTGCCTGTGTCTCTGCCTCTCTCTCTCTCTCTCACTG
```

```
d_CM016581.1:36417555 : TGGAGACCCGGGATCGAATCCCACGTCGGGCTT--CCGGTGCATGGAGCCTGCTTCTCCCTCTGCCTGTGTCTCTGC----CTCTCTCTCTCTCTCACTG
b_CM000014.4:26681805 : TGGAGACCCGGGATCGAATCCCACGTCGGGCTT--CCGGTGCATGGAGCCTGCTTCTCCCTCTGCCTGTGTCTCTGC----CTCTCTCTCTCTCTCACTG
s_CM021983.1:49473247 : TGGAGACCCGGGATCGAATCCCACGTCGGGCTT--CCGGTGCATGGAGCCTGCTTCTCCCTCTGCCTGTGTCTCTGC----CTCTCTCTCTCTCTCACTG
s_CM021969.1:53495922 : TGGAGACCCGGGATCGAATCCCACGTCGGGCTT--CCGGTGCATGGAGCCTGCTTCTCCCTCTGCCTGTGTCTCTGC----CTCTCTCTCTCTCTCACTG
d_CM016576.1:53100186 : TGGAGACCCGGGATCGAATCCCACGTCGGGCTT--CCGGTGCATGGAGCCTGCTTCTCCCTCTGCCTGTGTCTCTGC----CTCTCTCTCTCTCTCACTG
s_CM022000.1:124513739: TGGAGACCCGGGATCGAATCCCACGTCGGGCTT--CCGGTGCATGGAGCCTGCTTCTCCCTCTGCCTGTGTCTCTGC--CTCTCTCTCTCTCTCTCACTG
d_CM016607.1:122259922: TGGAGACCCGGGATCGAATCCCACGTCGGGCTT--CCGGTGCATGGAGCCTGCTTCTCCCTCTGCCTGTGTCTCTGC--CTCTCTCTCTCTCTCTCACTG
b_CM000039.4:108377960: TGGAGACCCGGGATCGAATCCCACGTCGGGCTT--CCGGTGCATGGAGCCTGCTTCTCCCTCTGCCTGTGTCTCTGC--CTCTCTCTCTCTCTCTCACTG
d_CM016583.1:30685492 : TGGAGACCCGGGATCGAATCCCACGTCGGGCTT--CCGGTGCATGGAGCCTGCTTCTCCCTCTGCCTGTGTCTCTGC------CTCTCTCTCTCTCACTG
b_CM000015.4:31191229 : TGGAGACCCGGGATCGAATCCCACGTCGGGCTT--CCGGTGCATGGAGCCTGCTTCTCCCTCTGCCTGTGTCTCTGC------CTCTCTCTCTCTCACTG
s_CM021983.1:27220998 : TGGAGACCCGGGATCGAATCCCACGTCGGGCTT--CCGGTGCATGGAGCCTGCTTCTCCCTCTGCCTGTGTCTCTGCCTCTCTCTCTCTCTCTCTCACTG
d_CM016590.1:27110103 : TGGAGACCCGGGATCGAATCCCACGTCGGGCTT--CCGGTGCATGGAGCCTGCTTCTCCCTCTGCCTGTGTCTCTGCCTCTCTCTCTCTCTCTCTCACTG
b_CM000022.4:26972534 : TGGAGACCCGGGATCGAATCCCACGTCGGGCTT--CCGGTGCATGGAGCCTGCTTCTCCCTCTGCCTGTGTCTCTGCCTCTCTCTCTCTCTCTCTCACTG
s_CM021990.1:35056255 : TGGAGACCCGGGATCGAATCCCACGTCGGGCTT--CCGGTGCATGGAGCCTGCTTCTCCCTCTGCCTGTGTCTCTGC--CTCTCTCTCTCTCTCTCACTG
b_CM000029.4:34574364 : TGGAGACCCGGGATCGAATCCCACGTCGGGCTT--CCGGTGCATGGAGCCTGCTTCTCCCTCTGCCTGTGTCTCTGC--CTCTCTCTCTCTCTCTCACTG
b_CM000030.4:32702878 : TGGAGACCCGGGATCGAATCCCACGTCGGGCTT--CCGGTGCATGGAGCCTGCTTCTCCCTCTGCCTGTGTCTCTGC--CTCTCTCTCTCTCTCTCACTG
s_CM021965.1:52147775 : TGGAGACCCGGGATCGAATCCCACGTCGGGCTT--CCGGTGCATGGAGCCTGCTTCTCCCTCTGCCTGTGTCTCTGC--CTCTCTCTCTCTCTCTCACTG
d_CM016572.1:51525463 : TGGAGACCCGGGATCGAATCCCACGTCGGGCTT--CCGGTGCATGGAGCCTGCTTCTCCCTCTGCCTGTGTCTCTGC--CTCTCTCTCTCTCTCTCACTG
b_CM000023.4:38752119 : TGGAGACCCGGGATCGAATCCCACGTCGGGCTT--CCGGTGCATGGAGCCTGCTTCTCCCTCTGCCTGTGTCTCTGC--CTCTCTCTCTCTCTCTCACTG
s_CM021995.1:32404702 : TGGAGACCCGGGATCGAATCCCACGTCGGGCTT--CCGGTGCATGGAGCCTGCTTCTCCCTCTGCCTGTGTCTCTGC--CTCTCTCTCTCTCTCTCACTG
b_CM000034.4:41229417 : TGGAGACCCGGGATCGAATCCCACGTCGGGCTT--CCGGTGCATGGAGCCTGCTTCTCCCTCTGCCTGTGTCTCTGC--CTCTCTCTCTCTCTCTCACTG
d_CM016602.1:32172425 : TGGAGACCCGGGATCGAATCCCACGTCGGGCTT--CCGGTGCATGGAGCCTGCTTCTCCCTCTGCCTGTGTCTCTGC--CTCTCTCTCTCTCTCTCACTG
s_CM021997.1:14667324 : TGGAGACCCGGGATCGAATCCCACGTCGGGCTT--CCGGTGCATGGAGCCTGCTTCTCCCTCTGCCTGTGTCTCTGC--CTCTCTCTCTCTCTCTCACTG
d_CM016604.1:14565027 : TGGAGACCCGGGATCGAATCCCACGTCGGGCTT--CCGGTGCATGGAGCCTGCTTCTCCCTCTGCCTGTGTCTCTGC--CTCTCTCTCTCTCTCTCACTG
s_CM021963.1:71894134 : TGGAGACCCGGGATCGAATCCCACGTCGGGCTT--CCGGTGCATGGAGCCTGCTTCTCCCTCTGCCTGTGTCTCTGC--CTCTCTCTCTCTCTCTCACTG
s_CM021983.1:45943175 : TGGAGACCCGGGATCGAATCCCACGTCGGGCTT--CCGGTGCATGGAGCCTGCTTCTCCCTCTGCCTGTGTCTCTGC--CTCTCTCTCTCTCTCTCACTG
s_CM021963.1:18318682 : TGGAGACCCGGGATCGAATCCCACGTCGGGCTT--CCGGTGCATGGAGCCTGCTTCTCCCTCTGCCTATGTCTCTGC----CTCTCTCTCTCTCTCACTG
b_CM000002.4:16472404 : TGGAGACCCGGGATCGAATCCCACGTCGGGCTT--CCGGTGCATGGAGCCTGCTTCTCCCTCTGCCTATGTCTCTGC----CTCTCTCTCTCTCTCACTG
s_CM021981.1:32911909 : TGGAGACCCGGGATCGAATCCCACGTCGGGCTT--CCGGTGCATGGAGCCTGCTTCTCCCTCTGCCTGTGTCTCTGC--CTCTCTCTCTCTCTCTCACTG
d_CM016588.1:32382961 : TGGAGACCCGGGATCGAATCCCACGTCGGGCTT--CCGGTGCATGGAGCCTGCTTCTCCCTCTGCCTGTGTCTCTGC--CTCTCTCTCTCTCTCTCACTG
b_CM000020.4:32582772 : TGGAGACCCGGGATCGAATCCCACGTCGGGCTT--CCGGTGCATGGAGCCTGCTTCTCCCTCTGCCTGTGTCTCTGCCTCTCTCTCTCTCTCTCTCACTG
s_CM021991.1:17604839 : TGGAGACCCGGGATCGAATCCCACGTCGGGCTT--CCGGTGCATGGAGCCTGCTTCTCCCTCTGCCTGTGTCTCTGC--CTCTCTCTCTCTCTCTCACTG
d_CM016598.1:17380206 : TGGAGACCCGGGATCGAATCCCACGTCGGGCTT--CCGGTGCATGGAGCCTGCTTCTCCCTCTGCCTGTGTCTCTGC--CTCTCTCTCTCTCTCTCACTG
s_CM021972.1:23265552 : TGGAGACCCGGGATCGAATCCCACATCGGGCTT--CCGGTGCATGGAGCCTGCTTCTCCCTCTGCCTATGTCTCTGC----CTCTCTCTCTCTCTCACTG
d_CM016579.1:22756425 : TGGAGACCCGGGATCGAATCCCACGTCGGGCTT--CCGGTGCATGGAGCCTGCTTCTCCCTCTGCCTATGTCTCTGC----CTCTCTCTCTCTCTCACTG
b_CM000011.4:22000418 : TGGAGACCCGGGATCGAATCCCACGTCGGGCTT--CCGGTGCATGGAGCCTGCTTCTCCCTCTGCCTATGTCTCTGC----CTCTCTCTCTCTCTCACTG
s_CM021988.1:9926312  : TGGAGACCCGGGATCGAATCCCACGTCGGGCTT--CCGGTGCATGGAGCCTGCTTCTCCCTCTGCCTATGTCTCTGC------CTCTCTCTCTCTCACTG
b_CM000027.5:10401007 : TGGAGACCCGGGATCGAATCCCACGTCGGGCTT--CCGGTGCATGGAGCCTGCTTCTCCCTCTGCCTATGTCTCTGC------CTCTCTCTCTCTCACTG
b_CM000008.4:72800272 : TGGAGACCCGGGATCGAATCCCACGTCGGGCTTCCCCGGTGCATGGAGCCTGCTTCTCCCTCTGCCTGTGTCTCTGC------CTCTCTCTCTCTCACTG
s_CM021970.1:18974382 : TGGAGACCCGGGATCGAATCCCACGTCGGGCTTCCCCGGTGCATGGAGCCTGCTTCTCCCTCTGCCTGTGTCTCTGC------CTCTCTCTCTCTCACTG
s_CM021998.1:30009360 : TGGAGACCCGGGATCGAATCCCACGTCGGGCTC--CCGGTGCATGGAGCCTGCTTCTCCCTCTGCCTGTCTCT--------GCCTCTCTCTCTCTCACTG
d_CM016605.1:30057161 : TGGAGACCCGGGATCGAATCCCACGTCGGGCTC--CCGGTGCATGGAGCCTGCTTCTCCCTCTGCCTGTCTCT--------GCCTCTCTCTCTCTCACTG
b_CM000037.4:30888146 : TGGAGACCCGGGATCGAATCCCACGTCGGGCTC--CCGGTGCATGGAGCCTGCTTCTCCCTCTGCCTGTCTCT--------GCCTCTCTCTCTCTCACTG
b_CM000032.5:23742260 : TGGAGACCCGGGATCGAATCCCACGTCGGGCTT--CCGGTGCAT-GAGCCTGCTTCTCCCTCTGCCTGTGTCTCTGC--CTCTCTCTCTCTCTCTCACTG
```

```
chr29 Can              : TGTGCCTATCATAAATAAAT-----AAAAAATT-AAAAAAAAAAAGGATATAATGGGCAAGAGATG----------------------------------
s_CM021973.1:11564704  : TGTGCCTATCATAAATAAAT-----AAAAAATT-AAAAAAAAAAAAAAAAAAAGATTCTCCATTGCTATCTTTTGGCACGAGAAACATTTCTAGAAATTG
d_CM016580.1:11386360  : TGTGCCTATCATAAATAAAT-----AAAAAATT-AAAAAAAAAAAAAAAAAAAGATTCTCCATTGCTATCTTTTGGCACGAGAAACATTTCTAGAAATTG
b_CM000012.4:11404058  : TGTGCCTATCATAAATAAAT-----AAAAAATT--AAAAAAAAAAAAAAAAAAGATTCTCCATTGCTATCTTTTGGCACGAGAAACATTTCTAGAAATTG
b_CM000029.4:16380901  : TGTGCCTATCATAAATAAAT-----AAAAAATT-AAAAAAAAAAAAAAGGATATAATGGGCAAGAGATGTTTGTTCTACTGTTCATTAGTTGTAGATGAG
s_CM021990.1:16532681  : TGTGCCTATCATAAATAAAT-----AAAAAATT----AAAAAAAAAAAGGATATAATGGGCAAGAGATGTTTGTTCTACTGTTCATTAGTTGTAGATGAG
d_CM016577.1:13904421  : TGTGCCTATCATAAATAAAT-----AAAAAATTTAAAAAAAAAAATTTAAAACTAGGAAGAAGTATTTGCTTTTAAATCTCAAGTGAAATTTGAATTTTA
s_CM021965.1:28875877  : TGTGCCTATCATAAATAAAT-----AAAAAATT-AAAAAAAAAAAAAAAAAAAAGAACCGAACTTAAGGTCATCCAGCCCTCCCATTTGATTACTGAATT
d_CM016572.1:28320367  : TGTGCCTATCATAAATAAAT-----AAAAAATT--AAAAAAAAAAAAAAAAAAAGAACCGAACTTAAGGTCATCCAGCCCTCCCATTTGATTACTGAATT
b_CM000004.4:28272540  : TGTGCCTATCATAAATAAAT-----AAAAAATT--AAAAAAAAAAAAAAAAAAAGAACCGAACTTAAGGTCATCCAGCCCTCCCATTTGATTACTGAATT
s_CM021995.1:29864792  : TGTGCCTATCATAAATAAAT-----AAAAAATT---AAAAAAAAAAAAAAAGAAAAACTGAATAGATAATTGTCTCCTTCTCAGCTGAGCCTTTGAACCT
b_CM000034.4:38698570  : TGTGCCTATCATAAATAAAT-----AAAAAATT--AAAAAAAAAAAAAAAAGAAAAACTGAATAGATAATTGTCTCCTTCTCAGCTGAGCCTTTGAACCT
d_CM016602.1:29638837  : TGTGCCTATCATAAATAAAT-----AAAAAATTT-AAAAAAAAAAAAAAAAGAAAAACTGAATAGATAATTGTCTCCTTCTCAGCTGAGCCTTTGAACCT
s_CM021979.1:45176522  : TGTGCCTATCATAAATAAAT-----AAAAAATT-AAAAAAAAAAAAGAAGGAAAGAAAGATGTCTTGTCTCGTATACAGGTAATTAAACAGCCAGTTACA
s_CM021973.1:55357485  : TGTGCCTATCATAAATAAAT-----AAAAAATT-AAAAAAAAAAAAGAAGTTGAATACTAATTAAAACAAAACTACCAGTCTTTAAAATCATTAATTATT
d_CM016580.1:55248210  : TGTGCCTATCATAAATAAAT-----AAAAAATT-AAAAAAAAAAAAGAAGTTGAATACTAATTAAAACAAAACTACCAGTCTTTAAAATCATTAATTATT
b_CM000012.4:54909836  : TGTGCCTATCATAAATAAAT-----AAAAAATT-AAAAAAAAAA---AAGTTGAATACTAATTAAAACAAAACTACCAGTCTTTGAAATCATTAATTATT
s_CM021976.1:36514210  : TGTGCCTATCATAAATAAAT-----AAAAAATT-AAAAAAAAAAAAAAAGAAAAAAAGAAAAAAAAAAA--AAAAAGTCTAAACTCCTAAGCTTTTCATC
b_CM000015.4:36603774  : TGTGCCTATCATAAATAAAT-----AAAAAATT-AAAAAAAAAAAAAAAGAAAAAAAAAAAAAAAAAAAAAAAAAAGTCTAAACTCCTAAGCTTTTCATC
s_CM021987.1:29177051  : TGTGCCTATCATAAATAAAT-----AAAAAATT-AAAAAAAAAACAATAAAAAGACACTTATACCAAATTGGAGAACAATAAGAAGGCTTGAGTAGACAT
b_CM000026.4:28974192  : TGTGCCTATCATAAATAAAT-----AAAAAATT-AAAAAAAAAACAATAAAAAGACACTTATACCAAATTGGAGAACAATAAGAAGGCTTGAGTAGACAT
s_CM021992.1:39506440  : TGTGCCTATCATAAATAAAT-----AAAAAATT-AAAAAAAAAAAAAAAAAAAGAAATGGGCAGAAGATGTGAGCAAACATATCTCCAGAGGAGACCCAC
d_CM016599.1:39027764  : TGTGCCTATCATAAATAAAT-----AAAAAATT-AAAAAAAAAAAAAAAA-AAGAAATGGGCAGAAGATGTGAGCAAACATATCTCCAGAGGAGACCCAC
b_CM000031.4:38752766  : TGTGCCTATCATAAATAAAT-----AAAAAATT-AAAAAAAAAAAAAAA--AAGAAATGGGCAGAAGATGTGAGCAAACATATCTCCAGAGGAGACCCAC
d_CM016588.1:3987471   : TGTGCCTATCATAAATAAAT-----AAAAAATT-AAAAAAAATTTAAAAAAAAAATACCATGGACTTTCTTCAGAGAGTTGGAACAAATCATCTTAAGAT
s_CM021995.1:14669264  : TGTGCCTATCATAAATAAAT-----AAAAAATT--AAAAAAAAAAAAAAAACTACGTTTCAGTGGGACATCTGTAATAGTTGGTGCTGTTATAACTAAAA
d_CM016602.1:14442100  : TGTGCCTATCATAAATAAAT-----AAAAAATT-AAAAAAAAAAAAAAAAACTACGTTTCAGTGGGACATCTGTAATAGTTGGTGCTGTTATAACTAAAA
b_CM000034.4:18577358  : TGTGCCTATCATAAATAAAT-----AAAAAATT-AAAAAAAAAAAAAAAAACTACGTTTCAGTGGGACATCTGTAATAGTTGGTGCTGTTATAACTAAAA
s_CM021983.1:15827289  : TGTGCCTATCATAAATAAAT-----AAAAAAAAAAAAAAAAAAAAAAAAAAAAAAATTAAAAAAAAAAAAAAAAAGAAAGTAAAAATATATCCCTGCT
b_CM000022.4:15829122  : TGTGCCTATCATAAATAAAT-----AAAAAAAAAAAAAAAAAAAAAAAAAAAAAAAAAAAAAAAAAAAAAAAAAAAGAAAGTAAAAATATATCCCTGCT
s_CM021974.1:52019303  : TGTGCCTATCATAAATAAAT-----AAAAAATT-AAAAAAAAATTTAGAAAGGGAGAAATTAATGTGGAAGCGCATAGAGATAGAAACTAGACATTTCTG
b_CM000013.4:51160355  : TGTGCCTATCATAAATAAAT-----AAAAAATTTAAAAAAAAATTTAGAAAGGGAGAAATTAATGTGGAAGCGCATAGAGATAGAAACTAGACATTTCTG
s_MU018711.1:230916    : TGTGCCTATCATAAATAAAT-----AAAAAATT-AAAAAAATTTTAAAAAAGTGAAGATGGAGATGATAGAGAAACTTAAAAGATATTCAGGCTATAGTT
s_CM021981.1:2716075   : TGTGCCTATCATAAATAAAT-----AAAAAATT-AAAAAAATAATAAATAAAATGTCTAGTTTTCAACAAGAAAATATGAGAACACTTCCCAATTCAATC
d_CM016588.1:2429172   : TGTGCCTATCATAAATAAAT-----AAAAAATT-AAAAAAATAATAAATAAAATGTCTAGTTTTCAACAAGAAAATATGAGAACACTTCCCAATTCAATC
b_CM000020.4:2724602   : TGTGCCTATCATAAATAAAT-----AAAAAAT--AAAAAAAAAATAAATAAAATGTCTAGTTTTCAACAAGAAAATATGAGAACACTTCCCAATTCAATC
s_CM021980.1:49263820  : TGTGCCTATCATAAATAAATAAAAAAAAAAATTAAAAAAAAAAAAAAAAAGGCAATGGTCCTCAGGCAAGGACAATGGTGTTTTCCAATCAAC
d_CM016587.1:47735003  : TGTGCCTATCATAAATAAATAAAAAAAAAAATTAAAAAAAAAAAAAAAAAGGCAATGGTCCTCAGGCAAGGACAATGGTGTTTTCCAATCAAC
s_CM021967.1:7694852   : TGTGCCTATCATAAATAAAT-----AAAAAATT-AAAAAAAAATAATTCCTTTATATCATTTAATACCAAGTCAATGTTTTATTTTTTATTTTTTAAAAG
d_CM016574.1:7567605   : TGTGCCTATCATAAATAAAT-----AAAAAATT-AAAAAAAAATAATTCCTTTATATCATTTAATACCAAGTCAATGTTTTATTTTTTATTTTTTAAAAG
s_CM021962.1:106324370 : TGTGCCTATCATAAATAAAT-AAAAAAAAAATTAAAAAAAATAAAATAAAATAAAAAGTAGAAAGAACCATGAGATCAATTTTAATGTAATGTAT
d_CM016569.1:105888100 : TGTGCCTATCATAAATAAAT-AAAAAAAAAATTAAAAAAAATAAAATAAAATAAAAAGTAGAAAGAACCATGAGATCAATTTTAATGTAATGTAT
b_CM000001.4:105279680 : TGTGCCTATCATAAATAAAT-AAAAAAAAAATTAAAAAAAATAAAATAAAATAAAAAGTAGAAAGAACCATGAGATCAATTTTAATGTAATGTAT
s_CM021982.1:34324034  : TGTGCCTATCATAAATAAAT-----AAAAAAAAAAAAAAAAAAAAAGAATTCAGGCTCTGGTTAATAGAATAGTTAACAACACTCCAAACCAATGTG
b_CM000021.4:33288465  : TGTGCCTATCATAAATAAAT-----AAAAAAAAAAAAAAAAAA-AAAGAATTCAGGCTCTGGTTAATAGAATAGTTAACAACACTCCAAACCAATGTG
s_CM021971.1:29008115  : TGTGCCTATCATAAATAAAT-----AAAAAATTT-AAAAAAAAAAAAAAAAAAAGAAGATCTCTCCAGGGGCAGCTGGGTGGCTCAGTCAGTTAAGCATG
d_CM016578.1:28521418  : TGTGCCTATCATAAATAAAT-----AAAAAATTT-AAAAAAAAAAAAAAAAAAAGAAGATCTCTCCAGGGGCAGCTGGGTGGCTCAGTCAGTTAAGCATG
b_CM000010.4:27962182  : TGTGCCTATCATAAATAAAT-----AAAAAATT-AAAAAAAAAAAAAAAA--AAGAAGATCTCTCCAGGGGCAGCTGGGTGGCTCAGTCAGTTAAGCATG
```

```
d_CM016581.1:36417555  : TGTGCCTATCATAAATAAAT-----AAAAAATT-AAAAAAAAAATTAAAAAAAAAAAAAAAAAAAAAAGGAAAAGGCCAGCTCGAGAGCCCTGGCCCGCAT
b_CM000014.4:26681805  : TGTGCCTATCATAAATAAAT-----AAAAAATT-AAAAAAAAAATGTAAATTGGTACACTGGGTGAGGTACAGAAGGTCCTCAATACAGGTGCTTCTGTG
s_CM021983.1:49473247  : TGTGCCTATCATAAATAAAT-----AAAAAATT-AAAAAAAAATTTTATTTTTTGAAATTATGGTAAAAAAAAAATCCCACATAATATAAATTTATCTAG
s_CM021969.1:53495922  : TGTGCCTATCATAAATAAAT-----AAAAAAAAAAAAAAAAAAAA-TTAAAGCTTCCTGTCCATTTTAGGTTACTTAACTAACCTAACTCTGGAAAAGAG
d_CM016576.1:53100186  : TGTGCCTATCATAAATAAAT-----AAAAAAAAAAAAAAAAAAAAAATTAAAGCTTCCTGTCCATTTTAGGTTACTTAACTAACCTAACTCTGGAAAAGAG
s_CM022000.1:124513739: TGTGCCTATCATAAATAAAT-----AAAAAATT-AAAAAAAATAAAATAAAATAAAATTTCCCATTCAGAATTAATAAACTTATAATTTTGAAGAAAAAT
d_CM016607.1:122259922: TGTGCCTATCATAAATAAAT-----AAAAAATT-AAAAAAAATAAAATAAAATAAAATTTCCCATTCAGAATTAATAAACTTATAATTTTGAAGAAAAAT
b_CM000039.4:108377960: TGTGCCTATCATAAATAAAT-----AAAAAATT-AAAAAAAATAAAATAAAATAAAATTTCCCATTCAGAATTAATAAACTTATAATTTTGAAGAAAAAT
d_CM016583.1:30685492  : TGTGCCTATCATAAATAAAT-----AAAAAATT-AAAAAAAAAAAAAAAATTAAAAAAAAAAAAAAAAAAAAATAGAAAGCTTCTTGATGCACATTTATCA
b_CM000015.4:31191229  : TGTGCCTATCATAAATAAAT-----AAAAAATT-AAAAAAAAAAAAAAAATTAAAAAAAAAAAAAAAAAA-AATAGAAAGCTTCTTGATGCACATTTATCA
s_CM021983.1:27220998  : TGTGCCTATCATAAATAAAT-----AAAAAATT-AAAAAAAAAAAAAAAAAAAAAAGCTAGTTTAAAAAACAATATTGGCAAAATGTTTGTCATTGTTACAA
d_CM016590.1:27110103  : TGTGCCTATCATAAATAAAT-----AAAAAATT-AAAAAAAAAAAAAAAA-AAAGCTAGTTTAAAAAACAATATTGGCAAAATGTTTGTCATTGTTACAA
b_CM000022.4:26972534  : TGTGCCTATCATAAATAAAT-----AAAAAATT-AAAAAAAAAAAAA----AAAACTAGTTTAAAAAACAATATTGGCAAAATGTTTGTCATTGTTACAA
s_CM021990.1:35056255  : TGTGCCTATCATAAATAAAT-----AAAAAATT-AAAAAAAAAATTTAAAGGAAAAATAATGAAGTGACTATTATGATAATTATTTCATATTATTCTAGA
b_CM000029.4:34574364  : TGTGCCTATCATAAATAAAT-----AAAAAATT-AAAAAAAAAATTTAAAGGAAAAATAATGAAGTGACTATTATGATAATTATTTCATATTATTCTAGA
b_CM000030.4:32702878  : TGTGCCTATCATAAATAAAT-----AAAAAATT-AAAAAAAAAAAAAAAAAAAAGACAAATAAAGGTGGGAGAAATACCCCAGCTGGAGAAGGACCAAAGG
s_CM021965.1:52147775  : TGTGCCTATCATAAATAAAT-----AAAAAATT-AAAAAAAAATTTTTTTAAAAAGGGAATGTTAAAAGTCTTGTCTACTTATAAATAGAATATTATTT
d_CM016572.1:51525463  : TGTGCCTATCATAAATAAAT-----AAAAAATT-AAAAAAAAATTTTTTTAAAAAGGGAATGTTAAAAGTCTTGTCTACTTATAAATAGAATATTATTT
b_CM000023.4:38752119  : TGTGCCTATCATAAATAAAT-----AAAAAATT-AAAAAAAAAAAAAAAAAAGAATATATTTTCAGTTTACTTGACCAGGGTTCAAAAATTCCTCAGATAG
s_CM021995.1:32404702  : TGTGCCTATCATAAATAAAT-----AAAAAATT-AAAAAAAATTAAAAAAAAAAAAAAACCCTGCAATGGTCAACTATTGCATTTAAAATGCAAATAAT
b_CM000034.4:41229417  : TGTGCCTATCATAAATAAAT-----AAAAAATT-AAAAAAATTAAAAAAAAAAAAAAACCCTGCAATGGTCAACTATTGCATTTAAAATGCAAATAAT
d_CM016602.1:32172425  : TGTGCCTATCATAAATAAAT-----AAAAAATT-AAAAAAAAATTTAAAAAAAAAAAAAAAACCTGCAATGGTCAACTATTGCATTTAAAATGCAAATAAT
s_CM021997.1:14667324  : TGTGCCTATCATAAATAAAT-AAAAAAAAAATTAAAAAAAAAAAAAAAATTAAAAAAAATTAAAAAAATAAAAATAAAATAAAAATAAAAATAAAAA
d_CM016604.1:14565027  : TGTGCCTATCATAAATAAAT-AAAAAAAAAATTAAAAAAAAAAAAAAAATTAAAAAAAATTAAAAAAATAAAAATAAAATAAAAATAAAAATAAAAA
s_CM021963.1:71894134  : TGTGCCTATCATAAATAAAT-----AAAAAATT-AAAAAAAATTTTTTTAAAATTAAATTAAATTAAATTTTAAAGATTTTATTTATTTATTCATGAGAC
s_CM021983.1:45943175  : TGTGCCTATCATAAATAAAT-----AAAAAATTAAAAAAAAAAAAAGAAACGTAGCAGGTTTGTAATATTTGATCTTAAATCCCCTGAAAACATAGGATT
s_CM021963.1:18318682  : TGTGCCTATCATAAAT---------AAAAAATT-AAAAAAAAAATTTAAAAAAAAAAAAAAAAAACAAAAGGACAAAATACATATGCCTTCCCTTTTCAAC
b_CM000002.4:16472404  : TGTGCCTATCATAAAT---------AAAAAATT-AAAAAAAAAATTAAAAAAAAAAAAAAAAAA--AAAAGGACAAAATACATATGCCTTCCCTTTTCAAC
s_CM021981.1:32911909  : TGTGCCTATCATAAATAAAT-----AAAAAATTT-AAAAAAAATGTATAAAAATGTGCCATAACCACAGCTGAATGCTATGTGAGCATTTTCACCTTGCT
d_CM016588.1:32382961  : TGTGCCTATCATAAATAAAT-----AAAAAATT-AAAAAAAAATGTATAAAAATGTGCCATAACCACAGCTGAATGCTATGTGAGCATTTTCACCTTGCT
b_CM000020.4:32582772  : TGTGCCTATCATAAATAAAT-----AAAAAATTT-AAAAAAAATGTATAAAAATGTGCCATAACCACAGCTGAATGCTATGTGAGCATTTTCACCTTGCT
s_CM021991.1:17604839  : TGTGCCTATCATAAATAAAT---TAAAAAAATT---AAAAAAAATAGAAAGGTAACAGTTTTGTGTCACTAACAGAACATGCCAATACAACTGTAAAGTA
d_CM016598.1:17380206  : TGTGCCTATCATAAATAAATTTAAAAAAAAATT-AAAAAAAAAATAGAAAGGTAACAGTTTTGTGTCACTAACAGAACATGCCAATACAACTGTAAAGTA
s_CM021972.1:23265552  : TGTGCCTATCATAAATAAAT------AAAAAAAAAAATTAGAACAAAATTGGGACCTTTAGCCAAGATGACAGTTGACACTAACTGGTTTTTGTTATTG
d_CM016579.1:22756425  : TGTGCCTATCATAAATAAAT-------AAAAAAAAAATTAGAACAAAATTGGGACCTTTAGCCAAGATGACAGTTGACACTAACTGGTTTTTGTTATTG
b_CM000011.4:22000418  : TGTGCCTATCATAAATAAAT-----AAAAAAAAAAAATTAGAACAAAATTGGGACCTTTAGCCAAGATGACAGTTGACACTAACTGGTTTTTGTTATTG
s_CM021988.1:9926312   : TGTGCCTATCATAAATAAAT-----AAAAAATT-AAAAAAAAAATTCCATTATATCTTAGGAATAACATAGACATGTTTCTCTCTCTCTCTTGTTCCCTC
b_CM000027.5:10401007  : TGTGCCTATCATAAATAAAT-----AAAAAATT-AAAAAAAAAATTCCATTATATCTTAGGAATAACATAGACATGTTTCTCTCTCTCTCTTGTTCTCTC
b_CM000008.4:72800272  : TGTGCCTATCATAAATAAAT-----AAAAAATT-AAAAAAAAAAATTTTAAAAAATAACATATATTACATATACATATATAATTTTTTAAATCAGGACTA
s_CM021970.1:18974382  : TGTGCCTATCATAAATAAAT-----AAAAAATT-AAAAAAAAAAAATTTTTTAAAAAAAGAAATTCAACATTGATGAAGCAGTATCTACTGTACAGGCCT
s_CM021998.1:30009360  : TGTGCCTATCATAAATAAATTTA--AAAAAATT--AAAAAAAATAAATAAAACATTATT--AAAAAAAATAGTAACTTCTACACAGTAGAACACATTGTT
d_CM016605.1:30057161  : TGTGCCTATCATAAATAAATTTA--AAAAAATT--AAAAAAAATAAATAAAACATTATT--AAAAAAAATAGTAACTTCTACACAGTAGAACACATTGTT
b_CM000037.4:30888146  : TGTGCCTATCATAAATAAATTTA--AAAAAATT-AAAAAAAAATAAATAAAACATTATTAAAAAAAAAATAGTAACTTCTACACAGTAGAACACATTGTT
b_CM000032.5:23742260  : TGTGCCTATCATAAATAAAT-----AAAAAATT-AAAAAAAAATTAAAAAACAAAAAAAAACTGACTCTTAGCTTAATTGGATGGGTTCCGAAAATCAAA
```

Figure S6. Members of the Can_b2 tribe 1 in the three dog breeds. The upper sequence "chr29 Can" (chr29:16532880-16533057, German Shepherd) was used as the search query. The letter prefix in sequence names indicates the genome where they are

present (s, German Shepherd; d, Great Dane; and b, Boxer). Sequence names are given in black if present in all three breeds, while those available in one or two genomes are given in blue and violet, respectively. Rudimentary terminators (TT and TTT) are marked green; TSDs are underlined. Notice that no copies contain a functional terminator ($T_{>3}$) in the proper place, which indicates that their retrotransposition proceeds without polyadenylation of pol III transcripts. Can copies from orthologous loci were not combined to demonstrate their sequence conservation and absence of terminators in either breed.
