# Supplementary material for: Tail Wags Dog’s SINE: Retropositional Mechanisms of Can SINE Depend on Its A-Tail Structure
Source: Biology (Basel). 2022 Sep 26;11(10):1403. doi: 10.3390/biology11101403 (PMC9599045; doi:10.3390/biology11101403)
Supplement: Supplementary file 1 [file biology-11-01403-s001.zip › Figure S7.pdf]

```
chr9:16208268-16208630 canFam4
AAAATAAATAAATAATAAGTTGCAAAACAAGAGTATAATTTAATTTCTGTAAAAAATACACATAAAAATCAGTAAGGTGGGATCCCTGGGTGGCGCAGTGGTTTAGCGCCTGCCTTTGGCCCAGGGCGCGATCCTGGAGACCCGGGATCGAATCCCAC
GTCAGGCTCCCGGTGCATGGAGCCTGCTTCTCCCTCTGCCTGTGTCTCTGCCTCTCTCTCTCTCTCTGTGACTATCATAAATAAATAAAAATTTAAAAAAAAATTTATAAAAAAAAAAAAAATCAGTAAGGTGTTTTCTATGCATAAATATGCATG
CAAGTGGATAAGTACAGTATTTAAAAATATAGATATATCTCAAACTT

s_chr9_16208268         : TGC-----------------CTCTCTCTCTCTCTCTCTGTGACTATCAT--AAATAAATAAAAA------TTTAAAAAAAAATTTATAAAAAAAAA-------------------------------------------
sdb_CM021968.1:45603386 : TGC-----------------CTCTCTCTCTCTCTCTCTGTGACTATCAT--AAATAAATAAAAA------TTTAAAAAAAAATTTAAAAAAAAAAAAAAAAAAAAAAAAAGAACCTTGTTTGACACCAGGGACTTGTTGCCT
sdb_CM021981.1:3038333  : TGC-----------------CTCTCTCTCTCTCTCTCTGTGACTATCAT--AAATAAATAAAAA------TTAAAAAAAAAATTAAAAAAAAAAAAAAAACATTAAAAAGTCTTAGAAAAAAGAGGGGGAGGGACACATGGG
sdb_CM021970.1:515646   : TGC---------------CTCTCTCTCTCTCTCTCTCTGTGACTATCAT--AAATAAATAAAAA------TTTTAAAAAAAAAAAAAAAAAAAGATTTTTTTAGGGATCCCTGGGTGGCGCAGTGGTTTGGCGCCTGCCTT
sdb_CM021987.1:7464676  : TGC-----------------CTCTCTCTCTCTCTCTCTGTGACTATCAT--AAATAAATAAAAA------TTAAAAAAAAAAATTAAAAAAAAAAAAAAAGAAAGAAAAAGAAAACCTGAAGAGTTTTAGGGATGCCTGGGT
sdb_CM022000.1:12060335 : TGC---------------CTCTCTCTCTCTCTCTCTCTGTGACTATCAT--AAATAAATAAAAA------TTTAAAAAAAATTAAAAAAAAATAAAATAAAACTTGAGCGATTTAAAGCATTAAAAAAAAAAGAAATGTTTG
sdb_CM022000.1:100119889: TGCCTCTCTCTCTCTCTCTCTCTCTCTCTCTCTCTGTGACTATCAT--AAATAAATAAAAA------TTTAAAAAAAAAATTTAAAAAAAAAAAAAAAGAATGTGAGAGTGGGCAACCCAGGTGGCTCAGCAGTTTAGTG
sdb_CM022000.1:123010400: TGC---------------CTCTCTCTCTCTCTCTCTCTGTGACTATCAT--AAATAAATAAAAA------TTTAAAAAAAGTTTAAAAAAAAAAAAAAAGAAGAGGAAAAAGGCTATGGGTGTCCGTAGGGAGGCCACCCTG
sdb_CM021970.1:57912045 : TGC-----------------CTCTCTCTCTCTCTCTCTGTGACTATCAT--AAATAAATAAAAA------TTTAAAAAAAAAATTTTTTTTAAATAAAAAAAAAATAAAAAAATTTTCCGATTTTAATTCTTTCAAAGAT
sdb_CM021997.1:5656951  : TGC-----------------CTCTCTCTCTCTCTCTCTGTGACTATCAT-AAAATAAATAAAAA------TTAAAAAAAAAAATAAAGCTCTTTAAAAAAAAAAAAAAAAAAAAAAAGAATAAATAAATAAAATATTTTTTA
sdb_CM021970.1:11548102 : TGC-----------------CTCTCTCTCTCTCTCTCTGTGACTATCAT--AAATAAATAAAAA------TTAAAAAAAAAAATTTAAAATGGATCAACAACCAAATATAAGAACTACAGCTATAAAACTAGGTATACTAG
sdb_CM021977.1:34528805 : TGC---------------CTCTCTCTCTCTCTCTCTCTGTGACTATCAT--AAATAAATAAAAA------TTTAAAAAAAAAAATAAATAAAAATCTATTGAGGGGATCCCTGGGCAGCTCAGCAGTTTAGCACCTGCCTT
sdb_CM021994.1:32001086 : TGC-----------------CTCTCTCTCTCTCTCTCTGTGACTATCAT--AAATAAATAAAAA------TTAAAAAAAAAATTTTTAAAAAAAAAATTAAAAAAATAAAATAAAATAAAATAAAAATAAAATTTCTTTGT
sdb_CM022000.1:121668107: TGC-------------------CTCTCTCTCTCTCTCTGTGACTATCAT--AAATAAATAAAAA------TTTAAAAATAAAAATAAAAAAAAATAAAAGAAAACCAAAGCTGGAGACATCACAATTCCTGACTTCAAGCT
sdb_CM021986.1:549424   : TGCCT--CTCTCTCTCTCTCTCTCTCTCTCTCTCTGTGACTATCAT--AAATAAATAAAAA------TTTAAAAAAATAAAAAAATAAAAATAAATAAAATAAAATAAAATTAATCACGGAGCTTGGGTGACTGAATT
sdb_CM022000.1:46315319 : TGC--------CTCTCTCTCTCTCTCTCTCTCTCTGTGACTATCAT--AAATAAATAAAAA------TTTAAAAAATTAAAAAAATAAATAAATAAATAAATAAAAAAAATAAAAATGTAATCCTTAGGGTGCCTGGG
sdb_CM021971.1:68568680 : TGC-----------------CTCTCTCTCTCTCTCTCTGTGACTATCAT--AAATAAATAAAAA------TTTAAAAAAAAATTAAAAAAAAAAAAGAAACATGAACTATGTGATACATTTATAAATATCTTAATCGAAGT
sdb_CM021977.1:16985747 : TGC-----------------CTCTCTCTCTCTCTCTCTGTGACTATCAT--AAATAAATAAAAA------TTAAAAAAAAAAAAAAATTTAAAAAAAAAGAAAGAAAAGGAAAGAAATACCTGTGAGAGTGAAATATAATT
sdb_CM021975.1:47499171 : TGC-----------------CTCTCTCTCTCTCTCTCTGTGACTATCAT--AAATAAATAAAAA------TTAAAAAAAAAATTTAAAAAAAAAAAAAAAAGAAACTTCACTAGAGAGGCTCAACAACATATCTGAGCTAGC
sdb_CM021967.1:39885994 : TGC------CTCTCTCTCTCTCTCTCTCTCTCTCTGTGACTATCAT--AAATAAATAAAAA------TTAAAAAAATAATTTATTTATAAATATTTATTTATTTATTTAATTTATTTATTTATTAATTATGATAGACAT
sdb_CM021962.1:65976109 : TGC-----------------CTCTCTCTCTCTCTCTCTGTGACTATCAT--AAATAAATAAAAA------TTAAAAAAAAAAAAATAATGGCTGAAAAGCTGTCAAATCTGAGGAGGAATTATGAATCCAAGATTAATGAATC
sdb_CM021981.1:40203621 : TGC-----------------CTCTCTCTCTCTCTCTCTGTGACTATCAT--AAATAAATAAAAA------TTAAAAAAATATATATATATTTTATTTATTTATTCATGAGAGACACACACAGAGAGGCAGAGGTATAGGCA
sdb_CM021970.1:6073219  : TGC---------------CTCTCTCTCTCTCTCTCTCTGTGACTATCAT--AAATAAATAAAAA------TTAAAAAAAAAATTTAAAATTTTTTAAATTATTTTTTATTTTTTATTAGAGAGAGAGAGAGACAGGCAGAG
sdb_CM021979.1:53347874 : TGC-----------------CTCTCTCTCTCTCTCTCTGTGACTATCAT--AAATAAATAAAAA------TTAAAAAAAAAAAAAAGATAAGTCTCCAGAAAGTAAAATAAGAAGACAACAAGCTTAGAAATGAGAAAGACA
sdb_CM021993.1:19891769 : TGC-----------------CTCTCTCTCTCTCTCTCTGTGACTATCAT--AAATAAATAAAAA------TTAAAAAAAAAATAATAAAAAAAAAAATAAAAAAATAAAACTTGAATACCAAAACAATCTTCTACCCCCAAT
sdb_CM021984.1:16210926 : TGC-----------------CTCTCTCTCTCTCTCTCTGTGACTATCAT--AAATAAATAAAAA------TTAAAAAAAAAAAAATTAAAAAAAAAAATAAAATAAAATTTCCTAATGACCTTGTGAGTTTTGTACCATTCC
sdb_CM021974.1:752618   : TGC-----------------CTCTCTCTCTCTCTCTCTGTGACTATCAT--AAATAAATAAAAA------TTAAAAAAAAAATTAAAAAAAAATAAATAAAAGGAGGATTCTTGTTGAGACTGAATTAGTAAGAATGGTTAA
sdb_CM021966.1:32118285 : TGC-----------------CTCTCTCTCTCTCTCTCTGTGACTATCAT--AAATAAATAAAAA------TTAAAAAAAAAAATTAAAAAAAAAAAAAAAGAAAAGACATGCGCACCCCTATGTTTATCATGGCATTATTTA
sdb_CM021974.1:14653741 : TGC-----------------CTCTCTCTCTCTCTCTCTGTGACTATCAT--AAATAAATAAAAA------TTAAAAAAAAAATTTATATAAAAAAAGAAAGAAATAAGGTCACATATATGTAGTGGTACATGTGTTTCATAC
sdb_CM021980.1:37790837 : TGC-----------------CTCTCTCTCTCTCTCTCTGTGACTATCAT--AAATAAATAAAAA------TTAAAAAAAAAAAAAGAAATAGTGGAAGCAACAAGTTCTCAAAATTCTGGTAAATGTAGGATTTTGATAGG
sdb_CM021984.1:3764406  : TGC---------------CTCTCTCTCTCTCTCTCTCTGTGACTATCAT--AAATAAATAAAAA------TTAAAAAAAAAAAAAAAAAAAACTACTAGGGTCATGTAAAAGGACTGAGGAGTCAAAGATAAGATCATTTGA
sdb_CM021972.1:1300139  : TGC-----------------CTCTCTCTCTCTCTCTCTGTGACTATCAT--AAATAAATAAAAA------TTAAAAAAAAAAATTAAAAAAATATATATAAAAATTTGTATAAATCAGGTATGTCTTCTTAAAGAGGCAATA
sdb_CM021969.1:4452852  : TGC-------------CTCTCTCTCTCTCTCTCTCTCTGTGACTATCAT-AAAATAAATAAAAA------TTAAAAAAAAAATTAAAAAAAAATAAATAAAATGTCAACTATAATAGGATATTAGAGTACTGAGAGATTGGC
sdb_CM021983.1:41009587 : TGC-----------------CTCTCTCTCTCTCTCTCTGTGACTATCAT-AAAATAAATAAAAA------TTAAAAAAAAAAATAAAAACATTAGGAATGAATATATATAGAAAAATACCTAAAATACTCAATTAGGAACAA
sdb_CM021981.1:43172451 : TGC-----------------CTCTCTCTCTCTCTCTCTGTGACTATCAT-AAAATAAATAAAAA------TTAAAAAAAAAATTTTATAAAAAAAAAAAAAAAGTAGTTTGTTTTTAAGTCCCATCTCATTGATGTGTAAG
sdb_CM021977.1:22530433 : TGC-----------------CTCTCTCTCTCTCTCTCTGTGACTATCAT--AAATAAATAAAAA------TTTTAAAAAAAATTTTTTTAAATTGACATTAAGGTACAGCCCATCAAATCAGGTATCTGACAAAAATATT
sdb_CM021964.1:73434505 : TGC---------------CTCTCTCTCTCTCTCTCTCTGTGACTATCAT--AAATAAATAAAAA------TTAAAAAAAAAAAATTTTAAATCTCTTGTGTCCAGAGACATTAAGAAGCTGCTGTATTGTGGGCAAGACAGA
sdb_CM021990.1:40080157 : TGC---------------CTCTCTCTCTCTCTCTCTCTGTGACTATCAT--AAATAAATAAAAA------TTAAAAAAAAAAAAAGATACATTTTATTTTCCACTTAATTGAAAATGTCATTGTTACCCATTTAACTAACA
sdb_CM021971.1:12537937 : TGC---------------CTCTCTCTCTCTCTCTCTCTGTGACTATCAT--AAATAAATAAAAA------TTAAAAAAAAAAAAAAAAAGATTAATCCTCCAAATTTTAGGAAGTTGCCAACTATCTTATACTTACTAGAAA
sdb_CM021968.1:3092017  : TGC---------------CTCTCTCTCTCTCTCTCTCTGTGACTATCAT--AAATAAATAAAAA------TTTTAAAAAAATTAAAAAAAAAATTCAATGATTTAGGTGGAAATGATCAATTCCTTGAAAGTCAAAAACTAC
sdb_CM021982.1:46772267 : TGC---------------CTCTCTCTCTCTCTCTCTCTGTGACTATCAT--AAATAAATAAAAA------TTAAAAAAAATCTTTAAAAAAAAAAAAAAAAAAAAAAGATTTGACACTGGCCTCTGAATTTCAAGCAGTTTTT
sdb_CM021965.1:73467710 : TGC---------------CTCTCTCTCTCTCTCTCTCTGTGACTATCAT--AAATAAATAAAAA------TTAAAAAAAAATAAAATAAAAAAATAAAAAAATCTCCAAATTCATCACCTTTGATGAAAAACAAGTCTGAG
sdb_CM021986.1:18220342 : TGC---------------CTCTCTCTCTCTCTCTCTCTGTGACTATCAT--AAATAAATAAAAA------TTAAAAAAAATTAAAAAAAAAAAATGAAGAGATAATCACGTAAAAGAATGAGTATAAGCAATGAAGCATAAC
sdb_CM021992.1:36961385 : TGC---------------CTCTCTCTCTCTCTCTCTGTGTGACTATCAT--AAATAAATAAAAA------TTTAAAAAAATTTAAAAAAAAAAAAAAAAGAAGCAGACCCCCAAGTGATGAACATGCTGGAAATAGCAGAA
```

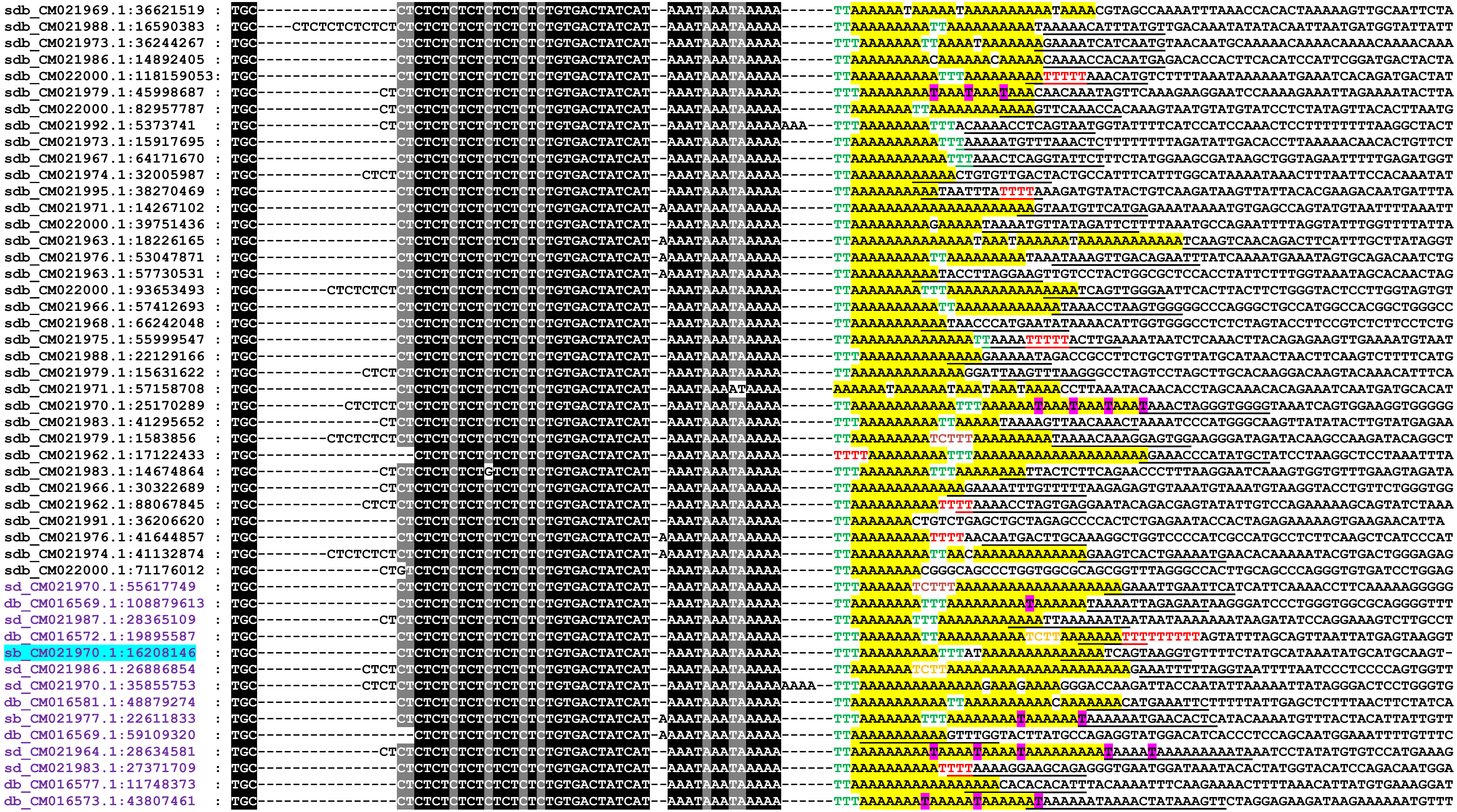

sdb_CM021969.1:36621519
sdb_CM021988.1:16590383
sdb_CM021973.1:36244267
sdb_CM021986.1:14892405
sdb_CM022000.1:118159053
sdb_CM021979.1:45998687
sdb_CM022000.1:82957787
sdb_CM021992.1:5373741
sdb_CM021973.1:15917695
sdb_CM021967.1:64171670
sdb_CM021974.1:32005987
sdb_CM021995.1:38270469
sdb_CM021971.1:14267102
sdb_CM022000.1:39751436
sdb_CM021963.1:18226165
sdb_CM021976.1:53047871
sdb_CM021963.1:57730531
sdb_CM022000.1:93653493
sdb_CM021966.1:57412693
sdb_CM021968.1:66242048
sdb_CM021975.1:55999547
sdb_CM021988.1:22129166
sdb_CM021979.1:15631622
sdb_CM021971.1:57158708
sdb_CM021970.1:25170289
sdb_CM021983.1:41295652
sdb_CM021979.1:1583856
sdb_CM021962.1:17122433
sdb_CM021983.1:14674864
sdb_CM021966.1:30322689
sdb_CM021962.1:88067845
sdb_CM021991.1:36206620
sdb_CM021976.1:41644857
sdb_CM021974.1:41132874
sdb_CM022000.1:71176012
sd_CM021970.1:55617749
db_CM016569.1:108879613
sd_CM021987.1:28365109
db_CM016572.1:19895587
sb_CM021970.1:16208146
sd_CM021986.1:26886854
sd_CM021970.1:35855753
db_CM016581.1:48879274
sb_CM021977.1:22611833
db_CM016569.1:59109320
sd_CM021964.1:28634581
sd_CM021983.1:27371709
db_CM016577.1:11748373
db_CM016573.1:43807461

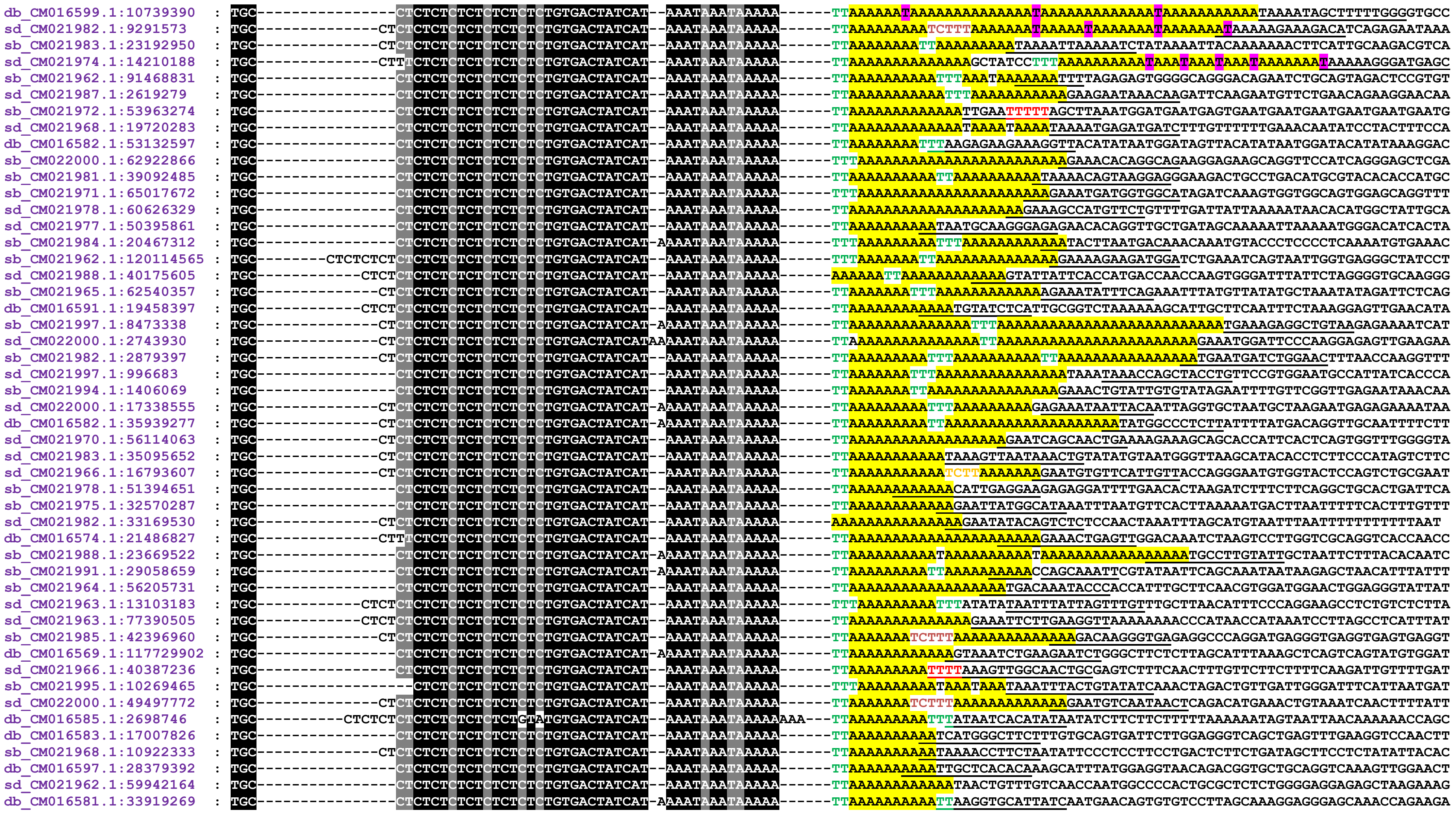
db_CM016599.1:10739390
sd_CM021982.1:9291573
sb_CM021983.1:23192950
sd_CM021974.1:14210188
sb_CM021962.1:91468831
sd_CM021987.1:2619279
sb_CM021972.1:53963274
sd_CM021968.1:19720283
db_CM016582.1:53132597
sb_CM022000.1:62922866
sb_CM021981.1:39092485
sb_CM021971.1:65017672
sd_CM021978.1:60626329
sd_CM021977.1:50395861
sb_CM021984.1:20467312
sb_CM021962.1:120114565
sd_CM021988.1:40175605
sb_CM021965.1:62540357
db_CM016591.1:19458397
sb_CM021997.1:8473338
sd_CM022000.1:2743930
sb_CM021982.1:2879397
sd_CM021997.1:996683
sb_CM021994.1:1406069
sd_CM022000.1:17338555
db_CM016582.1:35939277
sd_CM021970.1:56114063
sd_CM021983.1:35095652
sd_CM021966.1:16793607
sb_CM021978.1:51394651
sb_CM021975.1:32570287
sd_CM021982.1:33169530
db_CM016574.1:21486827
sb_CM021988.1:23669522
sb_CM021991.1:29058659
sb_CM021964.1:56205731
sd_CM021963.1:13103183
sd_CM021963.1:77390505
sb_CM021985.1:42396960
db_CM016569.1:117729902
sd_CM021966.1:40387236
sb_CM021995.1:10269465
sd_CM022000.1:49497772
db_CM016585.1:2698746
db_CM016583.1:17007826
sb_CM021968.1:10922333
db_CM016597.1:28379392
sd_CM021962.1:59942164
db_CM016581.1:33919269

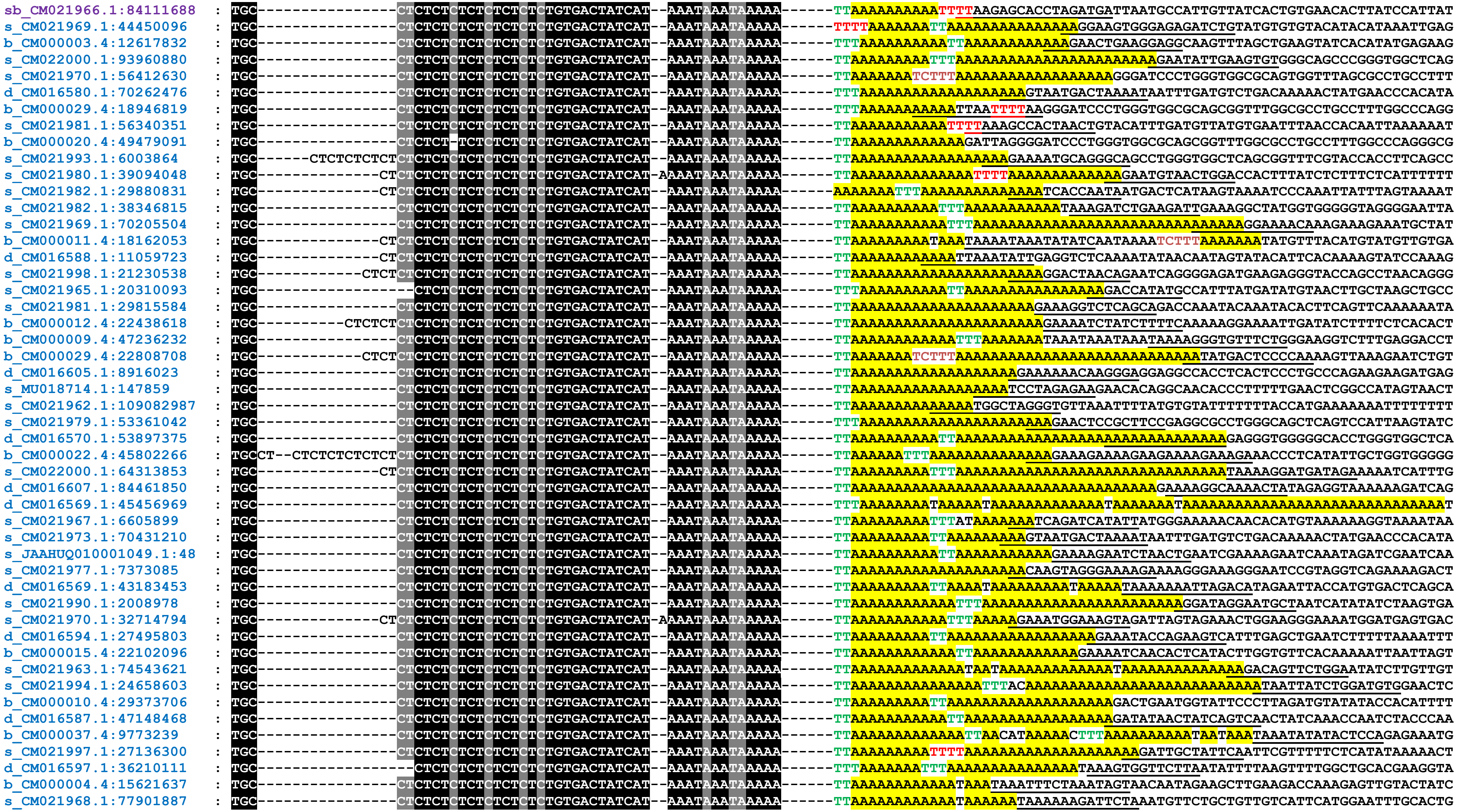
sb_CM021966.1:84111688
s_CM021969.1:44450096
b_CM000003.4:12617832
s_CM022000.1:93960880
s_CM021970.1:56412630
d_CM016580.1:70262476
b_CM000029.4:18946819
s_CM021981.1:56340351
b_CM000020.4:49479091
s_CM021993.1:6003864
s_CM021980.1:39094048
s_CM021982.1:29880831
s_CM021982.1:38346815
s_CM021969.1:70205504
b_CM000011.4:18162053
d_CM016588.1:11059723
s_CM021998.1:21230538
s_CM021965.1:20310093
s_CM021981.1:29815584
b_CM000012.4:22438618
b_CM000009.4:47236232
b_CM000029.4:22808708
d_CM016605.1:8916023
s_MU018714.1:147859
s_CM021962.1:109082987
s_CM021979.1:53361042
d_CM016570.1:53897375
b_CM000022.4:45802266
s_CM022000.1:64313853
d_CM016607.1:84461850
d_CM016569.1:45456969
s_CM021967.1:6605899
s_CM021973.1:70431210
s_JAAHUQ010001049.1:48
s_CM021977.1:7373085
d_CM016569.1:43183453
s_CM021990.1:2008978
s_CM021970.1:32714794
d_CM016594.1:27495803
b_CM000015.4:22102096
s_CM021963.1:74543621
s_CM021994.1:24658603
b_CM000010.4:29373706
d_CM016587.1:47148468
b_CM000037.4:9773239
s_CM021997.1:27136300
d_CM016597.1:36210111
b_CM000004.4:15621637
s_CM021968.1:77901887

```
s_CM021985.1:12979734   : TGC----------------CTCTCTCTCTCTCTCTCTGTGACTATCAT--AAATAAATAAAAA------TTAAAAAAAATTAAAAAAAAAAAAAAAAGAATTAAATGGTAAAATGTATGTAATGCATTTATTATGGTACA
s_CM021986.1:3551925    : TGC--------------CTCTCTCTCTCTCTCTCTCTGTGACTATCAT--AAATAAATAAAAA------TTAAAAAAAAATTAAAAAAAAAAAAAAAAATTGAAGTGGCAATCAAAACCATGCCTTCCTAAAAACCTAGAG
s_CM021964.1:90067882   : TGC----------------CTCTCTCTCTCTCTCTCTGTGACTATCAT--AAATAAATAAAAA------TTAAAAAAAAATTAAAAAAAAAATGTGAAGAGAGACACAAACACATTTTATGGTGTACCTGGGTACTCGGC
s_CM021963.1:53507754   : TGC----------------CTCTCTCTCTCTCTCTCTGTGACTATCAT--AAATAAATAAAAA------TTAAAAAAAAAAAAAAAAAAAAAAAAAAAAGACAGTGTGGTACTGGCACAAAAACAGACACATAGATCAATG
s_CM021999.1:22451582   : TGC----------------CTCTCTCTCTCTCTCTCTGTGACTATCAT--AAATAAATAAAAA------TTAAAAAAAAAAATTTAAAAAAAAAAAAAGGAAGTGCTCACTCGTGGCACATATACTAAAAATAGAACGAT
b_CM000028.4:13319145   : TGC----------------CTCTCTCTCTCTCTCTCTGTGACTATCAT--AAATAAATAAAAA------TTTAAAAAAAAAAAAAAAAAAGAAAAGGCTATTTTTGCAATGATTTAGTTACCACAAGAATCAGGAAGAAA
b_CM000036.4:28374235   : TGC----------------CTCTCTCTCTCTCTCTCTGTGACTATCAT--AAATAAATAAAAA------TTTAAAAAAAAAAAATCAAAACCACAATGAGATCCCACCTCACACCAGTGAGAATGGGGAAAATTAACAAG
d_CM016571.1:72545224   : TGC----------------CTCTCTCTCTCTCTCTCTGTGACTATCAT--AAATAAATAAAAA------TTAAAAAAAAATTAAAAAAAAAAAAAAAAAAAAAAGTCCCCTTAATGAAGTTGATGCTTTGAAAAGCTACT
s_CM021976.1:43588485   : TGC----------------CTCTCTCTCTCTCTCTCTGTGACTATCAT--AAATAAATAAAAA------TTAAAAAAATCTTAAAAAAAAAAAGAATGCAGTTAAAGTGAATATGGGGCCCTTATTAAAAGCATATTGGG
s_CM021966.1:22526754   : TGC----------CTCTCTCTCTCTCTCTCTCTCTCTGTGACTATCAT--AAATAAATAAAAA------TTAAAAAAAAAAAAAAAAAAAAGAAAATAAGTCCCAATATATCCAAAGTAAATGGATTAAATACACCAGTG
d_CM016581.1:41697618   : TGC------------CTCTCTCTCTCTCTCTCTCTCTGTGACTATCAT--AAATAAATAAAAAAA----TTTAAAAAAAAAAAAAAAAGAAATAAATGAGATAAAGATGAAAAGATAATGAAACCAAGAACTCATAGTTG
d_CM016597.1:319731     : TGC------------CTCTCTCTCTCTCTCTCTCTCTGTGACTATCAT--AAATAAATAAAAAAAAAAATTAAAAAAAAAAAAAAAAGAAATAAAAGGCAATTAAACTGATAAAGAGGAAGCAAAACTTTCACTATTTGCA
d_CM016570.1:52644359   : TGC----------CTCTCTCTCTCTCTCTCTCTCTCTGTGACTATCAT--AAATAAATAAAAA------TTAAAAAAAAATGATCAAGTGGGATTTATTCCCAGGATACAGAGTGGTTCAAAATTCACAAATCAATGTG
s_CM021978.1:33573116   : TGC----------------CTCTCTCTCTCTCTCTCTGTGACTATCAT--AAATAAATAAAAA------TTTAAAAAAAAAAAAAAAAAAAAACAAAGGCCATATACAAAAGACCCACAGCTAATATTATCCTGAAGGGGG
d_CM016569.1:46456947   : TGC----------------CTCTCTCTCTCTCTCTCTGTGACTATCAT--AAATAAATAAAAA------TTAAAAAAAAAAAAAAAAAAAAAAAAAGATATAAACCAGAGGGAAGCAAACTATGGCCTGCAGACCAAATC
b_CM000025.4:23351496   : TGC--------------CTCTCTCTCTCTCTCTCTCTGTGACTATCAT--AAATAAATAAAAA------TTAAAAAAAATAAATAAATAAAGATTTACTATCTATTCTTCTCAAACTATTCCAAAAAGTAGGAAAGGAG
s_CM021978.1:55925182   : TGC----------------CTCTCTCTCTCTCTCTCTGTGACTATCAT-AAAATAAATAAAAA------TAAAAAAAAAATAAAGCTCTTTAAAAAAAAATAAAATAAAATAAAAGATTAAATGATCTAACATGTGTAAA
s_CM021982.1:33964332   : TGC--------------CTCTCTCTCTCTCTCTCTCTGTGACTATCAT-AAAATAAATAAAAA------TTAAAAAAAAAAAAAAAAAAAAAAAAAAAAAAAAAAAAAAAAAAGTCATTAATATTGCCAACATACAAAATT
s_CM021986.1:17205820   : TGC--------------CTCTCTCTCTCTCTCTCTCTGTGACTATCAT-AAAATAAATAAAAA------TTAAAAAAAAAAAAAAAAAAAAAAAAAAAAAAAAAAAAAAAAAAAAAAAAAAAAAAGTCGGACACTTAACTGA
s_CM021979.1:28963445   : TGC----------------CTCTCTCTCTCTCTCTCTGTGACTATCAT--AAATAAATAAAAA------TTAAAAAAAAAATTAAAAAAAAAAAATAAAACCTTACTATATTCTATTACCAAATGTAAAGAAATGAAATC
s_CM021988.1:31624815   : TGC----------------CTCTCTCTCTCTCTCTCTGTGACTATCAT--AAATAAATAAAAA------TTAAAAAAATTTAAAAAAAAATAAAATAAAATAAAATAATGAATAAATCAATATACAGATGGGAAGAACAT
d_CM016580.1:51905502   : TGC--------------CTCTCTCTCTCTCTCTCTCTGGTGACTATCAT--AAATAAATAAAAA------TTAAAAAAAAATTTATCAAGGCTTTATGTATGATCTAGTACATGGTTTTTGTGGGGTTTTTTTTGCTATTA
s_CM021992.1:4968148    : TGC--------------CTCTCTCTCTCTCTCTCTCTGTGACTATCAT--AAATAAATAAAAA------TTAAAAAAAATTAAAAAAAAAAAAATAACTTCTGATGTGATATAATAGAAAATATACATTGATCTCTGCTC
s_CM021967.1:62283347   : TGC--------------CTCTCTCTCTCTCTCTCTCTGTGACTATCAT--AAATAAATAAAAAAAA---TTCAAAAAAAAATTATAAAAAAAAAAAAAAAAATACATGTCTCTGAATTATTAATAGAATATAAAATGTAA
b_CM000029.4:7539689    : TGC--------------CTCTCTCTCTCTCTCTCTCTGTGACTATCAT--AAATAAATAAAAA------TTAAAAAAAAAAAAAAAAAAGAATCAAAACCAATGACTGGTTGGGTGGCCTTAACAAGTTACGTAACTTCC
s_CM021993.1:26241987   : TGC----------------CTCTCTCTCTCTCTCTCTGTGACTATCAT--AAATAAATAAAAA------TTTAAAAAAAAAATTAAAAAATAAATAAATAAATAAATAAATAAATAAAATTCATGCTCTCTGATCCCAAG
s_CM021993.1:16362063   : TGC----------------CTCTCTCTCTCTCTCTCTGTGACTATCAT--AAATAAATAAAAA------TTAAAAAAAAAAAAAAAAAAGAGTGAGTTTCAGTAGTTGATGATGGATGTGACAGTTCTGATGTGGTACAG
b_CM000022.4:7528504    : TGC----------------CTCTCTCTCTCTCTCTCTGTGACTATCAT--AAATAAATAAAAA------TTAAAAAAAAAATAAATAAAAAAAAATAAAGTTACCTAGGAGAATCTGTGATAAAATTCACATCAATTGGG
s_CM021962.1:28666279   : TGC------CTCTCTCTCTCTCTCTCTCTCTCTCTCTGTGACTATCAT--AAATAAATAAAAA------TTAAAAAAAAATTAAAAAAAAAACATTTAAAAAAAAATTTCATCTATCATTTTTAGGTTGTTTTTCCAA
s_JAAHUQ010001506.      : TGC----------------CTCTCTCTCTCTCTCTCTGTGACTATCAT--AAATAAATAAAAA------TTAAAAAAAAATTTAAAAAAAAAAAAAATAAATAAAATGAGTCTCTTGTAGACAGCAAATAGATGGGTCCT
d_CM016595.1:37131982   : TGC----------------CTCTCTCTCTCTCTCTCTGTGACTATCAT--AAATAAATAAAAA------TTAAAAAAAAATAAATAAATATAAAATATATTTTTAAAAGGATCAAAAAAGTTGGGATACTACAAATAAAG
s_CM021966.1:78813649   : TGC--------------TTCTCTCTCTCTCTCTCTCTGTGACTATCAT--AAATAAATAAAAA------TTAAAAAAATTAAAAAAAAAATAATGACAACTTCTACTTTGTAGGGTTGTTGTGAATGAATAAAATGTCAC
b_CM000002.4:9755893    : TGC--------------CTCTCTCTCTCTCTCTCTCTGTGACTATCAT--AAATAAATAAAAA------TTAAAAAAAAAAAAAAAAAAGAATCCTATATTACCATAATGATGATGTGTAAATCATTTTCAAGTCAGGTA
s_CM021966.1:76956477   : TGC--------------CTCTCTCTCTCTCTCTCTCTGTGACTATCAT--AAATAAATAAAAA------TTTAAAAAAAAAAATAAAAAAATAAAATAAAATTTTTTTTTTAAGCAAGAGAGCACAAGTAGGAGGGGCAGA
s_CM022000.1:19572129   : TGC------------CTCTCTCTCTCTCTCTCTCTCTGTGACTATCAT--AAATAAATAAAAA------TTAAAAAAAAAAAAAAAAAAAAAAAAAAAAAAACAAAGACCTGGTGTTCTTGAAAACAATCTGGGGAGGAG
b_CM000003.4:15826478   : TGC------------------CTCTCTCTCTCTCTCTGTGACTATCAT--AAATAAATAAAAA------TTTAAAAAAAAAAAAAAAAGCTTAAATAGTTCACACTACTTTTGGAAGTATTTCCCATATTAATGTATTTCA
s_CM021999.1:24084567   : TGC--------------CTCTCTCTCTCTCTCTCTCTGTGACTATCAT--AAATAAATAAAAA------TTAAAAAAAAAAAAAAAAAATTTAAGTTCATAAAAATGAAGTCAAACATCATATTCATTTTTGTATCTTGGTT
d_CM016573.1:935476     : TGC----------------CTCTCTCTCTCTCTCTCTGTGACTATCAT--AAATAAATAAAAA------TTAAAAAAAAAAAAAAAGAAACCCTTCCTTTTTTTTTTTTTTTTTAATTTGTAACATTTTAATTCTGGTCATT
b_CM000005.4:13770141   : TGC----------------CTCTCTCTCTCTCTCTCTGTGACTATCAT--AAATAAATAAAAA------TTAAAAAAAAAAAAAAAAAGATTTTAAATCAGAGGACTTGAGTTCAGATCCTAGTTCTCCTACTAGCCAGC
d_CM016595.1:34377988   : TGC--------CTCTCTCTCTCTCTCTCTCTCTCTCTGTGACTATCAT--AAATAAATAAAAA------TTAAAAAAAAAAAAAAGAAAATTTCCTCTCAAAGCAGTCACTGATTGCTTTTCTGAAATTGATACTGTTCT
s_CM021980.1:55487704   : TGC--------------CTCTCTCTCTCTCTCTCTCTGTGACTATCAT-AAAATAAATAAAAA------TTAAAAAAAATAAAAAAATAAAAAAAATAAATCTAATATAAATGATAGCTAGATTCTAAGATAGTTAACCA
d_REHQ01000435.1:20479  : TGC----------------CTCTCTCTCTCTCTCTCTGTGACTATCAT--AAAT-AATAAAAA------TTAAAAAAAAAAAAATTTAAAAAAAAGAAACTTAACCACCATGAAAACTAAACCCTGACATTGCCAGTCAC
s_CM021995.1:11935052   : TGC--------------CTCTCTCTCTCTCTCTCTCTGTGACTATCAT--AAATAAATAAAAA------TTAAAAAAAAAAAAATTAAAAAAAAATAAATAAAAAAAACTAAGTACTGCAAACTGGTCAGCTTAAAACAA
s_CM021986.1:27978682   : TGC----------------CTCTCTCTCTCTCTCTCTGTGACTATCAT-AAAATAAATAAAAA------TTAAAAAAAAAATAAAACCAAATAGTATTAAACTCAAACAAAACTCACTATATTTATGTAAGTCACTTTAA
s_CM021997.1:16547481   : TGC------------CTCTCTCTCTCTCTCTCTCTCTGTGACTATCAT--AAATAAATAAAAA------AAAAAAAAAAAAAAAAAAAAAAAGTGGTAAGAGTTGGCACCCCTGTCTTTTCCTGATCGTAGAGCAAAAACA
s_CM021992.1:24843655   : TGC----------------CTCTCTCTCTCTCTCTCTGTGACTATCAT-AAAATAAATAAAAA------TTAAAAAAAAAAAAACTATCAATAATTGGATTCATGTATAATTTTTTTTTTAGAAAAACTTTTTCCTAAAA
d_CM016589.1:17347947   : TGC----------------CTCTCTCTCTCTCTCTCTGTGACTATCAT-AAAATAAATAAAAA------TTAAAAAAAATAAATAAATAAAGTAAGGTTAATCCCTGTCCTAAACACCAATTTTTCTCACTTAATCCTCA
d_CM016596.1:33879188   : TGC----------------CTCTCTCTCTCTCTCTCTGTGACTATCAT--AAATAAATAAAAA------TTAAAAAAAAAAAAAAATTAAAAAAAAAAATAATAATAATAATAAAAAGATAAAAATGAAAGTTCCTCAG
s_CM021968.1:41346073   : TGC----------------CTCTCTCTCTCTCTCTCTGTGACTATCAT--AAATAAATAAAAA------TTAAAAAAATCTTTAAAAAAAAAAAAAAAAAAAAAAAAAAAAAAAAAGCAGACTGAGAACCTACTATGGAC
```

```
s_CM021999.1:1302320    : TGC----------CTCTCTCTCTCTCTCTCTCTCTCTCTGTGACTATCAT--AAATAAATAAAAA------TTTAAAAAAAAAAAAAAAAAAAAAAAAATCCATCAGGTAACTTCCAAACACTGGACTCACACATGATCCTC
d_CM016585.1:39244977   : TGC----------------CTCTCTCTCTCTCTCTCTGTGACTATCAT--AAATAAATAAAAA------TTAAAAAAAAAAAATTTGAAGGTGGTACTAAACTGCTGAGCCACCCGGGCTGCCCATGTTTGGATGTTCTT
s_CM021974.1:2158052    : TGC------------------CTCTCTCTCTCTCTCTGTGACTATCAT-AAAATAAATAAAAA------TTAAAAAAAAAAAAAAAACAAGTATAAAAAAAAAAAATAAAAAAAAAAAAAAACAGAAGGAACTGTATTAG
s_JAAHUQ010001567.1     : TGC----------------CTCTCTCTCTCTCTCTCTGTGACTATCAT--AAATAAATAAAAA------TTAAAAAAAAAAATTTAAAAAAAAAAAAAAAAAAAAAAAAAAACAAGTCCAGGTGAGACCCAGGTGGCGAC
s_CM021992.1:33557050   : TGC----------------CTCTCTCTCTCTCTCTCTGTGACTATCAT--AAATAAATAAAAA------TTAAAAAAAAAATTTAAAAAAAAAAAAAAAAAAAGAAAAGGAACTTGCTATTTTCCGTTGCCAAGCTGTGT
s_CM021964.1:30379999   : TGC----------------CTCTCTCTCTCTCTCTCTGTGACTATCAT--AAATAAATAAAAA------TTAAAAAAAAAAAGTCAAAAAAAAGAAAACACGGTGTGAGCCACTTTCCTACCAAACTGGGACGCCAGTCA
s_CM021983.1:39843791   : TGC------CTCTCTCTCTCTCTCTCTCTCTCTCTCTGTGACTATCAT--AAATAAATAAAAA------TTAAAAAAAAAAAATGAATTGTTAAGAGGATTGAAAATACCTTTAATCTGGAATTTTTATTTCCCATAAAA
d_CM016602.1:37922450   : TGC----------------CTCTCTCTCTCTCTCTCTGTGACTATCAT--AAATAAATAAAAA------TTAAAAAAAAAAAAAAAAAGTCCAGATACTAAATATGTTGGTAGCTTTGAGGGCCACGGGGTCTCTTTTCA
s_CM021975.1:44948071   : TGC----------------CTCTCTCTCTCTCTCTCTGTGACTATCAT--AAATAAATAAAAA------TTAAAAAAAAAAAAAAAAAAGGAAGGCAAGGGGAGAGTCAGGCCTTCTCTTTAAAAAAAAAAAAAAAAAAA
b_CM000015.4:12045115   : TGC----------CTCTCTCTCTCTCTCTCTCTCTCTCTGTGACTATCAT--AAATAAATAAAAA----AATTAAAAAAATTTAAAAAAAAAAAAAAAAAAAAAAAAACTTTTTTCCTTACTTTTAAGGATGTGTCTGAGAA
d_CM016593.1:25664656   : TGC----------CTCTCTCTCTCTCTCTCTCTCTCTCTGTGACTATCAT--AAATAAATAAAAA------AAAAATAAAATAAAAAAAAATAGAACTTCATAGCTAGATAGAAGAAGTCATTGCCTAGCTTCAAAGCTTTG
s_CM021979.1:3998940    : TGC----------CTCTCTCTCTCTCTCTCTCTCTCTCTGTGACTATCAT--AAATAAATAAAAA------TTAAAAAAAAATAAAAATAAAAAAATAAAACAGGGAAGCAGACACACAATGAGAAAAGGAACTTCAGGGGA
d_CM016595.1:17341917   : TGC--------------CTCTCTCTCTCTCTCTCTCTGTGACTATCAT--AAATAAATAAAAA------TTAAAAAAAAAAAATTTAAAAAGGGGGAGGAGGGAAGCTTGAGGTTACTAATTTAAGTAAGAAGAAAGGG
b_CM000008.4:45177897   : TGC----------------CTCTCTCTCTCTCTCTCTGTGACTATCAT--AAATAAATAAAAA------TTAAAAAAAAAAAGCCACCCAGTCTATGATATAGTTCCTCTTCTTCTATGAGGGAATCTTTGGAGTTCTTG
s_CM021968.1:70022581   : TGC----------------CTCTCTCTCTCTCTCTCTGTGACTATCAT--AAATAAATAAAAA------TTTAAAAAAAAAAAATATGACATCTGGTGTGGTCTTGTACATTTTGGGTAGTTTTTCCCGAATTTCTGTCT
b_CM000026.4:29896458   : TGC--------------CTCTCTCTCTCTCTCTCTCTGTGACTATCAT--AAATAAATAAAAA------AAAAAAAAAAGAAAGAAAGCCAATTCAGAAGCACTATTATACAGCTACTGGTGGCTCTAGAAAAAAGCATA
b_CM000037.4:499477     : TGC----------CTCTCTCTCTCTCTCTCTCTCTCTCTGTGACTATCAT--AAATAAATAAAAA------TTAAAAAAATCTTTAAAAAAAAAAAAAACAATTCAATGTCAGAAAGCCAATTCAGAAGCATTATTATACAG
b_CM000004.4:83154346   : TGC----------------CTCTCTCTCTCTCTCTCTGTGACTATCAT--AAATAAATAAAAA------TTTAAAAAAAAAAAAAAAAATTCCCCTGGCTATTCGGGGTCTTTTCTGATTCCACACAAATCTTAAAATAA
s_CM021966.1:24511004   : TGC----------CTCTCTCTCTCTCTCTCTCTCTCTCTGTGACTATCAT--AAATAAATAAAAA------TTAAAAAAATTTAAAAAAAAAAAATATAATATCTGACTACTAATTATGTGTCAGGTACTATTTTAAATATT
s_CM021985.1:13731355   : TGC----------CTCTCTCTCTCTCTCTCTCTGTGTGTGACTATCAT--AAATAAATAAAAA------TTAAAAAAAAATTTTAAAAAAATAAAACAATTCTCCTGTATCATTTACCACCAGCAATTTTTTATAGTTCA
d_CM016584.1:49524041   : TGC----------------CTCTCTCTCTCTCTCTCTGTGACTATCAT--AAATAAATAAAAA------TTAAAAAAAAAAAAATTTACAGATTTATTGCCTATCTCTAAGCCTCAGGGTCCTTTTCTGCCACATATTCC
b_CM000013.4:4729652    : TGC--------------CTCTCTCTCTCTCTCTCTCTGTGACTATCAT--AAATAAATAAAAA------TTAAAAAAATATATATATATAAATGGAGAAGGGGCAGGCCAAGTACTATGGGAGAAGCATTTCTTCTTCCC
b_CM000019.4:44555175   : TGC------------CTCTCTCTCTCTCTCTCTCTCTGTGACTATCAT--AAATAAATAAAAA--AAAATTAAAAAAAAATATATAATATGTATGTGTTTTATCTTCTATCACTCCACATGTACCTTCTACTCTACTCTA
s_CM021965.1:38018467   : TGC----CTCTCTCTCTCTCTCTCTCTCTCTCTCTCTCTGTGACTATCAT--AAATAAATAAAAA------TTTAAAAAAAATAAATAAATAAAGCACAAGTCCAGCAGTTTCCTGCTGAAGAAACAACTAATGAGCTGCCA
s_CM021989.1:34192529   : TGC--------------CTCTCTCTCTCTCTCTCTCTGTGTGACTATCAT--AAATAAATAAAAA------TTAAAAAAAAAATTTAAACAAAAGGACAAAGTTCAACAAGTGTATGTTGTCTCTGTTCAAGTCCATAATCC
d_CM016578.1:31768031   : TGC--------------CTCTCTCTCTCTCTCTCTCTGTGACTATCAT-AAAATAAATAAAAA------TTAAAAAAAAAATTTAAAATTCTTGGGTCCCAGTCCAGGCCTACAGAATCTGGGACTCTGGGAACGGGGTC
```

Figure S7. Analysis of 3’-terminal regions of Can_b2 sequences of the tribe 2. The upper copy (chr9:16208268-16208630) was used as the search query (marked blue in the sequence above). Sequence names are given in black if present in all three breeds, while those available in one or two genomes are given in blue and violet, respectively. The letter prefix in sequence names indicates the genome where they are present (s, German Shepherd; d, Great Dane; and b, Boxer). Only the 3’-terminal Can and flanking sequences are presented; the missing 5’-terminal sequences are identical in these 265 copies. Rudimentary terminators (TT and TTT) are marked green, while functional ones are marked red ($T_{>3}$) or brown (TCTTT). A-stretches are marked yellow and T residues within TA_{3-5} repeats are marked purple; TSDs are underlined.
