# Supplementary material for: Tail Wags Dog’s SINE: Retropositional Mechanisms of Can SINE Depend on Its A-Tail Structure
Source: Biology (Basel). 2022 Sep 26;11(10):1403. doi: 10.3390/biology11101403 (PMC9599045; doi:10.3390/biology11101403)
Supplement: Supplementary file 1 [file biology-11-01403-s001.zip › Figure S8.pdf]

A

```
FGFR2_Alu_Yb8         : GCAGTCCGACCTGGGCGACAGAGCGAGACTCCGTCTCAAAAAAAAAAAAAAAAAAAAAAAAAAAAAAAAAAAAAAAAAAAAAAAAGAATTACCCGCCAAGCA-----------------------
NC000018.10:14822818  : GCAGTCCGACCTGGGCGACAGAGCGAGACTCCGTCTCAAAAAAAAAAAAAAAAAAAAAAAAAAAAAAAAAAAAAAAAAAAAAAAAAAAAAAAAAAAAAAAAAAAAAAAAAACCAAGCACGTATAGAAATTGC
NC000007.14:73775678  : GCAGTCCGACCTGGGCGACAGAGCGAGACTCCGTCTCAAAAAAAAAAAAAAAAAAAAAAAAAAAAAAAAAAAAAAAAAAAAAAAAAAAAAAAAAAAGAAATTGAAACCATCCTGGCCAACATGGTGA
NC000007.14:27743617  : GCAGTCCGACCTGGGCGACAGAGCGAGACTCCGTCTCAAAAAAAAAAAAAAAAAAAAAAAAAAAAAAAAAAAAAAAAAAAAAAAAAAAAATAAAGATACTAAACTAACTGTAAGTGAAATGTCTTGA
NC000014.9:19713750-  : GCAGTCCGACCTGGGCGACAGAGCGAGACTCCGTCTCAAAAAAAAAAAAAAAAAAAAAAAAAAAAAAAAAAAAAAAAAAAAAAAAAAAATGTGTTCCTTTTTTAAGAATGAAGAGAAGATAATGAAC
NC000005.10:81886516  : GCAGTCCGACCTGGGCGACAGAGCGAGACTCCGTCTCAAAAAAAAAAAAAAAAAAAAAAAAAAAAAAAAAAAAAAAAAAAAAAAAAAGAAAAGCAGTACTAAAGCTACAGATATGCCTTTATTTCCC
NC000004.12:80381224  : GCAGTCCGACCTGGGCGACAGAGCGAGACTCCGTCTCAAAAAAAAAAAAAAAAAAAAAAAAAAAAAAAAAAAAAAAAAAAAAAATGTATATGTTAAGAGTAATTATAAATAAAAGAATCAGCAAGAG
NC000004.12:16618679  : GCAGTCCGACCTGGGCGACAGAGCGAGACTCCGTCTCAAAAAAAAAAAAAAAAAAAAAAAAAAAAAAAAAAAAAAAAGAAAAGGTTGGAAGATAAACATTTGGGGGCAAGAATCTAGAATATGG
NC000006.12:28222267  : GCAGTCCGACCTGGGCGACAGAGCGAGACTCCGTCTCAAAAAAAAAAAAAAAAAAAAAAAAAAAAAAAAAAAAAAAAAGATGATAATAATAGCATAAACAATAGTCACCCAAGTGAGTCAGAGTC
NC000021.9:14884557-  : GCAGTCCGACCTGGGCGACAGAGCGAGACTCCGTCTCAAAAAAAAAAAAAAAAAAAAAAAAAAAAAAAAAAAAAAAAGAAGCCTCAAAGTTGGTATTGGAAAAGATTTCAAAGTTTCTAACACAG
NC000012.12:20709400  : GCAGTCCGACCTGGGCGACAGAGCGAGACTCCGTCTCAAAAAAAAAAAAAAAAAAAAAAAAAAAAAAAAAAAAAAAAGAATTGAAAACAGTTAACCTCATTAAGTGATTCTTAACCTACTATGGT
NC000007.14:38473223  : GCAGTCCGACCTGGGCGACAGAGCGAGACTCCGTCTCAAAAAAAAAAAAAAAAAAAAAAAAAAAAAAAAAAAAAGTATTCCATGAAATGTAAGAGCAAAAAAGTAATATCTGTATTTTTTAAA
NC000007.14:80112605  : GCAGTCCGACCTGGGCGACAGAGCGAGACTCCGTCTCAAAAAAAAAAAAAAAAAAAAAAAAAAAAAAAAAAATTGGGATTGAAAATAAATTATTTAGAAGAACACTCATTTGTTTTAAATGAA
NC000001.11:81221913  : GCAGTCCGACCTGGGCGACAGAGCGAGACTCCGTCTCAAAAAAAAAAAAAAAAAAAAAAAAAAAAAAAAATGTGTTAAAATAACTCATTTAGCACTTTTCTATGTGGCTATATCCTACATTGT
NC000004.12:17723551  : GCAGTCCGACCTGGGCGACAGAGCGAGACTCCGTCTCAAAAAAAAAAAAAAAAAAAAAAAAAAAAAAAAAGAATACTTATGTGGTCTTGTCCTTAAGAGATCTACACCAGAATTAAGATTAGA
NT187614.1:283622-28  : GCAGTCCGACCTGGGCGACAGAGCGAGACTCCGTCTCAAAAAAAAAAAAAAAAAAAAAAAAAAAAAAAGAAAAACGCTTGAAGGTAGCTAGAAAGAAGGATCAAGTCACCTATAAAGGAAGTC
NC000018.10:39481483  : GCAGTCCGACCTGGGCGACAGAGCGAGACTCCGTCTCAAAAAAAAAAAAAAAAAAAAAAAAAAAAAAAGAATCAGATTCATAGACTGTTAGGTTTTTATGCACAACTATTTACATTCAATTAT
NC000003.12:3831334-  : GCAGTCCGACCTGGGCGACAGAGCGAGACTCCGTCTCAAAAAAAAAAAAAAAAAAAAAAAAAAAAAAGACTATGACACAAAGCTGGAGAGTTGATATACCCCTGGGGTAGGACAGTAAAGGTA
NC000002.12:57115434  : GCAGTCCGACCTGGGCGACAGAGCGAGACTCCGTCTCAAAAAAAAAAAAAAAAAAAAAAAAAAAAAAGCTACTAGCCCCGGGAGAGGGGCTGATGTATGCAAAGTGCAGAGAAAGAGAGAAAG
NC000003.12:35010006  : GCAGTCCGACCTGGGCGACAGAGCGAGACTCCGTCTCAAAAAAAAAAAAAAAAAAAAAAAAAAAAAGAATTTAAATATACCTTCTTTAAATACCTCGAAAAACATCTTCCCATAGGCTGAAAA
NC000011.10:59041630  : GCAGTCCGACCTGGGCGACAGAGCGAGACTCCGTCTCAAAAAAAAAAAAAAAAAAAAAAAAAAAAAGAATTCCAAGTGTTATTTCAAAGGAAATTTACAGATATGTTTCCAAATTTTCTATGC
NC000006.12:16963783  : GCAGTCCGACCTGGGCGACAGAGCGAGACTCCGTCTCAAAAAAAAAAAAAAAAAAAAAAAAAAAAAGAAACTAATCAATAACTGTTATTGCATTGAAGCAATAACTTTTAATCAGTAACTTTT
NC000009.12:67538137  : GCAGTCCGACCTGGGCGACAGAGCGAGACTCCGTCTCAAAAAAAAAAAAAAAAAAAAAAAAAAAAAGAATGTGTTGTTTAATTTCCACAAATTTTTGAATTTTCTAGTTTTCCTTCTTTAAAA
NC000003.12:12759219  : GCAGTCCGACCTGGGCGACAGAGCGAGACTCCGTCTCAAAAAAAAAAAAAAAAAAAAAAAAAAAAACAGCATGTTGCTCCACACCGCCTCGTGTTGTGTGTCGGCGCGCTCGCAGGGTTCGAA
NC000002.12:19674973  : GCAGTCCGACCTGGGCGACAGAGCGAGACTCCGTCTCAAAAAAAAAAAAAAAAAAAAAAAAAAAAGAAATTTAATACTTTTATTAGAATGTGTCTCAAAGTTGATCATTCAATTTTTCCCAAG
NC000005.10:88736013  : GCAGTCCGACCTGGGCGACAGAGCGAGACTCCGTCTCAAAAAAAAAAAAAAAAAAAAAAAAAAAATATTTCAGCAACTCTCCATCTGGGCCTCTGGTCTCTACTTGAGGGGTTCTTCACCAGG
NC000020.11:35033664  : GCAGTCCGACCTGGGCGACAGAGCGAGACTCCGTCTCAAAAAAAAAAAAAAAAAAAAAAAAAAAGAGTAAAAAAAAAGAAGAGGTACCTCTTTTTATTTGCTTACCATCAAAATCTCCTCAGG
NC000008.11:13920365  : GCAGTCCGACCTGGGCGACAGAGCGAGACTCCGTCTCAAAAAAAAAAAAAAAAAAAAAAAAAAAGAAATTGGACCATTCCATCTAGATTATCAAATGTATGAGCCTAGAGTTGTTTATAGTAT
NC000009.12:94530464  : GCAGTCCGACCTGGGCGACAGAGCGAGACTCCGTCTCAAAAAAAAAAAAAAAAAAAAAAAAAAACATGGTGTTTGGTTTTCCGTTCCTGCATTAGTTTGTTGAGGATAATGGCTTCCAGCTCC
NC000003.12:21415842  : GCAGTCCGACCTGGGCGACAGAGCGAGACTCCGTCTCAAAAAAAAAAAAAAAAAAAAAAAAAAGAAGTTCATGCAGGTCATGAAGAGTCACAGAGTTTAAAGAATTCTCTGGTTGGGTCATCA
NC000016.10:74567380  : GCAGTCCGACCTGGGCGACAGAGCGAGACTCCGTCTCAAAAAAAAAAAAAAAAAAAAAAAAAGAAAGTGCACCATATATAGTAAGGTTTCAGCACAGGAAGCAGTGATTTTTATCTTTTGGAG
NC000012.12:84090534  : GCAGTCCGACCTGGGCGACAGAGCGAGACTCCGTCTCAAAAAAAAAAAAAAAAAAAAAAAAAGTTACAAGACAAAAAAGACATTATATGTTAATAAAATTTTCAATATAGCAAGAAGATTAAC
NC000010.11:24886782  : GCAGTCCGACCTGGGCGACAGAGCGAGACTCCGTCTCAAAAAAAAAAAAAAAAAAAAAAAAAGAAGGAGTATTAACAAGATACAAAGATGTAAAATACAAAGATGGTAAAATGCAGGATACGC
NC000011.10:11406992  : GCAGTCCGACCTGGGCGACAGAGCGAGACTCCGTCTCAAAAAAAAAAAAAAAAAAAAAAAAAGAAAGGTACTCTGACACCTATCGTGTTTAACAGCCCCAGCAGAGTCTAGGGCAGCACTCTG
NC000001.11:23643144  : GCAGTCCGACCTGGGCGACAGAGCGAGACTCCGTCTCAAAAAAAAAAAAAAAAAAAAAAAAAGACCCCAGGCAGACCTTATAGACTTGTTGGAGAGGCAGTGCACACAGTTTGATTCTCGGAT
NC000003.12:15569298  : GCAGTCCGACCTGGGCGACAGAGCGAGACTCCGTCTCAAAAAAAAAAAAAAAAAAAAAAAAGAAAGCACAGACTTAAAACACATTATTGAGATTATAGACTATTTTTCCCATCTTTACTCTTT
NC000013.11:50977640  : GCAGTCCGACCTGGGCGACAGAGCGAGACTCCGTCTCAAAAAAAAAAAAAAAAAAAAAAAAGAAAAGCATTAACTGGGAAAATAAATGAGCAGCAAAATTATCAGTTTCCATTCAGTGATTAG
NC000002.12:16803911  : GCAGTCCGACCTGGGCGACAGAGCGAGACTCCGTCTCCAAAAAAAAAAAAAAAAAAAAAAAGCAGATCAGTGACTGCCTTTGCCAGAGGTGGAGGGAGGAATGGATTGCAAAAGGGACACAAG
NC000005.10:24452544  : GCAGTCCGACCTGGGCGACAGAGCGAGACTCCGTCTCAAAAAAAAAAAAAAAAAAAAAAAGAAAACAATAGAACTTGGACATTTTAGTGTCTAAGACAACCAAACACTCAAACTTAGAAAAAA
NC000009.12:81863674  : GCAGTCCGACCTGGGCGACAGAGCGAGACTCCGTCTCAAAAAAAAAAAAAAAAAAAAAAAGTAGTACAATTTTAAAAAGAAAAATAAGTAAAAGCTTATTGTAACAGACAGGAGACTCAAGGC
NC000019.10:56600009  : GCAGTCCGACCTGGGCGACAGAGCGAGACTCCGTCTCAAAAAAAAAAAAAAAAAAAAAAATACAGAGGGTTGCAGTGAGCCAAGATTGCACCACTGCACTCCAGCCTGGCAACAGAGC-GAGA
NC000010.11:48708926  : GCAGTCCGACCTGGGCGACAGAGCGAGACTCCGTCTCAAAAAAAAAAAAAAAAAAAAAAAGAGCACACATCTGTCTGTGTGGCTGTAGGAGAGTCTGTCTGAGTCTTGTGTATAAGACCCCAC
NC000001.11:22011194  : GCAGTCCGACCTGGGCGACAGAGCGAGACTCCGTCTCAAAAAAAAAAAAAAAAAAAAAGTAGGTGGTCGGAGGGGTGTAGATAAAAACAAAATTAACCAAAAGTTGATTATTGTTGAAACTGA
NC000003.12:29578613  : GCAGTCCGACCTGGGCGACAGAGCGAGACTCCGTCTCAAAAAAAAAAAAAAAAAAAAAAGAAGCATGTATTACCTGTGATTCTTTGTTTTTGTCATACTGTACATTCTGTATATTCCCTCCGC
NC000020.11:30980029  : GCAGTCCGACCTGGGCGACAGAGCGAGACTCCGTCTCAAAAAAAAAAAAAAAAAAAAAGATGTTTCTGAGAATGCTTCTGTCTGGTTTTTATGTGAAGATATTCCTTTTCCACCATAGGCC
NC000010.11:10747532  : GCAGTCCGACCTGGGCGACAGAGCGAGACTCCGTCTCAAAAAAAAAAAAAAAAAAAAAGAAATTTCACTTTTCTTTTATACAATTAAGAACAATGATTCTGCACCAGATGAGGCAAGACCAAA
NC000007.14:95732177  : GCAGTCCGACCTGGGCGACAGAGCGAGACTCCGTCTCAAAAAAAAAAAAAAAAAAAAAGAATGGGAGTGACAACCTCCACGCTCTTTACTTGGTATAGCTAAACCAGAAGTCTCAAAAAGTTA
NC000002.12:15503449  : GCAGTCCGACCTGGGCGACAGAGCGAGACTCCGTCTCAAAAAAAAAAAAAAAAAAAGTAACAGCAGAACTGAACAAAAGAAATTCTTCACCTAGTAATAGACAGTCTAAACAGATATAATTTA
NC000004.12:18933251  : GCAGTCCGACCTGGGCGACAGAGCGAGACTCCGTCTCAAAAAAAAAAAAAAAAAAAGAGAAGTATCTGTTCATGTCTTCTGCCCATTTTTAATGGGGTGACTTGTTTTTTGCTTGTTTTGTTG
NC000007.14:24545512  : GCAGTCCGACCTGGGCGACAGAGCGAGACTCCGTCTCAAAAAAAAAAAAAAAAAAAGAGCTATTGCAGCAATATGTTCTTTGTAGCCAAAGGGAAATCTTAAAAAAGAGAAAGATAAGCCTTG
NC000003.12:54809130  : GCAGTCCGACCTGGGCGACAGAGCGAGACTCCGTCTCAAAAAAAAAAAAAAAAAAGAAAGAAGGAAGGAAGGAAAGAAAGAGAGAAGAAAGAGATAAAGGAAGGAAGGAAGAAAGGAAGAGAG
NC000001.11:8433372-  : GCAGTCCGACCTGGGCGACAGAGCGAGACTCCGTCTCAAAAAAAAAAAAAAAAAAGAAATGAATGGACTGTTACTCAGAGGGCTTGGATTTGGGATCCTGTTGTAATTACCATAGAATCTTCT
NC000023.11:13386346  : GCAGTCCGACCTGGGCGACAGAGCGAGACTCCGTCTCAAAAAAAAAAAAAAAAAAGGAATATTTTTATGTTAACTAGGGGTGTGTGTGTGTGTGTGTGTTGATGTTGGTGGTGGTTGCACA
NC000022.11:17750891  : GCAGTCCGACCTGGGCGACAGAGCGAGACTCCGTCTCAAAAAAAAAAAAAAAGACTGAACACAAATTTCTACAGAACACTGTGAATAGAATAATCCTGTTTTAAAAAGTATTTGCCTGTGTGT
NC000004.12:16358061  : GCAGTCCGACCTGGGCGACAGAGCGAGACTCCGTCTCAAAAAAAAAAAAAAACTTTAATACTTACTTAAATCATTCCTGATGGAAATCTTTTAAAACAAGTCATTCTAAAACAAAAATTTTTC
NC000012.12:11377700  : GCAGTCCGACCTGGGCGACAGAGCGAGACTCCGTCTCAAAAAAATAAATAAATAAATAAATAAATAAATAAATAAAGTAGTGCCCCAAACTCACCCTGGACTCATTACCCTAGCAATCGCCAG
```

B

```
FBP1_Alu_Ya5           : CTGGGCGACAGAGCGAGACTCCGTCTCAAAAAAAAAAAAAAAAAAAAAAAAAAAAAAAAAAAAAAAAAAAAAAAAAAAAAAAGAAGCTGGACGTCCTCTCCAA--------------------------------
NC000001.11:11491640 : CTGGGCGACAGAGCGAGACTCCGTCTCAAAAAAAAAAAAAAAAAAAAAAAGAAGTTAATAATCGTACTAGCTGCTCTGGTTCAGCAGGGGAGCAGTATCACCATCCCCATTTTACAGATTTCCCTCTTGGT
NC000013.11:35864083 : CTGGGCGACAGAGCGAGACTCCGTCTCAAAAAAAAAAAAAAAAAAAAAAAAAAAGTTGGCTATCTAAATGGAAATAAAAACTGTAAAACAAACAAAAAAACTCCATTTTTCTTCTCTCTTTTTTTTTTTTTTT
NC000016.10:11974966 : CTGGGCGACAGAGCGAGACTCCGTCTCAAAAAAAAAAAAAAAAAAAAAAAAAAGGTGATGGATTGAGTGTGAACACAAGTGTGACACATTCACAACGTTCTTTTGTTTTGTTTTGTTTTTGAGACGGCATCTC
NC000003.12:12577037 : CTGGGCGACAGAGCGAGACTCCGTCTCAAAAAAAAAAAAAAAAAAGACAGTACATGAAAAATGCTGGGCATAGCATTTATTTATTTTTATGTTTTTTAAAGACCTAATCTTACTCTGTTGCCCAGGCTGGG
NC000008.11:51735365 : CTGGGCGACAGAGCGAGACTCCGTCTCAAAAAAAAAAAAAAAAAAAAAAAAAAAAAAAAAAAAAAAACAATTTTTAATTAATTAATTAATTTTATTTATTTATTTTTTTCTGAGACAGTCTTGCTCTCTTGCC
NC000005.10:11804591 : CTGGGCGACAGAGCGAGACTCCGTCTCAAAAAAAAAAAAAAAAAAAAAAAAAAAAAAAAAAAAAATATATATATATATATATATATATATATATATTTATATATAATATAATATATAATATATATAAAATATATATAT
NC000010.11:15428890 : CTGGGCGACAGAGCGAGACTCCGTCTCAAAAAAAAAAAAAAAAAAAAAAAAAAAAAAAAATAACATTTATAGTCCTTGCAGACAGTTTATCATCACGTTGCCTCATTAAATGTAGTTTGCGTTATTTTTATTGTTG
NC000005.10:82037066 : CTGGGCGACAGAGCGAGACTCCGTCTCAAAAAAAAAAAAAAAAAAAAAAAAAAAAAAAAAAAAAAGTAAATGAGATCTTATTACAAAAAGTGATAAAACTGCAGATGATTTATTCACCTAAATAATCTAAGGGACTCA
NC000015.10:29492952 : CTGGGCGACAGAGCGAGACTCCGTCTCAAAAAAAAAAAAAAAAAAAAAAAAAAAAAAAAGGGTTGTCAGGGAAGCATTGGAAATACTATTATTGAATTACTAATAAGTTCACTAATTAGGATTTTAACTGAAGTCAC
NC000004.12:17046363 : CTGGGCGACAGAGCGAGACTCCGTCTCAAAAAAAAAAAAAAAAAAAAAAAAAAAAAAAAAAAAAAAAATATATATATATATATATATATATATATATGAAGACTTGCTGTATGTCCAAGAATGCAGTTGATCAGTTT
NC000015.10:37569911 : CTGGGCGACAGAGCGAGACTCCGTCTCAAAAAAAAAAAAAAAAAAAAAAAAAAAAAAAAAATCTTTAAAAATGTTTAATAAGAATTGCATTTAACCTGTATATTAATTTGGGAGAATTGACTTTTTTCTATGTTG
NC000024.10:14489239 : CTGGGCGACAGAGCGAGACTCCGTCTCAAAAAAAAAAAAAAAAAAAAAAAAAAAAAAAAAAAATATATATATATATATATATATATATATATATATAACACAATTTTCATAAATGTTTTTAAACATCAAACTTGA-
NC000002.12:13748758 : CTGGGCGACAGAGCGAGACTCCGTCTCAAAAAAAAAAAAAAAAAAAAAAAAAAAAAAAAAAAAAAAAAAAAAAAAAAAAAAGAAAGAAACCTCAAAATATTTCTTCTAGATTTTCTATTTTATTTGCGTAGAGGTGTTTG
NC000011.10:10395468 : CTGGGCGACAGAGCGAGACTCCGTCTCAAAAAAAAAAAAAAAAAAAAAAAAAAAAAAAAAAAAAAAAAAAAAAAAAAAAAACAGTAATGAACAAACACATGATCAACATCCATTCTTTCAACAAATTCTAAGTTTGTTC
NC000005.10:92184770 : CTGGGCGACAGAGCGAGACTCCGTCTCAAAAAAAAAAAAAAAAAAAAAAAAAAAAAAAAAAAAAAAAAAAAAAAAAAAAAAAAAAAAAAAATAATAATAATAAAGTCTATTATCACAAAAAATTCAATAAGCACTGACAGAAT
NC000013.11:88350866 : CTGGGCGACAGAGCGAGACTCCGTCTCAAAAAAAAAAAAAAAAAAAAAAAAAAAAAAAAAAAAAAAAAAAAAGAAAAATACTAGATTTAGATATCAGACATTAGAATTAGGGAAAGGACTGTTACAGTACTATGGTGA
NC000014.9:106332409 : CTGGGCGACAGAGCGAGACTCCGTCTCAAAAAAAAAAAAAAAAAAAAAAAAAAAAAAAAAAAAAAAAAAAAAAAGAATGGAAGAAAGGAATAGAATGAAAGCTCATAAACCAGGTTAAGTCATTCTGAATATCTTTTA
NC000006.12:51652397 : CTGGGCGACAGAGCGAGACTCCGTCTCAAAAAAAAAAAAAAAAAAAAAAAAAAAAAAAAAAAAAAAACAAGAGAGATAAAAAATGCTCAGGACATCATGAAGCATTCAATCAAAACATGAGGCTTTTATAGGAACT
NC000005.10:16710540 : CTGGGCGACAGAGCGAGACTCCGTCTCAAAAAAAAAAAAAAAAAAAAAAAAAAAAAAAAAAAAAAAAAAAAAAAGAAAGTGATCAAAGGAGGAATGTGTGTGCAAGCAGCTTTGAGAAATGTGAAGTATTATAATATG
NC000003.12:90303597 : CTGGGCGACAGAGCGAGACTCCGTCTCAAAAAAAAAAAAAAAAAAAAAAAAAAAAAAAAAAAAAAAAAAAAAAAAAAAAAAAAAAAGAAAGAGTGTATTAAAACTGCTCAGTCAAAACAGAGGTTCAACTCTGTGAGTTGAA
NC000006.12:8022403- : CTGGGCGACAGAGCGAGACTCCGTCTCAAAAAAAAAAAGAAAAAAAAAAAAAGAAAAAAAAAATAGAGTTTGAAAATAGAGTAACATATAGGTACATATTGTTCTATATAAAATCATTTTTGGCAGTTGTTAT
NC000004.12:12237780 : CTGGGCGACAGAGCGAGACTCCGTCTCAAAAAAAAAAAAAAAAAAAAAAAAAAAAAAAAAAAAAAAAAAAAAAAATTCTAGATTATCATTGAGATCATTAATGGGCACTTGATCGAAGCAACATAATGAACACACATTT
NC000004.12:94577649 : CTGGGCGACAGAGCGAGACTCCGTCTCAAAAAAAAAAAAAAAAAAAAAAAAAAAAAAAAAAGAAGAGTTTTTTATGAGATTTCAAGAAGCACTAGTAGAAAAGATTTTAGTTAAGACACTTCCTTCAATGGATGTTTT
NC000011.10:16201452 : CTGGGCGACAGAGCGAGACTCCGTCTCAAAAAAAAAAAAAAAAGAAAAAAAAAAAAAATACTTTGCAAATAAGGAGCATTTGAACAAACTTTATGAAACCAAGATTATTAGAACCTATACATTGTTAGGTTTA
NC000023.11:11363354 : CTGGGCGACAGAGCGAGACTCCGTCTCAAAAAAAAAAAAAAAAGAAAAAAAAAAAAAAAAAAAAAAAAAAAAAGTAAACTTCATTTTGACATATTTGTAGTTCCACCCGCAATTGAACTATTTGGATATTGCTATAT
NC000002.12:22593974 : CTGGGCGACAGAGCGAGACTCCGTCTCAAAAAAAAAAAAAAAAAAAAAAAAAAAAAAAAAAAAAAAAAAAAAAAGACTGCTTAGTCAATCTGTAATAATTCTGGGGAGTTCAGTGAATAAGTAACCTTGAGCAATTTT
NC000011.10:95957216 : CTGGGCGACAGAGCGAGACTCCGTCTCAAAAAAAAAAAAAAAAAAAAAAAAAAAAAAAAAAAAAAAAAGAATGGCAGGGAAGGGCATTGCAAGCACAGTAAACAGTACACCAATTCTTTGCTCTTCTAGTACTCTGAT
NC000018.10:71179304 : CTGGGCGACAGAGCGAGACTCCGTCTCAAAAAAAAAAAAAAAAAAAAAAAAAAAAAAAAAAAAAAAAAAAAAGAAAAATAGCAAGGTGTTGTAGTGTGTTAACTAAATCCTGAGTCATTCCTTACACTGCAAGGAAGC
NC000005.10:12262762 : CTGGGCGACAGAGCGAGACTCCGTCTCAAAAAAAAAAAAAAAAAAAAAAAAAAAAAAAAAAAAAAAAAAAAAAAAAGATTCTAGAGCTAAGCTTCATTAGCTTCGTGGTGAGTCCACCTCAGGGTCTTATACATCCGT
NC000006.12:29364317 : CTGGGCGACAGAGCGAGACTCCGTCTCAAAAAAAAAAAAAAAAAAAAAAAAAAAAAAAGAACAAAGCTGGAGGCATCATGTTACCTGACTTCAAACTAGACTACAAGGCTACAGTAACCAAAACAGCATGATACTG
NT113891.3:850904-85 : CTGGGCGACAGAGCGAGACTCCGTCTCAAAAAAAAAAAAAAAAAAAAAAAAAAAAAAAAAAAAAAAAAAAAGAACAAAGCTGGAGGCATCATGTTACCTGACTTCAAACTAGACTACAAGGCTACAGTAACCAAAA
NC000006.12:10373512 : CTGGGCGACAGAGCGAGACTCCGTCTCAAAAAAAAAAAAAAAAAAAAAAAAAAAAAAAAAAAAAAAAAAGAAATTCAGAGCTCAAAGACAAAGTCTTGGAAATAACCGAATCAAATGAAGACAAAGAAAAAAGAATA
NC000012.12:99998684 : CTGGGCGACAGAGCGAGACTCCGTCTCAAAAAAAAAAAAAAAAAAAAAAAAAAAAAAAAAAAAAAAAAAAAAGAGGAAATTAAGACAAAGACACTCATAGAGGGACAATAATCTCTAAGCCAAGGAGAGAGGCCTCA
NC000006.12:68629374 : CTGGGCGACAGAGCGAGACTCCGTCTCAAAAAAAAAAAAAAAAAAAAAAAAAAAAAAAAAAAAAAAAAAAAATACATAAATAAATAAATAAATAAATAAATAAAAGGAAATACACATATGCAAAAATAAAACATAAAA
NC000023.11:12119519 : CTGGGCGACAGAGCGAGACTCCGTCTCAAAAAAAAAAAAAAAAAAAAAAAAAAAAAAAAAAAAAAAAAAAAAAAAAATAGAAGAAATCCTGGAGCTAACAGACTCTTCCTAACTTGGAAGAGCCAGGGCTAGAAAGGAA
NC000007.14:49362272 : CTGGGCGACAGAGCGAGACTCCGTCTCAAAAAAAAAAAAAAAAAAAAAAAAAAAAAAAGAAAAACAAAATTATAACAATTAAATAGGACATGAGAAATTAGAAGTGTTCTATATCATTGGTGGGCATATAAAAAGGTA
NC000018.10:30868630 : CTGGGCGACAGAGCGAGACTCCGTCTCAAAAAAAAAAAAAAAAAAAAAAAAAAAAAAAAACAAAGAGGAGAAAAGATGCAAATGGTACTGGAAATATGAAATAGTGTCTTCATTATGTAAAATGGTTGTAACTGT
NC000002.12:35865257 : CTGGGCGACAGAGCGAGACTCCGTCTCAAAAAAAAAAAAAAAAAAAAAAAAAAAAAAAGAAATAAAGAGAAAAATCTTAAAGGCATATGGAGAAACAAAGACGCATTTTCTACAGAGCGGAAAAAAATGACCACAGA
NC000007.14:11755633 : CTGGGCGACAGAGCGAGACTCCGTCTCAAAAAAAAAAAAAAAAAAAAAAAAAAAAAAAGAAATGAAACAAATAAAACATAGAGAAAACTCATAAGGGACCGCTTGGAAAAAAAATTCAAAACAGGTAACTCCAGTGC
NC000017.11:2423932- : CTGGGCGACAGAGCGAGACTCCGTCTCAAAAAAAAAAAATAAAATAATTAAAATAAAATAAAATAAAATAAAGGAAAATTTTTTAAAAAAATCTTCAGGAATATCAAGTTTGTTGACTGACCATTGTATATGG
NC000011.10:41123091 : CTGGGCGACAGAGCGAGACTCCGTCTCAAAAAAAAAAAAAAAAAAAAAAAAAAAAAAAAAACAAAACATAGCTCAGGATAGAGAAAGCATTTATAAAATCCAGAGTTGCTTGAAATCAAGATATGATGACTCACTG
NC000002.12:10135855 : CTGGGCGACAGAGCGAGACTCCGTCTCAAAAAAAAAAAAAAAAAAAAAAAAAAAAAAAAAAAAAAAAAAAAAGAGAGAACTGAGGCAAGTTTAGAGCAGGAGTGAGAGTTTATTAAAAAGTTTTAGGGCAGGAATGA
NC000011.10:23966877 : CTGGGCGACAGAGCGAGACTCCGTCTCAAAAAAAAAAAAAACAAAAAAAACAAAAAAAACAAAAAAACAAAAAACAAAAAACAGATCAGCAGCTTCTAGGAGTACAGGGGAAAGGAGTAGGTTCATCAGGA
NC000001.11:56421867 : CTGGGCGACAGAGCGAGACTCCGTCTCAAAAAAAAAAAAAAAAAAAAAAAAAAAAAAAAAAAAAAGATAAGCAGTTGTCAGGGATTCGGGGGAGTACTGAACAGATGAGGTACAGGGAATCTTTTAGAGTGGTGAAA
NC000010.11:63556454 : CTGGGCGACAGAGCGAGACTCCGTCTCAAAAAAAAAAAAAAAAAAACAAAACAACAAAAAAAAAAAAACAAGCAAACAGAAAACAAAACAAAACAAAACAAAACAAAACAAAAACCCCACCAATTTTAAAAAT
NC000020.11:38955302 : CTGGGCGACAGAGCGAGACTCCGTCTCAAAAAAAAAAAAAAAAGACAAAAAAAAAAGAAATAAGAGCAAGAGATACATGGAGTCCTAAGAAAATGACCCAGTCTCTCAAAGTAAGCAGTCTTTCTGGAGTT
NC000001.11:61567453 : CTGGGCGACAGAGCGAGACTCCGTCTCAAAAAAAAAAAAAAAAAAAAAAAGATTGCTAAAATTTGCTAGACTTATAAATGGATCAATAAGATTTAAGCTACATTTAAAAGCTTAAGGACACACAGCCAGGC
NC000013.11:10706224 : CTGGGCGACAGAGCGAGACTCCGTCTCAAAAAATAAATAAATAAATAAATAAAATAAAATAAAGGTGAGTAATCTTGAATTACATACTCTAGTAATATTTATTTAATTTACTCAGAAGCAGTGAAATTCTACGTG
```

C

```
PKLR_Alu_Yb9         : GGGCGACAGAGCGAGACTCCGTCTCAAAAAAAAAAAAAAAAAAAAAAAAAAAAAAAAAAAAAAAAAAAAAAAAAAAAAAAAAAAAAAAAAAAAAAAAAAAGATCATCAGCAAAATTGAGAACC-------
NC000001.11:388703   : GGGCGACAGAGCGAGACTCCGTCTCAAAAAAAAAAAAAAAAAAAAAAAAAAAAACAAATAGAATTGTGTAAAGCACCTATGTTTGGAGCTGAAAATCAAATTTTTAATAAATCACATCAATACTGTG
NC000016.10:810856   : GGGCGACAGAGCGAGACTCCGTCTCAAAAAAAAAAAAAAAAAAAAAAAAAAAAAAAAAAAAAAAAAAAAAAAAAAAAAAAAACAAAAAAAACACAATTATGAACTCTGAACTACTGGCCAGGAACGGCG
NC000013.11:110587   : GGGCGACAGAGCGAGACTCCGTCTCAAAAAAAAAAAAAAAAAAAAAAAAAAAACTACAAGCCAATGTGCTAATGAATATAGATGCAAAGTCATTAACAAAATATTAGCAAATAGAATTCAGTACTAT
NC000001.11:673620   : GGGCGACAGAGCGAGACTCCGTCTCAAAAAAAAAAAAAAAAAAAAGAAAAAGAAAAAAAAAGAAACCATGCAAGCAAGGAAAGAGTAGAGTAAAATATTTAAAGTATTGAGAGAAAAAAAGCCCATC
NC000020.11:501560   : GGGCGACAGAGCGAGACTCCGTCTCAAAAAAAAAAAAAAAAAAAAAAAAAAGTCAGATCATGTCATTCCTCTACTCAAAACCCCACAGGGAAGGGACTTCCCAGCTCATTTTAAGTAACTGTCTCCC
NC000005.10:555593   : GGGCGACAGAGCGAGACTCCGTCTCAAAAAAAAAAAAAAAAAAAAAAAAAAAGATCCTGTCTCAAAAAAAAAAATAGATAAATAAATAAAATTAAAACAAAATTAACTTGTTAATATCAATGCATTTT
NC000009.12:495660   : GGGCGACAGAGCGAGACTCCGTCTCAAAAAAAAAAAAAAAAAAAAAAAGAGAATTTGCATAATCCCATTTTTTTGTATGCAGTCCTTTAATGAATTTAACAATGCCATACTTATTGCATGCATACAC
NC000011.10:192607   : GGGCGACAGAGCGAGACTCCGTCTCAAAAAAAAAAAAAATAAATAAAATAAAATAAATAAAATTAAATAATAAATAAATAAATACTTGTCTACTTTAGGCATTTTTTTACAGTCAGCATGTATTGTT
NC000004.12:170357   : GGGCGACAGAGCGAGACTCCGTCTCAAAAAAGAAAAAATAAAATAAAAGATAAAATAAAATAAAATAAAAATAAAAAATAAAAAAATAAAAAAATTTTAAAAAAAGTACTGGTGAAGATGTGAAGCA
NC000016.10:307990   : GGGCGACAGAGCGAGACTCCGTCTCAAAAAAAAAAAAAAAAAAAAGTGAGTAGTGAGTCTGTGAGTGCAAAAATTAGCCGGTTGTGGTGGTGTGCGCCTGTAGTCCCAGCTACTCGGGAGGATGAAG
NC000003.12:479299   : GGGCGACAGAGCGAGACTCCGTCTCAAAAAAAAAAACAAACAAACAAACAAAAAAAACAAAAGACAACAACAAAGGTACTATTAAAGAAAATGAAAAGAGAAGTCATAGACTTCGAGAAATATCTGC
NC000009.12:153946   : GGGCGACAGAGCGAGACTCCGTCTCAAAAAAAAAAAAAAAAAAATACATATATACACACATATGTATGTATATATGTATATATGTGTGTGTGTATATATATGTATGTGTGTGTGTGTGTATATAT
NC000006.12:419183   : GGGCGACAGAGCGAGACTCCGTCTCAAAAAAAAAAAAAAAATACAAAAAAATTAGCCAGGCATGGTGGTGGGCGCCTGTAGTCCCAGCTACTCGGGAGGCTGAGGCAGGAGAATGGCATGAACCCGG
NC000006.12:113743   : GGGCGACAGAGCGAGACTCCGTCTCAAAAAAAAAAAAAAAAAAAAAAAAAAAAATACAAAATTAGCCGGATGTGGTGGCACACACCTGTAGTCCCGGCTACTTGGGAGGCTGAGACAGGAGAATTGC
NC000005.10:777523   : GGGCGACAGAGCGAGACTCCGTCTCAAAAAAAGAAAAACAAAACAAAACAAAACAAAACAAAAATAAGCCGGACATGGTGGCATGTGCCTGTAATCCCAGCTACTTTGGAGGCTGAGGCAGTAGGAT
NC000014.9:5656431   : GGGCGACAGAGCGAGACTCCGTCTCAAAAAAAAAAAAAAAAAAAAAAAAAAAAAAAAAAAAAAAAAAAAAGAAAAGAGAAAAGCACCGAGGAGGGAAAATAATGAATCTAAAATTCTATACCCAGCT
NC000009.12:363091   : GGGCGACAGAGCGAGACTCCGTCTCAAAAAAAAAAAAAAAAAAAAAAAGATGCTTGACATCATTAACCATCAGGAAAATGCAAATCAAAACCTCAAAGAGATATCACTTCACACCCACTAGGATAAT
NC000011.10:626537   : GGGCGACAGAGCGAGACTCCGTCTCAAAAAAAAAAAAAAAAAAAAAAAGTCCTGAGTTGAACATTTTGTATTTTTGGCGTTAGGAAAAATGATTAAAATACCCCTGTGAGTGTTTTAGTTTCTCTCT
NC000015.10:903588   : GGGCGACAGAGCGAGACTCCGTCTCAAAAAAAAAAAAAAAAAAAAAATGTCTGCTAGTAAGAACAAAATTCTAAGGCAGCATTCCAAATTCTTCATCTAAACAAGGCTGAAATGCTAGATTTCCTTC
NC000011.10:613401   : GGGCGACAGAGCGAGACTCCGTCTCAAAAAAAAAAAAAAAAAAAAAAAAAAAAAAAAAAAAAAAGAGGAAGGCAGGGATGGCTGGGCGCGGTGGCTCACGCTCGTCATCCCAGCACTTTGGGAGGCC
NC000012.12:122973   : GGGCGACAGAGCGAGACTCCGTCTCAAAAAAAGAAAAAAAAAAAAAAAAAAAAAAAAAAAAAAACAAAAACTTTGGGAGGCCGAGGCAGGGGGACCACGAGGTCAGGAGATCAAGACCATCCTGGCT
NC000006.12:104463   : GGGCGACAGAGCGAGACTCCGTCTCAAAAAAAAAAAAAAAAAAAAAAAGAAAACAAAGCAGGCCAGGTACGGTGGCTCACGCCTGTATCCCAGCACTTTGAGAGGCTGAGGCAGGAGGACTGCTTGA
NC000014.9:5810233   : GGGCGACAGAGCGAGACTCCGTCTCAAAAAAAAAAAAAAAAAAAAAAGAGTAAGGCAGAGGTCAGGCACGGTGGCTCACACCTGTAATCCCAGCACTTTGGGAGGCCAAGGCAGGCAGATCACCTGA
NC000017.11:364221   : GGGCGACAGAGCGAGACTCCGTCTCAAAAAAAAAAAAAAAAAAAAAAAAAAGGTCTTAATTTTAAAAAATAGGCCAGGCGTGATGGCTCATGCCTGTAATTCCAGCACTCTGGGAGGCCAAGTCAGG
NC000003.12:577615   : GGGCGACAGAGCGAGACTCCGTCTCAAAAAAAAAAAAAAAAAAAAAGTTGGAAGAAATAGGCCAGGCTTGGTGGCTCATGCCTGTAATCCAGCACTTGGGGAGGCTGAGGCAGACGGATCACGAGGT
NC000017.11:816104   : GGGCGACAGAGCGAGACTCCGTCTCAAAAAAAAAAAAAAAAAAAAAAATGACACAAACCAAAGATAAAAGTGAATGAACTCAAAGTCAATGTAAGTAGTTTATTGGCCAAAGGCAGTGGCTCACACC
NC000008.11:106057   : GGGCGACAGAGCGAGACTCCGTCTCAAAAAAAAAAAAAAAAAAAAAAAAAAAAAAAAAAAAAAAAAAGAAGTTAAAGACCAGTCTGGCCAACATGGTGAAACCCCATCTCTACTAAAAATACAAAAAT
NC000023.11:852540   : GGGCGACAGAGCGAGACTCCGTCTCAAAAAAAAAAAAAAAAAAAAAAAAAAAAAAAAAAAAAAATTAGCCGGGCGCAGTGGCGGGCGCCTGTAGTCCCAGCTACTCAGGAGGCTGAGGCAGAAGAAT
NC000001.11:425423   : GGGCGACAGAGCGAGACTCCGTCTCAAAAAAAAAAAAAAAAAAAAAAGAAAAATTTAAAATTAGCCAGGTGTGGTGCCATGTGCCTGTGGTCTCAGCTCCTTGGGAGGCTGAGGCAGGACAATCACC
NC000007.14:139424   : GGGCGACAGAGCGAGACTCCGTCTCAAAAAAAAAAAAAACAAAAAAAAAAACAAAAAAAAAACAAAAAACAAAAAAAAAAAAAAAAAAAACAAGCCTGGCAGGGGTAGGGCAGGGAGGGGTTGCTCA
NC000022.11:228281   : GGGCGACAGAGCGAGACTCCGTCTCAAAAAAAAAAAAAAAAAAAAAAAAAAAAAAAAAAAAGAAGTAGAGGAATGATTGAATATATTAAGTGAAAATAAGGAATACCAACACAAAGAAAAATTTAAC
NC000016.10:667656   : GGGCGACAGAGCGAGACTCCGTCTCAAAAAAAAAAAAAAAAAAAAAAAAAAAAAAAAATACCCAATAGTTTGTTTGATTTGCTATCCTTTCAATTATTCATCCACTCCTCCCAGCAGATGCACATAT
NC000007.14:565259   : GGGCGACAGAGCGAGACTCCGTCTCAAAAAAAAAAAAAAAAAAAGATTTTCCAAGTTTCATTACAATTGTTATGTTTCCAAAATTGATGTTAATATTTACTTTAAAAAATATGGTTCTGGTCGGGTG
NC000003.12:858970   : GGGCGACAGAGCGAGACTCCGTCTCAAAAAAAAAAAAAAAAAAAAAAAAAAAAAAAAAAAAAAAGATGTAGGTTAAAGCAATTATTGTTGTAATTCTCAGAAACATTAATAATGCTAACATACATCC
NC000011.10:494033   : GGGCGACAGAGCGAGACTCCGTCTCAAAAAAAAAAAAAAAAAAAAAAAAAAAAAAAAAGAAATAATGGTGAAAACCCCTGAAATAATGTGGAAGAGTAATAGATACTTTTAATAGAGCATGGTTAGC
NC000010.11:928232   : GGGCGACAGAGCGAGACTCCGTCTCAAAAAAAAAAAAAAAAAAAAAAAAAGAAAAAGAAAAAGAATAATTATAGTTAATTAAAACAATTAGTCTAGAATTTCCTTGTCAAATTAGAAAAACACCAGT
NC000015.10:392277   : GGGCGACAGAGCGAGACTCCGTCTCAAAAAAAAAAAAAAAAAAAAAAAAAAAAAAAAAAAA_ TTTTAATTTATCAAGAAAGACTATAGAAGATGTAGAAAACCTGAATATACCACAATTCTCCG
NC000018.10:572362   : GGGCGACAGAGCGAGACTCCGTCTCAAAAAAAAAAAAAAAAAAAAAAAAAAAAAACAACAACAACAAACGGGGGAAACCATTTGAAACATACATAGAAGATAGATGTTAGTATTCTTAATATATAGA
NC000007.14:119981   : GGGCGACAGAGCGAGACTCCGTCTCAAAAAAAAAAAAAAAAAAAAAAAAAAAAGAAATGGAAAGCAAGAAGGTATGGTTCCCCAGAAAAATCACTGGAACATACCAATAAATTACAATAAGTAGTTC
NC000008.11:109497   : GGGCGACAGAGCGAGACTCCGTCTCAAAAAAAAAAAAAAAAAAAAAAAAAGAAAACATGATAATGAATAGTACTAAATTCTACTGTAGTAAATAAAATGCAAACTATAAATTCAAAAGTGGATCAT
NC000011.10:437571   : GGGCGACAGAGCGAGACTCCGTCTCAAAAAAAAAAAAAAAAAAAAAAAAAAAACAAATAGGTAAAAAACAAACAAATAAACCAAACCAAAAAAAGCACACACAAAAAAAACAGACAAATAAATAATT
NC000003.12:186311   : GGGCGACAGAGCGAGACTCCGTCTCAAAAAAAAAAAAAAAAAAAAAAAAAAAAAAAAAAAAGAACACAGAGTATTTGAGCATTAGACCATTAAACTGTTAAACTCCGAAAAAACTATAAATCAACAT
NC000005.10:964524   : GGGCGACAGAGCGAGACTCCGTCTCAAAAAAAAAAAAAAAAAAAACAGAAGAAAGAAAAGAAAAAATCCTGTCACAGAAATTCCGAGTAACCAAGGAATGACTTCAGCTAATGAACAAATATGATCC
NC000001.11:180323   : GGGCGACAGAGCGAGACTCCGTCTCAAAAAAAAAAAAAAAAAAAAAAAAAGACATAGAGTAACTGAGTGGATTAAAAAATCAAGACCCAATGATCTGTTGCCTACAAGAGACACACTTCACCTATAA
NC000016.10:801642   : GGGCGACAGAGCGAGACTCCGTCTCAAAAAAAAAAAAAAAAAAAAAAAAGAACAAATAGAGAAAAAAAGTACATATGTAAAACACTACCACTCAACTTGACCTAAGCTACAATTATAGAACATTTTA
NC000012.12:292955   : GGGCGACAGAGCGAGACTCCGTCTCAAAAAAAAAAAAAAAAAAAAAAATTACGTAAAAAATCACTAGCTGCTTGTATATAAACAAAAATTAGAGAAGAAAAGGGAAGAAATGTCCAAAGTTGAACGT
NC000006.12:949489   : GGGCGACAGAGCGAGACTCCGTCTCAAAAAAAAAAAAAAAAAAAAAAAAAAAAAAAAACAACAACAATGTATTGTATACTAAAAATTGGTAAGAGAGTGTATGTTAACTATTCTCACCACAAAAAAG
NC000001.11:878704   : GGGCGACAGAGCGAGACTCCGTCTCAAAAAAAATAAATAAATAAATAAAAAAATAAATAAATAAATAAATAAATAAATAATTGTCTATTTTAATACTAAAAAGGAAAGAAAGAAGGAAGGAAGAAAA
NC000015.10:853990   : GGGCGACAGAGCGAGACTCCGTCTCAAAAAAAAAAAAAAAAAAAATAAAAAAATAAAAAAATAAATAAATAAATAAATAAAGTTTTAGATGCCATAAGATAATTACTCATAGAATTACTGGATTACT
NC000003.12:181491   : GGGCGACAGAGCGAGACTCCGTCTCAAAAAATAAAAAAAATAAAAAATAAATAAATAAAAATAAATAAAAAAAGAGTCTGGTATCAATGTCGGGGGCAGACAAATAGACACATAAATAAATGAAATA
NC000014.9:8744794   : GGGCGACAGAGCGAGACTCCGTCTCAAAAAAAAAAAAAAAAAAAAAGAAGGCGGATGACAAAGATGAATATATTCTACTGCCAAATGAAGTAAATTAATCATACCCCTCCTTTTTTGTATACCATAT
NC000005.10:437082   : GGGCGACAGAGCGAGACTCCGTCTCAAAAAAAAAAAAAAAAAAAAAAAAAATAGTCCATGGAAGATAATTTATTTGGGGAATAGTAGGGAATCCTAACAATGGTAGAGAAAGCTAAGAACTTTAAA
NC000002.12:137238   : GGGCGACAGAGCGAGACTCCGTCTCAAAAAAAAAAAAAAAAAAAAAAAAAAAAAAGAAAGATGAGTGTCATTATCAGGATATAGTGTTATTTAAACAAGCTACTCTGGTAAGGAAAATTTATATCAT
NC000004.12:368409   : GGGCGACAGAGCGAGACTCCGTCTCAAAAAAAAAAAAAAAAAAAAAAAAAAAAAAAAAAAAAAAAAAAGAAGATTCATCCAAAAGTGAAGTAAAAGGGAAAGGCAACATCTTTTTTTTTTTTATTATA
NC000006.12:119135   : GGGCGACAGAGCGAGACTCCGTCTCAAAAAAAAAAAAAAAAAAAAAAAGAATATTTCCAGAAGTATACCACATTTTTTTCTAATGTGTATACTAATCACTAAAATTGTAAAAGTAAAATTCTTGCCC
```

```
NC000009.12:965286 : GGGCGACAGAGCGAGACTCCGTCTCAAAAAAAAAAAAAAAAAAAAAAAAAAAAAAAAAAAAAAGTGCCTCATTCTCATTTTACGCCTAAAGAAAATATAATGGTTAGCAAATATAGGTCCTTTGGTA
NC000013.11:870234 : GGGCGACAGAGCGAGACTCCGTCTCAAAAAAAAAAAAAAAAAAAAAAAAAAATTACATGTGACAAGACATAGGATAATAATACTGGTAATGAATAGAAATTAATAAACATTGGAATCAAACCTACAG
NC000005.10:343271 : GGGCGACAGAGCGAGACTCCGTCTCAAAAAAAAAAAAAAAAAAAAAAAAAAAAAAAAAAAAAAAGAATTGTTTTTTCCTTTCCAACATTTATTATAGAATCGAGGTACATGGCAGCCTTGAAACATG
```

Figure S8. A-tails of Alu copies closely related to those that induced mutations in the *FGFR2*, *FBP1,* and *PKLR* genes (alignments A-C, respectively). Only the 3'-terminal sequences are given since the upstream ones are identical. The upper sequence corresponds to the Alu copy that caused gene mutations. All copies of the *FGFR2* tribe are given, while randomly selected 49 out of 419 and 59 out of 232 copies are given for the *FBP1* and *PKLR* tribes, respectively. All identified TSDs are underlined. Within A-rich tails, A residues are highlighted yellow; Ts are highlighted purple; while Cs and Gs are highlighted green.
