# Supplementary material for: Tail Wags Dog’s SINE: Retropositional Mechanisms of Can SINE Depend on Its A-Tail Structure
Source: Biology (Basel). 2022 Sep 26;11(10):1403. doi: 10.3390/biology11101403 (PMC9599045; doi:10.3390/biology11101403)
Supplement: Supplementary file 1 [file biology-11-01403-s001.zip › Figures S1 and S2.pdf]

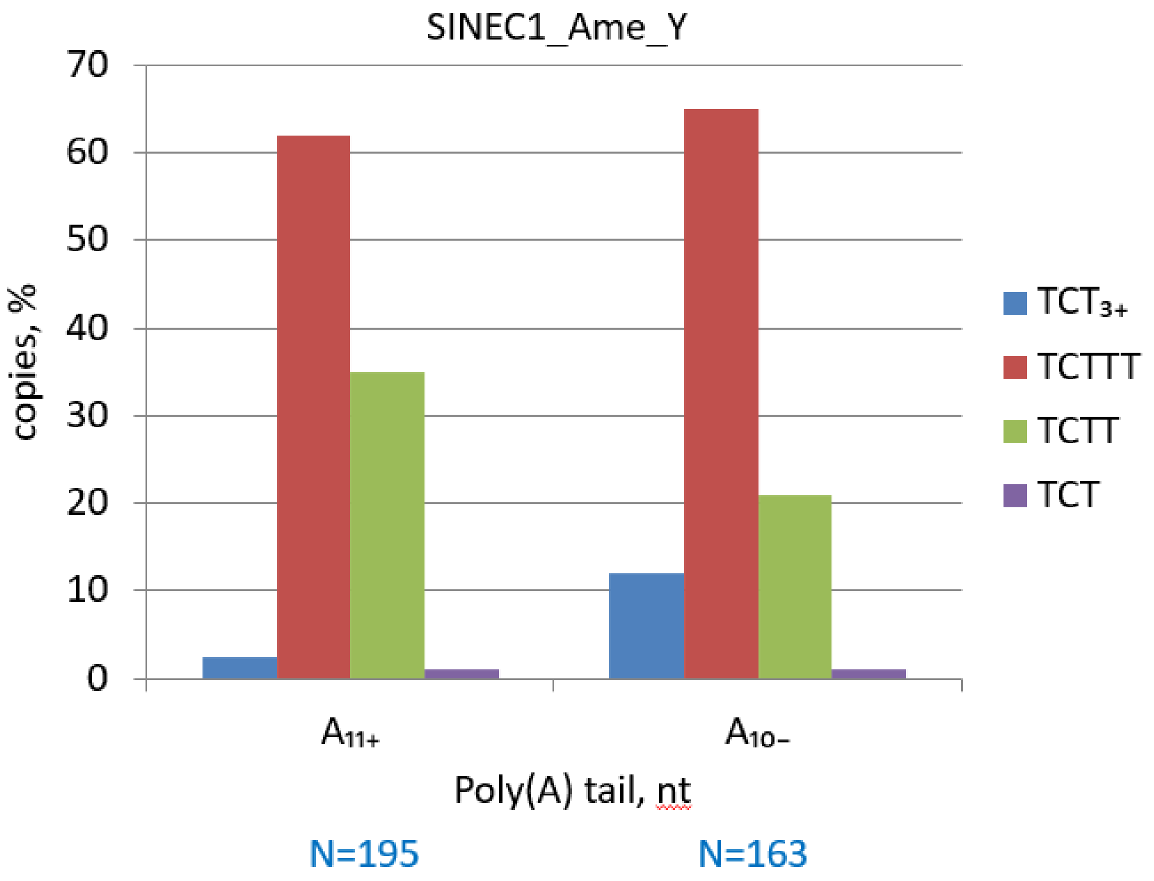


**Figure S1.** Distribution of pol III terminators or their rudiments among relatively young Can copies (SINEC1_Ame_Y) present in the giant panda (*Ailuropoda melanoleuca*) but missing in orthologous loci of the polar bear (*Ursus maritimus*). Notice that the shortening of poly(A) tails ($A_{\leq 10}$) is accompanied by an increased proportion of copies with strong terminators ($TCT_{>3}$).

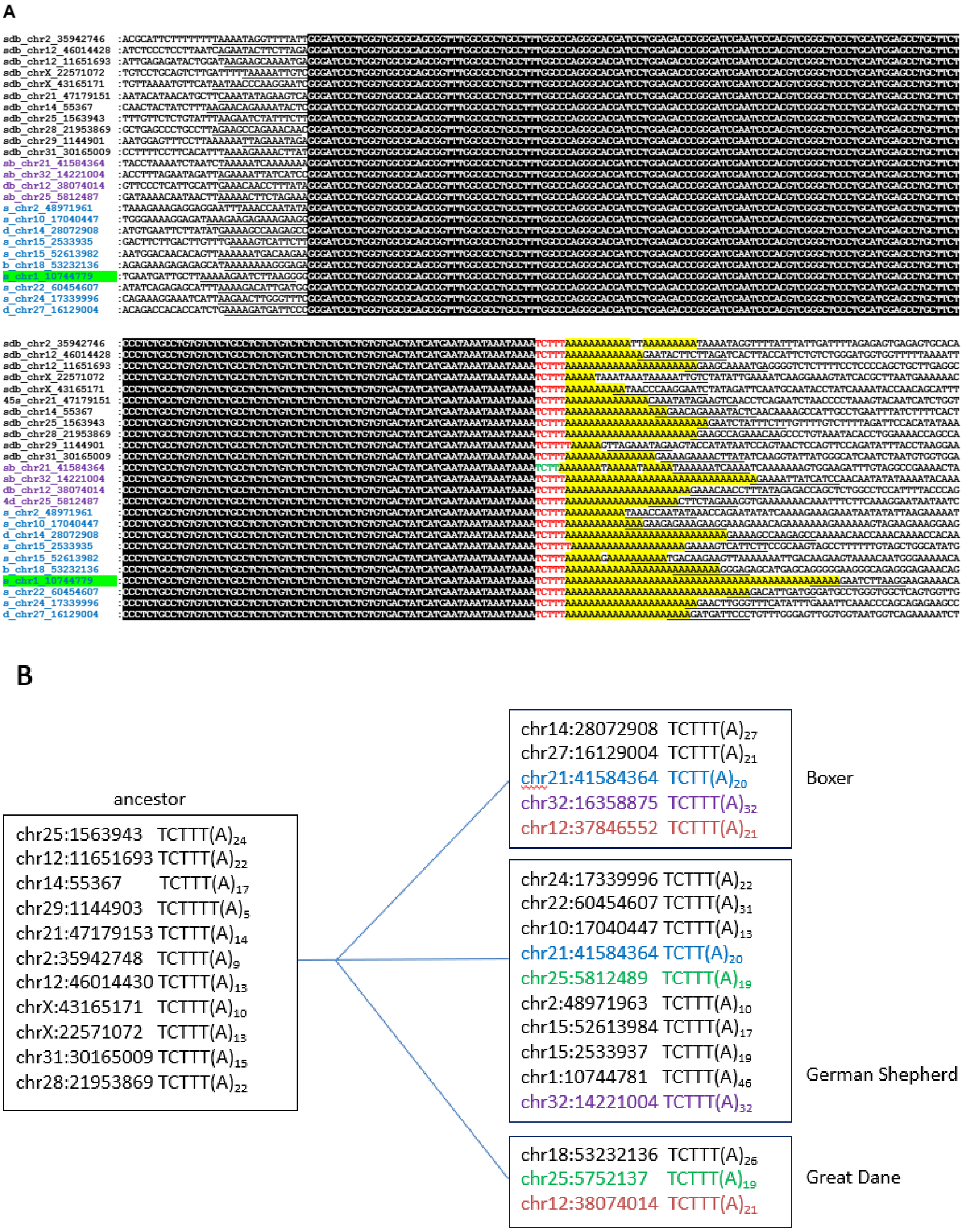


**Figure S2. A.** Multiple alignment of sequences from one of Can_b1 tribes in the three dog breeds. The copy marked green (chr1_10744779) was used as the search query. The names of copies found in all genomes are given in black while those available in one or two genomes are given in blue and violet, respectively. The letter prefixes in sequence names indicate the genomes where they are present (s, German Shepherd; d, Great Dane; and b, Boxer). The functional terminators and poly(A) tails are marked red and yellow, respectively. TSDs are underlined. **B.** Analysis of the chr1_10744779 tribe demonstrating their $T^{+}$ retrotransposition. The descriptors of the copies include the left coordinate, terminator sequence, and poly(A) length. The left box (ancestor) includes copies present in the three dog breeds. The right boxes include daughter copies

found in one or two breeds (copies found in two breeds are given in the same color).
