# Supplementary material for: Tail Wags Dog’s SINE: Retropositional Mechanisms of Can SINE Depend on Its A-Tail Structure
Source: Biology (Basel). 2022 Sep 26;11(10):1403. doi: 10.3390/biology11101403 (PMC9599045; doi:10.3390/biology11101403)
Supplement: Supplementary file 1 [file biology-11-01403-s001.zip › Table S1.pdf]

Table S1. Examples of mutation-inducing Alu insertions into human genes.

Gene	Alu subfamily	Poly(A) tail	References
CD40LG	Yb8	A_{13}	Apoil *et al.* (2007)
EYA1	Ya5	A_{97} mother<br>A_{37} daughter	Abdelhak *et al.* (1997)
FGFR2	Ya5 case 1<br>Yb8 case 2	A_{69}<br>A_{44-50}	Oldridge *et al.* (1999)
ChE	Yb8	A_{38}	Muratani *et al.,* (1991)
C1 inhibitor	Y	A_{57}	Stoppa-Lyonnet *et al.* (1991)
BRCA2	Y	A_{64}	Miki *et al.* (1996)
GK	Y	A_{74}	Zhang *et al.* (2000)
CLCN5	Ya5	A_{48-53}	Claverie-Martin *et al.* (2003)
HESX1	Yb8	A_{31}	Sobrier *et al.,* (2005)
CFTR ex 16 ex 17b	Y<br>Ya5	A_{57}<br>A_{56}	Chen *et al.* (2007)
PTEN	Ya5<br>Yb8	A_{40}<br>A_{31}	Crivelli *et al.* (2017)
ATP7A	Ya5a2	A_{89}	Gu *et al.* (2007)
PKLR	Yb9	A_{72}	Lesmana *et al.* (2018)
LPL	YB9	$A_{>60}$	Okubo *et al.* (2007)
GNPTAB	???	A_{17}	Tappino *et al.* (2007)
FGFR2	Yk13	A_{12}	Bochukova *et al.* (2009)
FBP1	Ya5	A_{49}	Ramakrishna *et al.,* (2017)
POMT1	Ya5	$A_{\sim 53}$	Bouchet *et al.* (2007)
OPA1	Yb8	A_{25}	Gallus *et al.* (2010)
APC	Yb8	A_{42}	Halling *et al.* (1999)
BTK	Y	A_{74}	Conley *et al.* (2005)
CAR	Ya5	A_{92-94}	Janicic *et al.* (1995)
CRB1	Y	$A_{>70}$	den Hollander *et al.* (1999)
F9	Ya/Yb	A_{40}	Li *et al.* (2001)
HMBS	Ya5	A_{39}	Mustajoki *et al.* (1999)
NF1	Ya5	$A_{>40}$	Wallace *et al.* (1991)
TNFRSF6	AluYa5	A_{34}	Tighe *et al.* (2002)

F8	AluYb8	A47	Sukarova *et al.* (2001)

References

91. Abdelhak, S.; Kalatzis, V.; Heilig, R.; Compain, S.; Samson, D.; Vincent, C.; Levi-Acobas, F.; Cruaud, C.; Le Merrer, M.; Mathieu, M.; et al. Clustering of mutations responsible for branchio-oto-renal (BOR) syndrome in the eyes absent homologous region (eyaHR) of EYA1. *Hum. Mol. Genet.* **1997**, *6*, 2247–2255. https://doi.org/10.1093/hmg/6.13.2247.
92. Apoil, P.A.; Kuhlein, E.; Robert, A.; Rubie, H.; Blancher, A. HIGM syndrome caused by insertion of an AluYb8 element in exon 1 of the CD40LG gene. *Immunogenetics* **2006**, *59*, 17–23. https://doi.org/10.1007/s00251-006-0175-5.
93. Bochukova, E.G.; Roscioli, T.; Hedges, D.J.; Taylor, I.B.; Johnson, D.; David, D.J.; Deininger, P.L.; Wilkie, A.O. Rare mutations ofFGFR2causing apert syndrome: identification of the first partial gene deletion, and anAluelement insertion from a new subfamily. *Hum. Mutat.* **2009**, *30*, 204–211. https://doi.org/10.1002/humu.20825.
94. Bouchet, C.; Vuillaumier-Barrot, S.; Gonzales, M.; Boukari, S.; Le Bizec, C.; Fallet, C.; Delezoide, A.-L.; Moirot, H.; Laquerriere, A.; Encha-Razavi, F.; et al. Detection of an Alu insertion in the POMT1 gene from three French Walker Warburg syndrome families. *Mol. Genet. Metab.* **2007**, *90*, 93–96. https://doi.org/10.1016/j.ymgme.2006.09.005.
95. Chen, J.-M.; Masson, E.; Macek, M.; Raguénès, O.; Piskackova, T.; Fercot, B.; Fila, L.; Cooper, D.N.; Audrézet, M.-P.; Férec, C. Detection of two Alu insertions in the CFTR gene. *J. Cyst. Fibros.* **2008**, *7*, 37–43. https://doi.org/10.1016/j.jcf.2007.04.001.
96. Claverie-Martin, F.; González-Acosta, H.; Flores, C.; Antón-Gamero, M.; García-Nieto, V. *De novo* insertion of an Alu sequence in the coding region of the CLCN5 gene results in Dent's disease. *Hum. Genet.* **2003**, *113*, 480–485. https://doi.org/s00439-003-0991-8.
97. Conley, M.E.; Partain, J.D.; Norland, S.M.; Shurtleff, S.A.; Kazazian, H.H. Two independent retrotransposon insertions at the same site within the coding region of BTK. *Hum. Mutat.* **2005**, *25*, 324–325. https://doi.org/10.1002/humu.9321.
98. Crivelli, L.; Bubien, V.; Jones, N.; Chiron, J.; Bonnet, F.; Barouk-Simonet, E.; Couzigou, P.; Sevenet, N.; Caux, F.; Longy, M. Insertion of Alu elements at a PTEN hotspot in Cowden syndrome. *Eur. J. Hum. Genet.* **2017**, *25*, 1087–1091. https://doi.org/10.1038/ejhg.2017.81
99. Hollander, A.I.D.; Brink, J.B.T.; De Kok, Y.J.; Van Soest, S.; Born, L.I.V.D.; Van Driel, M.A.; Van De Pol, D.J.; Payne, A.; Bhattacharya, S.S.; Kellner, U.; et al. Mutations in a human homologue of Drosophila crumbs cause retinitis pigmentosa (RP12). *Nat. Genet.* **1999**, *23*, 217–221. https://doi.org/10.1038/13848.
100. Gallus, G.N.; Cardaioli, E.; Rufa, A.; Da Pozzo, P.; Bianchi, S.; D'Eramo, C.; Collura, M.; Tumino, M.; Pavone, L.; Federico, A. Alu-element insertion in an OPA1 intron sequence associated with autosomal dominant optic atrophy. *Mol. Vis.* **2010**, *16*, 178–183.
101. Gu, Y.; Kodama, H.; Watanabe, S.; Kikuchi, N.; Ishitsuka, I.; Ozawa, H.; Fujisawa, C.; Shiga, K. The first reported case of Menkes disease caused by an Alu insertion mutation. *Brain Dev.* **2007**, *29*, 105–108.
102. Halling, K.C.; Lazzaro, C.R.; Honchel, R.; Bufill, J.A.; Powell, S.M.; Arndt, C.A.; Lindor, N.M. Hereditary Desmoid Disease in a Family with a Germline Alu I Repeat Mutation of the APC Gene. *Hum. Hered.* **1999**, *49*, 97–102. https://doi.org/10.1159/000022852.
103. Janicic, N.; Pausova, Z.; Cole, D.E.; Hendy, G.N. Insertion of an Alu sequence in the Ca(2+)-sensing receptor gene in familial hypocalciuric hypercalcemia and neonatal severe hyperparathyroidism. *Am. J. Hum. Genet.* **1995**, *56*, 880-886.
104. Lesmana, H.; Dyer, L.; Li, X.; Denton, J.; Griffiths, J.; Chonat, S.; Seu, K.G.; Heeney, M.M.; Zhang, K.; Hopkin, R.J.; et al. Alu element insertion inPKLRgene as a novel cause of pyruvate kinase deficiency in Middle Eastern patients. *Hum. Mutat.* **2018**, *39*, 389–393. https://doi.org/10.1002/humu.23392.
105. Li, X.; Scaringe, W.A.; Hill, K.; Roberts, S.; Mengos, A.; Careri, D.; Pinto, M.T.; Kasper, C.K.; Sommer, S.S. Frequency of recent retrotransposition events in the human factor IX gene. *Hum. Mutat.* **2001**, *17*, 511–519. https://doi.org/10.1002/humu.1134.
106. Miki, Y.; Katagiri, T.; Kasumi, F.; Yoshimoto, T.; Nakamura, Y. Mutation analysis in the BRCA2 gene in primary breast cancers. *Nat. Genet.* **1996**, *13*, 245–247. https://doi.org/10.1038/ng0696-245.
107. Muratani, K.; Hada, T.; Yamamoto, Y.; Kaneko, T.; Shigeto, Y.; Ohue, T.; Furuyama, J.; Higashino, K. Inactivation of the cholinesterase gene by Alu insertion: possible mechanism for human gene transposition. *Proc. Natl. Acad. Sci.* **1991**, *88*, 11315–11319. https://doi.org/10.1073/pnas.88.24.11315.
108. Mustajoki, S.; Ahola, H.; Mustajoki, P.; Kauppinen, R. Insertion of Alu element responsible for acute intermittent porphyria. *Hum. Mutat.* **1999**, *13*, 431–438.

109. Okubo, M.; Horinishi, A.; Saito, M.; Ebara, T.; Endo, Y.; Kaku, K.; Murase, T.; Eto, M. A novel complex deletion–insertion mutation mediated by Alu repetitive elements leads to lipoprotein lipase deficiency. *Mol. Genet. Metab.* **2007**, *92*, 229–233. https://doi.org/10.1016/j.ymgme.2007.06.018.
110. Oldridge, M.; Zackai, E.H.; McDonald-McGinn, D.M.; Iseki, S.; Morriss-Kay, G.M.; Twigg, S.R.; Johnson, D.; Wall, S.A.; Jiang, W.; Theda, C.; et al. De Novo Alu-Element Insertions in FGFR2 Identify a Distinct Pathological Basis for Apert Syndrome. *Am. J. Hum. Genet.* **1999**, *64*, 446–461. https://doi.org/10.1086/302245.
111. Ramakrishna, S.H.; Patil, S.J.; Jagadish, A.A.; Sapare, A.K.; Sagar, H.; Kannan, S. Fructose-1,6-bisphosphatase deficiency caused by a novel homozygous Alu element insertion in the FBP1 gene and delayed diagnosis. *J. Pediatr. Endocrinol. Metab.* **2017**, *30*, 703–706.
112. Sobrier, M.-L.; Netchine, I.; Heinrichs, C.; Thibaud, N.; Vié-Luton, M.-P.; Van Vliet, G.; Amselem, S. Alu-element insertion in the homeodomain ofHESX1and aplasia of the anterior pituitary. *Hum. Mutat.* **2005**, *25*, 503–503. https://doi.org/10.1002/humu.9332.
113. Stoppa-Lyonnet, D.; E Carter, P.; Meo, T.; Tosi, M. Clusters of intragenic Alu repeats predispose the human C1 inhibitor locus to deleterious rearrangements. *Proc. Natl. Acad. Sci.* **1990**, *87*, 1551–1555, https://doi.org/10.1073/pnas.87.4.1551.
114. Sukarova, E.; Dimovski, A.; Tchacarova, P.; Petkov, G.; Efremov, G. An Alu Insert as the Cause of a Severe Form of Hemophilia A. *Acta Haematol.* **2001**, *106*, 126–129. https://doi.org/10.1159/000046602.
115. Tappino, B.; Regis, S.; Corsolini, F.; Filocamo, M. An Alu insertion in compound heterozygosity with a microduplication in GNPTAB gene underlies Mucolipidosis II. *Mol. Genet. Metab.* **2008**, *93*, 129–133. https://doi.org/10.1016/j.ymgme.2007.09.010.
116. Tighe, P.J.; E Stevens, S.; Dempsey, S.; Le Deist, F.; Rieux-Laucat, F.; Edgar, J.D.M. Inactivation of the Fas gene by Alu insertion: retrotransposition in an intron causing splicing variation and autoimmune lymphoproliferative syndrome. *Genes Immun.* **2002**, *3*, S66–S70.
117. Wallace, M.R.; Andersen, L.B.; Saulino, A.M.; Gregory, P.E.; Glover, T.W.; Collins, F.S. A de novo Alu insertion results in neurofibromatosis type 1. *Nature* **1991**, *353*, 864–866. https://doi.org/10.1038/353864a0.
118. Zhang, Y.H.; Dipple, K.M.; Vilain, E.; Huang, B.L.; Finlayson, G.; Therrell, B.L.; Worley, K.; Deininger, P.; McCabe, E.R. AluY insertion (IVS4-52ins316alu) in the glycerol kinase gene from an individual with benign glycerol kinase deficiency. *Hum. Mutat.* **2000**, *15*, 316–323.
