# Supplementary material for: Tail Wags Dog’s SINE: Retropositional Mechanisms of Can SINE Depend on Its A-Tail Structure
Source: Biology (Basel). 2022 Sep 26;11(10):1403. doi: 10.3390/biology11101403 (PMC9599045; doi:10.3390/biology11101403)
Supplement: Supplementary file 1 [file biology-11-01403-s001.zip › Figure S11. Original image of full western blot.pdf]

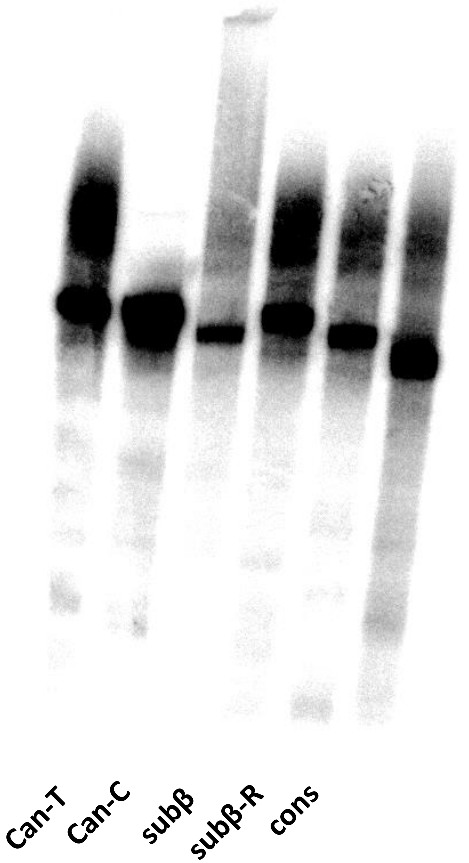
Can-T
Can-C
subβ
subβ-R
cons

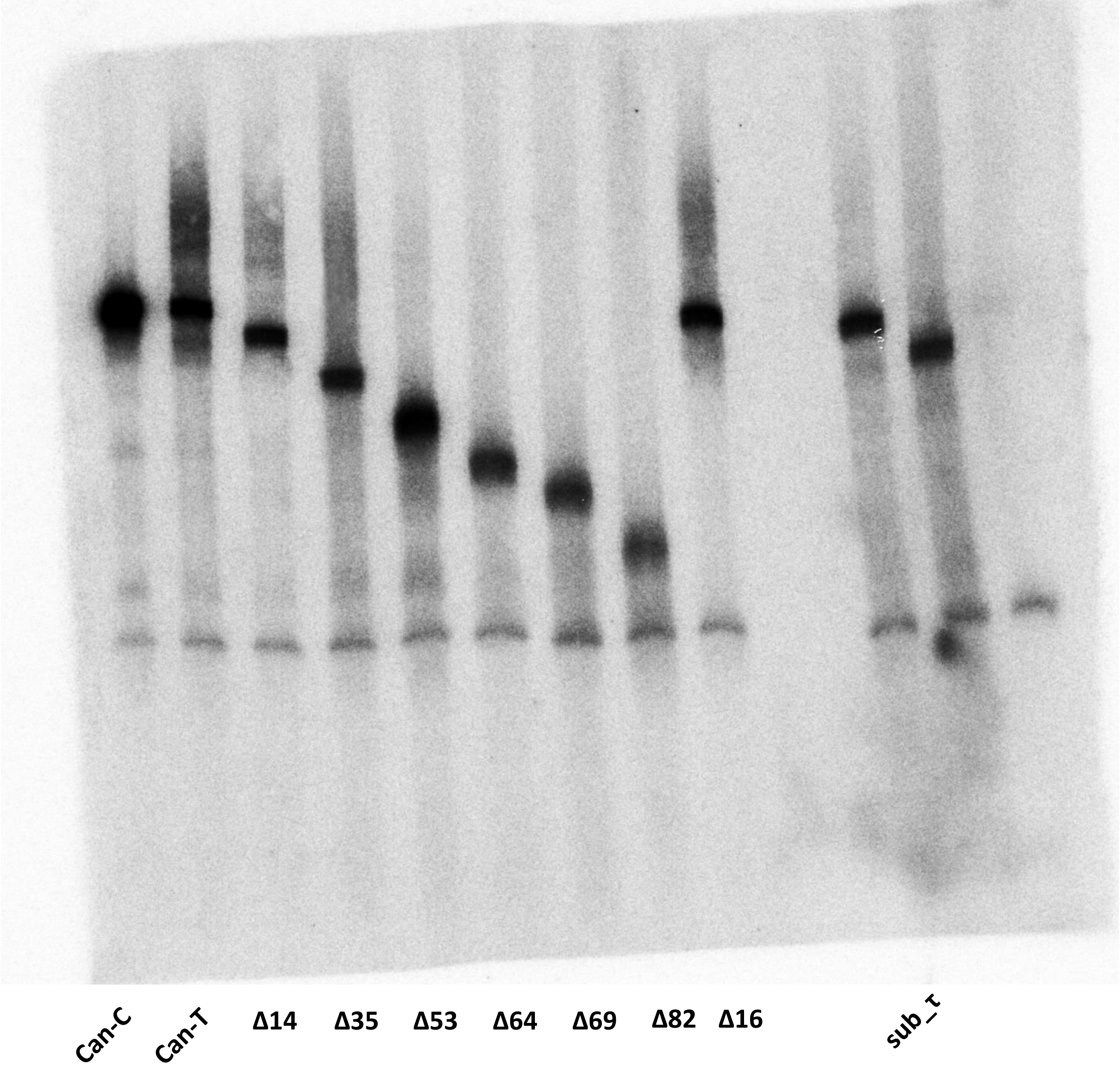
Can-C
Can-T
Δ14
Δ35
Δ53
Δ64
Δ69
Δ82
Δ16
sub_τ
